# Evolutionary analysis of mumps viruses of genotype F collected in mainland China in 2001–2015

**DOI:** 10.1038/s41598-017-17474-z

**Published:** 2017-12-07

**Authors:** Aili Cui, Pierre Rivailler, Zhen Zhu, Xiuying Deng, Ying Hu, Yan Wang, Fangcai Li, Zhaodan Sun, Jilan He, Yuan Si, Xiaoling Tian, Shujie Zhou, Yake Lei, Huanying Zheng, Paul A. Rota, Wenbo Xu

**Affiliations:** 1 WHO WPRO Regional Reference Measles/Rubella Laboratory and Key Laboratory of Medical Virology Ministry of Health, National Institute for Viral Disease Control and Prevention, China Center for Disease Control and Prevention, No. 155, Changbai Road, Changping District, Beijing 102206 People’s Republic of China; 2 Division of Viral Diseases, Centers for Disease Control and Prevention, 1600 Clifton Road Atlanta, Atlanta, GA 30329-4027 United States; 3 Jiangsu Provincial Centers for Disease Control and Prevention, No. 172, Jiangsu Road, Nanjing, 210009 The People’s Republic of China; 4 Liaoning Provincial Centers for Disease Control and Prevention, No. 242, Shayang Road, Heping District, Shenyang 110005 The People’s Republic of China; 5 Hunan Provincial Centers for Disease Control and Prevention, No. 450, Furongzhongluyiduan Road, Changsha, 410005 The People’s Republic of China; 6 Heilongjiang Provincial Centers for Disease Control and Prevention, No. 40, Youfang Road, Xiangfang District, Ha’erbin 150030 The People’s Republic of China; 7 Sichuan Provincial Centers for Disease Control and Prevention, No. 6, Zhongxue Road, Chengdu, 610041 The People’s Republic of China; 8 Shannxi Provincial Centers for Disease Control and Prevention, No. 3, Hepingwenwaijiandong Road, Xi’an, 710054 The People’s Republic of China; 9 Inner Mongolia Autonomous Region Center for Disease Control and Prevention, No. 50, E’erduosida Road, Huhehaote, 010031 The People’s Republic of China; 10 Anhui Provincial Centers for Disease Control and Prevention, No. 12560, Fanhuadadao Road, Hefei, 230601 The People’s Republic of China; 11 Hubei Provincial Centers for Disease Control and Prevention, No.6, Zhuodaoquanbeilu Road, Hongshan District, Wuhan 430079 The People’s Republic of China; 12 Guangdong Provincial Centers for Disease Control and Prevention, No. 176, Xingangxi Road, Guangzhou, 510300 The People’s Republic of China; 13 Medical school, Anhui University of Science and Technology, Huainan, 232001 People’s Republic of China

## Abstract

Mumps incidence in mainland China remains at a high level. Genotype F has been the predominant genotype of mumps virus (MuV) in the last 20 years in mainland China. To better understand the genetic characteristics of MuV in China, the sequences of the Small Hydrophobic (SH), Hemagglutinin-Neuraminidase (HN) and Fusion (F) genes of MuVs of genotype F collected during 2001–2015 were determined. The evolutionary rates of the HN and F genes were similar (0.5 × 10^−3^ substitutions/site/year) whereas the SH gene evolutionary rate was three times faster. The most recent common ancestor of genotype F was traced back to 1980. Four lineages were identified within HN and F MuV sequences. A phylogeographic analysis indicated that the genotype F viruses originally spread from the Liaoning and Shandong provinces followed by a spread to the South and East of China. This study provides important genetic baseline data for the development of prevention and control measures of mumps.

## Introduction

Mumps, caused by mumps virus (MuV), is a common infectious disease with high incidence primarily affecting children and adolescents. The main clinical symptoms are parotid gland swelling, pain and fever^1^. Mumps can be associated with pancreatitis, orchitis, deafness, aseptic meningitis or encephalitis. Mumps can be prevented by administration of live attenuated vaccine which is usually delivered as a component of measles/mumps/rubella vaccine (MMR)^2^. With the use of mumps vaccine, mumps incidence had a significant drop in countries with high vaccine coverage rates. However, some countries such as the United States are experiencing some large mumps outbreaks occurring in highly vaccinated populations^3,4^.

MuV belongs to a member of the *Rubulavirus* genus of *Paramyxoviridae* family, and is a non-segmented single-stranded negative strand RNA virus. The MuV genome contains about 15,384 nucleotides and encodes 7 viral proteins, including two surface glycoproteins, fusion (F) and hemagglutinin-neuraminidase (HN), four core proteins, nucleoprotein (NP), virion/phospho (V/P), matrix (M) and large protein (L) and the membrane associated small hydrophobic (SH) protein^1^. The World Health Organization (WHO) recommends a standard method for describing the genetic characteristics of wild type MuVs. MuVs have been divided into 12 genotypes based on the sequences of the SH and HN genes^4,5^. The standard genotyping protocol is based on SH gene sequences. According to the SH genotyping, 12 genotypes (A-N, except for E and M) have been found in the world^4^.

Even though virologic surveillance has been only routinely performed in a few countries, it appears that genotype F have had a limited circulation^4,5^. Indeed, genotype F has been mainly observed in mainland China^6,7^. It has been also detected in 10 other countries from North Americas, Europe and Asia but in most cases, an epidemiologic link to China was found^4^.

Mumps incidence maintained a high level in mainland China. Since the implementation of mumps surveillance in 2004, mumps has an average annual incidence rate of 24 per 100,000 people^6,8^. Children and adolescents are particularly susceptible to mumps, and outbreaks in kindergartens and primary schools are frequent. Genotype F has been the predominant genotype in mainland China^6,7^. The first MuV strain was isolated in mainland China in 1995^9^. Virologic surveillance for MuV was implemented beginning in 2001 as part of the measles/rubella laboratory network of mainland China. Hundreds of MuV isolates were obtained and genotyped by sequencing the SH gene. Recent analysis of SH sequences identified multiple chains of transmission in mainland China but failed to identify any geographical restrictions or chronologic correlation^7^. As Gouma *et al*. showed recently that sequences of HN and F were more evolutionary informative than SH sequences, we decided to analyze the evolution of MuVs collected in mainland China in the context of HN and F genes^10^. In this study, we showed that the sequences of the HN and F genes allowed the identification of 4 distinct lineages within MuVs collected in mainland China. Furthermore, a SPREAD analysis described the geographical spread of these lineages across Chinese provinces. Evolutionary rates and Time of Most Recent Common Ancestors (TMRCAs) were also estimated.

## Results

**Figure 1:**
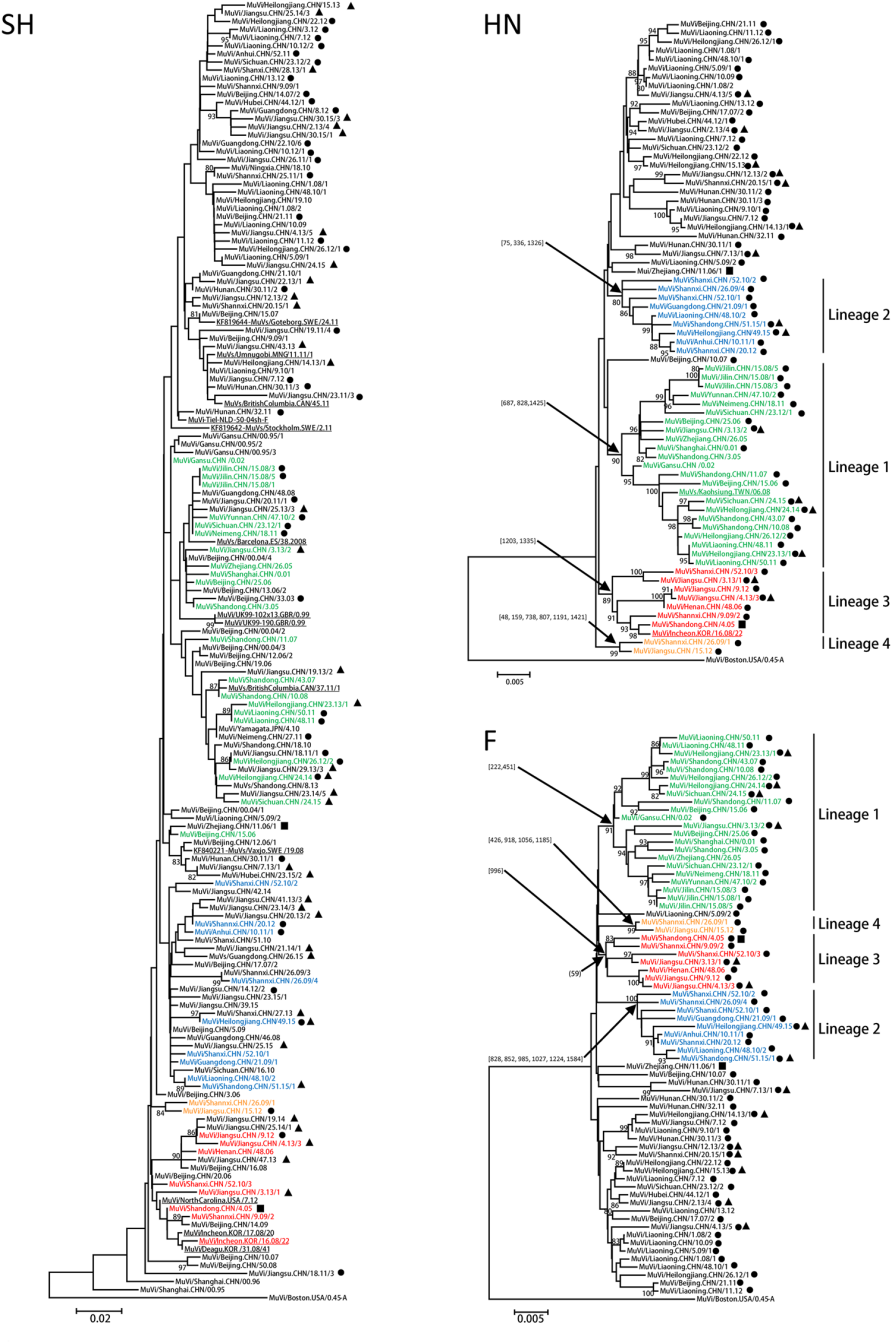
Neighbor joining phylogenetic trees based on the sequences of the 160 SH, 72 HN and 70 F genes of MuVs in genotype F. The sequences of viruses collected during 2013–2015 are identified with triangles. The sequences of MuVs not collected in China are underlined in the SH and HN trees. Sequences generated in this study are indicated by a black dot. The sequences of the two WHO reference viruses are indicated by a black square. Bootstrap values greater than 80% are indicated on the trees. When necessary, a bootstrap value less than 80% is indicated in parenthesis. SNP positions defining major nodes are indicated in brackets and are based on data from Supplementary Tables S2 and S3. Trees have been rooted with MuVi/Boston.USA/0.45 of genotype A. Sequences corresponding to the lineages identified in this study are color coded (lineage 1 in green, lineage 2 in blue, lineage 3 in red and lineage 4 in orange).

Sixty-eight MuVs collected from 2001 to 2015 from all 6 administrative regions of mainland China were selected for HN and F gene sequencing (Supplementary Table S1, Supplementary Figure S1). Phylogenetic trees were generated for SH, HN and F genes (Fig. 1). The SH tree showed a high level of diversity with an average p-distance of 3.7%. However, none of the major nodes were supported by a bootstrap value greater than 70%. As a consequence, SH sequences could not be clustered efficiently. In contrast, the HN tree featured major nodes with bootstrap values greater than 80%. At least, 4 distinct lineages (labeled 1 to 4, Fig. 1) were identified in the tree based on HN sequences. Similar lineages can be identified in the maximum likelihood tree (Supplementary Figure S2). The average p-distance between these groups was greater than 1.5%, ranging from 1.6 to 2.3% whereas the average p-distance within these groups was less than 1.5%, ranging from 0.9 to 1.4%. The highest p-distance was within lineage 1. Three of those lineages, 1, 2 and 4, were also visible in the phylogenetic tree showing the F sequences; lineage 3 was not as well supported (only 59%). Interestingly, the sequences of viruses collected in 2013, 2014 or 2015 (identified with a triangle in Fig. 1) belonged to 3 of the 4 lineages. Furthermore, viruses from all 4 lineages were detected in the region of Huadong suggesting a co-circulation of these lineages (Supplementary Table S1). It is worth noting that not all HN or F sequences were included in these lineages. Indeed, 30 sequences (42%) could not be clustered. A detailed analysis of the nucleotide differences in HN and F genes showed that the lineage-defining nodes (with bootstrap values greater than 80%) were characterized by 2 to 6 nucleotide differences (Fig. 1, Supplementary Tables S2 and S3). These nucleotide differences are specific to one lineage and are not seen in any sequences that do not belong to that lineage. Among the 14 nucleotide differences identified in the HN gene, only one resulted in an amino acid (aa) change. The Single Nucleotide Polymorphism (SNP) at position 1421, specific to lineage 4, corresponded to a A474 V mutation. Similarly, among the 13 nucleotide differences identified in the F gene, two resulted in an aa change. The SNP at position 451, specific to lineage 1, corresponded to a V151I mutation whereas the SNP at position 985, specific to lineage 2, corresponded to a H329Y mutation.

**Figure 2:**
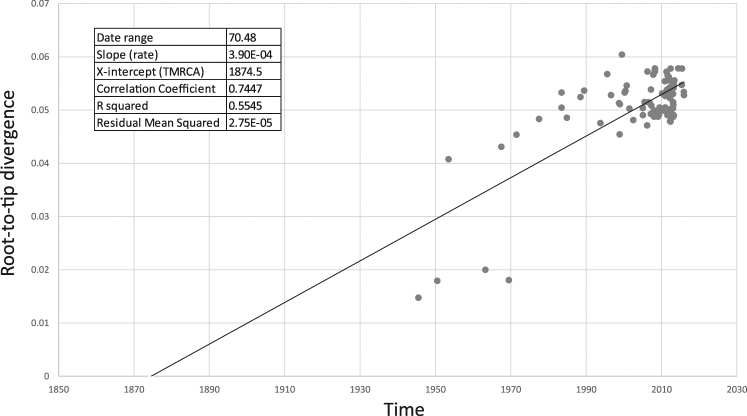
Regression of root-to-tip genetic distances against sampling date. The coefficient correlation of 0.74 and R2 of 0.55 show a positive correlation between genetic divergence and sampling time. The dataset appears therefore to be suitable for phylogenetic molecular clock analysis in BEAST or other programs.

The temporal structure of the data was tested using 95 HN sequences including 72 sequences of genotype F and 23 sequences of another genotype (Supplementary Tables S1 and S4). A regression of root-to-tip genetic distances against sampling date is shown in Fig. 2. The correlation coefficient is 0.74 and R^2^ value is 0.55. The dataset exhibited a positive correlation between genetic divergence and sampling time. The root- to- tip analysis shows that the samples collected after 2000 exhibit less dispersion around the best fit line compared to “old” samples. There are 4 outlier data points corresponding to the following viruses: MuVi/Boston.USA/0.45[A], MuVi/Kilham.USA/0.50[A], MuVi/L3_vector.RUS/0.53[N], and MuVi/Pennsylvannia.USA/13.63[A]. These old sequences are from WHO reference viruses and they have been widely used by the scientific community. As these “old” sequences are necessary for a reliable TMRCA estimation, they were kept and used for analysis even though the quality might be questionable.

The evolutionary rates of all 3 genes were estimated (Table 1), and the rates for HN and F were similar at approximately 0.5 × 10^−3^ substitutions/site/year {95% Highest Posterior Density (HPD) 0.3–0.7 × 10^−3^}. In contrast, the evolutionary rate for the SH gene was three times faster at 1.6 × 10^−3^ substitutions/site/year (95% HPD 1.17–2.21 × 10^−3^). Similarly, dN/dS value were similar for the HN and F genes, close to 0.1 but it was 6 times higher for the SH gene. These values showed a purifying selection on all 3 genes. The SH protein featured a greater variability than the HN and F proteins. Among the 160 SH sequences analyzed that code for the 58-aa protein, 49 positions (84%) were found variable. In contrast, only 16% of the aa positions were variable in the 72 582-aa HN and 70 538-aa F sequence datasets.

**Table 1:** Evolutionary rates and TMRCAs for SH, HN and F genes of MuVs from genotype F.

Evolutionary analysis		SH	HN	F
Evolutionary rate		1.59 (1.17–2.04)^1,2^	0.51 (0.3–0.73)	0.53 (0.31–0.76)
dN/dS		0.61	0.08	0.09
TMRCA	Genotype F	1980 (1967–1988)	1986 (1974–1994)	1986 (1974–1994)
	Lineage 1^3^	NA	1995 (1990–1999)	1992 (1985–1998)
	Lineage 2	NA	2003 (1998–2007)	2004 (1998–2007)
	Lineage 3	NA	1996 (1989–2002)	1999 (1991–2003)
	Lineage 4	NA	2006 (1998–2009)	2008 (2004–2009)

1: Mean values are indicated, 95% HPD values are in parenthesis.

2: Evolutionary rate is expressed as 10^−3^ substitutions/site/year.

3: HN and F lineages are identified in Fig. 1.

The TMRCA was estimated for genotype F (Table 1, Supplementary Figure S3) and was estimated to be 1986 (95% HPD 1974–1994). The F dataset gave a similar time estimate whereas the SH dataset gives a slightly earlier date, 1980 (95% HPD 1967–1988). This discrepancy might be because the SH dataset contained sequences collected as early as 1995 whereas the earliest HN and F sequences were collected in 2001 (Supplementary Figure S4). On the other hand, SH sequences might be less informative because the 316-nt long SH sequence contains less divergent sites than the 1749-nt HN or 1617-nt F sequences. The analysis of the HN dataset suggested a quasi-simultaneous establishment of lineages 1 and 3 in 1995–1996 followed by the appearance of lineages 2 and 4 in 2003–2006.

Geographic information was processed in Bayesian Evolutionary Analysis Sampling Trees (BEAST) to follow the spread of the 4 lineages over time. The output was generated in SPREAD and displayed in Google Earth (Supplementary Video S1). The time slider animation in Google Earth allowed observing the temporal spread of MuV in China. The SPREAD output showed that MuV started to spread from the Liaoning and Shandong provinces in 1987. Lineage 1 (green) spread to the west (Gansu) and the east (Shanghai), while the lineage 2 (blue) spread to the south (Beijing and Shandong) and reached Guangdong in 2008. Meanwhile, lineage 1 spread to the north (Jilin) and to the south (Yunnan). The spread of lineage 3 (red) was geographically limited, mostly in the central provinces of Shandong, Shaanxi, Hebei, and Henan. In approximately 2008, lineage 4 (orange) emerged in Shaanxi and spread east to Jiangsu. Overall, lineage 1 was the most frequently detected covering an area, from Jilin to Yunnan, and from Gansu to Zhejiang.

## Discussion

Our analysis showed that distinct lineages within HN and F genes can be identified for MuVs of genotype F collected in mainland China. This is in contrast to what is observed with the SH gene (this report^7^,). Gouma *et al*. came to the same conclusion when analyzing the sequences of genotype G strains associated with outbreaks in the Netherlands and proposed to concatenate the F, SH, and HN genes into a single sequence to increase the resolution of the molecular information^10^. The lineages identified in this study contained around 60% of the MuV sequences of genotype F. A similar outcome was obtained by Gouma *et al*. as not all HN or F sequences of genotype G fit a lineage supported by a bootstrap value greater than 70%^10^. The lineages identified in this study are characterized by a p-distance between groups greater than 1.5% and within groups less than 1.5%. These criteria, though arbitrary, are similar to those used for other viruses like influenza virus H5N1 or rubella virus^11,12^. Lineages 1 and 2 were strongly supported in HN and F trees with bootstrap greater 80% showing the robustness of such clustering. Lineages 3 and 4 were less robust as lineage 3 was not well supported in the F tree and lineage 4 was only defined by two sequences. Continuous monitoring of MuV evolution of genotype F will be necessary to test whether these lineages are stable through time.

Genotype F is known to be circulating mainly in mainland China^4,6,7^. As we reported, among the 4533 MuV sequences available in GenBank, only 268 (6%) are of genotype F and 90% were collected in mainland China. Genotype F was found in 10 other countries from North America, Europe and Asia. As Jin *et al*. reported in their review, in most cases, an epidemiologic link to China was found^4^. So, considering that genotype F is the main genotype circulating in China, which has the potential to spread globally, it is important for a global public health perspective to acquire as much knowledge as possible on this genotype. This study is the first report of lineages identified within genotype F. SH gene does not allow to differentiate between sequences^7^. HN and F genes can differentiate around 60% of sequences. 40% of sequences remain undifferentiated and it is possible that WGS data might help to differentiate these sequences. Identifying lineages is essential for molecular epidemiology which mainly tracks viruses in time and space. Lineages were identified in genotype G which is the most preponderant genotype (at least 67% of MuV sequences in GenBank) using HN and F genes^10^. It is very likely that the same type of analysis will be done for the other circulating genotypes. Unfortunately, the number of F and to a lesser extent HN sequences in GenBank is limited. WHO has recently proposed an updated nomenclature for MuV based on the SH and HN genes so it is likely that the number of HN sequences will increase soon^5^. In addition, implementation of whole genome sequencing projects should rapidly expand the sequence database for mumps.

The evolutionary rate of HN and F MuV genes were approximately 0.55 × 10^−3^ substitutions/site/year, while the rate for MuV SH was estimated to be 3 times higher, at 1.71 × 10^−3^ substitutions/site/year. These rates are comparable to rates reported for other RNA viruses. For example, the rate for the measles H gene was estimated at 0.75 × 10^−3^ substitutions/site/year^13^, the rate for rubella E1 gene was estimated to be approximately 1.5 × 10^−3^ substitutions/site/year^14^, the evolutionary rate of VP1 of human enterovirus A71 was approximately 4.5 × 10^−3^ nucleotide substitutions/site/year for sub genotype C4^15^. The capsid gene of GII.3 norovirus was reported to evolve at 4.2 × 10^−3^ substitutions/site/year^16^. The dN/dS values (0.1 for HN and F genes and 0.6 for SH gene) were similar to what was previously reported for SH gene for genotypes G (0.5–0.9), J (0.3) and F (0.8)^17^. The greater value for SH was reflected by a high variability at the aa level; 84% of the aa positions were variable in our dataset compared to 16% in HN and F proteins. This variability appeared to be random as no aa pattern was found among SH sequences. SH was recently shown to impede with NF-kB activation in MuV infected cells by decreasing the phosphorylation of IKKβ, IκBα, and p65 and the translocation of p65 into the nucleus^18^. However, it is not clear how a high aa variability would affect SH function.

Our analysis estimated the TMRCA of genotype F to be in the early 1980s, that is at least 9 years before the first reported detection of genotype F which was in 1995^9^. Our analysis also showed that the viruses of genotype F evolved sequentially, likely the consequence of separate introductions. A major lineage, lineage 1, started to spread in 1995 followed by lineage 3. In 2003, two distinct lineages 2 and 4 were initially identified. The SPREAD analysis allowed monitoring the geographic spread of the MuV in mainland China. The phylogeographic analysis revealed putative geographic linkage between mumps samples. However, due to the limited number of samples, such analysis needs to be considered carefully. As mumps surveillance is improving in mainland China, more precise analysis will be possible. This analysis indicated a spread from Liaoning and Shandong provinces to the South and the East of the country. It also demonstrated the spatial dominance of lineage 1 compared to the other lineages. Based on the TMRCA analysis, lineage 1 and lineage 3, appeared at the same period but lineage 1 seemed to be more widespread. It is not clear why lineage 1 was more widely distributed than the other 3 lineages.

In conclusion, genotype F of MuV is endemic in China; it was first identified in 1995 and continuously circulated throughout the country since then. Phylogenetic analyses of the HN and F genes identified at least 4 distinct lineages. The most recent common ancestor of genotype F may be traced back to the early 1980 s. This study provides important genetic baseline data for the development of prevention and control measures of mumps in China.

## Material and Methods

### Specimen collection and virus isolation

Mumps virologic surveillance has been performed by the China Measles/Rubella Laboratory Network in China CDC, including one national lab, 31 provincial labs and 331 prefecture labs since 2001. The range of mumps virologic surveillance covers the whole country. The throat swabs or urine were collected in hospitals by the clinic doctor or provincial CDC staff from several hundred patients with clinically suspected mumps from mumps outbreaks or sporadic cases. Isolation of MuV for these clinic specimens was performed using Vero/SLAM cells in accordance with standard protocols^19^. Cultures with cytopathic effect were tested by RT-PCR and sequencing as described below.

### RT-PCR amplification and sequence determination

Viral RNA was extracted from culture with cytopathic effect by using the QIAamp mini viral RNA extraction kit (Qiagen, USA). To obtain the complete SH, HN and F gene sequences, RT-PCR was performed with the Qiagen One-step RT-PCR kit (TaKaRa Biotechnology Dalian, China) following the manufacturer’s instructions. The primers of SH5–1(5′-AATATCAAGTAGTGTCGATGA-3′) and SH5–2 (5′-AGGTGCAAAGGTGGCATTGTC-3′) were used to amplify the entire SH gene, as described previously^20^. The primer pairs for HN and F gene (HN-forward primer: 5′ CTGTTCAATCATGAGACATAAAG-3′; HN-reverse primer: 5′-TCAGGTAAGAGTATCTCATT-3′; F-forward primer: 5′-CCTCCAGGAGGATCAACAAT-3′; F-reverse primer: 5′-TGGTTAACAGAATCCAGACATAC-3′) were designed to amplify the entire HN and F genes, respectively.

After purification of the PCR products with a QIA Gel Extraction Kit (Qiagen, Valencia, CA), the sequences were determined using the Sanger dideoxy terminator sequencing method with a BigDye Terminator Version 3.1 Cycle Sequencing kit (Life Technologies,NY, USA) and ABI PRISMTM 3100 Genetic Analyzer (Life Technologies, Japan). Sequencher software version 5.0 (Gene Codes Corporation) was used to edit and assemble the raw sequence data to obtain the 316-nt complete SH gene sequences, the 1749-nt of complete HN gene sequences, and the 1617-nt of complete F gene sequences. Sequences of 44 SH, 63 HN and 68 F genes were submitted to GenBank (Supplementary Table S1, GenBank IDs of the new sequences are shown in red font).

### Dataset

4533 MuV sequences were downloaded from GenBank. Two hundred sixty-eight sequences were of F genotype including 8 full genomes, 250 sequences corresponding to the sequencing window containing SH gene, and 9 HN sequences. Two hundred twenty-one of those sequences were unique (47 SH sequences were identical). One hundred ninety-eight sequences were from viruses collected in mainland China. The other 23 sequences came from viruses collected in 10 countries (in the decreasing order of number of sequences, Korea, Canada, Taiwan, Sweden, Spain, United Kingdom, the United States, Japan, Mongolia and the Netherlands). Datasets were generated for each gene, SH, HN and F. The sequences generated in this report were aligned with sequences of genotype F viruses downloaded from GenBank including sequences from viruses collected outside mainland China for which no epidemiologic link to mainland China was available (Supplementary Table S1). Sequences were aligned using MUSCLE^21^ in BioEdit (www.mbio.ncsu.edu/BioEdit/bioedit.html) and duplicate sequences were discarded. Sequences of viruses of non-genotype F, collected before 1995, the year when the first genotype F virus was detected, were added to the dataset for the BEAST analysis (see below) (Supplementary Table S4). The resulting datasets used in this analysis are the SH dataset with 175 sequences (144 from mainland China, 15 from another country and 16 non-F sequences), the HN dataset with 95 sequences (70 from mainland China, 2 from another country and 23 non-F sequences), and the F dataset with 80 sequences (70 from mainland China and 10 non-F sequences) (Supplementary Tables S1 and S4). All three datasets contained sequences of MuVs collected in all 6 regions (Supplementary Figure S1).

### Phylogenetic analyses

Each dataset was used to build a neighbor joining phylogenetic tree^22^ with Mega5^23^ using the maximum composite likelihood nucleotide substitution model^24^. The topology of the phylogenetic tree was tested with 1000 bootstrap replications^25^. Bootstrap values greater than 80% were indicated on the trees. Maximum likelihood tree was also generated with Mega. p-distances were computed in Mega. The number of synonymous nucleotide substitutions per synonymous site (dS) and the number of nonsynonymous substitutions per nonsynonymous site (dN) were estimated in Mega by Nei and Gojobori’s method^26^.

### Estimation of the TMRCA and evolutionary rate and analysis of geographic spread

The same datasets were analyzed with the BEAST package (version 1.7.5) to estimate the evolutionary rate and the TMRCA of genotype F and related lineages as well as to investigate the spread of MuV in mainland China across time. Initially, a nucleotide substitution model was analyzed with Jmodeltest (version 0.1)^27^; the GTR model (general time reversible model) + I (proportion of invariable sites) + G (gamma distribution shape parameter) was determined as the best-fit nucleotide substitution model for all the datasets. The temporal structure of the data was tested with TempEst^28^. Three different molecular clock models were implemented in the BEAST analysis, strict clock, uncorrelated exponential relaxed clock, and the uncorrelated log-normal relaxed clock. The uncorrelated exponential relaxed clock model was considered the best model for the three datasets using the Bayes factor test in Tracer (version 1.5). Finally, the latitude and longitude for each collection site was added to the model (http://www.findlatitudeandlongitude.com/). After fixing the clock model, three independent analyses were performed and combined using LogCombiner software included in the BEAST package. The Markov Chain Monte Carlo method was performed for 50 to 200 million generations and sampled so that 10,000 trees were saved for further analysis. After 10% burn-in and reaching an effective sample size of at least 200 for relevant analysis parameters, a Maximum Clade Credibility tree was created using TreeAnnotator and edited in FigTree (Version 1.4.0, http://tree.bio.ed.ac.uk/software/figtree/) (Supplementary Figure S5). Phylogeographic analysis was generated in SPREAD (www.phylogeography.org/SPREAD, version 1.0.6) and displayed in Google Earth. The output file was manually edited with Kml color converter (version 2.0, http://www.sgrillo.net/kml_color/) to feature lineages identified in Fig. 1. Bayes Factor (BF) tests were run in SPREAD on each geographical links (Supplementary Figure S6). A BF >3 designates a well-supported diffusion route in the migration graph^29^. The higher the BF value is, the more likely that a migration may exist between two locations.

### Ethics statement

In this study, the only human materials used were collected from the clinically suspected mumps patients for the purpose of public health and disease control. This study was approved by the second session of the Ethics Review Committee of the National Institute for Viral Disease Control and Prevention in the China CDC and the methods were performed in accordance with the approved guidelines. Written informed consent for the use of the clinical specimens was obtained from all of the patients involved in this study.

The findings and conclusions in this report are those of authors and do not necessarily represent the views of the U.S. Department of Health and Human Services.

## Electronic supplementary material

Supplementary Information

Supplementary Video
