# Supplementary material for: Evolutionary analysis of mumps viruses of genotype F collected in mainland China in 2001–2015
Source: Sci Rep. 2017 Dec 7;7:17144. doi: 10.1038/s41598-017-17474-z (PMC5719434; doi:10.1038/s41598-017-17474-z)
Supplement: Supplementary file 1 — Supplementary Information [file 41598_2017_17474_MOESM1_ESM.pdf]

Evolutionary analysis of mumps viruses of genotype F collected in mainland China in 2001-2015

Aili Cui [1+], Pierre Rivailler [1,2+], Zhen Zhu [1], Xiuying Deng[3], Ying Hu[3], Yan Wang[4], Fangcai Li[5], Zhaodan Sun[6], Jilan He[7], Yuan Si[8], Xiaoling Tian[9], Shujie Zhou[10], Yake Lei[11], Huanying Zheng[12], Paul A. Rota [2*] and Wenbo Xu [1,13*].

Supplementary table S1: list of sequences of MuVs of genotype F analyzed in this study

Strains	Year	Region	Province	City	Country	SH (GenBank ID) *	Lineage based on HN gene	HN (GenBank ID) *	F (GenBank ID) *
MuVi/Shanghai.CHN/00.95	1995	Huadong	Shanghai	Shanghai	China	Z77160	/	/	/
MuVi/Gansu.CHN/00.95/1	1995	Xibei	Gansu	/	China	Z77158	/	/	/
MuVi/Gansu.CHN/00.95/3	1995	Xibei	Gansu	/	China	Z77159	/	/	/
MuVi/Gansu.CHN/00.95/2	1995	Xibei	Gansu	/	China	Z77161	/	/	/
MuVi/Shanghai.CHN/00.96	1996	Huadong	Shanghai	Shanghai	China	Z81005	/	/	/
MuVi/UK99-102x13.GBR/0.99	1999	/	/	/	Great Britain	AY380065	/	/	/
MuVi/UK99-190.GBR/0.99	1999	/	/	/	Great Britain	AY380068	/	/	/
MuVi/UK99-102x13.GBR/0.99	1999	/	/	/	United Kingdom	AY380065	/	/	/
MuVi/UK99-190.GBR/0.99	1999	/	/	/	United Kingdom	AY380068	/	/	/
MuVi/Shanghai.CHN/0.01	2001	Huadong	Shanghai	Shanghai	China	KF022153	Lineage 1	KX690531	KY680471
MuVi/Gansu.CHN/0.02	2002	Xibei	Gansu	Lanzhou	China	KF022144	Lineage 1	JQ034459	KY680472
MuVi/Beijing.CHN/33.03	2003	Huabei	Beijing	Beijing	China	KX690580	/	/	/
MuVi/Tiel.NLD/50.04	2004	/	/	Tiel	Netherlands	KF876756	/	/	/
MuVi/Beijing.CHN/00.04/2	2004	Huabei	Beijing	Beijing	China	EU780217	/	/	/
MuVi/Beijing.CHN/00.04/4	2004	Huabei	Beijing	Beijing	China	EU780218	/	/	/
MuVi/Beijing.CHN/00.04/1	2004	Huabei	Beijing	Beijing	China	EU780219	/	/	/
MuVi/Beijing.CHN/00.04/3	2004	Huabei	Beijing	Beijing	China	EU780220	/	/	/
MuVi/Zhejiang.CHN/26.05	2005	Huadong	Zhejiang	/	China	KF170916	Lineage 1	KF170916	KF170916
MuVi/Shandong.CHN/3.05	2005	Huadong	Shandong	Liaocheng	China	KF022143	Lineage 1	JQ034462	KX690500
MuVi/Shandong.CHN/4.05	2005	Huadong	Shandong	Liaocheng	China	EU780221	Lineage 3	JQ034463	KX690501
MuVi/Beijing.CHN/19.06	2006	Huabei	Beijing	Beijing	China	KF022119	/	/	/
MuVi/Beijing.CHN/3.06	2006	Huabei	Beijing	Beijing	China	KF022120	/	/	/
MuVi/Beijing.CHN/12.06/2	2006	Huabei	Beijing	Beijing	China	KF022129	/	/	/
MuVi/Beijing.CHN/12.06/1	2006	Huabei	Beijing	Beijing	China	KF022131	/	/	/
MuVi/Beijing.CHN/13.06/2	2006	Huabei	Beijing	Beijing	China	KF022136	/	/	/
MuVi/Beijing.CHN/20.06	2006	Huabei	Beijing	Beijing	China	KF022140	/	/	/
MuVi/Beijing.CHN/15.06	2006	Huabei	Beijing	Beijing	China	KF022138	Lineage 1	KX690533	KY680473
MuVi/Beijing.CHN/25.06	2006	Huabei	Beijing	Beijing	China	KF022142	Lineage 1	KX690534	KY680474
MuVi/Zhejiang.CHN/11.06/1	2006	Huadong	Zhejiang	/	China	JQ945272	/	JQ946034	KF170917
MuVi/Henan.CHN/48.06	2006	Zhongnan	Henan	/	China	KF022145	Lineage 3	KX690535	KY680490
MuVi/Beijing.CHN/15.07	2007	Huabei	Beijing	Beijing	China	KF022135	/	/	/
MuVi/Beijing.CHN/14.07/2	2007	Huabei	Beijing	Beijing	China	KX690581	/	/	/
MuVi/Beijing.CHN/10.07	2007	Huabei	Beijing	Beijing	China	KF022127	/	KX690536	KY680464
MuVi/Beijing.CHN/17.07/2	2007	Huabei	Beijing	Beijing	China	KF022139	/	KX690537	KY680465
MuVi/Shandong.CHN/11.07	2007	Huadong	Shandong	Jinan	China	KF022146	Lineage 1	KX690538	KX690502
MuVi/Shandong.CHN/43.07	2007	Huadong	Shandong	Jinan	China	KF022147	Lineage 1	KX690539	KX690503

Strains	Year	Region	Province	City	Country	SH (GenBank ID) *	Lineage based on HN gene	HN (GenBank ID) *	F (GenBank ID) *
MuVi/Deagu.KOR/31.08/41	2008	/	/	/	South Korea	KP212949	/	/	/
MuVi/Incheon.KOR/16.08/22	2008	/	/	/	South Korea	KP212954	/	/	/
MuVi/Incheon.KOR/17.08/20	2008	/	/	/	South Korea	KP212956	/	/	/
MuVs/Barcelona.ES/38.2008	2008	/	/	/	Spain	KX609893	/	/	/
MuVi/Jilin.CHN/15.08/5	2008	Dongbei	Jilin	Changchun	China	KY680437	Lineage 1	KX690540	KY680475
MuVi/Jilin.CHN/15.08/3	2008	Dongbei	Jilin	Changchun	China	KY680438	Lineage 1	KX690541	KX690505
MuVi/Jilin.CHN/15.08/1	2008	Dongbei	Jilin	Changchun	China	KF022148	Lineage 1	KY680447	KX690504
MuVi/Liaoning.CHN/1.08/2	2008	Dongbei	Liaoning	Shenyang	China	KF022150	/	JQ034460	KX690507
MuVi/Liaoning.CHN/1.08/1	2008	Dongbei	Liaoning	Shenyang	China	KF022149	/	JQ034461	KX690508
MuVi/Beijing.CHN/50.08	2008	Huabei	Beijing	Beijing	China	KF022110	/	/	/
MuVi/Beijing.CHN/16.08	2008	Huabei	Beijing	Beijing	China	KF022133	/	/	/
MuVi/Shandong.CHN/10.08	2008	Huadong	Shandong	Jinan	China	KF022151	Lineage 1	KX690543	KX690509
MuVi/Guangdong.CHN/46.08	2008	Zhongnan	Guangdong	Shaoguan	China	KF022156	/	/	/
MuVi/Guangdong.CHN/48.08	2008	Zhongnan	Guangdong	Shaoguan	China	KF022157	/	/	/
MuVs/Vaxjo.SWE/19.08	2008	/	/	Vaxjo	Sweden	KF840221	/	/	/
MuVi/Liaoning.CHN/10.09	2009	Dongbei	Liaoning	Shenyang	China	KF022164	/	KX690546	KX690510
MuVi/Liaoning.CHN/5.09/1	2009	Dongbei	Liaoning	Shenyang	China	KF022165	/	KX690547	KX690511
MuVi/Liaoning.CHN/5.09/2	2009	Dongbei	Liaoning	Shenyang	China	KF022166	/	KX690548	KX690512
MuVi/Beijing.CHN/5.09	2009	Huabei	Beijing	Beijing	China	KF022112	/	/	/
MuVi/Beijing.CHN/9.09/1	2009	Huabei	Beijing	Beijing	China	KF022113	/	/	/
MuVi/Beijing.CHN/14.09	2009	Huabei	Beijing	Beijing	China	KF022115	/	/	/
MuVi/Shannxi.CHN/9.09/1	2009	Xibei	Shannxi	/	China	KF022159	/	/	/
MuVi/Shannxi.CHN/26.09/3	2009	Xibei	Shannxi	/	China	KF022163	/	/	/
MuVi/Shannxi.CHN/26.09/4	2009	Xibei	Shannxi	/	China	KF022158	Lineage 2	KX690549	KY680483
MuVi/Shannxi.CHN/9.09/2	2009	Xibei	Shannxi	/	China	KF022160	Lineage 3	KX690550	KX690513
MuVi/Shannxi.CHN/26.09/1	2009	Xibei	Shannxi	/	China	KF022161	Lineage 4	KX690551	KX690514
MuVi/Guangdong.CHN/21.09/1	2009	Zhongnan	Guangdong	Shaoguan	China	KF022167	Lineage 2	KX690545	KY680482
MuVi/Yamagata.JPN/4.10	2010	/	/	/	Japan	LC034329	/	/	/
MuVi/Heilongjiang.CHN/19.10	2010	Dongbei	Heilongjiang	Qiqihaer	China	KF022123	/	/	/
MuVi/Liaoning.CHN/48.10/1	2010	Dongbei	Liaoning	Shenyang	China	KF022103	/	KX690552	KY680466
MuVi/Liaoning.CHN/48.10/2	2010	Dongbei	Liaoning	Shenyang	China	KF022104	Lineage 2	KX690553	KY680484
MuVi/Liaoning.CHN/9.10/1	2010	Dongbei	Liaoning	Shenyang	China	KF022124	/	KX690554	KX690515
MuVi/Shanxi.CHN/51.10	2010	Huabei	Shanxi	/	China	KF022105	/	/	/
MuVi/Shanxi.CHN/52.10/1	2010	Huabei	Shanxi	/	China	KF022106	Lineage 2	KX690555	KY680485
MuVi/Shanxi.CHN/52.10/2	2010	Huabei	Shanxi	/	China	KF022107	Lineage 2	KX690556	KY680486
MuVi/Shanxi.CHN/52.10/3	2010	Huabei	Shanxi	/	China	KF022108	Lineage 3	KX690557	KY680491
MuVi/Shandong.CHN/18.10	2010	Huadong	Shandong	Jinan	China	KF022152	/	/	/
MuVi/Ningxia.CHN/18.10	2010	Xibei	Ningxia	/	China	KF022117	/	/	/

Strains	Year	Region	Province	City	Country	SH (GenBank ID) *	Lineage based on HN gene	HN (GenBank ID) *	F (GenBank ID) *
MuVi/Yunnan.CHN/47.10/2	2010	Xinan	Yunnan	Dehong	China	KY680439	Lineage 1	KX690558	KY680476
MuVi/Sichuan.CHN/16.10	2010	Xinan	Sichuan	Neijiang	China	KF022118	/	/	/
MuVi/Guangdong.CHN/21.10/1	2010	Zhongnan	Guangdong	Qingyuan	China	KF022121	/	/	/
MuVi/Guangdong.CHN/22.10/6	2010	Zhongnan	Guangdong	Qingyuan	China	KX690582	/	/	/
MuVs/BritishColumbia.CAN/37.11/1	2011	/	/	/	Canada	JQ042708	/	/	/
MuVs/BritishColumbia.CAN/45.11	2011	/	/	/	Canada	JQ783115	/	/	/
MuVs/BritishColumbia.CAN/37.11/1	2011	/	British Columbia	/	Canada	JQ042708	/	/	/
MuVs/BritishColumbia.CAN/45.11	2011	/	British Columbia	/	Canada	JQ783115	/	/	/
MuVs/Goteborg.SWE/24.11	2011	/	/	Goteborg	Sweden	KF819644	/	/	/
MuVs/Stockholm.SWE/2.11	2011	/	/	Stockholm	Sweden	KF819642	/	/	/
MuVs/Umnugobi.MNG/11.11/1	2011	/	/	/	Mongolia	KF477295	/	/	/
MuVi/Liaoning.CHN/50.11	2011	Dongbei	Liaoning	Shenyang	China	KY680441	Lineage 1	KX690564	KX690516
MuVi/Liaoning.CHN/48.11	2011	Dongbei	Liaoning	Shenyang	China	KX690595	Lineage 1	KX690565	KX690517
MuVi/Neimeng.CHN/18.11	2011	Huabei	Neimeng	Alashanmeng	China	KY680440	Lineage 1	KX690566	KY680477
MuVi/Beijing.CHN/21.11	2011	Huabei	Beijing	Beijing	China	KY680446	/	KY680456	KY680494
MuVi/Neimeng.CHN/27.11	2011	Huabei	Neimeng	Chifeng	China	KX690596	/	/	/
MuVi/Anhui.CHN/52.11	2011	Huadong	Anhui	Anqing	China	KX690583	/	/	/
MuVi/Anhui.CHN/10.11/1	2011	Huadong	Anhui	Hefei	China	KX690584	Lineage 2	KX690559	KY680487
MuVi/Jiangsu.CHN/18.11/1	2011	Huadong	Jiangsu	Nanjing	China	KX690589	/	/	/
MuVi/Jiangsu.CHN/18.11/3	2011	Huadong	Jiangsu	Nanjing	China	KX690590	/	/	/
MuVi/Jiangsu.CHN/19.11/4	2011	Huadong	Jiangsu	Nanjing	China	KX690591	/	/	/
MuVi/Jiangsu.CHN/20.11/1	2011	Huadong	Jiangsu	Nanjing	China	KX690592	/	/	/
MuVi/Jiangsu.CHN/23.11/3	2011	Huadong	Jiangsu	Nanjing	China	KX690593	/	/	/
MuVi/Jiangsu.CHN/26.11/1	2011	Huadong	Jiangsu	Nanjing	China	KX690594	/	/	/
MuVi/Shannxi.CHN/25.11/1	2011	Xibei	Shannxi	Yanan	China	KX690597	/	/	/
MuVi/Hunan.CHN/30.11/1	2011	Zhongnan	Hunan	Changsha	China	KX690585	/	KX690560	KY680467
MuVi/Hunan.CHN/30.11/2	2011	Zhongnan	Hunan	Changsha	China	KX690586	/	KX690561	KY680468
MuVi/Hunan.CHN/30.11/3	2011	Zhongnan	Hunan	Changsha	China	KX690587	/	KX690562	KY680469
MuVi/Hunan.CHN/32.11	2011	Zhongnan	Hunan	Huaihua	China	KX690588	/	KX690563	KY680470
MuVi/North Carolina.USA/7.12	2012	/	/	/	United States	KF143772	/	/	/
MuVi/Liaoning.CHN/3.12	2012	Dongbei	Liaoning	/	China	KX690605	/	/	/
MuVi/Heilongjiang.CHN/26.12/2	2012	Dongbei	Heilongjiang	Haerbin	China	KY680442	Lineage 1	KX690567	KX690518
MuVi/Heilongjiang.CHN/22.12	2012	Dongbei	Heilongjiang	Haerbin	China	KX690599	/	KX690568	KX690519
MuVi/Heilongjiang.CHN/26.12/1	2012	Dongbei	Heilongjiang	Haerbin	China	KX690600	/	KX690569	KX690520
MuVi/Liaoning.CHN/10.12/1	2012	Dongbei	Liaoning	Shenyang	China	KX690606	/	/	/

Supplementary Table S2: Listing of SNPs featured in the 72 HN sequences of MuVs of genotype F

Lineage [1]	Strain name	12 [2]	16	18	22	24	25	26	27	28	30	33	34	35	38	39	42	46	48 [3]
2	MuVi/Shanxi.CHN/52.10/2	G	C	C	A	A	A	T	A	T	G	C	A	A	C	C	C	G	A
2	MuVi/Shannxi.CHN/26.09/4	G	C	C	G	A	A	T	A	T	G	C	A	A	C	C	C	G	A
2	MuVi/Shanxi.CHN/52.10/1	G	C	C	A	A	A	T	A	T	G	C	A	A	C	C	C	G	A
2	MuVi/Guangdong.CHN/21.09/1	G	C	C	A	A	G	T	A	T	G	C	A	A	C	C	C	G	A
2	MuVi/Liaoning.CHN/48.10/2	G	C	C	A	A	A	T	A	T	G	C	A	A	C	C	C	G	A
2	MuVi/Shandong.CHN/51.15/1	G	C	C	A	A	A	T	A	T	G	C	A	A	C	C	C	G	A
2	MuVi/Heilongjiang.CHN/49.15	G	C	C	A	A	A	T	A	T	G	C	A	A	C	C	C	G	A
2	MuVi/Anhui.CHN/10.11/1	G	C	C	A	A	A	T	A	T	G	C	A	A	C	C	C	G	A
2	MuVi/Shannxi.CHN/20.12	G	C	C	A	A	A	T	A	T	G	C	A	A	C	C	C	G	A
3	MuVi/Shanxi.CHN/52.10/3	G	C	C	A	A	A	T	A	T	G	C	A	A	C	C	C	G	A
3	MuVi/Jiangsu.CHN/3.13/1	G	C	C	A	A	A	T	A	T	G	C	A	A	C	C	C	G	A
3	MuVi/Shandong.CHN/4.05	G	C	C	A	A	A	T	A	T	G	C	A	A	C	C	C	G	A
3	MuVi/Shannxi.CHN/9.09/2	A	C	C	A	A	A	T	A	T	G	C	G	G	C	C	C	G	A
3	MuVi/Incheon.KOR/16.08/22	G	C	C	A	A	A	T	A	T	G	C	A	A	C	C	C	G	A
3	MuVi/Henan.CHN/48.06	G	C	C	A	A	A	T	A	T	G	C	A	A	C	C	C	G	A
3	MuVi/Jiangsu.CHN/9.12	G	C	C	A	A	A	T	A	T	G	C	A	A	C	C	C	G	A
3	MuVi/Jiangsu.CHN/4.13/3	G	C	C	A	A	A	T	A	T	G	C	A	A	C	C	C	G	A
4	MuVi/Shannxi.CHN/26.09/1	G	C	C	A	A	A	T	A	T	G	C	A	A	C	C	C	G	G
4	MuVi/Jiangsu.CHN/15.12	G	C	C	A	A	A	T	A	T	G	C	A	A	C	C	C	G	G
1	MuVi/Jilin.CHN/15.08/5	A	C	C	A	A	A	T	G	T	A	C	A	A	C	C	C	G	A
1	MuVi/Jilin.CHN/15.08/1	A	C	C	A	A	A	T	G	T	A	C	A	A	C	C	C	G	A
1	MuVi/Jilin.CHN/15.08/3	A	C	C	A	A	A	T	G	T	A	C	A	A	C	C	C	G	A
1	MuVi/Yunnan.CHN/47.10/2	A	C	C	A	A	A	T	A	T	A	C	A	A	C	C	C	G	A
1	MuVi/Neimeng.CHN/18.11	A	C	C	A	A	A	T	A	T	A	C	A	A	C	C	C	G	A
1	MuVi/Sichuan.CHN/23.12/1	A	C	C	A	A	A	T	A	T	A	C	A	A	C	C	C	G	A
1	MuVi/Jiangsu.CHN/3.13/2	A	C	C	A	A	A	T	A	T	G	C	A	A	C	C	C	G	A
1	MuVi/Beijing.CHN/25.06	A	C	A	A	A	A	T	A	T	G	C	A	A	C	C	C	G	A
1	MuVi/Zhejiang.CHN/26.05	A	C	C	A	A	A	T	A	C	G	C	A	A	C	C	C	G	A
1	MuVi/Shanghai.CHN/0.01	A	C	C	A	A	A	T	A	T	G	C	A	A	C	C	C	G	A
1	MuVi/Shandong.CHN/3.05	A	T	C	A	A	A	T	A	T	G	C	A	A	C	C	C	G	A
1	MuVi/Gansu.CHN/0.02	A	C	C	A	A	A	T	A	T	G	C	A	A	C	C	C	G	A
1	MuVi/Beijing.CHN/15.06	A	C	C	A	A	A	T	A	T	G	C	A	A	C	C	C	G	A
1	MuVi/Shandong.CHN/11.07	A	C	C	A	A	A	T	A	T	G	C	A	A	C	C	C	G	A
1	MuVi/Sichuan.CHN/24.15	A	C	C	A	A	A	T	A	T	G	C	A	A	C	C	C	G	A
1	MuVi/Heilongjiang.CHN/24.14	A	C	C	A	A	A	T	A	T	G	C	A	A	C	C	C	G	A
1	MuVi/Shandong.CHN/43.07	A	C	C	A	A	C	T	A	T	G	C	A	A	C	C	C	G	A
1	MuVi/Shandong.CHN/10.08	A	C	C	A	A	A	T	A	T	G	C	A	A	C	C	C	G	A
1	MuVi/Heilongjiang.CHN/26.12/2	A	C	C	A	A	A	T	A	T	G	C	A	A	C	C	C	G	A
1	MuVi/Liaoning.CHN/48.11	A	C	C	A	A	A	T	A	T	G	C	A	A	C	C	C	G	A
1	MuVi/Heilongjiang.CHN/23.13/1	A	C	C	A	A	A	T	A	T	G	C	A	A	C	C	C	G	A
1	MuVi/Liaoning.CHN/50.11	A	C	C	A	A	A	T	A	T	G	C	A	A	C	C	C	G	A
1	MuVs/Kaohsiung.TWN/06.08	A	C	C	A	A	A	T	A	T	G	C	A	A	C	C	C	G	A
	MuVi/Jiangsu.CHN/12.13/2	G	T	C	A	A	A	T	A	T	G	C	A	A	C	C	T	G	A
	MuVi/Shannxi.CHN/20.15/1	G	T	C	A	A	A	T	A	T	G	C	A	A	C	C	T	G	A
	MuVi/Beijing.CHN/10.07	G	C	C	A	A	A	T	A	T	G	C	A	A	C	C	C	G	A
	MuVi/Hunan.CHN/30.11/3	G	T	C	A	A	A	T	A	T	G	C	A	A	C	C	C	G	A
	MuVi/Liaoning.CHN/9.10/1	G	T	C	A	A	A	T	A	T	G	C	A	A	C	C	C	G	A
	MuVi/Jiangsu.CHN/7.12	G	T	C	A	A	A	T	A	T	G	C	A	A	C	C	C	A	A
	MuVi/Heilongjiang.CHN/14.13/1	G	T	C	A	A	A	T	A	T	G	C	A	A	C	C	C	A	A
	MuVi/Hunan.CHN/32.11	C	T	C	A	A	A	T	A	T	G	T	A	A	C	C	C	G	A
	MuVi/Hunan.CHN/30.11/1	G	C	C	A	A	A	T	A	G	G	T	A	A	C	C	C	G	A
	MuVi/Jiangsu.CHN/7.13/1	G	C	C	A	A	A	T	A	G	G	C	A	A	C	C	C	G	A
	MuVi/Hunan.CHN/30.11/2	G	T	C	A	A	A	T	A	T	G	C	A	A	C	C	T	G	A
	MuVi/Liaoning.CHN/5.09/2	G	C	C	A	A	A	T	A	T	G	C	A	A	C	C	T	G	A
	Mui/Zhejiang.CHN/11.06/1	G	C	C	A	A	A	T	A	T	G	C	A	A	C	C	C	G	A
	MuVi/Liaoning.CHN/13.12	G	T	C	A	A	A	C	A	T	G	C	A	A	C	C	C	G	A
	MuVi/Beijing.CHN/17.07/2	G	T	C	A	A	A	T	A	T	G	C	A	A	C	C	C	G	A
	MuVi/Liaoning.CHN/7.12	G	T	C	A	C	A	T	A	T	G	C	A	A	C	C	C	G	A
	MuVi/Beijing.CHN/21.11	G	T	C	A	A	A	T	A	T	G	C	A	A	C	T	C	G	A
	MuVi/Liaoning.CHN/11.12	G	T	C	A	A	A	T	A	T	G	C	A	A	C	T	C	G	A
	MuVi/Heilongjiang.CHN/26.12/1	G	T	C	A	A	A	T	A	T	G	C	A	A	C	C	C	G	A
	MuVi/Liaoning.CHN/1.08/1	G	T	C	A	A	A	T	A	T	G	C	A	A	C	C	C	G	A
	MuVi/Liaoning.CHN/48.10/1	G	T	C	A	A	A	T	A	T	G	C	A	A	C	C	C	G	A
	MuVi/Jiangsu.CHN/4.13/5	G	T	C	A	A	A	T	A	T	G	T	A	A	C	C	C	G	A
	MuVi/Liaoning.CHN/10.09	G	T	C	A	A	A	T	A	T	G	C	A	A	C	C	C	G	A
	MuVi/Liaoning.CHN/1.08/2	G	T	C	A	A	A	T	A	T	G	C	A	A	C	C	C	G	A
	MuVi/Liaoning.CHN/5.09/1	G	T	C	A	A	A	T	A	T	G	C	A	A	C	C	C	G	A
	MuVi/Heilongjiang.CHN/22.12	G	T	C	A	A	A	T	A	T	G	C	A	A	C	C	C	G	A
	MuVi/Heilongjiang.CHN/15.13	G	T	C	A	A	A	T	A	T	G	C	A	A	T	C	C	G	A
	MuVi/Sichuan.CHN/23.12/2	G	T	C	A	A	A	T	A	T	G	C	A	A	C	C	C	G	A
	MuVi/Hubei.CHN/44.12/1	G	T	C	A	A	A	T	A	T	G	C	A	A	C	C	C	G	A
	MuVi/Jiangsu.CHN/2.13/4	G	T	C	A	A	A	T	A	T	G	C	A	A	C	C	C	G	A

1: Lineage based on Figure 1
2: SNPs are noted in green.
3: SNP position corresponding to a lineage-defining node in Figure 1 are indicated in green, blue, red or orange according to Figure 1.

Supplementary Table S2: Listing of SNPs featured in the 7

Lineage [1]	Strain name	50	54	55	57	58	59	60	61	67	70	73	74	75	93	99	106	108	109
2	MuVi/Shanxi.CHN/52.10/2	C	G	C	T	G	T	T	A	G	G	G	A	T	C	C	T	G	G
2	MuVi/Shannxi.CHN/26.09/4	C	G	C	T	G	T	T	A	G	G	G	A	T	C	C	T	G	G
2	MuVi/Shanxi.CHN/52.10/1	C	G	C	T	G	T	T	A	G	G	G	A	T	C	C	T	G	A
2	MuVi/Guangdong.CHN/21.09/1	C	G	C	T	G	T	T	A	G	G	A	A	T	C	C	T	G	G
2	MuVi/Liaoning.CHN/48.10/2	C	G	C	T	G	T	T	A	G	G	A	A	T	C	C	T	G	G
2	MuVi/Shandong.CHN/51.15/1	C	G	C	T	G	T	T	A	G	G	A	A	T	C	C	T	G	G
2	MuVi/Heilongjiang.CHN/49.15	C	G	C	T	G	T	T	A	G	G	A	A	T	C	C	T	G	G
2	MuVi/Anhui.CHN/10.11/1	C	G	C	T	G	T	T	A	G	G	A	A	T	C	C	T	G	G
2	MuVi/Shannxi.CHN/20.12	C	G	C	T	G	T	T	A	G	G	A	A	T	C	C	T	G	G
3	MuVi/Shanxi.CHN/52.10/3	C	G	C	T	G	C	T	A	G	G	G	A	C	C	C	T	G	G
3	MuVi/Jiangsu.CHN/3.13/1	C	G	C	T	G	C	T	A	G	G	G	A	C	C	C	T	G	G
3	MuVi/Shandong.CHN/4.05	C	G	C	T	G	T	T	A	G	G	G	A	C	C	C	T	G	G
3	MuVi/Shannxi.CHN/9.09/2	C	G	C	T	G	T	T	A	G	G	G	A	C	C	C	T	G	G
3	MuVi/Incheon.KOR/16.08/22	C	G	C	T	G	T	T	A	G	G	G	A	C	C	C	T	G	G
3	MuVi/Henan.CHN/48.06	C	G	C	T	G	T	T	A	G	G	G	A	C	C	C	T	G	G
3	MuVi/Jiangsu.CHN/9.12	C	G	C	T	G	T	T	A	G	G	G	A	C	C	C	T	G	G
3	MuVi/Jiangsu.CHN/4.13/3	C	G	C	T	G	T	T	A	G	G	G	A	C	C	C	T	G	G
4	MuVi/Shannxi.CHN/26.09/1	C	G	C	T	G	C	C	A	G	A	G	A	C	C	C	T	G	G
4	MuVi/Jiangsu.CHN/15.12	C	G	C	T	G	C	T	A	G	G	G	A	C	C	C	T	G	G
1	MuVi/Jilin.CHN/15.08/5	C	G	C	T	A	T	T	A	G	G	G	A	C	C	C	T	G	G
1	MuVi/Jilin.CHN/15.08/1	C	G	C	T	G	T	T	A	G	G	G	A	C	C	C	T	G	G
1	MuVi/Jilin.CHN/15.08/3	C	G	C	T	G	T	T	A	G	G	G	A	C	C	C	T	G	G
1	MuVi/Yunnan.CHN/47.10/2	T	G	C	T	G	T	T	A	G	G	G	A	C	C	C	T	G	G
1	MuVi/Neimeng.CHN/18.11	C	G	C	T	G	T	T	A	G	G	G	A	C	C	C	T	G	G
1	MuVi/Sichuan.CHN/23.12/1	C	G	C	T	G	T	T	G	G	G	G	A	C	C	C	T	G	G
1	MuVi/Jiangsu.CHN/3.13/2	C	G	C	T	G	T	T	A	G	G	G	A	C	C	C	T	G	G
1	MuVi/Beijing.CHN/25.06	C	G	C	T	G	C	T	A	G	G	G	A	C	C	C	T	G	G
1	MuVi/Zhejiang.CHN/26.05	C	G	T	T	G	T	T	A	G	G	G	A	C	C	C	T	G	G
1	MuVi/Shanghai.CHN/0.01	C	G	C	T	G	T	T	A	G	G	G	A	C	C	C	T	G	G
1	MuVi/Shandong.CHN/3.05	C	G	C	T	G	T	T	A	G	G	G	A	C	C	C	T	G	G
1	MuVi/Gansu.CHN/0.02	C	G	C	T	G	T	T	A	G	G	G	A	C	C	C	T	G	G
1	MuVi/Beijing.CHN/15.06	C	G	C	T	G	T	T	A	G	G	G	A	C	C	C	T	G	G
1	MuVi/Shandong.CHN/11.07	C	G	C	T	G	T	T	A	G	G	G	G	C	C	C	T	G	G
1	MuVi/Sichuan.CHN/24.15	C	G	C	T	G	T	T	A	G	G	G	A	C	C	C	T	G	G
1	MuVi/Heilongjiang.CHN/24.14	C	G	C	T	G	T	T	A	G	G	G	A	C	C	C	T	G	G
1	MuVi/Shandong.CHN/43.07	C	G	C	T	G	T	T	A	G	G	G	A	C	C	C	T	G	G
1	MuVi/Shandong.CHN/10.08	C	G	C	T	G	T	T	A	G	G	G	A	C	C	C	T	G	G
1	MuVi/Heilongjiang.CHN/26.12/2	C	G	C	T	G	T	T	A	G	G	G	A	C	C	C	T	G	G
1	MuVi/Liaoning.CHN/48.11	C	G	C	T	G	T	T	A	G	G	G	A	C	C	C	T	G	G
1	MuVi/Heilongjiang.CHN/23.13/1	C	G	C	T	G	T	T	A	G	G	G	A	C	C	C	T	G	G
1	MuVi/Liaoning.CHN/50.11	C	G	C	T	G	T	T	A	G	G	G	A	C	C	C	T	G	G
1	MuVs/Kaohsiung.TWN/06.08	C	G	C	T	G	T	T	A	G	G	G	A	C	C	C	T	A	G
	MuVi/Jiangsu.CHN/12.13/2	C	G	C	T	G	T	T	A	G	G	G	A	C	C	C	T	G	G
	MuVi/Shannxi.CHN/20.15/1	C	G	C	T	G	T	T	A	G	G	G	A	C	C	C	T	G	G
	MuVi/Beijing.CHN/10.07	C	G	C	T	G	T	T	A	A	G	G	A	C	C	C	T	G	G
	MuVi/Hunan.CHN/30.11/3	C	G	C	T	G	T	T	A	G	G	G	A	C	A	C	C	A	G
	MuVi/Liaoning.CHN/9.10/1	C	G	C	T	G	T	T	A	G	G	G	A	C	A	C	C	G	G
	MuVi/Jiangsu.CHN/7.12	C	G	C	T	G	T	T	A	G	G	G	A	C	A	C	C	G	G
	MuVi/Heilongjiang.CHN/14.13/1	C	G	C	T	G	T	T	A	G	G	G	A	C	A	C	C	G	G
	MuVi/Hunan.CHN/32.11	C	G	C	T	G	T	T	A	T	G	G	A	C	C	C	T	G	G
	MuVi/Hunan.CHN/30.11/1	C	G	C	T	G	T	T	A	G	G	G	A	C	C	C	T	G	G
	MuVi/Jiangsu.CHN/7.13/1	C	G	C	T	G	T	T	A	G	G	G	A	C	C	C	T	G	G
	MuVi/Hunan.CHN/30.11/2	C	G	C	T	G	T	T	A	G	G	G	A	C	C	C	T	G	G
	MuVi/Liaoning.CHN/5.09/2	C	G	C	T	G	T	T	A	G	G	G	A	C	C	C	T	G	G
	Mui/Zhejiang.CHN/11.06/1	C	G	C	T	G	T	T	A	G	G	G	A	C	C	C	T	G	G
	MuVi/Liaoning.CHN/13.12	C	G	C	T	G	C	C	A	G	G	G	A	C	C	C	T	G	G
	MuVi/Beijing.CHN/17.07/2	C	G	C	T	G	T	T	A	G	G	G	A	C	C	C	T	G	G
	MuVi/Liaoning.CHN/7.12	C	G	C	T	G	T	T	A	G	G	G	A	C	C	C	T	G	G
	MuVi/Beijing.CHN/21.11	C	G	C	T	G	T	T	A	G	G	G	A	C	C	T	T	G	G
	MuVi/Liaoning.CHN/11.12	C	G	C	T	G	T	T	A	G	G	G	A	C	C	T	T	G	G
	MuVi/Heilongjiang.CHN/26.12/1	C	A	C	C	G	T	T	A	G	G	G	A	C	C	T	T	G	G
	MuVi/Liaoning.CHN/1.08/1	C	G	C	T	G	T	T	A	G	G	G	A	C	C	T	T	G	G
	MuVi/Liaoning.CHN/48.10/1	C	G	C	T	G	T	T	A	G	G	G	A	C	C	T	T	G	G
	MuVi/Jiangsu.CHN/4.13/5	C	G	C	T	G	T	T	A	G	G	G	A	C	C	T	T	G	G
	MuVi/Liaoning.CHN/10.09	C	G	C	T	G	T	T	A	G	G	G	A	C	C	T	T	G	G
	MuVi/Liaoning.CHN/1.08/2	C	G	C	T	G	T	T	A	G	G	G	A	C	C	T	T	G	G
	MuVi/Liaoning.CHN/5.09/1	C	G	C	T	G	T	T	A	G	G	G	A	C	C	T	T	G	G
	MuVi/Heilongjiang.CHN/22.12	C	G	C	T	G	T	T	A	G	G	G	A	C	C	C	T	G	G
	MuVi/Heilongjiang.CHN/15.13	C	G	C	T	G	T	T	A	G	G	G	A	C	C	C	T	G	G
	MuVi/Sichuan.CHN/23.12/2	C	G	C	T	T	T	T	A	G	G	G	A	C	C	C	T	G	G
	MuVi/Hubei.CHN/44.12/1	C	G	C	T	G	T	T	A	G	G	G	A	C	C	C	T	G	G
	MuVi/Jiangsu.CHN/2.13/4	C	G	C	T	G	T	T	A	G	G	G	A	C	C	C	T	G	G

1: Lineage based on Figure 1
2: SNPs are noted in green.
3: SNP position corresponding to a lineage-defining node ir

Supplementary Table S2: Listing of SNPs featured in the 7

Lineage [1]	Strain name	112	114	125	126	128	132	136	138	144	152	153	157	159	166	171
2	MuVi/Shanxi.CHN/52.10/2	C	G	C	T	T	C	A	G	C	C	T	G	T	G	G
2	MuVi/Shannxi.CHN/26.09/4	C	G	C	C	T	C	A	A	C	C	T	A	T	G	G
2	MuVi/Shanxi.CHN/52.10/1	C	G	C	T	T	C	A	A	C	C	T	G	T	G	G
2	MuVi/Guangdong.CHN/21.09/1	C	G	C	T	T	C	A	A	C	T	T	G	T	G	G
2	MuVi/Liaoning.CHN/48.10/2	C	G	C	T	T	C	A	A	C	C	T	G	T	G	G
2	MuVi/Shandong.CHN/51.15/1	C	G	C	T	T	C	A	A	C	C	T	G	T	G	G
2	MuVi/Heilongjiang.CHN/49.15	C	G	C	T	T	C	A	A	C	C	T	G	T	G	G
2	MuVi/Anhui.CHN/10.11/1	C	G	C	T	T	C	A	A	C	C	T	G	T	G	G
2	MuVi/Shannxi.CHN/20.12	C	G	C	T	T	C	A	A	C	C	T	G	T	G	G
3	MuVi/Shanxi.CHN/52.10/3	T	G	C	T	T	C	A	A	C	C	T	G	T	G	G
3	MuVi/Jiangsu.CHN/3.13/1	C	G	C	T	T	C	A	A	C	C	T	G	T	G	G
3	MuVi/Shandong.CHN/4.05	C	G	C	T	T	C	A	A	C	C	T	G	T	G	T
3	MuVi/Shannxi.CHN/9.09/2	C	G	C	T	T	C	A	A	C	C	T	G	T	G	G
3	MuVi/Incheon.KOR/16.08/22	C	G	C	T	T	C	A	A	C	C	T	G	T	G	G
3	MuVi/Henan.CHN/48.06	C	G	C	T	T	C	A	A	C	C	T	G	T	G	G
3	MuVi/Jiangsu.CHN/9.12	C	G	C	T	T	C	A	A	C	C	T	G	T	G	G
3	MuVi/Jiangsu.CHN/4.13/3	C	G	C	T	T	C	A	A	C	C	T	G	T	G	G
4	MuVi/Shannxi.CHN/26.09/1	C	G	C	T	T	C	A	A	C	C	T	G	C	G	G
4	MuVi/Jiangsu.CHN/15.12	C	G	C	T	T	C	A	A	C	C	T	G	C	G	G
1	MuVi/Jilin.CHN/15.08/5	C	G	C	T	T	T	A	A	C	C	T	G	T	G	G
1	MuVi/Jilin.CHN/15.08/1	C	G	C	T	T	T	A	A	C	C	T	G	T	G	G
1	MuVi/Jilin.CHN/15.08/3	C	G	C	T	T	T	A	A	C	C	T	G	T	G	G
1	MuVi/Yunnan.CHN/47.10/2	C	G	C	T	T	T	A	A	C	C	T	G	T	G	G
1	MuVi/Neimeng.CHN/18.11	C	G	C	T	T	T	A	A	C	C	T	G	T	G	G
1	MuVi/Sichuan.CHN/23.12/1	C	G	C	T	T	T	A	A	C	C	T	G	T	G	G
1	MuVi/Jiangsu.CHN/3.13/2	C	G	C	T	T	T	A	A	C	C	T	G	T	G	G
1	MuVi/Beijing.CHN/25.06	C	G	C	T	T	T	A	A	C	C	T	G	T	G	G
1	MuVi/Zhejiang.CHN/26.05	C	G	C	T	T	T	A	A	C	C	T	G	T	G	G
1	MuVi/Shanghai.CHN/0.01	C	G	C	T	T	T	A	A	C	C	T	G	T	G	G
1	MuVi/Shandong.CHN/3.05	C	G	C	T	T	T	A	A	C	C	T	G	T	G	G
1	MuVi/Gansu.CHN/0.02	C	G	C	T	T	C	A	A	C	C	T	G	T	G	G
1	MuVi/Beijing.CHN/15.06	C	G	C	T	T	C	A	A	C	C	T	G	T	G	G
1	MuVi/Shandong.CHN/11.07	C	G	C	T	C	C	A	A	C	C	T	G	T	G	G
1	MuVi/Sichuan.CHN/24.15	C	G	C	T	T	C	A	A	C	C	T	G	T	G	G
1	MuVi/Heilongjiang.CHN/24.14	C	G	T	T	T	C	A	A	C	C	T	G	T	G	G
1	MuVi/Shandong.CHN/43.07	C	G	C	T	T	C	A	A	C	C	T	G	T	G	G
1	MuVi/Shandong.CHN/10.08	C	G	C	T	T	C	A	A	C	C	T	G	T	G	G
1	MuVi/Heilongjiang.CHN/26.12/2	C	G	C	T	T	C	A	A	C	C	T	G	T	G	G
1	MuVi/Liaoning.CHN/48.11	C	G	C	T	T	C	A	A	C	C	G	G	T	G	G
1	MuVi/Heilongjiang.CHN/23.13/1	C	G	C	T	T	C	A	A	C	C	G	G	T	G	G
1	MuVi/Liaoning.CHN/50.11	C	G	C	T	T	C	A	A	C	C	G	G	T	G	G
1	MuVs/Kaohsiung.TWN/06.08	C	G	C	T	T	C	A	A	C	C	T	G	T	G	G
	MuVi/Jiangsu.CHN/12.13/2	C	G	C	T	T	C	A	A	C	C	T	G	T	G	G
	MuVi/Shannxi.CHN/20.15/1	C	G	C	T	T	C	A	A	C	C	T	G	T	G	G
	MuVi/Beijing.CHN/10.07	T	G	C	T	T	C	A	A	C	C	T	G	T	G	G
	MuVi/Hunan.CHN/30.11/3	C	G	C	T	T	C	A	A	C	C	T	G	T	G	G
	MuVi/Liaoning.CHN/9.10/1	C	G	C	T	T	C	A	A	C	C	T	G	T	G	G
	MuVi/Jiangsu.CHN/7.12	C	G	C	T	T	C	A	A	C	C	T	G	T	G	G
	MuVi/Heilongjiang.CHN/14.13/1	C	G	C	T	T	C	A	A	C	C	T	G	T	G	G
	MuVi/Hunan.CHN/32.11	C	G	C	T	T	C	A	A	C	C	T	G	T	G	G
	MuVi/Hunan.CHN/30.11/1	C	A	C	T	T	C	A	A	C	C	T	G	T	T	G
	MuVi/Jiangsu.CHN/7.13/1	C	A	C	T	T	C	A	A	C	C	T	G	T	T	G
	MuVi/Hunan.CHN/30.11/2	C	G	C	T	T	C	A	A	C	C	T	G	T	G	G
	MuVi/Liaoning.CHN/5.09/2	C	G	C	T	T	C	A	A	C	C	T	G	T	G	G
	Mui/Zhejiang.CHN/11.06/1	C	G	C	T	T	C	A	A	C	C	T	G	T	G	G
	MuVi/Liaoning.CHN/13.12	C	G	C	T	T	C	A	A	C	C	T	G	T	G	G
	MuVi/Beijing.CHN/17.07/2	C	G	C	T	T	C	A	A	C	C	T	G	T	G	G
	MuVi/Liaoning.CHN/7.12	C	G	C	T	T	C	A	A	T	C	T	G	T	G	G
	MuVi/Beijing.CHN/21.11	C	G	C	T	T	C	G	A	C	C	T	G	T	G	G
	MuVi/Liaoning.CHN/11.12	C	G	C	T	T	C	A	A	C	C	T	G	T	G	G
	MuVi/Heilongjiang.CHN/26.12/1	C	G	C	T	T	C	G	A	C	C	T	G	T	G	G
	MuVi/Liaoning.CHN/1.08/1	C	G	C	T	T	C	A	A	C	C	T	G	T	G	G
	MuVi/Liaoning.CHN/48.10/1	C	G	C	T	T	C	A	A	C	C	T	G	T	G	G
	MuVi/Jiangsu.CHN/4.13/5	C	G	C	T	T	C	A	A	C	C	T	G	T	G	G
	MuVi/Liaoning.CHN/10.09	C	G	C	T	T	C	A	A	C	C	T	G	T	G	G
	MuVi/Liaoning.CHN/1.08/2	C	G	C	T	T	C	A	A	C	C	T	G	T	G	G
	MuVi/Liaoning.CHN/5.09/1	C	G	C	T	T	C	A	A	C	C	T	G	T	G	G
	MuVi/Heilongjiang.CHN/22.12	C	G	C	T	T	C	A	A	C	C	T	G	T	G	G
	MuVi/Heilongjiang.CHN/15.13	C	G	C	T	T	C	A	A	C	C	T	G	T	G	G
	MuVi/Sichuan.CHN/23.12/2	C	G	C	T	T	C	A	A	C	C	T	G	T	G	G
	MuVi/Hubei.CHN/44.12/1	C	G	C	T	T	C	A	A	C	C	T	G	T	G	G
	MuVi/Jiangsu.CHN/2.13/4	T	G	C	T	T	C	A	A	C	C	T	G	T	G	G

1: Lineage based on Figure 1
2: SNPs are noted in green.
3: SNP position corresponding to a lineage-defining node ir

Supplementary Table S2: Listing of SNPs featured in the 7

Lineage [1]	Strain name	180	189	193	195	196	198	202	216	217	219	227	241	242	252	253
2	MuVi/Shanxi.CHN/52.10/2	T	T	A	T	A	T	T	A	A	C	G	A	T	T	G
2	MuVi/Shannxi.CHN/26.09/4	T	C	A	T	A	T	T	A	A	C	G	A	T	T	G
2	MuVi/Shanxi.CHN/52.10/1	T	C	A	T	A	T	T	A	A	C	G	A	T	T	G
2	MuVi/Guangdong.CHN/21.09/1	T	C	A	T	A	T	T	A	A	C	G	A	T	T	G
2	MuVi/Liaoning.CHN/48.10/2	T	C	A	T	A	T	T	A	A	C	G	A	T	T	G
2	MuVi/Shandong.CHN/51.15/1	T	C	A	T	A	T	T	A	A	C	G	A	T	T	G
2	MuVi/Heilongjiang.CHN/49.15	T	C	A	T	A	T	T	A	A	C	G	A	T	T	G
2	MuVi/Anhui.CHN/10.11/1	T	C	A	T	A	T	T	A	A	C	G	A	T	T	G
2	MuVi/Shannxi.CHN/20.12	T	C	A	T	A	T	T	A	A	C	G	A	T	T	G
3	MuVi/Shanxi.CHN/52.10/3	T	C	A	C	A	T	T	A	A	C	G	A	T	T	G
3	MuVi/Jiangsu.CHN/3.13/1	T	C	A	C	A	T	T	A	A	C	G	A	T	T	G
3	MuVi/Shandong.CHN/4.05	T	C	A	T	A	T	T	A	A	C	G	A	T	T	G
3	MuVi/Shannxi.CHN/9.09/2	T	C	A	T	A	T	T	A	A	C	G	A	T	T	G
3	MuVi/Incheon.KOR/16.08/22	T	C	A	T	A	T	T	A	A	C	G	A	T	T	G
3	MuVi/Henan.CHN/48.06	T	C	A	T	G	C	T	A	A	C	G	A	T	T	G
3	MuVi/Jiangsu.CHN/9.12	T	C	A	T	G	C	T	A	A	C	G	A	T	T	G
3	MuVi/Jiangsu.CHN/4.13/3	T	C	A	T	G	C	T	A	A	C	G	A	T	T	G
4	MuVi/Shannxi.CHN/26.09/1	T	C	A	T	A	T	T	A	A	C	G	A	T	T	G
4	MuVi/Jiangsu.CHN/15.12	T	C	A	T	A	T	T	A	A	C	G	A	T	T	G
1	MuVi/Jilin.CHN/15.08/5	T	C	A	T	A	T	T	A	A	C	G	A	T	T	G
1	MuVi/Jilin.CHN/15.08/1	T	C	A	T	A	T	T	A	A	C	G	A	T	T	G
1	MuVi/Jilin.CHN/15.08/3	T	C	A	T	A	T	T	A	A	C	G	A	T	T	G
1	MuVi/Yunnan.CHN/47.10/2	T	C	A	T	A	T	T	A	A	C	G	A	T	T	G
1	MuVi/Neimeng.CHN/18.11	T	C	A	T	A	T	T	A	A	C	G	A	T	T	G
1	MuVi/Sichuan.CHN/23.12/1	C	C	A	T	A	T	T	A	A	C	G	A	T	T	G
1	MuVi/Jiangsu.CHN/3.13/2	T	C	A	T	A	T	T	A	A	C	G	A	T	T	G
1	MuVi/Beijing.CHN/25.06	T	C	A	T	A	T	T	A	A	C	G	A	T	T	G
1	MuVi/Zhejiang.CHN/26.05	T	C	A	T	A	T	T	A	A	C	G	A	C	T	G
1	MuVi/Shanghai.CHN/0.01	T	C	A	T	A	T	T	A	A	C	G	G	T	T	G
1	MuVi/Shandong.CHN/3.05	T	C	A	T	A	T	T	A	A	C	G	G	T	T	G
1	MuVi/Gansu.CHN/0.02	T	C	A	T	A	T	T	A	A	C	G	A	T	T	G
1	MuVi/Beijing.CHN/15.06	T	C	A	T	A	T	C	A	A	C	A	A	T	T	G
1	MuVi/Shandong.CHN/11.07	T	C	A	T	A	T	T	A	A	C	A	A	T	T	G
1	MuVi/Sichuan.CHN/24.15	T	C	A	T	A	T	T	A	A	C	A	A	T	T	G
1	MuVi/Heilongjiang.CHN/24.14	T	C	A	T	A	T	T	A	A	C	A	A	T	T	G
1	MuVi/Shandong.CHN/43.07	T	C	A	T	A	T	T	A	A	C	A	A	T	T	G
1	MuVi/Shandong.CHN/10.08	T	C	A	T	A	T	T	A	A	C	A	A	T	T	G
1	MuVi/Heilongjiang.CHN/26.12/2	T	C	A	T	A	T	T	A	A	C	A	A	T	T	G
1	MuVi/Liaoning.CHN/48.11	T	C	A	T	A	T	T	A	A	C	A	A	T	T	G
1	MuVi/Heilongjiang.CHN/23.13/1	T	C	A	T	A	T	T	A	A	C	A	A	T	T	G
1	MuVi/Liaoning.CHN/50.11	T	C	A	T	A	T	T	A	A	C	A	A	T	T	G
1	MuVs/Kaohsiung.TWN/06.08	T	C	A	T	A	T	T	A	A	C	A	A	T	T	G
	MuVi/Jiangsu.CHN/12.13/2	T	C	A	T	A	T	T	A	G	C	G	A	T	T	G
	MuVi/Shannxi.CHN/20.15/1	T	C	A	T	A	T	T	A	G	C	G	A	T	T	G
	MuVi/Beijing.CHN/10.07	T	C	A	T	A	T	T	A	A	C	G	A	T	T	G
	MuVi/Hunan.CHN/30.11/3	T	C	A	T	A	T	T	A	A	C	G	A	T	T	G
	MuVi/Liaoning.CHN/9.10/1	T	C	A	T	A	T	T	A	A	C	G	A	T	T	G
	MuVi/Jiangsu.CHN/7.12	T	C	A	T	A	T	T	A	A	C	G	A	T	T	G
	MuVi/Heilongjiang.CHN/14.13/1	T	C	A	T	A	T	T	A	A	C	G	A	T	T	G
	MuVi/Hunan.CHN/32.11	T	C	A	T	A	T	T	A	A	C	G	A	T	T	G
	MuVi/Hunan.CHN/30.11/1	T	C	A	T	A	T	T	A	A	C	G	A	T	T	G
	MuVi/Jiangsu.CHN/7.13/1	T	C	A	T	A	T	T	A	A	T	G	A	T	T	G
	MuVi/Hunan.CHN/30.11/2	T	C	A	T	A	T	T	C	A	C	G	A	T	T	G
	MuVi/Liaoning.CHN/5.09/2	T	C	A	T	A	T	T	A	A	C	G	A	T	T	G
	Mui/Zhejiang.CHN/11.06/1	T	C	A	T	A	T	T	A	A	C	G	A	T	T	G
	MuVi/Liaoning.CHN/13.12	T	C	A	T	G	T	T	A	A	C	G	A	T	T	G
	MuVi/Beijing.CHN/17.07/2	T	C	A	T	A	T	T	A	A	C	G	A	T	T	G
	MuVi/Liaoning.CHN/7.12	T	C	A	T	A	T	T	A	A	C	G	A	T	T	G
	MuVi/Beijing.CHN/21.11	T	C	A	T	A	T	T	A	A	C	G	A	T	C	G
	MuVi/Liaoning.CHN/11.12	T	C	A	T	A	T	T	A	A	C	G	A	T	T	G
	MuVi/Heilongjiang.CHN/26.12/1	T	C	A	T	A	T	T	A	A	C	G	A	T	T	G
	MuVi/Liaoning.CHN/1.08/1	T	C	A	T	A	T	T	A	A	C	G	A	T	T	G
	MuVi/Liaoning.CHN/48.10/1	T	C	G	T	A	T	T	A	A	C	G	A	T	T	G
	MuVi/Jiangsu.CHN/4.13/5	T	C	A	T	A	T	T	A	A	C	G	A	T	T	G
	MuVi/Liaoning.CHN/10.09	T	C	A	T	A	T	C	A	A	C	G	A	T	T	G
	MuVi/Liaoning.CHN/1.08/2	T	C	A	T	A	T	C	A	A	C	G	A	T	T	G
	MuVi/Liaoning.CHN/5.09/1	T	C	A	T	A	T	A	A	A	C	G	A	T	T	G
	MuVi/Heilongjiang.CHN/22.12	T	T	G	T	A	T	T	A	A	C	G	A	T	T	G
	MuVi/Heilongjiang.CHN/15.13	T	T	A	T	A	T	T	A	A	C	G	A	T	T	G
	MuVi/Sichuan.CHN/23.12/2	T	C	A	T	A	T	T	A	A	C	G	A	T	T	G
	MuVi/Hubei.CHN/44.12/1	T	C	A	T	A	T	T	A	A	C	G	A	T	T	A
	MuVi/Jiangsu.CHN/2.13/4	T	C	A	T	A	T	T	A	A	C	G	A	T	T	G

1: Lineage based on Figure 1
2: SNPs are noted in green.
3: SNP position corresponding to a lineage-defining node ir

Supplementary Table S2: Listing of SNPs featured in the 7:

Lineage [1]	Strain name	263	279	285	286	288	291	297	300	303	312	315	321	336	348	351
2	MuVi/Shanxi.CHN/52.10/2	C	T	A	G	A	G	C	A	C	T	G	C	G	C	A
2	MuVi/Shannxi.CHN/26.09/4	T	T	A	G	A	G	C	A	T	T	G	C	G	C	A
2	MuVi/Shanxi.CHN/52.10/1	T	T	A	G	A	G	C	A	C	T	G	C	G	C	A
2	MuVi/Guangdong.CHN/21.09/1	T	T	A	G	A	G	C	A	C	T	G	C	G	C	A
2	MuVi/Liaoning.CHN/48.10/2	T	T	A	G	A	G	C	A	C	T	A	C	G	C	A
2	MuVi/Shandong.CHN/51.15/1	T	T	A	G	A	G	C	A	C	T	A	C	G	C	A
2	MuVi/Heilongjiang.CHN/49.15	T	T	A	G	A	G	C	A	C	T	A	C	G	C	A
2	MuVi/Anhui.CHN/10.11/1	T	T	A	G	A	G	C	A	C	T	A	C	G	C	A
2	MuVi/Shannxi.CHN/20.12	T	T	A	G	A	G	C	A	C	T	A	C	G	C	A
3	MuVi/Shanxi.CHN/52.10/3	T	T	A	G	G	G	C	A	C	T	G	C	A	C	A
3	MuVi/Jiangsu.CHN/3.13/1	T	T	A	G	G	G	C	A	C	T	G	C	A	C	A
3	MuVi/Shandong.CHN/4.05	T	T	A	G	G	G	C	A	C	T	G	C	A	C	A
3	MuVi/Shannxi.CHN/9.09/2	T	T	A	G	G	G	C	A	C	T	G	C	A	C	A
3	MuVi/Incheon.KOR/16.08/22	T	T	A	G	G	G	C	A	C	T	G	C	A	C	A
3	MuVi/Henan.CHN/48.06	T	T	A	G	G	G	C	A	C	T	G	C	A	C	A
3	MuVi/Jiangsu.CHN/9.12	T	T	A	G	G	G	C	A	C	T	G	C	A	C	A
3	MuVi/Jiangsu.CHN/4.13/3	T	T	A	G	G	G	C	A	C	T	G	C	A	C	A
4	MuVi/Shannxi.CHN/26.09/1	T	T	A	G	A	G	C	A	C	T	G	C	A	C	A
4	MuVi/Jiangsu.CHN/15.12	T	T	A	G	A	G	C	A	C	T	G	C	A	C	A
1	MuVi/Jilin.CHN/15.08/5	T	T	A	G	A	T	C	A	C	T	G	C	A	C	A
1	MuVi/Jilin.CHN/15.08/1	T	T	A	G	A	T	C	A	C	T	G	C	A	C	A
1	MuVi/Jilin.CHN/15.08/3	T	T	A	G	A	T	C	A	C	T	G	C	A	C	A
1	MuVi/Yunnan.CHN/47.10/2	T	T	A	G	A	T	C	A	C	T	G	C	A	C	A
1	MuVi/Neimeng.CHN/18.11	T	T	A	G	A	T	C	A	C	T	G	C	A	C	A
1	MuVi/Sichuan.CHN/23.12/1	T	T	A	G	A	T	C	A	C	T	G	C	A	C	A
1	MuVi/Jiangsu.CHN/3.13/2	T	T	A	G	A	T	C	A	C	T	G	C	A	C	A
1	MuVi/Beijing.CHN/25.06	T	T	A	G	A	T	C	A	C	T	G	C	A	C	A
1	MuVi/Zhejiang.CHN/26.05	T	T	A	G	A	T	C	A	C	T	G	C	A	C	A
1	MuVi/Shanghai.CHN/0.01	T	T	A	G	A	T	C	A	C	T	G	C	A	C	A
1	MuVi/Shandong.CHN/3.05	T	T	A	G	A	T	C	A	C	T	G	C	A	C	A
1	MuVi/Gansu.CHN/0.02	T	T	A	G	A	G	C	A	C	T	G	C	A	C	A
1	MuVi/Beijing.CHN/15.06	T	T	A	G	A	G	C	A	C	T	G	C	A	C	A
1	MuVi/Shandong.CHN/11.07	T	T	A	G	A	G	C	A	C	T	G	C	A	C	A
1	MuVi/Sichuan.CHN/24.15	T	T	A	G	A	G	C	A	C	T	G	C	A	C	A
1	MuVi/Heilongjiang.CHN/24.14	T	T	A	G	A	G	C	A	C	T	G	C	A	C	A
1	MuVi/Shandong.CHN/43.07	T	T	A	G	A	G	C	A	C	T	G	C	A	C	A
1	MuVi/Shandong.CHN/10.08	T	T	A	G	A	G	C	A	C	T	G	C	A	C	A
1	MuVi/Heilongjiang.CHN/26.12/2	T	T	A	G	A	G	C	A	C	T	G	C	A	C	A
1	MuVi/Liaoning.CHN/48.11	T	T	A	G	A	G	C	A	C	T	G	C	A	C	A
1	MuVi/Heilongjiang.CHN/23.13/1	T	T	A	G	A	G	C	A	C	T	G	C	A	C	A
1	MuVi/Liaoning.CHN/50.11	T	T	A	G	A	G	C	A	C	T	G	C	A	C	A
1	MuVs/Kaohsiung.TWN/06.08	T	T	A	G	A	A	C	A	C	T	G	C	A	C	A
	MuVi/Jiangsu.CHN/12.13/2	T	T	A	G	G	G	C	G	C	T	G	C	A	C	A
	MuVi/Shannxi.CHN/20.15/1	T	T	A	G	G	G	C	A	C	T	G	C	A	C	A
	MuVi/Beijing.CHN/10.07	T	T	A	G	A	A	C	A	C	T	G	C	A	C	C
	MuVi/Hunan.CHN/30.11/3	T	T	A	G	A	G	C	A	C	T	G	C	A	C	A
	MuVi/Liaoning.CHN/9.10/1	T	T	A	G	A	G	C	A	C	T	G	C	A	C	A
	MuVi/Jiangsu.CHN/7.12	T	T	A	G	A	G	C	A	C	T	G	C	A	C	A
	MuVi/Heilongjiang.CHN/14.13/1	T	T	A	G	A	G	C	A	C	T	G	C	A	C	A
	MuVi/Hunan.CHN/32.11	T	T	A	G	A	G	C	A	C	T	G	C	A	C	A
	MuVi/Hunan.CHN/30.11/1	T	T	A	G	A	G	C	A	C	T	G	C	A	C	A
	MuVi/Jiangsu.CHN/7.13/1	T	T	A	G	A	G	C	A	C	T	G	C	A	C	A
	MuVi/Hunan.CHN/30.11/2	T	C	A	G	G	G	C	A	C	T	G	C	A	C	A
	MuVi/Liaoning.CHN/5.09/2	T	T	A	A	A	G	C	A	C	T	G	T	A	T	A
	Mui/Zhejiang.CHN/11.06/1	T	T	A	G	A	G	C	A	C	T	G	C	A	C	A
	MuVi/Liaoning.CHN/13.12	T	T	A	G	A	G	C	A	C	T	G	C	A	C	A
	MuVi/Beijing.CHN/17.07/2	T	T	A	G	A	G	T	A	C	T	G	C	A	C	A
	MuVi/Liaoning.CHN/7.12	T	T	T	G	A	G	C	A	C	T	G	C	A	C	A
	MuVi/Beijing.CHN/21.11	T	T	A	G	A	G	C	A	C	T	G	T	A	C	A
	MuVi/Liaoning.CHN/11.12	T	T	A	G	A	G	C	A	C	T	G	T	A	C	A
	MuVi/Heilongjiang.CHN/26.12/1	T	T	A	G	A	G	C	A	C	T	G	T	A	C	A
	MuVi/Liaoning.CHN/1.08/1	T	T	A	G	A	G	C	A	C	T	G	C	A	C	A
	MuVi/Liaoning.CHN/48.10/1	T	T	A	G	A	G	C	A	C	T	G	C	A	C	A
	MuVi/Jiangsu.CHN/4.13/5	T	T	A	G	A	G	C	A	C	T	G	C	A	C	A
	MuVi/Liaoning.CHN/10.09	T	T	A	G	A	G	C	A	C	T	G	C	A	C	A
	MuVi/Liaoning.CHN/1.08/2	T	T	A	G	A	G	C	A	C	T	G	C	A	C	A
	MuVi/Liaoning.CHN/5.09/1	T	T	A	G	A	G	C	A	C	T	G	C	A	C	A
	MuVi/Heilongjiang.CHN/22.12	T	T	A	G	A	G	C	A	C	T	G	C	A	C	A
	MuVi/Heilongjiang.CHN/15.13	T	T	A	G	A	G	C	A	C	T	G	C	A	C	A
	MuVi/Sichuan.CHN/23.12/2	T	T	A	G	A	G	C	A	C	C	G	C	A	C	A
	MuVi/Hubei.CHN/44.12/1	T	T	A	G	A	G	C	A	C	T	G	C	A	C	A
	MuVi/Jiangsu.CHN/2.13/4	T	T	A	G	A	G	C	A	C	T	G	C	A	C	A

1: Lineage based on Figure 1
2: SNPs are noted in green.
3: SNP position corresponding to a lineage-defining node ir

Supplementary Table S2: Listing of SNPs featured in the 7

Lineage [1]	Strain name	354	358	363	368	372	378	381	387	389	390	392	393	396	402	403
2	MuVi/Shanxi.CHN/52.10/2	C	A	C	A	A	A	C	T	C	G	A	C	C	A	G
2	MuVi/Shannxi.CHN/26.09/4	C	A	C	A	A	A	C	T	C	A	A	C	C	A	G
2	MuVi/Shanxi.CHN/52.10/1	C	A	C	A	A	A	C	T	C	G	A	C	C	A	G
2	MuVi/Guangdong.CHN/21.09/1	C	A	C	A	A	A	C	T	C	G	A	C	C	A	G
2	MuVi/Liaoning.CHN/48.10/2	C	A	C	A	A	A	C	T	C	G	A	C	C	A	G
2	MuVi/Shandong.CHN/51.15/1	C	A	C	A	A	A	C	T	C	G	A	C	C	A	G
2	MuVi/Heilongjiang.CHN/49.15	C	A	C	A	A	A	C	T	C	G	A	C	C	A	G
2	MuVi/Anhui.CHN/10.11/1	C	A	C	A	A	A	C	T	C	G	A	C	C	A	G
2	MuVi/Shannxi.CHN/20.12	C	A	C	A	A	A	C	T	C	G	A	C	C	A	G
3	MuVi/Shanxi.CHN/52.10/3	C	A	C	G	A	A	C	T	T	G	A	C	C	A	G
3	MuVi/Jiangsu.CHN/3.13/1	C	A	C	A	A	A	C	T	T	G	A	C	C	A	G
3	MuVi/Shandong.CHN/4.05	C	A	C	A	A	A	C	T	C	G	A	T	C	A	G
3	MuVi/Shannxi.CHN/9.09/2	C	A	C	A	A	A	C	T	C	G	A	T	C	A	G
3	MuVi/Incheon.KOR/16.08/22	C	A	C	A	A	A	C	T	C	G	A	T	C	A	G
3	MuVi/Henan.CHN/48.06	C	A	C	A	A	A	C	T	C	G	A	T	C	A	G
3	MuVi/Jiangsu.CHN/9.12	C	A	C	A	A	A	C	T	C	G	A	T	C	G	G
3	MuVi/Jiangsu.CHN/4.13/3	C	A	C	A	A	A	C	T	C	G	A	T	C	G	G
4	MuVi/Shannxi.CHN/26.09/1	C	A	C	A	A	A	C	T	C	G	A	C	C	A	G
4	MuVi/Jiangsu.CHN/15.12	C	A	T	A	A	A	C	T	C	G	A	C	C	A	G
1	MuVi/Jilin.CHN/15.08/5	C	A	C	A	A	A	C	T	C	G	A	C	C	A	G
1	MuVi/Jilin.CHN/15.08/1	C	A	C	A	A	A	C	T	C	G	A	C	C	A	G
1	MuVi/Jilin.CHN/15.08/3	C	A	C	A	A	A	C	T	C	A	A	C	C	A	G
1	MuVi/Yunnan.CHN/47.10/2	C	A	C	A	A	A	C	T	C	G	A	C	C	A	G
1	MuVi/Neimeng.CHN/18.11	C	A	C	A	A	A	C	T	C	G	A	C	C	A	G
1	MuVi/Sichuan.CHN/23.12/1	C	A	C	A	A	A	C	T	C	A	A	C	C	A	G
1	MuVi/Jiangsu.CHN/3.13/2	C	A	C	A	A	A	C	T	C	G	A	C	C	A	G
1	MuVi/Beijing.CHN/25.06	C	A	C	A	A	A	C	T	C	G	A	C	C	A	G
1	MuVi/Zhejiang.CHN/26.05	C	A	C	A	A	C	C	T	C	G	A	C	C	A	G
1	MuVi/Shanghai.CHN/0.01	C	A	C	A	A	A	C	T	C	G	A	C	C	A	G
1	MuVi/Shandong.CHN/3.05	C	A	C	A	A	A	C	T	C	G	A	C	C	A	G
1	MuVi/Gansu.CHN/0.02	C	A	C	A	A	A	C	T	C	G	A	C	C	A	G
1	MuVi/Beijing.CHN/15.06	C	G	C	A	A	A	C	T	C	A	A	C	C	A	G
1	MuVi/Shandong.CHN/11.07	C	G	C	A	A	A	C	T	C	G	A	C	C	A	G
1	MuVi/Sichuan.CHN/24.15	C	G	C	A	A	A	T	T	C	G	A	C	C	A	G
1	MuVi/Heilongjiang.CHN/24.14	C	G	C	A	A	A	C	T	C	G	A	C	C	A	G
1	MuVi/Shandong.CHN/43.07	C	G	C	A	A	A	C	T	C	G	A	C	C	A	G
1	MuVi/Shandong.CHN/10.08	C	G	C	A	G	A	C	T	C	G	A	C	C	A	G
1	MuVi/Heilongjiang.CHN/26.12/2	C	G	C	A	A	A	C	T	C	G	A	C	C	A	G
1	MuVi/Liaoning.CHN/48.11	C	G	C	A	A	A	C	T	C	G	A	C	C	A	G
1	MuVi/Heilongjiang.CHN/23.13/1	C	G	C	A	A	A	C	T	C	G	A	C	C	A	G
1	MuVi/Liaoning.CHN/50.11	C	G	C	A	A	A	C	T	C	G	A	C	C	A	G
1	MuVs/Kaohsiung.TWN/06.08	C	G	C	A	A	A	C	T	C	G	A	C	C	A	G
	MuVi/Jiangsu.CHN/12.13/2	C	A	C	A	A	A	C	T	C	G	A	C	C	A	G
	MuVi/Shannxi.CHN/20.15/1	C	A	C	A	A	A	C	T	C	G	A	C	C	A	G
	MuVi/Beijing.CHN/10.07	C	A	C	A	A	A	C	T	C	G	A	C	T	A	G
	MuVi/Hunan.CHN/30.11/3	C	A	C	A	A	A	C	T	C	G	A	C	C	A	A
	MuVi/Liaoning.CHN/9.10/1	C	A	C	A	A	A	C	T	C	G	A	C	C	A	A
	MuVi/Jiangsu.CHN/7.12	C	A	C	A	A	A	C	T	C	G	A	C	C	A	A
	MuVi/Heilongjiang.CHN/14.13/1	C	A	C	A	A	A	C	T	C	G	A	C	C	A	A
	MuVi/Hunan.CHN/32.11	C	A	C	A	A	A	C	T	C	G	A	C	C	A	G
	MuVi/Hunan.CHN/30.11/1	C	A	C	A	A	A	C	T	C	G	A	C	C	A	G
	MuVi/Jiangsu.CHN/7.13/1	C	A	C	A	A	A	C	T	A	G	A	C	C	A	G
	MuVi/Hunan.CHN/30.11/2	C	A	C	A	A	A	C	T	C	G	A	C	C	A	G
	MuVi/Liaoning.CHN/5.09/2	C	A	C	A	A	A	C	T	C	G	A	C	C	A	G
	Mui/Zhejiang.CHN/11.06/1	C	A	C	A	A	A	C	T	C	G	A	C	C	A	G
	MuVi/Liaoning.CHN/13.12	T	A	C	A	A	A	C	T	C	G	G	C	C	A	G
	MuVi/Beijing.CHN/17.07/2	C	A	C	A	A	A	C	T	C	G	A	C	C	A	G
	MuVi/Liaoning.CHN/7.12	C	A	C	A	A	A	C	T	C	G	A	C	C	A	G
	MuVi/Beijing.CHN/21.11	C	A	C	A	A	A	C	T	C	G	A	T	C	A	G
	MuVi/Liaoning.CHN/11.12	C	A	C	A	A	A	C	C	C	G	A	T	C	A	G
	MuVi/Heilongjiang.CHN/26.12/1	C	A	C	A	A	A	C	T	C	G	A	T	C	A	G
	MuVi/Liaoning.CHN/1.08/1	C	A	C	A	A	A	C	T	C	G	A	T	C	A	G
	MuVi/Liaoning.CHN/48.10/1	C	A	C	A	A	A	C	T	C	G	A	T	C	A	G
	MuVi/Jiangsu.CHN/4.13/5	C	A	C	A	A	A	C	T	C	G	A	T	C	A	G
	MuVi/Liaoning.CHN/10.09	C	A	C	A	A	A	C	T	C	G	A	T	C	A	G
	MuVi/Liaoning.CHN/1.08/2	C	A	C	A	A	A	C	T	C	G	A	T	C	A	G
	MuVi/Liaoning.CHN/5.09/1	C	A	C	A	A	A	C	T	C	G	A	T	C	A	G
	MuVi/Heilongjiang.CHN/22.12	C	A	C	A	A	A	C	T	C	G	A	C	C	A	G
	MuVi/Heilongjiang.CHN/15.13	C	A	C	A	A	A	C	T	C	G	A	C	C	A	G
	MuVi/Sichuan.CHN/23.12/2	C	A	C	A	A	A	C	T	C	G	A	C	C	A	G
	MuVi/Hubei.CHN/44.12/1	C	A	C	A	A	A	C	T	C	G	A	C	C	A	G
	MuVi/Jiangsu.CHN/2.13/4	C	A	C	A	A	A	C	T	C	G	A	C	C	A	G

1: Lineage based on Figure 1
2: SNPs are noted in green.
3: SNP position corresponding to a lineage-defining node ir

Supplementary Table S2: Listing of SNPs featured in the 7

Lineage [1]	Strain name	414	421	429	438	444	447	457	466	471	472	474	483	489	492	513
2	MuVi/Shanxi.CHN/52.10/2	T	A	G	G	C	T	G	G	C	T	T	T	C	C	T
2	MuVi/Shannxi.CHN/26.09/4	T	A	G	G	C	T	G	G	C	T	T	A	C	C	T
2	MuVi/Shanxi.CHN/52.10/1	T	A	G	G	C	T	G	G	C	T	T	T	C	C	T
2	MuVi/Guangdong.CHN/21.09/1	T	A	G	G	C	T	G	G	C	T	T	T	C	C	T
2	MuVi/Liaoning.CHN/48.10/2	T	A	G	G	C	T	G	G	C	T	T	T	C	C	T
2	MuVi/Shandong.CHN/51.15/1	T	A	G	G	C	T	G	A	C	T	T	T	C	C	T
2	MuVi/Heilongjiang.CHN/49.15	T	A	G	G	C	T	G	G	C	T	T	T	C	C	T
2	MuVi/Anhui.CHN/10.11/1	T	A	G	G	C	T	G	G	C	T	T	T	C	C	T
2	MuVi/Shannxi.CHN/20.12	C	A	G	G	C	T	G	G	C	T	T	T	C	C	T
3	MuVi/Shanxi.CHN/52.10/3	T	A	G	A	A	T	G	G	C	T	T	T	C	C	T
3	MuVi/Jiangsu.CHN/3.13/1	T	A	G	A	C	T	G	G	T	T	T	T	C	C	T
3	MuVi/Shandong.CHN/4.05	T	A	G	A	C	T	G	G	C	T	C	T	C	C	T
3	MuVi/Shannxi.CHN/9.09/2	T	A	G	A	C	T	G	G	C	G	C	T	C	C	T
3	MuVi/Incheon.KOR/16.08/22	T	A	G	A	C	T	T	G	C	T	C	T	C	C	T
3	MuVi/Henan.CHN/48.06	T	A	G	A	C	T	G	G	C	T	T	T	C	C	T
3	MuVi/Jiangsu.CHN/9.12	T	A	G	A	C	T	G	G	C	T	T	T	C	C	T
3	MuVi/Jiangsu.CHN/4.13/3	T	A	G	A	C	T	G	G	C	T	T	T	C	C	T
4	MuVi/Shannxi.CHN/26.09/1	T	A	G	G	C	T	G	G	C	T	T	T	C	C	T
4	MuVi/Jiangsu.CHN/15.12	T	A	G	A	C	T	G	G	C	T	T	T	C	C	T
1	MuVi/Jilin.CHN/15.08/5	T	A	G	A	C	T	G	G	C	T	C	T	C	C	C
1	MuVi/Jilin.CHN/15.08/1	T	A	G	A	C	T	G	G	C	T	C	T	C	C	C
1	MuVi/Jilin.CHN/15.08/3	T	A	G	A	C	T	G	G	C	T	C	T	C	C	C
1	MuVi/Yunnan.CHN/47.10/2	T	A	G	A	C	T	G	G	C	T	T	T	C	C	C
1	MuVi/Neimeng.CHN/18.11	T	A	G	A	C	T	G	G	C	T	T	T	C	C	C
1	MuVi/Sichuan.CHN/23.12/1	C	A	G	A	C	T	G	G	C	T	T	T	C	C	C
1	MuVi/Jiangsu.CHN/3.13/2	T	A	G	A	C	T	G	G	C	T	T	T	C	C	T
1	MuVi/Beijing.CHN/25.06	T	A	G	A	C	T	G	G	C	T	T	T	C	C	T
1	MuVi/Zhejiang.CHN/26.05	T	A	G	A	C	T	G	G	C	T	T	C	C	C	T
1	MuVi/Shanghai.CHN/0.01	T	A	G	A	C	T	G	G	C	T	T	T	T	C	T
1	MuVi/Shandong.CHN/3.05	T	A	G	A	C	T	G	G	C	T	T	T	C	C	T
1	MuVi/Gansu.CHN/0.02	T	A	G	A	C	T	G	G	C	T	T	T	C	C	T
1	MuVi/Beijing.CHN/15.06	T	A	C	A	C	T	G	G	C	T	T	C	C	T	T
1	MuVi/Shandong.CHN/11.07	T	A	G	A	C	C	G	G	C	T	T	C	C	T	T
1	MuVi/Sichuan.CHN/24.15	T	A	G	A	C	T	G	G	C	T	T	C	C	T	T
1	MuVi/Heilongjiang.CHN/24.14	T	A	T	A	C	T	G	G	C	T	T	C	C	T	T
1	MuVi/Shandong.CHN/43.07	T	A	G	A	C	T	G	G	C	T	T	C	C	T	T
1	MuVi/Shandong.CHN/10.08	T	A	G	A	C	T	G	G	C	T	T	C	C	T	T
1	MuVi/Heilongjiang.CHN/26.12/2	T	A	G	A	C	T	G	G	C	T	T	C	C	T	T
1	MuVi/Liaoning.CHN/48.11	T	A	G	A	C	T	G	G	C	T	T	C	C	T	T
1	MuVi/Heilongjiang.CHN/23.13/1	T	A	G	A	C	T	G	G	C	T	T	C	C	T	T
1	MuVi/Liaoning.CHN/50.11	T	A	G	A	C	T	G	G	C	T	T	C	C	T	T
1	MuVs/Kaohsiung.TWN/06.08	T	A	G	A	C	T	G	G	C	T	T	C	C	T	T
	MuVi/Jiangsu.CHN/12.13/2	T	A	G	A	C	T	G	G	C	T	T	T	C	C	T
	MuVi/Shannxi.CHN/20.15/1	T	A	G	A	C	T	G	G	C	T	T	T	C	C	T
	MuVi/Beijing.CHN/10.07	T	A	G	A	C	T	G	G	C	T	T	T	C	C	T
	MuVi/Hunan.CHN/30.11/3	T	A	G	A	C	T	G	G	C	T	T	T	C	C	T
	MuVi/Liaoning.CHN/9.10/1	T	A	G	A	C	T	G	G	C	T	T	T	C	C	T
	MuVi/Jiangsu.CHN/7.12	T	A	G	A	C	T	G	G	C	T	T	T	C	C	T
	MuVi/Heilongjiang.CHN/14.13/1	T	A	G	A	C	T	G	G	C	T	T	T	C	C	T
	MuVi/Hunan.CHN/32.11	T	A	G	A	C	T	G	G	C	T	T	T	C	C	T
	MuVi/Hunan.CHN/30.11/1	T	C	G	A	C	T	G	G	C	T	T	T	C	C	T
	MuVi/Jiangsu.CHN/7.13/1	T	C	G	A	C	T	G	G	C	T	T	T	C	C	T
	MuVi/Hunan.CHN/30.11/2	T	A	G	A	C	T	G	G	C	T	T	T	C	C	T
	MuVi/Liaoning.CHN/5.09/2	T	A	G	A	C	T	G	G	C	T	T	T	C	C	T
	Mui/Zhejiang.CHN/11.06/1	T	A	G	A	C	T	G	G	C	T	T	T	C	C	T
	MuVi/Liaoning.CHN/13.12	T	A	G	A	C	T	G	G	C	T	T	T	C	C	T
	MuVi/Beijing.CHN/17.07/2	T	A	G	A	C	T	G	G	C	T	T	T	C	C	T
	MuVi/Liaoning.CHN/7.12	T	A	G	A	C	T	G	G	C	T	T	T	C	C	T
	MuVi/Beijing.CHN/21.11	T	A	G	A	C	T	G	G	C	T	T	T	T	C	T
	MuVi/Liaoning.CHN/11.12	T	A	G	A	C	T	G	G	C	T	T	T	T	C	T
	MuVi/Heilongjiang.CHN/26.12/1	T	A	G	A	C	T	G	G	C	T	T	T	T	C	T
	MuVi/Liaoning.CHN/1.08/1	T	A	G	A	C	T	G	G	C	T	T	T	T	C	T
	MuVi/Liaoning.CHN/48.10/1	T	A	G	A	C	T	G	G	C	T	T	T	T	C	T
	MuVi/Jiangsu.CHN/4.13/5	T	A	G	A	C	T	G	G	C	T	T	T	C	C	T
	MuVi/Liaoning.CHN/10.09	T	A	G	A	C	T	G	G	C	T	T	T	C	C	T
	MuVi/Liaoning.CHN/1.08/2	T	A	G	A	C	T	G	G	C	T	T	T	C	C	T
	MuVi/Liaoning.CHN/5.09/1	T	A	G	A	C	T	G	G	C	T	T	T	C	C	T
	MuVi/Heilongjiang.CHN/22.12	T	A	G	A	C	T	G	G	C	T	T	T	C	C	T
	MuVi/Heilongjiang.CHN/15.13	T	A	G	A	C	T	G	G	C	T	T	T	C	C	T
	MuVi/Sichuan.CHN/23.12/2	T	A	G	A	C	T	G	G	C	T	T	T	C	C	T
	MuVi/Hubei.CHN/44.12/1	T	A	G	A	C	T	G	G	C	T	T	T	C	C	T
	MuVi/Jiangsu.CHN/2.13/4	T	A	G	A	C	T	G	G	C	T	T	T	C	C	T

1: Lineage based on Figure 1
2: SNPs are noted in green.
3: SNP position corresponding to a lineage-defining node ir

Supplementary Table S2: Listing of SNPs featured in the 7[illegible]

Lineage [1]	Strain name	543	556	561	564	570	576	579	582	585	588	594	595	603	606	612
2	MuVi/Shanxi.CHN/52.10/2	T	C	T	G	C	C	C	A	T	T	T	A	C	C	T
2	MuVi/Shannxi.CHN/26.09/4	T	C	T	G	C	C	C	A	T	T	T	A	C	C	T
2	MuVi/Shanxi.CHN/52.10/1	T	C	T	G	C	C	C	A	T	T	T	A	C	C	T
2	MuVi/Guangdong.CHN/21.09/1	T	C	T	G	C	C	C	A	T	T	T	A	C	C	T
2	MuVi/Liaoning.CHN/48.10/2	T	T	T	G	C	C	C	A	T	T	T	A	C	C	T
2	MuVi/Shandong.CHN/51.15/1	T	T	T	G	C	C	C	A	T	T	T	A	C	C	T
2	MuVi/Heilongjiang.CHN/49.15	T	T	T	G	C	C	C	A	T	T	T	A	C	C	T
2	MuVi/Anhui.CHN/10.11/1	T	T	T	G	C	C	C	A	T	T	T	A	C	C	T
2	MuVi/Shannxi.CHN/20.12	T	T	T	G	C	C	C	A	T	T	T	A	C	C	T
3	MuVi/Shanxi.CHN/52.10/3	T	C	T	G	C	C	C	A	T	T	T	A	C	C	T
3	MuVi/Jiangsu.CHN/3.13/1	T	C	T	G	C	C	C	A	T	T	T	A	C	C	T
3	MuVi/Shandong.CHN/4.05	T	C	T	G	C	C	C	A	T	T	T	A	C	C	T
3	MuVi/Shannxi.CHN/9.09/2	T	C	T	G	C	C	C	A	T	T	T	A	C	C	T
3	MuVi/Incheon.KOR/16.08/22	T	C	T	G	C	C	C	A	T	T	T	A	C	C	T
3	MuVi/Henan.CHN/48.06	T	C	T	G	C	C	C	A	T	T	T	A	C	C	T
3	MuVi/Jiangsu.CHN/9.12	T	C	T	G	C	C	C	A	T	T	T	A	C	C	T
3	MuVi/Jiangsu.CHN/4.13/3	T	C	T	G	C	C	C	A	T	T	T	A	C	C	T
4	MuVi/Shannxi.CHN/26.09/1	T	C	T	G	C	C	C	A	T	T	T	A	C	C	T
4	MuVi/Jiangsu.CHN/15.12	T	C	T	G	C	C	C	A	T	T	T	A	C	C	T
1	MuVi/Jilin.CHN/15.08/5	T	C	T	G	C	C	T	C	C	C	C	A	C	C	T
1	MuVi/Jilin.CHN/15.08/1	T	C	T	G	C	C	T	C	C	C	C	A	C	C	T
1	MuVi/Jilin.CHN/15.08/3	T	C	T	G	C	C	T	C	C	C	C	A	C	C	T
1	MuVi/Yunnan.CHN/47.10/2	T	C	T	G	C	C	T	C	T	T	T	A	C	C	T
1	MuVi/Neimeng.CHN/18.11	T	C	C	A	C	C	T	C	T	T	T	A	C	C	T
1	MuVi/Sichuan.CHN/23.12/1	T	C	T	G	C	C	T	T	T	T	T	A	C	C	T
1	MuVi/Jiangsu.CHN/3.13/2	T	C	T	G	C	C	T	A	T	T	T	A	C	C	T
1	MuVi/Beijing.CHN/25.06	T	C	T	G	C	C	T	A	T	T	T	A	C	C	T
1	MuVi/Zhejiang.CHN/26.05	T	C	T	G	C	C	T	A	T	T	T	A	C	C	T
1	MuVi/Shanghai.CHN/0.01	T	C	T	G	C	C	T	A	T	T	T	A	C	C	T
1	MuVi/Shandong.CHN/3.05	T	C	T	G	C	C	T	A	T	T	T	A	C	C	T
1	MuVi/Gansu.CHN/0.02	T	C	T	G	C	C	C	A	T	T	T	A	C	C	T
1	MuVi/Beijing.CHN/15.06	T	C	T	G	C	C	C	A	T	T	T	A	C	C	C
1	MuVi/Shandong.CHN/11.07	T	C	T	G	T	C	C	A	T	T	T	A	C	C	T
1	MuVi/Sichuan.CHN/24.15	T	C	T	G	C	C	C	A	T	T	T	A	T	C	T
1	MuVi/Heilongjiang.CHN/24.14	T	C	T	G	C	C	C	A	T	T	T	A	T	C	T
1	MuVi/Shandong.CHN/43.07	T	C	C	G	C	C	C	A	T	T	T	A	T	C	T
1	MuVi/Shandong.CHN/10.08	T	C	C	G	C	C	C	A	T	T	T	T	T	C	T
1	MuVi/Heilongjiang.CHN/26.12/2	T	C	T	G	C	C	C	A	T	T	T	A	T	C	T
1	MuVi/Liaoning.CHN/48.11	T	C	T	G	C	C	C	A	T	C	T	A	T	C	T
1	MuVi/Heilongjiang.CHN/23.13/1	T	C	T	G	C	C	C	A	T	C	T	A	T	C	T
1	MuVi/Liaoning.CHN/50.11	T	C	T	G	C	C	C	A	T	C	T	A	T	C	T
1	MuVs/Kaohsiung.TWN/06.08	T	C	T	G	C	C	C	A	T	T	T	A	C	C	T
	MuVi/Jiangsu.CHN/12.13/2	T	C	T	G	C	C	C	A	T	T	T	A	C	C	T
	MuVi/Shannxi.CHN/20.15/1	T	C	T	G	C	C	C	A	T	T	T	A	C	C	T
	MuVi/Beijing.CHN/10.07	T	C	T	G	C	C	C	A	T	T	T	A	C	C	T
	MuVi/Hunan.CHN/30.11/3	T	C	T	G	C	C	C	A	T	T	T	A	C	C	T
	MuVi/Liaoning.CHN/9.10/1	T	C	T	G	C	C	C	A	T	T	T	A	C	C	T
	MuVi/Jiangsu.CHN/7.12	T	C	T	G	C	C	C	A	T	T	T	A	C	C	T
	MuVi/Heilongjiang.CHN/14.13/1	T	C	T	G	C	C	C	A	T	T	T	A	C	C	T
	MuVi/Hunan.CHN/32.11	C	C	T	G	C	T	C	A	T	T	T	A	C	C	T
	MuVi/Hunan.CHN/30.11/1	T	C	T	G	C	C	C	A	T	T	T	A	C	C	T
	MuVi/Jiangsu.CHN/7.13/1	T	C	T	G	C	C	C	A	T	T	T	A	C	C	T
	MuVi/Hunan.CHN/30.11/2	T	C	G	G	C	C	C	A	T	T	T	A	C	C	T
	MuVi/Liaoning.CHN/5.09/2	T	C	T	G	C	C	C	A	T	T	T	A	C	C	T
	Mui/Zhejiang.CHN/11.06/1	T	C	T	G	C	C	C	A	T	T	T	A	C	C	T
	MuVi/Liaoning.CHN/13.12	T	C	T	G	C	C	C	A	T	T	T	A	C	T	T
	MuVi/Beijing.CHN/17.07/2	T	C	T	G	C	C	C	A	T	T	T	A	C	T	T
	MuVi/Liaoning.CHN/7.12	T	C	T	G	C	C	C	A	T	T	T	A	C	T	T
	MuVi/Beijing.CHN/21.11	T	C	T	G	C	C	C	A	T	T	T	A	C	T	T
	MuVi/Liaoning.CHN/11.12	T	C	T	G	C	C	C	A	T	T	T	A	C	T	T
	MuVi/Heilongjiang.CHN/26.12/1	T	C	T	G	C	C	C	A	T	T	T	A	C	T	T
	MuVi/Liaoning.CHN/1.08/1	T	C	T	G	C	C	C	A	T	T	T	A	C	T	T
	MuVi/Liaoning.CHN/48.10/1	T	C	T	G	C	C	C	A	T	T	T	A	C	T	T
	MuVi/Jiangsu.CHN/4.13/5	T	C	T	G	C	C	C	A	T	T	T	A	C	T	T
	MuVi/Liaoning.CHN/10.09	T	C	T	G	C	C	C	A	T	T	T	A	C	T	T
	MuVi/Liaoning.CHN/1.08/2	T	C	T	G	C	C	C	A	T	T	T	A	C	T	T
	MuVi/Liaoning.CHN/5.09/1	T	C	T	G	C	C	C	A	T	T	T	A	C	T	T
	MuVi/Heilongjiang.CHN/22.12	T	C	T	G	C	C	C	A	T	T	T	A	C	T	T
	MuVi/Heilongjiang.CHN/15.13	T	C	T	G	C	C	C	A	T	T	T	A	C	T	T
	MuVi/Sichuan.CHN/23.12/2	T	C	T	G	C	C	C	A	T	T	T	A	C	T	T
	MuVi/Hubei.CHN/44.12/1	T	C	T	G	C	C	C	A	T	T	T	A	C	T	T
	MuVi/Jiangsu.CHN/2.13/4	T	C	T	G	C	C	C	A	T	T	T	A	C	T	T

1: Lineage based on Figure 1
2: SNPs are noted in green.
3: SNP position corresponding to a lineage-defining node i[illegible]

Supplementary Table S2: Listing of SNPs featured in the 7:

Lineage [1]	Strain name	615	621	624	633	639	645	651	652	653	654	663	672	681	684	687
2	MuVi/Shanxi.CHN/52.10/2	T	G	T	T	C	T	C	G	T	T	G	T	C	A	C
2	MuVi/Shannxi.CHN/26.09/4	T	G	T	T	C	G	C	G	T	T	G	T	C	A	C
2	MuVi/Shanxi.CHN/52.10/1	T	G	T	T	C	G	C	G	T	T	G	T	C	A	C
2	MuVi/Guangdong.CHN/21.09/1	T	G	T	T	C	G	C	G	T	T	G	T	C	A	C
2	MuVi/Liaoning.CHN/48.10/2	T	G	T	T	C	G	C	G	T	T	G	T	C	A	C
2	MuVi/Shandong.CHN/51.15/1	T	G	T	T	C	G	C	G	T	T	G	T	C	A	C
2	MuVi/Heilongjiang.CHN/49.15	T	G	T	T	C	G	C	G	T	T	G	T	C	A	C
2	MuVi/Anhui.CHN/10.11/1	T	G	T	T	C	G	C	G	T	T	G	T	C	A	C
2	MuVi/Shannxi.CHN/20.12	T	G	T	T	C	G	C	G	T	T	G	T	C	A	C
3	MuVi/Shanxi.CHN/52.10/3	T	T	T	T	C	G	C	G	T	T	G	T	C	A	C
3	MuVi/Jiangsu.CHN/3.13/1	T	G	T	T	C	G	C	A	T	T	G	T	A	A	C
3	MuVi/Shandong.CHN/4.05	T	G	T	T	C	G	C	G	T	T	G	T	C	A	C
3	MuVi/Shannxi.CHN/9.09/2	T	G	T	T	C	G	C	G	T	T	G	T	C	A	C
3	MuVi/Incheon.KOR/16.08/22	T	G	T	T	C	G	C	G	T	T	G	T	C	A	C
3	MuVi/Henan.CHN/48.06	T	G	T	T	C	G	C	G	T	C	G	T	C	A	C
3	MuVi/Jiangsu.CHN/9.12	T	G	T	T	C	G	C	G	T	C	G	T	C	A	C
3	MuVi/Jiangsu.CHN/4.13/3	T	G	T	T	C	G	C	G	T	C	G	T	C	A	C
4	MuVi/Shannxi.CHN/26.09/1	T	G	T	T	C	G	C	G	T	T	G	T	C	A	C
4	MuVi/Jiangsu.CHN/15.12	T	G	T	T	C	G	C	G	T	T	G	T	C	A	C
1	MuVi/Jilin.CHN/15.08/5	C	G	C	T	C	G	T	G	T	T	G	T	C	A	A
1	MuVi/Jilin.CHN/15.08/1	C	G	C	T	C	G	T	G	T	T	G	T	C	A	A
1	MuVi/Jilin.CHN/15.08/3	C	G	C	T	C	G	T	G	T	T	G	T	C	A	A
1	MuVi/Yunnan.CHN/47.10/2	T	G	T	T	C	G	C	G	T	T	G	T	C	A	A
1	MuVi/Neimeng.CHN/18.11	T	G	T	T	C	G	C	G	T	T	G	T	C	A	A
1	MuVi/Sichuan.CHN/23.12/1	T	G	T	T	C	G	C	G	T	T	G	T	C	A	A
1	MuVi/Jiangsu.CHN/3.13/2	T	G	T	T	C	G	C	G	T	T	G	T	C	A	A
1	MuVi/Beijing.CHN/25.06	T	G	T	T	C	G	C	G	T	T	G	T	C	A	A
1	MuVi/Zhejiang.CHN/26.05	T	G	T	T	C	G	C	G	T	T	G	T	C	A	A
1	MuVi/Shanghai.CHN/0.01	T	G	T	T	C	G	C	G	T	T	G	T	C	A	A
1	MuVi/Shandong.CHN/3.05	T	G	T	T	C	G	C	G	T	T	G	C	C	A	A
1	MuVi/Gansu.CHN/0.02	T	G	T	T	C	G	C	G	T	T	G	T	C	A	A
1	MuVi/Beijing.CHN/15.06	T	G	T	T	C	G	C	G	T	T	G	T	C	A	A
1	MuVi/Shandong.CHN/11.07	T	G	T	T	C	G	C	G	T	T	G	T	C	A	A
1	MuVi/Sichuan.CHN/24.15	T	G	T	T	C	G	C	G	T	T	G	T	C	A	A
1	MuVi/Heilongjiang.CHN/24.14	T	G	T	T	C	G	C	G	T	T	G	T	C	A	A
1	MuVi/Shandong.CHN/43.07	T	G	T	T	C	G	C	G	T	T	G	T	C	A	A
1	MuVi/Shandong.CHN/10.08	T	G	T	T	C	G	C	G	T	T	G	T	C	A	A
1	MuVi/Heilongjiang.CHN/26.12/2	T	G	T	T	C	G	C	G	T	T	G	T	C	A	A
1	MuVi/Liaoning.CHN/48.11	T	G	T	T	C	G	C	G	T	T	G	T	C	A	A
1	MuVi/Heilongjiang.CHN/23.13/1	T	G	T	T	C	G	C	G	T	T	G	T	C	A	A
1	MuVi/Liaoning.CHN/50.11	T	G	T	T	C	G	C	G	T	T	G	T	C	A	A
1	MuVs/Kaohsiung.TWN/06.08	T	G	T	T	C	G	C	G	T	T	G	T	C	A	A
	MuVi/Jiangsu.CHN/12.13/2	T	G	T	T	T	G	T	G	C	T	G	T	C	A	C
	MuVi/Shannxi.CHN/20.15/1	T	G	T	T	T	G	T	G	C	T	G	T	C	A	C
	MuVi/Beijing.CHN/10.07	T	G	T	T	C	G	C	G	T	T	A	T	C	A	C
	MuVi/Hunan.CHN/30.11/3	T	G	T	T	T	G	C	G	C	T	G	T	C	A	C
	MuVi/Liaoning.CHN/9.10/1	T	G	T	T	T	G	C	G	C	T	G	T	C	A	C
	MuVi/Jiangsu.CHN/7.12	T	G	T	T	T	G	C	G	C	T	G	T	C	A	T
	MuVi/Heilongjiang.CHN/14.13/1	T	G	T	T	T	G	C	G	C	T	G	T	C	A	T
	MuVi/Hunan.CHN/32.11	T	G	T	C	T	G	C	G	C	T	G	T	C	G	C
	MuVi/Hunan.CHN/30.11/1	T	G	T	T	T	G	C	G	T	T	G	T	C	A	C
	MuVi/Jiangsu.CHN/7.13/1	T	G	T	T	C	G	C	G	T	T	G	T	C	A	C
	MuVi/Hunan.CHN/30.11/2	T	G	T	T	T	G	C	G	T	T	G	T	C	A	C
	MuVi/Liaoning.CHN/5.09/2	T	G	C	T	C	G	C	G	T	T	G	T	C	A	C
	Mui/Zhejiang.CHN/11.06/1	T	G	T	T	C	G	C	G	T	T	G	T	C	A	C
	MuVi/Liaoning.CHN/13.12	T	G	T	T	T	G	C	G	C	T	G	T	C	A	C
	MuVi/Beijing.CHN/17.07/2	T	G	T	T	T	G	C	G	C	T	G	T	C	A	C
	MuVi/Liaoning.CHN/7.12	T	G	T	T	T	G	C	G	T	T	G	T	C	A	C
	MuVi/Beijing.CHN/21.11	T	G	T	T	T	G	A	G	C	T	G	T	C	A	C
	MuVi/Liaoning.CHN/11.12	T	G	T	T	T	G	A	G	C	T	G	T	C	A	C
	MuVi/Heilongjiang.CHN/26.12/1	T	G	T	T	T	G	C	G	C	T	G	T	C	A	C
	MuVi/Liaoning.CHN/1.08/1	T	G	T	T	T	G	C	G	C	T	G	T	C	A	C
	MuVi/Liaoning.CHN/48.10/1	T	G	T	T	T	G	C	G	C	T	G	T	C	A	C
	MuVi/Jiangsu.CHN/4.13/5	T	G	T	T	T	G	C	G	C	T	G	T	C	A	C
	MuVi/Liaoning.CHN/10.09	T	G	T	T	T	G	C	G	C	T	G	T	C	A	C
	MuVi/Liaoning.CHN/1.08/2	T	G	T	T	T	G	C	G	C	T	G	T	C	A	C
	MuVi/Liaoning.CHN/5.09/1	T	G	T	T	T	G	C	G	C	T	G	T	C	A	C
	MuVi/Heilongjiang.CHN/22.12	T	G	T	T	T	G	A	G	T	T	G	T	C	A	C
	MuVi/Heilongjiang.CHN/15.13	T	G	T	T	T	G	A	G	T	T	G	T	C	A	C
	MuVi/Sichuan.CHN/23.12/2	T	G	T	T	T	G	C	G	T	T	G	T	C	A	C
	MuVi/Hubei.CHN/44.12/1	T	G	T	T	T	G	C	G	T	T	G	T	C	A	C
	MuVi/Jiangsu.CHN/2.13/4	T	G	T	T	T	G	C	G	T	T	G	T	C	A	C

1: Lineage based on Figure 1
2: SNPs are noted in green.
3: SNP position corresponding to a lineage-defining node ir

Supplementary Table S2: Listing of SNPs featured in the 7[illegible]

Lineage [1]	Strain name	688	696	705	717	738	739	741	745	752	756	759	762	768	771	774
2	MuVi/Shanxi.CHN/52.10/2	T	C	C	G	T	G	A	G	A	A	C	A	T	T	T
2	MuVi/Shannxi.CHN/26.09/4	T	C	C	G	T	G	A	G	A	A	C	A	T	T	C
2	MuVi/Shanxi.CHN/52.10/1	T	C	C	G	T	G	A	G	A	A	C	A	T	T	C
2	MuVi/Guangdong.CHN/21.09/1	T	C	C	G	T	G	A	G	A	A	C	A	T	T	C
2	MuVi/Liaoning.CHN/48.10/2	T	C	C	G	T	G	A	G	A	A	C	A	T	T	C
2	MuVi/Shandong.CHN/51.15/1	T	C	C	G	T	G	A	G	A	A	C	A	T	T	C
2	MuVi/Heilongjiang.CHN/49.15	T	C	C	G	T	G	A	G	A	A	C	A	T	T	C
2	MuVi/Anhui.CHN/10.11/1	T	C	C	G	T	G	A	G	A	A	C	A	T	T	C
2	MuVi/Shannxi.CHN/20.12	T	C	C	G	T	G	A	G	A	A	C	A	T	T	C
3	MuVi/Shanxi.CHN/52.10/3	T	C	C	G	T	G	A	G	A	A	C	A	T	T	C
3	MuVi/Jiangsu.CHN/3.13/1	T	C	C	G	T	G	A	G	A	A	C	A	T	T	C
3	MuVi/Shandong.CHN/4.05	T	C	C	G	T	G	A	G	A	A	C	A	C	T	C
3	MuVi/Shannxi.CHN/9.09/2	T	C	C	G	T	G	A	G	A	A	C	A	T	T	C
3	MuVi/Incheon.KOR/16.08/22	T	C	C	G	T	G	A	G	A	A	C	A	T	T	C
3	MuVi/Henan.CHN/48.06	T	C	C	G	T	G	A	G	A	A	C	A	T	T	C
3	MuVi/Jiangsu.CHN/9.12	T	C	C	G	T	G	A	G	A	A	C	A	T	T	C
3	MuVi/Jiangsu.CHN/4.13/3	T	C	C	G	T	G	A	G	A	A	C	A	T	T	C
4	MuVi/Shannxi.CHN/26.09/1	T	C	C	G	C	G	A	G	A	A	C	A	T	T	C
4	MuVi/Jiangsu.CHN/15.12	T	C	C	G	C	G	A	G	G	A	C	A	T	T	T
1	MuVi/Jilin.CHN/15.08/5	T	C	C	A	T	G	A	G	A	A	C	A	T	T	C
1	MuVi/Jilin.CHN/15.08/1	T	C	C	A	T	G	A	G	A	A	C	A	T	T	C
1	MuVi/Jilin.CHN/15.08/3	T	C	C	A	T	G	A	G	A	A	C	A	T	T	C
1	MuVi/Yunnan.CHN/47.10/2	T	C	C	A	T	G	A	G	A	A	C	A	T	T	C
1	MuVi/Neimeng.CHN/18.11	T	C	C	A	T	G	A	G	A	A	C	A	T	T	C
1	MuVi/Sichuan.CHN/23.12/1	T	C	C	A	T	G	A	G	A	A	C	A	T	T	C
1	MuVi/Jiangsu.CHN/3.13/2	T	T	C	A	T	G	A	G	A	A	C	A	T	T	T
1	MuVi/Beijing.CHN/25.06	T	C	C	A	T	G	A	G	A	A	C	A	T	T	C
1	MuVi/Zhejiang.CHN/26.05	T	C	C	A	T	G	A	G	A	A	C	A	T	T	C
1	MuVi/Shanghai.CHN/0.01	T	C	C	A	T	G	A	G	A	A	C	A	T	T	C
1	MuVi/Shandong.CHN/3.05	T	C	C	A	T	G	A	G	A	A	T	A	T	T	C
1	MuVi/Gansu.CHN/0.02	T	C	C	G	T	G	A	G	A	A	C	A	T	T	C
1	MuVi/Beijing.CHN/15.06	T	C	C	G	T	G	A	G	A	G	C	A	T	T	C
1	MuVi/Shandong.CHN/11.07	T	C	C	G	T	G	A	G	A	G	C	A	T	T	C
1	MuVi/Sichuan.CHN/24.15	T	C	C	G	T	G	A	A	A	G	C	C	T	C	C
1	MuVi/Heilongjiang.CHN/24.14	T	C	C	G	T	G	A	A	A	G	C	C	T	C	C
1	MuVi/Shandong.CHN/43.07	T	C	C	G	T	G	A	A	A	G	C	C	T	T	C
1	MuVi/Shandong.CHN/10.08	T	C	C	G	T	G	A	A	A	G	C	C	T	T	C
1	MuVi/Heilongjiang.CHN/26.12/2	T	C	C	G	T	G	A	A	A	G	C	C	T	T	C
1	MuVi/Liaoning.CHN/48.11	T	C	C	G	T	G	A	A	A	G	C	C	T	T	C
1	MuVi/Heilongjiang.CHN/23.13/1	T	C	C	G	T	G	A	A	A	G	C	C	T	T	C
1	MuVi/Liaoning.CHN/50.11	T	C	C	G	T	G	A	A	A	G	C	C	T	T	C
1	MuVs/Kaohsiung.TWN/06.08	T	C	C	G	T	G	A	A	A	G	C	A	T	T	C
	MuVi/Jiangsu.CHN/12.13/2	T	C	C	G	T	G	A	G	A	A	C	A	T	T	C
	MuVi/Shannxi.CHN/20.15/1	T	C	T	G	T	G	A	G	A	A	C	A	T	T	C
	MuVi/Beijing.CHN/10.07	T	C	C	G	T	G	A	G	A	A	C	A	T	T	C
	MuVi/Hunan.CHN/30.11/3	T	C	C	G	T	G	A	G	A	A	C	A	T	T	C
	MuVi/Liaoning.CHN/9.10/1	T	C	C	G	T	G	C	G	A	A	C	A	T	T	C
	MuVi/Jiangsu.CHN/7.12	T	C	C	G	T	G	A	G	A	A	C	A	T	T	C
	MuVi/Heilongjiang.CHN/14.13/1	T	C	C	G	T	G	A	G	A	A	C	A	T	T	C
	MuVi/Hunan.CHN/32.11	T	C	C	T	T	G	A	G	A	A	C	G	T	T	C
	MuVi/Hunan.CHN/30.11/1	T	C	C	G	T	G	A	G	A	A	C	A	T	T	C
	MuVi/Jiangsu.CHN/7.13/1	T	C	C	G	T	G	A	G	A	A	C	A	T	T	C
	MuVi/Hunan.CHN/30.11/2	T	C	C	G	T	G	A	G	A	A	C	A	T	T	C
	MuVi/Liaoning.CHN/5.09/2	T	C	C	G	T	G	A	A	A	A	C	A	T	T	C
	Mui/Zhejiang.CHN/11.06/1	C	C	C	G	T	G	A	G	A	A	C	A	T	T	C
	MuVi/Liaoning.CHN/13.12	T	C	C	G	T	G	A	G	A	A	C	A	T	T	C
	MuVi/Beijing.CHN/17.07/2	T	C	C	G	T	G	A	G	A	A	T	A	T	T	C
	MuVi/Liaoning.CHN/7.12	T	C	C	G	T	G	A	G	A	A	C	A	T	T	C
	MuVi/Beijing.CHN/21.11	T	C	C	G	T	G	A	G	A	A	C	A	T	T	C
	MuVi/Liaoning.CHN/11.12	T	C	C	G	T	G	A	G	A	A	C	A	T	T	C
	MuVi/Heilongjiang.CHN/26.12/1	T	C	C	G	T	G	A	G	A	A	C	A	T	T	C
	MuVi/Liaoning.CHN/1.08/1	T	C	C	G	T	G	A	G	A	A	C	A	T	T	C
	MuVi/Liaoning.CHN/48.10/1	T	C	C	G	T	G	A	G	A	A	C	A	T	T	C
	MuVi/Jiangsu.CHN/4.13/5	T	C	C	G	T	G	A	G	A	A	C	A	T	T	T
	MuVi/Liaoning.CHN/10.09	T	C	C	G	T	G	A	G	A	A	C	A	T	T	C
	MuVi/Liaoning.CHN/1.08/2	T	C	C	G	T	G	A	G	A	A	C	A	T	T	C
	MuVi/Liaoning.CHN/5.09/1	T	C	C	G	T	G	A	G	A	A	C	A	T	T	C
	MuVi/Heilongjiang.CHN/22.12	T	C	C	G	T	G	A	G	A	A	C	A	T	T	C
	MuVi/Heilongjiang.CHN/15.13	T	C	C	G	T	G	A	G	A	A	C	A	T	T	C
	MuVi/Sichuan.CHN/23.12/2	T	C	C	G	T	A	A	G	A	A	C	A	T	T	C
	MuVi/Hubei.CHN/44.12/1	T	C	C	G	T	G	A	G	A	A	C	A	T	T	C
	MuVi/Jiangsu.CHN/2.13/4	T	C	C	G	T	G	A	G	A	A	C	A	T	T	C

1: Lineage based on Figure 1
2: SNPs are noted in green.
3: SNP position corresponding to a lineage-defining node i[illegible]

Supplementary Table S2: Listing of SNPs featured in the 7

Lineage [1]	Strain name	780	786	795	807	810	812	813	819	824	828	831	840	843	849	855
2	MuVi/Shanxi.CHN/52.10/2	A	A	C	A	G	C	C	A	C	G	G	G	A	T	C
2	MuVi/Shannxi.CHN/26.09/4	A	A	C	A	G	C	C	A	C	G	A	G	A	T	C
2	MuVi/Shanxi.CHN/52.10/1	A	A	C	A	G	C	C	A	C	G	A	G	A	T	C
2	MuVi/Guangdong.CHN/21.09/1	A	A	C	A	G	C	C	A	C	G	A	G	A	T	C
2	MuVi/Liaoning.CHN/48.10/2	A	A	C	A	G	C	C	A	C	G	A	G	A	T	C
2	MuVi/Shandong.CHN/51.15/1	A	A	C	A	G	C	C	A	C	G	A	G	A	T	C
2	MuVi/Heilongjiang.CHN/49.15	A	A	C	A	A	T	C	A	C	G	A	G	A	C	C
2	MuVi/Anhui.CHN/10.11/1	A	A	C	A	G	C	C	A	C	G	A	G	A	C	C
2	MuVi/Shannxi.CHN/20.12	A	A	C	A	G	C	C	A	C	G	A	G	A	C	C
3	MuVi/Shanxi.CHN/52.10/3	A	A	C	A	G	C	C	A	C	G	A	A	A	T	C
3	MuVi/Jiangsu.CHN/3.13/1	A	A	C	A	G	C	C	A	C	G	A	A	A	T	C
3	MuVi/Shandong.CHN/4.05	A	A	C	A	G	C	C	A	C	G	A	G	G	T	C
3	MuVi/Shannxi.CHN/9.09/2	A	A	C	A	G	C	C	A	C	G	A	G	A	T	C
3	MuVi/Incheon.KOR/16.08/22	A	A	C	A	G	C	C	A	C	G	A	G	G	T	C
3	MuVi/Henan.CHN/48.06	A	A	C	A	G	C	T	A	C	G	A	G	A	T	C
3	MuVi/Jiangsu.CHN/9.12	A	A	C	A	G	C	T	A	C	G	A	G	A	T	C
3	MuVi/Jiangsu.CHN/4.13/3	A	A	C	A	G	C	T	A	C	G	A	G	A	T	C
4	MuVi/Shannxi.CHN/26.09/1	T	A	C	G	G	C	C	A	C	G	A	G	A	T	C
4	MuVi/Jiangsu.CHN/15.12	A	A	C	G	G	C	C	A	C	G	A	G	A	T	C
1	MuVi/Jilin.CHN/15.08/5	A	A	C	A	G	C	C	A	C	A	A	G	A	T	C
1	MuVi/Jilin.CHN/15.08/1	A	A	C	A	G	C	C	A	C	A	A	G	A	T	C
1	MuVi/Jilin.CHN/15.08/3	A	A	C	A	G	C	C	A	C	A	A	G	A	T	C
1	MuVi/Yunnan.CHN/47.10/2	A	A	C	A	G	C	C	A	C	A	A	G	A	T	C
1	MuVi/Neimeng.CHN/18.11	G	A	C	A	G	C	C	A	C	A	A	G	A	T	C
1	MuVi/Sichuan.CHN/23.12/1	A	A	C	A	G	C	C	A	C	A	A	A	A	T	C
1	MuVi/Jiangsu.CHN/3.13/2	A	A	C	A	G	C	C	A	C	A	A	G	A	T	C
1	MuVi/Beijing.CHN/25.06	A	A	C	A	G	C	C	A	C	A	A	G	A	T	C
1	MuVi/Zhejiang.CHN/26.05	A	A	C	A	G	C	C	A	C	A	A	G	A	T	C
1	MuVi/Shanghai.CHN/0.01	A	A	C	A	G	C	C	A	C	A	A	G	A	T	C
1	MuVi/Shandong.CHN/3.05	A	A	C	A	G	C	C	A	C	A	A	G	A	T	C
1	MuVi/Gansu.CHN/0.02	A	A	C	A	G	C	C	A	C	A	A	G	A	T	C
1	MuVi/Beijing.CHN/15.06	A	A	C	A	G	C	C	A	C	A	A	G	A	T	C
1	MuVi/Shandong.CHN/11.07	A	A	C	A	G	C	C	A	C	A	A	G	A	T	C
1	MuVi/Sichuan.CHN/24.15	A	A	C	A	A	C	C	A	C	A	A	G	A	T	C
1	MuVi/Heilongjiang.CHN/24.14	A	A	C	A	A	C	C	A	C	A	A	G	A	T	C
1	MuVi/Shandong.CHN/43.07	A	A	C	T	A	C	C	A	C	A	A	G	A	T	C
1	MuVi/Shandong.CHN/10.08	A	A	C	T	A	C	C	A	C	A	A	G	A	T	C
1	MuVi/Heilongjiang.CHN/26.12/2	A	A	C	A	A	C	C	A	C	A	A	G	A	T	C
1	MuVi/Liaoning.CHN/48.11	A	A	C	A	A	C	C	A	C	A	A	G	A	T	C
1	MuVi/Heilongjiang.CHN/23.13/1	A	A	C	A	A	C	C	A	C	A	A	G	A	T	C
1	MuVi/Liaoning.CHN/50.11	A	A	C	A	A	C	C	A	C	A	A	G	A	T	C
1	MuVs/Kaohsiung.TWN/06.08	A	A	C	A	G	C	C	A	C	A	A	G	A	T	C
	MuVi/Jiangsu.CHN/12.13/2	A	G	C	A	G	C	C	A	T	G	A	G	A	T	C
	MuVi/Shannxi.CHN/20.15/1	A	G	C	A	G	C	C	A	T	G	A	G	A	T	C
	MuVi/Beijing.CHN/10.07	A	A	C	A	G	C	C	A	C	G	A	G	A	T	C
	MuVi/Hunan.CHN/30.11/3	A	G	C	A	G	C	C	A	C	G	A	G	A	T	C
	MuVi/Liaoning.CHN/9.10/1	A	G	C	A	G	C	C	A	C	G	A	G	A	T	C
	MuVi/Jiangsu.CHN/7.12	A	G	C	A	G	C	C	A	C	G	A	G	A	T	C
	MuVi/Heilongjiang.CHN/14.13/1	A	G	C	A	G	C	C	A	C	G	A	G	A	T	C
	MuVi/Hunan.CHN/32.11	A	A	C	A	G	C	C	A	C	G	A	G	A	T	T
	MuVi/Hunan.CHN/30.11/1	A	A	C	A	G	C	C	A	C	G	A	G	A	T	C
	MuVi/Jiangsu.CHN/7.13/1	A	A	C	A	G	C	C	A	C	G	A	G	A	T	C
	MuVi/Hunan.CHN/30.11/2	A	G	C	A	G	C	C	A	C	G	A	G	A	T	C
	MuVi/Liaoning.CHN/5.09/2	A	A	C	A	G	C	C	A	C	G	A	G	A	T	C
	Mui/Zhejiang.CHN/11.06/1	A	A	C	A	G	C	C	A	C	G	A	G	A	T	C
	MuVi/Liaoning.CHN/13.12	A	G	C	A	G	C	C	A	C	G	A	G	A	T	C
	MuVi/Beijing.CHN/17.07/2	A	G	C	A	G	C	C	A	C	G	A	G	A	T	C
	MuVi/Liaoning.CHN/7.12	A	G	C	A	G	C	C	A	C	G	A	G	A	T	C
	MuVi/Beijing.CHN/21.11	A	G	C	A	G	C	C	A	C	G	A	G	A	T	C
	MuVi/Liaoning.CHN/11.12	A	G	C	A	G	C	C	A	C	G	A	G	A	T	C
	MuVi/Heilongjiang.CHN/26.12/1	A	G	C	A	G	C	C	A	C	G	A	G	A	T	C
	MuVi/Liaoning.CHN/1.08/1	A	G	C	A	G	C	C	A	C	G	A	G	A	T	C
	MuVi/Liaoning.CHN/48.10/1	A	G	C	A	G	C	C	A	C	G	A	G	A	T	C
	MuVi/Jiangsu.CHN/4.13/5	A	G	C	A	G	C	C	A	C	G	A	G	A	T	C
	MuVi/Liaoning.CHN/10.09	A	G	C	A	G	C	C	A	C	G	A	G	A	T	C
	MuVi/Liaoning.CHN/1.08/2	A	G	C	A	G	C	C	A	C	G	A	G	A	T	C
	MuVi/Liaoning.CHN/5.09/1	A	G	C	A	G	C	C	A	C	G	A	G	A	T	C
	MuVi/Heilongjiang.CHN/22.12	A	G	T	A	G	C	C	A	C	G	A	G	A	T	C
	MuVi/Heilongjiang.CHN/15.13	A	G	T	A	G	C	C	A	C	G	A	G	A	T	C
	MuVi/Sichuan.CHN/23.12/2	A	G	C	A	G	C	C	A	C	G	A	G	A	T	C
	MuVi/Hubei.CHN/44.12/1	A	G	C	A	G	C	C	G	C	G	A	G	A	T	C
	MuVi/Jiangsu.CHN/2.13/4	A	G	C	A	G	T	C	A	C	G	A	G	A	T	C

1: Lineage based on Figure 1
2: SNPs are noted in green.
3: SNP position corresponding to a lineage-defining node ir

Supplementary Table S2: Listing of SNPs featured in the 7

Lineage [1]	Strain name	858	859	861	863	867	879	882	885	900	905	906	909	912	915	918
2	MuVi/Shanxi.CHN/52.10/2	C	G	C	C	A	T	A	T	T	C	T	T	G	A	A
2	MuVi/Shannxi.CHN/26.09/4	C	G	C	C	A	T	A	T	T	C	T	T	G	A	A
2	MuVi/Shanxi.CHN/52.10/1	C	G	T	C	A	T	A	T	T	C	T	T	G	A	A
2	MuVi/Guangdong.CHN/21.09/1	C	G	C	C	A	T	A	A	T	C	T	T	G	A	A
2	MuVi/Liaoning.CHN/48.10/2	C	G	C	C	A	T	A	T	T	C	T	T	G	A	A
2	MuVi/Shandong.CHN/51.15/1	C	G	C	C	A	T	A	T	T	C	T	T	G	A	A
2	MuVi/Heilongjiang.CHN/49.15	C	G	C	C	A	T	A	T	T	C	T	T	G	A	A
2	MuVi/Anhui.CHN/10.11/1	C	G	C	C	A	T	A	T	T	T	T	T	G	A	A
2	MuVi/Shannxi.CHN/20.12	C	G	C	C	A	T	A	T	T	T	T	T	G	A	A
3	MuVi/Shanxi.CHN/52.10/3	C	G	C	C	A	T	A	T	T	C	T	T	G	G	A
3	MuVi/Jiangsu.CHN/3.13/1	C	G	C	C	A	T	A	T	T	C	T	T	G	G	A
3	MuVi/Shandong.CHN/4.05	C	G	C	C	A	T	A	T	T	C	T	T	G	G	A
3	MuVi/Shannxi.CHN/9.09/2	C	A	C	C	A	T	A	T	T	C	T	T	G	G	A
3	MuVi/Incheon.KOR/16.08/22	C	G	C	C	A	T	A	T	T	C	T	T	G	G	A
3	MuVi/Henan.CHN/48.06	C	G	C	C	A	T	A	T	T	C	T	T	G	G	A
3	MuVi/Jiangsu.CHN/9.12	C	G	C	C	A	T	A	T	T	C	T	T	G	G	A
3	MuVi/Jiangsu.CHN/4.13/3	C	G	C	C	A	T	A	T	T	C	T	T	G	G	A
4	MuVi/Shannxi.CHN/26.09/1	C	G	C	C	A	A	A	T	T	C	T	T	G	G	A
4	MuVi/Jiangsu.CHN/15.12	C	G	C	C	A	A	A	T	T	C	T	T	G	G	A
1	MuVi/Jilin.CHN/15.08/5	C	G	C	C	A	T	A	T	T	C	T	T	G	G	A
1	MuVi/Jilin.CHN/15.08/1	C	G	C	C	A	T	A	T	T	C	T	T	G	G	A
1	MuVi/Jilin.CHN/15.08/3	C	G	C	C	A	T	A	T	T	C	T	T	G	G	A
1	MuVi/Yunnan.CHN/47.10/2	C	G	C	C	A	T	A	T	T	C	T	T	G	G	A
1	MuVi/Neimeng.CHN/18.11	C	G	C	C	A	T	A	T	T	C	T	T	G	G	A
1	MuVi/Sichuan.CHN/23.12/1	C	G	C	C	A	T	A	T	T	C	T	T	G	G	T
1	MuVi/Jiangsu.CHN/3.13/2	C	G	C	C	A	T	A	T	T	C	T	T	G	G	A
1	MuVi/Beijing.CHN/25.06	C	G	C	C	A	T	A	T	T	C	T	C	G	G	A
1	MuVi/Zhejiang.CHN/26.05	C	G	C	C	A	T	A	T	T	C	A	T	G	G	A
1	MuVi/Shanghai.CHN/0.01	C	G	C	C	A	T	A	T	T	C	T	T	G	G	A
1	MuVi/Shandong.CHN/3.05	C	G	C	C	A	T	A	T	T	C	T	T	G	G	A
1	MuVi/Gansu.CHN/0.02	C	G	C	C	A	T	A	T	T	C	T	T	G	G	A
1	MuVi/Beijing.CHN/15.06	T	G	C	C	A	A	A	T	T	C	T	T	G	G	A
1	MuVi/Shandong.CHN/11.07	T	G	C	C	A	T	A	T	T	C	T	T	G	G	A
1	MuVi/Sichuan.CHN/24.15	T	G	C	T	A	T	A	T	T	C	T	T	G	G	A
1	MuVi/Heilongjiang.CHN/24.14	T	G	C	T	A	T	A	T	T	C	T	T	G	G	A
1	MuVi/Shandong.CHN/43.07	T	G	C	T	A	C	A	T	T	C	T	T	G	G	A
1	MuVi/Shandong.CHN/10.08	T	G	C	T	A	C	A	T	T	C	T	T	G	G	A
1	MuVi/Heilongjiang.CHN/26.12/2	T	G	C	T	A	T	A	T	T	C	T	T	G	G	A
1	MuVi/Liaoning.CHN/48.11	T	G	C	T	A	T	A	T	T	C	T	T	G	G	A
1	MuVi/Heilongjiang.CHN/23.13/1	T	G	C	T	A	T	A	T	T	C	T	T	G	G	A
1	MuVi/Liaoning.CHN/50.11	T	G	C	T	A	T	A	T	T	C	T	T	G	G	A
1	MuVs/Kaohsiung.TWN/06.08	T	G	C	C	A	T	A	T	T	C	T	T	G	G	A
	MuVi/Jiangsu.CHN/12.13/2	C	A	C	C	A	T	A	T	T	C	T	T	A	A	A
	MuVi/Shannxi.CHN/20.15/1	C	G	C	C	A	T	A	T	T	C	T	T	A	A	A
	MuVi/Beijing.CHN/10.07	C	G	C	C	A	T	T	T	T	C	T	T	G	G	A
	MuVi/Hunan.CHN/30.11/3	C	G	C	C	G	T	A	T	T	C	C	T	G	A	A
	MuVi/Liaoning.CHN/9.10/1	C	G	C	C	A	T	A	T	T	C	C	T	G	A	A
	MuVi/Jiangsu.CHN/7.12	C	G	C	C	A	T	A	T	T	C	C	T	G	A	A
	MuVi/Heilongjiang.CHN/14.13/1	C	G	C	C	A	T	A	T	T	C	C	T	G	A	A
	MuVi/Hunan.CHN/32.11	C	G	C	T	A	T	A	T	C	C	T	T	G	A	A
	MuVi/Hunan.CHN/30.11/1	C	G	C	C	A	T	A	T	T	C	T	T	G	A	A
	MuVi/Jiangsu.CHN/7.13/1	C	G	C	C	A	T	A	T	T	C	T	T	G	A	A
	MuVi/Hunan.CHN/30.11/2	C	G	C	C	A	T	A	T	T	C	T	T	G	A	A
	MuVi/Liaoning.CHN/5.09/2	T	G	C	C	A	T	A	T	T	C	T	T	G	A	A
	Mui/Zhejiang.CHN/11.06/1	T	G	C	C	A	T	A	T	T	C	T	T	G	A	A
	MuVi/Liaoning.CHN/13.12	C	G	C	C	A	T	A	T	T	C	T	T	G	G	A
	MuVi/Beijing.CHN/17.07/2	C	G	C	C	A	T	A	T	T	C	T	T	G	A	A
	MuVi/Liaoning.CHN/7.12	C	G	C	C	A	T	A	T	T	C	T	T	G	A	A
	MuVi/Beijing.CHN/21.11	C	G	C	C	A	T	A	T	T	C	C	T	G	A	A
	MuVi/Liaoning.CHN/11.12	C	G	C	C	A	T	A	T	T	C	C	T	G	A	A
	MuVi/Heilongjiang.CHN/26.12/1	C	G	C	C	A	T	A	T	T	C	T	T	G	A	A
	MuVi/Liaoning.CHN/1.08/1	C	G	C	C	A	T	A	T	T	C	T	T	G	A	A
	MuVi/Liaoning.CHN/48.10/1	C	G	C	C	A	T	A	T	T	C	T	T	G	A	A
	MuVi/Jiangsu.CHN/4.13/5	C	G	C	C	A	T	A	T	T	C	T	T	G	A	A
	MuVi/Liaoning.CHN/10.09	C	G	C	C	A	T	A	T	T	C	T	T	G	A	A
	MuVi/Liaoning.CHN/1.08/2	C	G	C	C	A	T	A	T	T	C	T	T	G	A	A
	MuVi/Liaoning.CHN/5.09/1	C	G	C	C	A	T	A	T	T	C	T	T	G	A	A
	MuVi/Heilongjiang.CHN/22.12	C	G	C	C	A	C	A	T	T	C	T	T	G	A	A
	MuVi/Heilongjiang.CHN/15.13	C	G	C	C	A	C	A	T	T	C	T	T	G	A	A
	MuVi/Sichuan.CHN/23.12/2	C	G	C	C	A	T	A	T	T	C	T	T	G	A	A
	MuVi/Hubei.CHN/44.12/1	C	G	C	C	A	T	A	T	T	C	T	T	G	A	A
	MuVi/Jiangsu.CHN/2.13/4	C	G	C	C	A	T	A	T	T	C	T	T	G	A	A

1: Lineage based on Figure 1
2: SNPs are noted in green.
3: SNP position corresponding to a lineage-defining node i

Supplementary Table S2: Listing of SNPs featured in the 7

Lineage [1]	Strain name	921	930	934	943	951	954	963	966	969	978	979	981	984	S<br>1002
2	MuVi/Shanxi.CHN/52.10/2	A	T	A	A	A	G	T	A	T	C	T	G	C	A
2	MuVi/Shannxi.CHN/26.09/4	A	T	A	G	A	G	T	A	T	C	T	G	C	T
2	MuVi/Shanxi.CHN/52.10/1	A	T	A	G	A	G	T	A	T	C	T	G	C	T
2	MuVi/Guangdong.CHN/21.09/1	A	T	A	G	A	G	T	A	T	C	T	G	C	T
2	MuVi/Liaoning.CHN/48.10/2	A	T	A	G	A	G	T	G	T	C	T	G	C	T
2	MuVi/Shandong.CHN/51.15/1	A	T	A	G	A	G	T	G	T	C	T	G	C	T
2	MuVi/Heilongjiang.CHN/49.15	A	T	A	G	A	G	T	G	T	C	T	G	C	T
2	MuVi/Anhui.CHN/10.11/1	A	T	G	G	A	G	T	G	T	C	T	G	C	T
2	MuVi/Shannxi.CHN/20.12	A	T	G	G	A	G	T	G	T	C	T	G	C	T
3	MuVi/Shanxi.CHN/52.10/3	A	T	A	G	A	G	T	A	T	C	T	G	C	T
3	MuVi/Jiangsu.CHN/3.13/1	A	T	A	G	A	G	T	A	T	C	T	G	C	T
3	MuVi/Shandong.CHN/4.05	A	T	A	G	A	G	T	A	T	C	T	G	C	T
3	MuVi/Shannxi.CHN/9.09/2	A	T	A	G	A	G	T	A	T	C	T	G	C	T
3	MuVi/Incheon.KOR/16.08/22	A	T	A	G	A	G	T	A	T	T	T	G	C	T
3	MuVi/Henan.CHN/48.06	A	T	A	G	A	G	T	A	T	C	T	G	C	T
3	MuVi/Jiangsu.CHN/9.12	A	T	A	G	A	G	T	A	T	C	T	G	C	T
3	MuVi/Jiangsu.CHN/4.13/3	A	T	A	G	A	G	T	A	T	C	T	G	C	T
4	MuVi/Shannxi.CHN/26.09/1	A	T	A	G	A	G	T	A	T	C	T	G	C	T
4	MuVi/Jiangsu.CHN/15.12	A	T	A	G	A	G	T	A	T	C	T	G	C	T
1	MuVi/Jilin.CHN/15.08/5	A	T	A	G	A	G	T	A	T	C	T	G	C	T
1	MuVi/Jilin.CHN/15.08/1	A	T	A	G	A	G	T	A	T	C	T	G	C	T
1	MuVi/Jilin.CHN/15.08/3	A	T	A	G	A	G	T	A	T	C	T	G	C	T
1	MuVi/Yunnan.CHN/47.10/2	A	T	A	G	A	G	T	A	T	C	T	G	C	T
1	MuVi/Neimeng.CHN/18.11	A	T	A	G	A	G	T	A	T	C	T	G	C	T
1	MuVi/Sichuan.CHN/23.12/1	A	T	A	G	A	A	T	A	T	C	T	G	C	T
1	MuVi/Jiangsu.CHN/3.13/2	A	T	A	G	A	G	T	A	T	C	T	G	C	T
1	MuVi/Beijing.CHN/25.06	A	T	A	G	A	G	T	A	T	C	T	G	C	T
1	MuVi/Zhejiang.CHN/26.05	A	T	A	G	A	G	T	A	T	C	T	G	C	T
1	MuVi/Shanghai.CHN/0.01	A	T	A	G	A	G	T	A	T	C	T	G	C	T
1	MuVi/Shandong.CHN/3.05	A	T	A	G	A	G	T	A	T	C	T	G	C	T
1	MuVi/Gansu.CHN/0.02	A	T	A	G	A	G	T	A	T	C	T	G	C	T
1	MuVi/Beijing.CHN/15.06	A	T	A	G	A	G	T	A	T	C	C	G	C	T
1	MuVi/Shandong.CHN/11.07	A	T	A	G	A	G	T	A	T	C	C	G	C	T
1	MuVi/Sichuan.CHN/24.15	A	T	A	G	A	G	T	A	T	C	C	G	T	T
1	MuVi/Heilongjiang.CHN/24.14	A	T	A	G	A	G	C	A	C	C	C	G	T	T
1	MuVi/Shandong.CHN/43.07	A	T	A	G	A	G	T	A	T	C	C	G	C	T
1	MuVi/Shandong.CHN/10.08	A	T	A	G	A	G	T	A	T	C	C	G	C	T
1	MuVi/Heilongjiang.CHN/26.12/2	A	T	A	G	A	G	T	A	T	C	C	G	C	T
1	MuVi/Liaoning.CHN/48.11	A	T	A	G	A	G	T	A	T	C	C	G	C	T
1	MuVi/Heilongjiang.CHN/23.13/1	A	T	A	G	A	G	T	A	T	C	C	G	C	T
1	MuVi/Liaoning.CHN/50.11	A	T	A	G	A	G	T	A	T	C	C	G	C	T
1	MuVs/Kaohsiung.TWN/06.08	A	T	G	G	A	G	T	A	T	C	C	G	C	T
	MuVi/Jiangsu.CHN/12.13/2	A	T	A	G	A	G	T	A	T	C	T	G	T	T
	MuVi/Shannxi.CHN/20.15/1	A	T	A	G	A	G	T	A	T	C	T	G	T	T
	MuVi/Beijing.CHN/10.07	A	T	A	G	A	G	T	A	T	C	T	G	C	T
	MuVi/Hunan.CHN/30.11/3	A	T	A	G	A	G	T	A	T	C	T	G	T	T
	MuVi/Liaoning.CHN/9.10/1	A	T	A	G	A	G	T	A	T	C	T	G	T	T
	MuVi/Jiangsu.CHN/7.12	A	T	A	G	A	G	T	A	T	C	T	G	T	T
	MuVi/Heilongjiang.CHN/14.13/1	A	T	A	G	A	G	T	A	T	C	T	G	T	T
	MuVi/Hunan.CHN/32.11	G	C	A	G	A	G	T	A	T	C	T	G	C	T
	MuVi/Hunan.CHN/30.11/1	A	T	A	G	A	G	T	A	T	C	T	G	C	T
	MuVi/Jiangsu.CHN/7.13/1	A	T	A	G	A	G	T	A	T	C	T	G	C	T
	MuVi/Hunan.CHN/30.11/2	A	T	A	G	A	G	T	A	T	C	T	A	C	T
	MuVi/Liaoning.CHN/5.09/2	G	T	G	G	A	G	T	A	T	C	T	G	C	T
	Mui/Zhejiang.CHN/11.06/1	A	T	A	G	A	G	T	A	T	C	T	G	C	T
	MuVi/Liaoning.CHN/13.12	A	T	A	G	A	G	T	A	T	C	T	G	C	T
	MuVi/Beijing.CHN/17.07/2	A	T	A	G	A	G	T	A	T	C	T	G	C	T
	MuVi/Liaoning.CHN/7.12	A	T	A	G	A	G	T	G	T	C	T	G	C	T
	MuVi/Beijing.CHN/21.11	A	T	A	G	G	G	T	A	T	C	T	G	C	T
	MuVi/Liaoning.CHN/11.12	A	T	A	G	G	G	T	A	T	C	T	G	C	T
	MuVi/Heilongjiang.CHN/26.12/1	A	T	A	G	G	G	T	A	T	C	T	G	C	T
	MuVi/Liaoning.CHN/1.08/1	A	T	A	G	A	G	T	A	T	C	T	G	C	T
	MuVi/Liaoning.CHN/48.10/1	A	T	A	G	A	G	T	A	T	C	T	G	C	T
	MuVi/Jiangsu.CHN/4.13/5	A	T	A	G	A	G	T	A	T	C	T	G	C	T
	MuVi/Liaoning.CHN/10.09	A	T	A	G	A	G	T	A	T	C	T	G	C	T
	MuVi/Liaoning.CHN/1.08/2	A	T	A	G	A	G	T	A	T	C	T	G	C	T
	MuVi/Liaoning.CHN/5.09/1	A	T	A	G	A	G	T	A	T	C	T	G	C	T
	MuVi/Heilongjiang.CHN/22.12	A	T	A	G	A	G	T	A	T	C	T	G	C	T
	MuVi/Heilongjiang.CHN/15.13	A	T	A	G	A	G	T	A	T	C	T	G	C	T
	MuVi/Sichuan.CHN/23.12/2	A	T	A	G	A	G	T	A	T	C	T	G	C	T
	MuVi/Hubei.CHN/44.12/1	A	T	A	G	A	G	T	A	T	C	T	G	C	T
	MuVi/Jiangsu.CHN/2.13/4	A	T	A	G	A	G	T	A	T	C	T	G	C	T

1: Lineage based on Figure 1
2: SNPs are noted in green.
3: SNP position corresponding to a lineage-defining node ir

Supplementary Table S2: Listing of SNPs featured in the 7

Lineage [1]	Strain name	NP position in HN											
		1005	1012	1013	1026	1029	1032	1041	1047	1056	1060	1064	1065
2	MuVi/Shanxi.CHN/52.10/2	A	A	G	A	T	T	T	A	A	C	A	A
2	MuVi/Shannxi.CHN/26.09/4	A	A	G	A	T	T	T	A	A	C	A	A
2	MuVi/Shanxi.CHN/52.10/1	A	A	G	A	T	T	T	A	A	C	A	A
2	MuVi/Guangdong.CHN/21.09/1	A	A	G	A	T	T	T	A	A	C	A	A
2	MuVi/Liaoning.CHN/48.10/2	A	A	G	A	T	T	T	A	A	C	A	A
2	MuVi/Shandong.CHN/51.15/1	A	A	G	A	T	T	T	A	A	C	A	A
2	MuVi/Heilongjiang.CHN/49.15	A	A	G	A	T	T	T	A	A	A	A	A
2	MuVi/Anhui.CHN/10.11/1	A	A	G	A	T	T	T	A	A	A	A	A
2	MuVi/Shannxi.CHN/20.12	A	A	G	A	T	T	T	A	A	A	A	A
3	MuVi/Shanxi.CHN/52.10/3	A	A	G	G	T	T	T	A	A	C	A	A
3	MuVi/Jiangsu.CHN/3.13/1	A	A	G	G	T	T	T	A	A	C	A	A
3	MuVi/Shandong.CHN/4.05	G	A	G	G	T	T	T	A	A	C	A	A
3	MuVi/Shannxi.CHN/9.09/2	G	A	G	G	T	T	T	A	A	C	A	A
3	MuVi/Incheon.KOR/16.08/22	G	A	G	G	T	T	T	A	A	C	A	A
3	MuVi/Henan.CHN/48.06	A	A	G	G	T	T	T	A	G	C	A	A
3	MuVi/Jiangsu.CHN/9.12	A	A	G	G	T	T	T	A	G	C	A	A
3	MuVi/Jiangsu.CHN/4.13/3	A	A	G	G	T	T	T	A	G	C	A	A
4	MuVi/Shannxi.CHN/26.09/1	A	A	G	G	T	T	C	A	A	C	T	A
4	MuVi/Jiangsu.CHN/15.12	A	A	G	G	T	T	T	A	A	C	A	A
1	MuVi/Jilin.CHN/15.08/5	A	A	G	G	C	T	T	A	A	C	A	A
1	MuVi/Jilin.CHN/15.08/1	A	A	G	G	C	T	T	A	A	C	A	A
1	MuVi/Jilin.CHN/15.08/3	A	A	G	G	C	T	T	A	A	C	A	A
1	MuVi/Yunnan.CHN/47.10/2	A	A	G	G	C	T	T	A	A	C	A	A
1	MuVi/Neimeng.CHN/18.11	A	A	G	G	C	T	T	A	A	C	A	A
1	MuVi/Sichuan.CHN/23.12/1	A	A	G	G	C	T	T	A	A	C	A	A
1	MuVi/Jiangsu.CHN/3.13/2	A	A	G	A	C	T	T	G	A	C	A	A
1	MuVi/Beijing.CHN/25.06	A	G	G	A	C	T	T	A	A	C	A	A
1	MuVi/Zhejiang.CHN/26.05	A	A	G	A	C	T	T	A	A	C	A	A
1	MuVi/Shanghai.CHN/0.01	A	A	G	A	C	T	T	A	A	C	A	A
1	MuVi/Shandong.CHN/3.05	A	A	G	A	C	T	T	A	A	C	A	A
1	MuVi/Gansu.CHN/0.02	A	A	G	A	C	T	T	A	A	C	A	A
1	MuVi/Beijing.CHN/15.06	A	A	G	A	C	T	T	A	A	C	A	A
1	MuVi/Shandong.CHN/11.07	A	A	G	A	C	T	T	A	A	C	A	A
1	MuVi/Sichuan.CHN/24.15	A	A	G	A	C	T	T	A	A	C	A	A
1	MuVi/Heilongjiang.CHN/24.14	A	A	G	A	C	T	T	A	A	C	A	A
1	MuVi/Shandong.CHN/43.07	A	A	G	A	C	T	T	A	A	C	A	A
1	MuVi/Shandong.CHN/10.08	A	A	G	A	C	T	T	A	A	C	A	A
1	MuVi/Heilongjiang.CHN/26.12/2	A	A	G	A	C	T	T	A	A	C	A	A
1	MuVi/Liaoning.CHN/48.11	A	A	G	A	C	T	T	A	A	C	A	A
1	MuVi/Heilongjiang.CHN/23.13/1	A	A	G	A	C	T	T	A	A	C	A	A
1	MuVi/Liaoning.CHN/50.11	A	A	G	A	C	T	T	A	A	C	A	A
1	MuVs/Kaohsiung.TWN/06.08	A	A	G	A	C	T	T	A	A	C	A	A
	MuVi/Jiangsu.CHN/12.13/2	A	A	G	A	T	T	T	A	A	C	A	A
	MuVi/Shannxi.CHN/20.15/1	A	A	G	A	T	T	T	A	A	C	A	A
	MuVi/Beijing.CHN/10.07	A	A	G	A	C	T	T	A	A	C	A	A
	MuVi/Hunan.CHN/30.11/3	A	A	G	A	T	T	T	A	A	C	A	A
	MuVi/Liaoning.CHN/9.10/1	A	A	G	A	T	T	T	A	A	C	A	A
	MuVi/Jiangsu.CHN/7.12	A	A	G	A	T	T	T	A	A	C	A	A
	MuVi/Heilongjiang.CHN/14.13/1	A	A	G	A	T	T	T	A	A	C	A	A
	MuVi/Hunan.CHN/32.11	A	A	G	A	T	C	C	A	A	C	A	A
	MuVi/Hunan.CHN/30.11/1	A	A	G	A	T	T	T	A	A	C	A	A
	MuVi/Jiangsu.CHN/7.13/1	A	A	A	A	T	T	T	A	A	C	A	A
	MuVi/Hunan.CHN/30.11/2	A	A	G	A	T	T	T	A	A	C	A	A
	MuVi/Liaoning.CHN/5.09/2	A	A	G	A	T	T	T	A	A	C	A	A
	Mui/Zhejiang.CHN/11.06/1	A	A	G	A	T	T	T	A	A	C	A	A
	MuVi/Liaoning.CHN/13.12	A	A	G	A	T	T	T	A	A	C	A	A
	MuVi/Beijing.CHN/17.07/2	A	A	A	A	T	T	T	A	A	C	A	A
	MuVi/Liaoning.CHN/7.12	A	A	G	A	T	T	T	A	A	C	A	A
	MuVi/Beijing.CHN/21.11	A	A	G	A	T	T	T	A	A	C	A	A
	MuVi/Liaoning.CHN/11.12	A	A	G	A	T	T	T	A	A	C	A	A
	MuVi/Heilongjiang.CHN/26.12/1	A	A	G	A	T	T	T	A	A	C	A	A
	MuVi/Liaoning.CHN/1.08/1	A	A	G	A	T	T	T	A	A	C	A	A
	MuVi/Liaoning.CHN/48.10/1	A	A	G	A	T	T	T	A	A	C	A	A
	MuVi/Jiangsu.CHN/4.13/5	A	A	G	A	T	T	T	A	A	C	A	A
	MuVi/Liaoning.CHN/10.09	A	A	G	A	T	T	T	A	A	C	A	C
	MuVi/Liaoning.CHN/1.08/2	A	A	G	A	T	T	T	A	A	C	A	A
	MuVi/Liaoning.CHN/5.09/1	A	A	G	A	T	T	T	A	A	C	A	A
	MuVi/Heilongjiang.CHN/22.12	A	A	G	A	T	T	T	A	A	C	A	A
	MuVi/Heilongjiang.CHN/15.13	A	A	G	A	T	T	T	A	A	C	A	A
	MuVi/Sichuan.CHN/23.12/2	A	A	G	A	T	T	T	A	A	C	A	A
	MuVi/Hubei.CHN/44.12/1	A	A	G	A	T	T	T	A	A	C	A	A
	MuVi/Jiangsu.CHN/2.13/4	A	A	G	A	T	T	T	A	A	C	A	A

1: Lineage based on Figure 1
2: SNPs are noted in green.
3: SNP position corresponding to a lineage-defining node ir

Supplementary Table S2: Listing of SNPs featured in the 7

Lineage [1]	Strain name	1068	1074	1077	1080	1083	1088	1095	1097	1098	1108	1110	1117
2	MuVi/Shanxi.CHN/52.10/2	T	T	G	T	T	A	C	T	C	T	C	C
2	MuVi/Shannxi.CHN/26.09/4	T	T	A	T	T	G	C	T	C	T	C	C
2	MuVi/Shanxi.CHN/52.10/1	T	C	G	T	T	G	C	T	T	T	C	C
2	MuVi/Guangdong.CHN/21.09/1	T	C	G	T	T	G	C	T	C	T	C	C
2	MuVi/Liaoning.CHN/48.10/2	T	C	G	T	T	G	C	T	C	T	C	C
2	MuVi/Shandong.CHN/51.15/1	T	C	G	T	T	G	C	T	C	T	C	C
2	MuVi/Heilongjiang.CHN/49.15	T	C	G	T	T	G	C	T	C	T	C	C
2	MuVi/Anhui.CHN/10.11/1	T	C	G	T	G	G	C	T	C	T	C	C
2	MuVi/Shannxi.CHN/20.12	T	C	G	T	G	G	C	T	C	T	C	C
3	MuVi/Shanxi.CHN/52.10/3	T	T	G	T	C	G	C	T	C	T	C	C
3	MuVi/Jiangsu.CHN/3.13/1	T	T	G	T	T	G	C	T	C	T	C	A
3	MuVi/Shandong.CHN/4.05	T	T	G	T	T	G	C	T	C	T	C	C
3	MuVi/Shannxi.CHN/9.09/2	T	T	G	T	T	G	C	T	C	T	C	C
3	MuVi/Incheon.KOR/16.08/22	T	T	G	T	T	G	C	T	C	T	C	C
3	MuVi/Henan.CHN/48.06	T	T	G	T	T	G	C	T	C	T	C	C
3	MuVi/Jiangsu.CHN/9.12	T	T	G	T	T	G	C	T	C	T	C	C
3	MuVi/Jiangsu.CHN/4.13/3	T	T	G	T	T	G	T	T	C	T	C	C
4	MuVi/Shannxi.CHN/26.09/1	T	T	G	T	T	G	C	T	C	T	C	C
4	MuVi/Jiangsu.CHN/15.12	T	T	G	T	T	G	C	T	C	T	C	C
1	MuVi/Jilin.CHN/15.08/5	T	T	G	T	T	G	C	T	T	T	C	C
1	MuVi/Jilin.CHN/15.08/1	T	T	G	T	T	G	C	T	T	T	C	C
1	MuVi/Jilin.CHN/15.08/3	T	T	G	T	T	G	C	T	T	T	C	C
1	MuVi/Yunnan.CHN/47.10/2	T	T	G	T	T	G	C	T	T	T	C	C
1	MuVi/Neimeng.CHN/18.11	T	T	G	A	T	G	C	A	T	T	C	C
1	MuVi/Sichuan.CHN/23.12/1	T	T	G	T	T	G	C	T	T	T	C	C
1	MuVi/Jiangsu.CHN/3.13/2	T	T	G	T	T	G	C	T	C	T	T	C
1	MuVi/Beijing.CHN/25.06	T	T	G	T	T	G	C	T	C	T	C	C
1	MuVi/Zhejiang.CHN/26.05	T	T	G	T	T	G	C	T	C	T	C	C
1	MuVi/Shanghai.CHN/0.01	T	T	G	T	T	G	C	T	C	T	C	C
1	MuVi/Shandong.CHN/3.05	T	T	G	T	T	G	C	T	C	T	C	C
1	MuVi/Gansu.CHN/0.02	T	T	G	T	T	G	C	T	C	T	C	C
1	MuVi/Beijing.CHN/15.06	C	T	G	T	T	G	C	T	C	T	C	C
1	MuVi/Shandong.CHN/11.07	T	T	G	T	T	G	C	T	C	T	C	C
1	MuVi/Sichuan.CHN/24.15	T	T	G	T	T	G	C	T	C	C	C	C
1	MuVi/Heilongjiang.CHN/24.14	T	T	G	T	T	G	C	T	C	C	C	C
1	MuVi/Shandong.CHN/43.07	T	T	G	T	T	G	C	T	C	T	C	C
1	MuVi/Shandong.CHN/10.08	T	T	G	T	T	G	C	T	C	T	C	C
1	MuVi/Heilongjiang.CHN/26.12/2	T	T	G	T	T	G	C	T	C	T	C	C
1	MuVi/Liaoning.CHN/48.11	T	T	G	T	T	G	C	T	C	T	C	C
1	MuVi/Heilongjiang.CHN/23.13/1	T	T	G	T	T	G	C	T	C	T	C	C
1	MuVi/Liaoning.CHN/50.11	T	T	G	T	T	G	C	T	C	T	C	C
1	MuVs/Kaohsiung.TWN/06.08	T	T	G	T	T	G	C	T	C	T	C	C
	MuVi/Jiangsu.CHN/12.13/2	T	T	G	T	T	G	C	T	C	T	C	C
	MuVi/Shannxi.CHN/20.15/1	T	T	G	T	T	G	C	T	C	T	C	C
	MuVi/Beijing.CHN/10.07	T	T	G	T	T	G	C	T	C	T	C	C
	MuVi/Hunan.CHN/30.11/3	T	T	G	T	T	G	C	T	C	T	C	C
	MuVi/Liaoning.CHN/9.10/1	T	T	G	T	T	G	C	T	C	T	C	C
	MuVi/Jiangsu.CHN/7.12	T	T	G	T	T	G	C	T	C	T	C	C
	MuVi/Heilongjiang.CHN/14.13/1	T	T	G	T	T	G	C	T	C	T	C	C
	MuVi/Hunan.CHN/32.11	T	T	G	T	T	G	C	T	C	T	C	C
	MuVi/Hunan.CHN/30.11/1	T	T	G	T	T	G	C	T	C	T	C	C
	MuVi/Jiangsu.CHN/7.13/1	T	T	G	T	T	G	C	T	C	T	C	C
	MuVi/Hunan.CHN/30.11/2	T	T	G	T	C	G	C	T	C	T	C	C
	MuVi/Liaoning.CHN/5.09/2	T	T	G	T	T	G	C	T	C	T	C	C
	Mui/Zhejiang.CHN/11.06/1	T	T	G	T	T	G	C	T	C	T	C	C
	MuVi/Liaoning.CHN/13.12	T	T	G	T	T	G	C	T	C	T	C	C
	MuVi/Beijing.CHN/17.07/2	T	T	G	T	T	G	C	T	C	T	C	C
	MuVi/Liaoning.CHN/7.12	T	T	G	T	T	G	C	T	C	T	C	C
	MuVi/Beijing.CHN/21.11	T	T	G	T	T	G	C	T	C	T	C	C
	MuVi/Liaoning.CHN/11.12	T	T	G	T	T	G	C	T	C	T	C	C
	MuVi/Heilongjiang.CHN/26.12/1	T	T	G	T	T	G	C	T	C	T	C	C
	MuVi/Liaoning.CHN/1.08/1	T	T	G	T	T	G	C	T	C	T	C	C
	MuVi/Liaoning.CHN/48.10/1	T	T	G	T	T	G	C	T	C	T	C	C
	MuVi/Jiangsu.CHN/4.13/5	T	T	G	T	T	G	C	T	C	T	C	C
	MuVi/Liaoning.CHN/10.09	T	T	G	T	T	G	C	T	C	T	C	C
	MuVi/Liaoning.CHN/1.08/2	T	T	G	T	T	G	C	T	C	T	C	C
	MuVi/Liaoning.CHN/5.09/1	T	T	G	T	T	G	C	T	C	T	C	C
	MuVi/Heilongjiang.CHN/22.12	T	T	G	T	T	G	C	T	C	T	C	C
	MuVi/Heilongjiang.CHN/15.13	T	T	G	T	T	G	C	T	C	T	C	C
	MuVi/Sichuan.CHN/23.12/2	T	T	G	T	T	G	C	T	C	T	C	C
	MuVi/Hubei.CHN/44.12/1	T	T	G	T	T	G	C	T	C	T	C	C
	MuVi/Jiangsu.CHN/2.13/4	T	T	G	T	T	G	C	T	C	T	C	C

1: Lineage based on Figure 1
2: SNPs are noted in green.
3: SNP position corresponding to a lineage-defining node ir

Supplementary Table S2: Listing of SNPs featured in the 7

Lineage [1]	Strain name	1121	1131	1137	1141	1149	1158	1161	1162	1164	1167	1173	1176
2	MuVi/Shanxi.CHN/52.10/2	G	T	T	G	A	G	C	C	A	T	T	C
2	MuVi/Shannxi.CHN/26.09/4	G	T	T	G	C	G	C	C	A	T	T	C
2	MuVi/Shanxi.CHN/52.10/1	G	T	T	G	C	G	C	C	A	T	T	C
2	MuVi/Guangdong.CHN/21.09/1	G	T	T	G	C	G	C	C	A	T	T	C
2	MuVi/Liaoning.CHN/48.10/2	G	T	T	G	C	G	C	C	A	T	T	T
2	MuVi/Shandong.CHN/51.15/1	G	T	T	A	C	G	C	C	A	T	T	T
2	MuVi/Heilongjiang.CHN/49.15	G	T	T	G	C	G	C	C	A	T	T	T
2	MuVi/Anhui.CHN/10.11/1	G	T	T	G	C	G	C	C	A	T	T	T
2	MuVi/Shannxi.CHN/20.12	G	T	T	G	C	G	C	C	A	T	T	T
3	MuVi/Shanxi.CHN/52.10/3	A	T	T	G	C	G	T	C	A	T	T	C
3	MuVi/Jiangsu.CHN/3.13/1	G	T	T	G	C	G	T	C	A	T	T	C
3	MuVi/Shandong.CHN/4.05	G	T	C	G	T	G	T	C	A	T	T	C
3	MuVi/Shannxi.CHN/9.09/2	G	T	C	G	T	G	T	C	A	T	T	C
3	MuVi/Incheon.KOR/16.08/22	G	T	C	G	T	G	T	C	A	T	T	C
3	MuVi/Henan.CHN/48.06	G	T	T	G	C	G	T	C	A	T	T	C
3	MuVi/Jiangsu.CHN/9.12	G	T	T	G	C	G	T	C	A	T	T	C
3	MuVi/Jiangsu.CHN/4.13/3	G	T	T	G	C	G	T	C	A	T	T	C
4	MuVi/Shannxi.CHN/26.09/1	G	T	T	G	C	G	C	C	A	T	T	C
4	MuVi/Jiangsu.CHN/15.12	G	T	T	G	C	G	C	C	A	T	T	C
1	MuVi/Jilin.CHN/15.08/5	G	T	T	G	C	G	C	C	A	T	T	C
1	MuVi/Jilin.CHN/15.08/1	G	T	T	G	C	G	C	C	A	T	T	C
1	MuVi/Jilin.CHN/15.08/3	G	T	T	G	C	G	C	C	A	T	T	C
1	MuVi/Yunnan.CHN/47.10/2	G	T	T	G	C	A	C	C	A	T	T	C
1	MuVi/Neimeng.CHN/18.11	G	T	T	G	C	G	C	C	A	T	T	C
1	MuVi/Sichuan.CHN/23.12/1	G	T	T	G	C	G	C	C	A	A	T	C
1	MuVi/Jiangsu.CHN/3.13/2	G	T	T	G	C	G	C	C	A	T	T	C
1	MuVi/Beijing.CHN/25.06	G	T	T	G	C	G	C	C	A	T	T	C
1	MuVi/Zhejiang.CHN/26.05	G	T	T	G	C	G	C	C	A	T	T	C
1	MuVi/Shanghai.CHN/0.01	G	T	T	G	C	G	C	C	A	T	T	C
1	MuVi/Shandong.CHN/3.05	G	T	T	G	C	G	C	C	A	T	T	C
1	MuVi/Gansu.CHN/0.02	G	T	T	G	C	G	C	C	A	T	T	C
1	MuVi/Beijing.CHN/15.06	G	T	T	G	C	G	C	C	A	T	T	C
1	MuVi/Shandong.CHN/11.07	G	T	T	G	C	G	C	C	A	T	T	C
1	MuVi/Sichuan.CHN/24.15	G	T	T	G	C	G	C	C	A	T	T	C
1	MuVi/Heilongjiang.CHN/24.14	G	T	T	G	C	G	C	C	A	T	T	C
1	MuVi/Shandong.CHN/43.07	G	T	T	G	C	G	C	C	A	T	T	C
1	MuVi/Shandong.CHN/10.08	G	T	T	G	C	G	C	C	A	T	T	C
1	MuVi/Heilongjiang.CHN/26.12/2	G	T	T	G	C	G	C	C	A	T	T	C
1	MuVi/Liaoning.CHN/48.11	G	T	T	G	C	G	C	C	A	T	T	C
1	MuVi/Heilongjiang.CHN/23.13/1	G	T	T	G	C	G	C	C	A	T	T	C
1	MuVi/Liaoning.CHN/50.11	G	T	T	G	C	G	C	C	A	T	T	C
1	MuVs/Kaohsiung.TWN/06.08	G	T	T	G	C	G	C	C	A	T	T	C
	MuVi/Jiangsu.CHN/12.13/2	G	C	T	G	C	G	T	C	A	T	T	C
	MuVi/Shannxi.CHN/20.15/1	G	C	T	G	C	G	C	C	A	T	T	C
	MuVi/Beijing.CHN/10.07	G	T	T	G	C	G	C	T	A	T	T	C
	MuVi/Hunan.CHN/30.11/3	G	T	T	G	C	G	C	C	A	T	T	C
	MuVi/Liaoning.CHN/9.10/1	G	T	T	G	C	G	C	C	A	T	T	C
	MuVi/Jiangsu.CHN/7.12	G	T	T	G	C	G	C	C	A	T	T	C
	MuVi/Heilongjiang.CHN/14.13/1	G	T	T	G	C	G	C	C	A	T	T	C
	MuVi/Hunan.CHN/32.11	G	T	C	G	C	G	C	C	A	T	T	C
	MuVi/Hunan.CHN/30.11/1	G	T	T	G	C	G	C	C	G	T	T	C
	MuVi/Jiangsu.CHN/7.13/1	G	T	T	G	C	G	C	C	G	T	T	C
	MuVi/Hunan.CHN/30.11/2	G	T	T	G	C	G	C	C	A	T	T	C
	MuVi/Liaoning.CHN/5.09/2	G	T	T	G	C	G	C	C	A	T	T	C
	Mui/Zhejiang.CHN/11.06/1	G	T	T	G	C	G	C	C	A	T	T	C
	MuVi/Liaoning.CHN/13.12	G	T	T	G	C	G	C	C	A	T	T	C
	MuVi/Beijing.CHN/17.07/2	G	T	T	G	C	G	C	C	A	T	C	C
	MuVi/Liaoning.CHN/7.12	G	T	T	G	C	G	C	C	A	T	T	C
	MuVi/Beijing.CHN/21.11	G	T	T	G	C	G	C	C	A	T	T	C
	MuVi/Liaoning.CHN/11.12	G	T	T	G	C	G	C	C	A	T	T	C
	MuVi/Heilongjiang.CHN/26.12/1	G	T	T	G	C	G	C	C	A	T	T	C
	MuVi/Liaoning.CHN/1.08/1	G	T	T	G	C	G	C	C	A	T	T	C
	MuVi/Liaoning.CHN/48.10/1	G	T	T	G	C	G	C	C	A	T	T	C
	MuVi/Jiangsu.CHN/4.13/5	G	T	T	G	C	G	C	C	A	T	T	T
	MuVi/Liaoning.CHN/10.09	G	T	T	G	C	G	C	C	A	T	T	C
	MuVi/Liaoning.CHN/1.08/2	G	T	T	G	C	G	C	C	A	T	T	C
	MuVi/Liaoning.CHN/5.09/1	G	T	T	G	C	G	C	C	A	T	T	C
	MuVi/Heilongjiang.CHN/22.12	G	T	T	G	C	G	C	C	A	T	T	C
	MuVi/Heilongjiang.CHN/15.13	G	T	T	G	C	G	C	C	A	T	T	C
	MuVi/Sichuan.CHN/23.12/2	G	T	T	G	C	G	C	C	A	T	T	C
	MuVi/Hubei.CHN/44.12/1	G	T	T	G	C	G	C	C	A	T	T	C
	MuVi/Jiangsu.CHN/2.13/4	G	T	T	G	C	G	C	C	A	T	T	C

1: Lineage based on Figure 1
2: SNPs are noted in green.
3: SNP position corresponding to a lineage-defining node ir

Supplementary Table S2: Listing of SNPs featured in the 7:

Lineage [1]	Strain name	1185	1188	1191	1194	1196	1197	1203	1209	1216	1221	1224	1232
2	MuVi/Shanxi.CHN/52.10/2	T	C	C	A	A	C	G	G	G	A	A	T
2	MuVi/Shannxi.CHN/26.09/4	T	T	C	A	A	C	G	G	G	A	A	T
2	MuVi/Shanxi.CHN/52.10/1	T	C	C	A	A	C	G	G	G	A	G	T
2	MuVi/Guangdong.CHN/21.09/1	T	C	C	A	A	C	G	G	G	A	G	T
2	MuVi/Liaoning.CHN/48.10/2	T	C	C	A	A	C	G	G	G	A	G	T
2	MuVi/Shandong.CHN/51.15/1	T	C	C	A	A	C	G	G	G	A	G	T
2	MuVi/Heilongjiang.CHN/49.15	T	C	C	A	A	C	G	G	G	A	G	T
2	MuVi/Anhui.CHN/10.11/1	T	C	C	A	A	C	G	G	G	A	G	T
2	MuVi/Shannxi.CHN/20.12	T	C	C	A	A	C	G	G	G	A	G	T
3	MuVi/Shanxi.CHN/52.10/3	T	C	C	A	A	C	A	G	G	G	A	T
3	MuVi/Jiangsu.CHN/3.13/1	T	C	C	A	A	C	A	G	G	G	A	T
3	MuVi/Shandong.CHN/4.05	T	C	C	A	A	C	A	G	G	A	A	T
3	MuVi/Shannxi.CHN/9.09/2	T	C	C	A	A	C	A	G	G	A	A	T
3	MuVi/Incheon.KOR/16.08/22	T	C	C	A	A	C	A	G	G	A	A	T
3	MuVi/Henan.CHN/48.06	T	C	C	A	A	C	A	G	G	A	A	T
3	MuVi/Jiangsu.CHN/9.12	T	C	C	A	A	C	A	G	G	A	A	T
3	MuVi/Jiangsu.CHN/4.13/3	T	C	C	A	A	C	A	G	G	A	A	T
4	MuVi/Shannxi.CHN/26.09/1	T	C	A	A	A	C	G	G	G	A	A	T
4	MuVi/Jiangsu.CHN/15.12	T	C	A	A	A	C	G	G	G	A	A	T
1	MuVi/Jilin.CHN/15.08/5	T	C	C	A	A	C	G	G	G	A	A	T
1	MuVi/Jilin.CHN/15.08/1	T	C	C	A	A	C	G	G	G	A	A	T
1	MuVi/Jilin.CHN/15.08/3	T	C	C	A	A	C	G	G	G	A	A	T
1	MuVi/Yunnan.CHN/47.10/2	T	C	C	A	A	C	G	G	G	A	G	T
1	MuVi/Neimeng.CHN/18.11	T	C	C	A	A	C	G	G	G	A	G	T
1	MuVi/Sichuan.CHN/23.12/1	T	C	C	A	A	C	G	T	G	A	A	T
1	MuVi/Jiangsu.CHN/3.13/2	T	C	C	A	A	C	G	G	G	A	A	T
1	MuVi/Beijing.CHN/25.06	T	C	C	A	A	C	G	G	G	A	A	T
1	MuVi/Zhejiang.CHN/26.05	T	C	C	A	A	C	G	G	G	A	A	T
1	MuVi/Shanghai.CHN/0.01	T	C	C	A	A	C	G	G	G	A	A	T
1	MuVi/Shandong.CHN/3.05	T	C	C	A	A	C	G	G	G	A	A	T
1	MuVi/Gansu.CHN/0.02	A	C	C	G	A	C	G	G	G	A	A	T
1	MuVi/Beijing.CHN/15.06	T	C	C	G	A	C	G	G	G	A	A	T
1	MuVi/Shandong.CHN/11.07	T	C	C	G	A	C	G	G	G	A	A	T
1	MuVi/Sichuan.CHN/24.15	T	C	C	G	A	C	G	G	G	A	A	T
1	MuVi/Heilongjiang.CHN/24.14	T	C	C	G	A	C	G	G	G	A	A	T
1	MuVi/Shandong.CHN/43.07	T	C	C	G	G	C	G	G	G	A	A	T
1	MuVi/Shandong.CHN/10.08	T	C	C	G	G	C	G	G	G	A	A	T
1	MuVi/Heilongjiang.CHN/26.12/2	T	C	C	G	A	C	G	G	G	A	A	T
1	MuVi/Liaoning.CHN/48.11	T	C	C	G	A	C	G	G	G	A	A	T
1	MuVi/Heilongjiang.CHN/23.13/1	T	C	C	G	A	C	G	G	G	A	A	T
1	MuVi/Liaoning.CHN/50.11	T	C	C	G	A	C	G	G	G	A	A	T
1	MuVs/Kaohsiung.TWN/06.08	T	C	C	G	A	C	G	G	G	A	A	T
	MuVi/Jiangsu.CHN/12.13/2	T	C	C	A	A	C	G	G	G	A	A	T
	MuVi/Shannxi.CHN/20.15/1	T	C	C	A	A	C	G	G	G	A	A	T
	MuVi/Beijing.CHN/10.07	T	C	C	A	A	C	G	G	G	A	A	T
	MuVi/Hunan.CHN/30.11/3	T	C	C	A	A	C	G	G	G	A	A	T
	MuVi/Liaoning.CHN/9.10/1	T	C	C	A	A	C	G	G	G	A	A	T
	MuVi/Jiangsu.CHN/7.12	T	C	C	A	A	C	G	G	G	A	A	T
	MuVi/Heilongjiang.CHN/14.13/1	T	C	C	A	A	C	G	G	G	A	A	T
	MuVi/Hunan.CHN/32.11	T	C	C	A	A	C	G	G	G	A	A	T
	MuVi/Hunan.CHN/30.11/1	T	C	C	A	A	C	G	G	G	A	A	T
	MuVi/Jiangsu.CHN/7.13/1	T	C	C	A	A	C	G	G	G	A	A	T
	MuVi/Hunan.CHN/30.11/2	T	C	C	A	A	C	G	G	G	A	A	A
	MuVi/Liaoning.CHN/5.09/2	T	C	C	A	A	C	G	G	G	A	A	T
	Mui/Zhejiang.CHN/11.06/1	T	C	C	A	A	T	G	G	G	A	A	T
	MuVi/Liaoning.CHN/13.12	T	C	C	A	A	C	G	G	G	A	A	T
	MuVi/Beijing.CHN/17.07/2	T	C	C	A	A	C	G	G	G	A	A	T
	MuVi/Liaoning.CHN/7.12	T	C	C	A	A	C	G	G	G	A	A	T
	MuVi/Beijing.CHN/21.11	T	C	C	A	A	C	G	G	T	A	A	T
	MuVi/Liaoning.CHN/11.12	T	C	C	A	A	C	G	G	T	A	A	T
	MuVi/Heilongjiang.CHN/26.12/1	T	C	C	A	A	C	G	G	T	A	A	T
	MuVi/Liaoning.CHN/1.08/1	T	C	C	A	A	C	G	G	T	A	A	T
	MuVi/Liaoning.CHN/48.10/1	T	C	C	A	A	C	G	G	T	A	A	T
	MuVi/Jiangsu.CHN/4.13/5	T	C	C	A	A	C	G	G	G	A	A	T
	MuVi/Liaoning.CHN/10.09	T	C	C	A	A	C	G	G	G	A	A	T
	MuVi/Liaoning.CHN/1.08/2	T	C	C	A	A	C	G	G	G	A	A	T
	MuVi/Liaoning.CHN/5.09/1	T	C	C	A	A	C	G	G	G	A	A	T
	MuVi/Heilongjiang.CHN/22.12	T	C	C	A	A	C	G	G	G	A	A	T
	MuVi/Heilongjiang.CHN/15.13	T	C	C	A	A	C	G	G	G	A	A	T
	MuVi/Sichuan.CHN/23.12/2	T	C	C	A	A	C	G	G	G	A	A	T
	MuVi/Hubei.CHN/44.12/1	T	C	C	A	A	C	G	G	G	A	A	T
	MuVi/Jiangsu.CHN/2.13/4	T	C	C	A	A	C	G	G	G	A	A	T

1: Lineage based on Figure 1
2: SNPs are noted in green.
3: SNP position corresponding to a lineage-defining node ir

Supplementary Table S2: Listing of SNPs featured in the 7:

Lineage [1]	Strain name	1233	1235	1236	1245	1263	1266	1272	1284	1285	1287	1293	1296
2	MuVi/Shanxi.CHN/52.10/2	A	C	G	T	G	A	C	G	T	T	C	C
2	MuVi/Shannxi.CHN/26.09/4	A	T	A	T	G	A	C	G	T	T	C	C
2	MuVi/Shanxi.CHN/52.10/1	A	T	G	T	G	A	C	G	T	T	C	T
2	MuVi/Guangdong.CHN/21.09/1	A	T	G	T	G	A	C	G	T	T	C	C
2	MuVi/Liaoning.CHN/48.10/2	A	T	G	T	G	A	C	G	T	C	C	C
2	MuVi/Shandong.CHN/51.15/1	A	T	G	T	G	A	C	G	T	T	C	C
2	MuVi/Heilongjiang.CHN/49.15	A	T	G	T	G	A	C	G	T	T	C	C
2	MuVi/Anhui.CHN/10.11/1	A	T	G	T	G	A	C	G	T	T	C	C
2	MuVi/Shannxi.CHN/20.12	A	T	G	T	G	A	C	G	T	T	C	C
3	MuVi/Shanxi.CHN/52.10/3	A	T	G	T	G	A	C	G	T	T	C	C
3	MuVi/Jiangsu.CHN/3.13/1	A	T	G	T	G	A	C	G	T	T	C	C
3	MuVi/Shandong.CHN/4.05	A	T	G	T	G	A	C	G	T	T	C	C
3	MuVi/Shannxi.CHN/9.09/2	A	T	G	T	G	A	C	G	T	T	C	C
3	MuVi/Incheon.KOR/16.08/22	A	T	G	T	G	A	C	G	T	T	C	C
3	MuVi/Henan.CHN/48.06	A	T	G	T	G	A	C	G	T	T	C	C
3	MuVi/Jiangsu.CHN/9.12	A	T	G	T	G	A	C	G	T	T	C	C
3	MuVi/Jiangsu.CHN/4.13/3	A	T	G	T	G	A	C	G	T	T	C	C
4	MuVi/Shannxi.CHN/26.09/1	A	T	G	T	G	A	C	G	T	T	C	C
4	MuVi/Jiangsu.CHN/15.12	A	T	G	T	G	A	C	G	T	T	C	C
1	MuVi/Jilin.CHN/15.08/5	A	T	G	T	G	A	C	G	T	T	C	C
1	MuVi/Jilin.CHN/15.08/1	A	T	G	T	G	A	C	G	T	T	C	C
1	MuVi/Jilin.CHN/15.08/3	A	T	G	T	G	A	C	G	T	T	C	C
1	MuVi/Yunnan.CHN/47.10/2	A	T	G	T	G	A	C	G	T	T	C	C
1	MuVi/Neimeng.CHN/18.11	A	T	G	T	G	A	C	G	T	T	C	C
1	MuVi/Sichuan.CHN/23.12/1	A	T	G	T	G	A	C	G	T	T	C	C
1	MuVi/Jiangsu.CHN/3.13/2	A	T	G	T	G	A	C	G	T	T	C	C
1	MuVi/Beijing.CHN/25.06	A	T	G	T	G	A	C	G	T	T	C	C
1	MuVi/Zhejiang.CHN/26.05	A	T	G	T	G	A	C	G	T	T	C	C
1	MuVi/Shanghai.CHN/0.01	A	T	G	T	G	A	C	G	T	T	C	C
1	MuVi/Shandong.CHN/3.05	A	T	G	T	G	A	C	G	T	T	C	C
1	MuVi/Gansu.CHN/0.02	A	T	G	T	G	A	C	G	T	T	C	C
1	MuVi/Beijing.CHN/15.06	A	T	G	C	G	A	C	G	T	T	C	C
1	MuVi/Shandong.CHN/11.07	A	T	G	T	G	A	T	G	T	T	C	C
1	MuVi/Sichuan.CHN/24.15	A	T	G	T	G	A	C	G	C	T	C	C
1	MuVi/Heilongjiang.CHN/24.14	A	T	G	T	G	A	C	G	T	T	C	C
1	MuVi/Shandong.CHN/43.07	A	T	G	T	G	A	C	G	T	T	C	C
1	MuVi/Shandong.CHN/10.08	A	T	G	T	G	A	C	G	T	T	C	C
1	MuVi/Heilongjiang.CHN/26.12/2	A	T	G	T	G	A	C	G	T	T	C	C
1	MuVi/Liaoning.CHN/48.11	A	T	G	T	G	A	C	G	T	T	C	C
1	MuVi/Heilongjiang.CHN/23.13/1	A	T	G	T	G	A	C	G	T	T	C	C
1	MuVi/Liaoning.CHN/50.11	A	T	G	T	G	A	C	G	T	T	C	C
1	MuVs/Kaohsiung.TWN/06.08	A	T	G	T	G	A	C	G	T	T	C	C
	MuVi/Jiangsu.CHN/12.13/2	A	T	G	T	A	A	C	G	T	T	C	C
	MuVi/Shannxi.CHN/20.15/1	A	T	G	T	A	A	C	A	T	T	C	C
	MuVi/Beijing.CHN/10.07	A	T	G	T	G	A	C	G	T	T	C	C
	MuVi/Hunan.CHN/30.11/3	A	T	G	T	G	A	C	G	T	T	T	C
	MuVi/Liaoning.CHN/9.10/1	A	T	G	T	G	A	C	G	T	T	T	C
	MuVi/Jiangsu.CHN/7.12	A	T	G	T	G	A	C	G	T	T	T	C
	MuVi/Heilongjiang.CHN/14.13/1	A	T	G	T	G	A	C	G	T	T	T	C
	MuVi/Hunan.CHN/32.11	A	T	G	T	G	A	C	G	T	T	C	C
	MuVi/Hunan.CHN/30.11/1	A	T	G	T	G	A	C	G	T	T	C	C
	MuVi/Jiangsu.CHN/7.13/1	A	T	G	T	G	A	C	G	T	T	C	C
	MuVi/Hunan.CHN/30.11/2	T	T	G	T	G	A	C	G	T	T	C	C
	MuVi/Liaoning.CHN/5.09/2	A	T	G	T	G	A	C	G	T	T	C	C
	Mui/Zhejiang.CHN/11.06/1	A	T	G	T	G	A	C	G	T	T	C	C
	MuVi/Liaoning.CHN/13.12	A	T	G	T	G	A	C	G	T	T	C	C
	MuVi/Beijing.CHN/17.07/2	A	T	G	T	G	A	C	G	T	T	C	C
	MuVi/Liaoning.CHN/7.12	A	T	G	T	G	A	C	G	T	T	C	C
	MuVi/Beijing.CHN/21.11	A	T	G	T	G	G	C	G	T	T	C	C
	MuVi/Liaoning.CHN/11.12	A	T	G	T	G	G	C	G	T	T	C	C
	MuVi/Heilongjiang.CHN/26.12/1	A	T	G	T	G	G	C	G	T	T	C	C
	MuVi/Liaoning.CHN/1.08/1	A	T	G	T	G	G	C	G	T	T	C	C
	MuVi/Liaoning.CHN/48.10/1	A	T	G	T	G	G	C	G	T	T	C	C
	MuVi/Jiangsu.CHN/4.13/5	A	T	G	T	G	G	C	G	T	T	C	C
	MuVi/Liaoning.CHN/10.09	A	T	G	T	G	G	C	G	T	T	C	C
	MuVi/Liaoning.CHN/1.08/2	A	T	G	T	G	G	C	G	T	T	C	C
	MuVi/Liaoning.CHN/5.09/1	A	T	G	T	G	G	C	G	T	T	C	C
	MuVi/Heilongjiang.CHN/22.12	A	T	G	T	G	A	C	G	T	T	C	C
	MuVi/Heilongjiang.CHN/15.13	A	T	G	T	G	A	C	G	T	T	C	C
	MuVi/Sichuan.CHN/23.12/2	A	T	G	T	G	A	C	G	T	T	C	C
	MuVi/Hubei.CHN/44.12/1	A	T	G	T	G	A	C	G	T	T	C	C
	MuVi/Jiangsu.CHN/2.13/4	A	T	G	T	G	A	C	G	T	T	C	C

1: Lineage based on Figure 1
2: SNPs are noted in green.
3: SNP position corresponding to a lineage-defining node ir

Supplementary Table S2: Listing of SNPs featured in the 7:

Lineage [1]	Strain name	1302	1305	1311	1313	1318	1320	1323	1324	1326	1335	1341	1344
2	MuVi/Shanxi.CHN/52.10/2	G	A	C	C	A	A	C	T	C	A	G	C
2	MuVi/Shannxi.CHN/26.09/4	G	A	C	C	A	A	C	T	C	A	G	C
2	MuVi/Shanxi.CHN/52.10/1	G	A	C	C	A	A	C	T	C	A	G	C
2	MuVi/Guangdong.CHN/21.09/1	G	A	C	C	A	A	C	T	C	A	G	T
2	MuVi/Liaoning.CHN/48.10/2	A	A	C	C	A	A	C	T	C	A	G	C
2	MuVi/Shandong.CHN/51.15/1	A	A	C	C	A	A	C	T	C	A	G	C
2	MuVi/Heilongjiang.CHN/49.15	A	A	C	C	A	A	C	T	C	A	G	C
2	MuVi/Anhui.CHN/10.11/1	A	A	C	C	A	A	C	T	C	A	G	C
2	MuVi/Shannxi.CHN/20.12	A	A	C	C	A	A	C	T	C	A	G	C
3	MuVi/Shanxi.CHN/52.10/3	G	A	C	C	A	A	C	T	T	G	G	T
3	MuVi/Jiangsu.CHN/3.13/1	G	A	C	C	A	A	C	T	T	G	G	T
3	MuVi/Shandong.CHN/4.05	G	A	C	C	A	A	C	T	T	G	G	T
3	MuVi/Shannxi.CHN/9.09/2	G	A	C	C	A	A	C	T	T	G	G	T
3	MuVi/Incheon.KOR/16.08/22	G	A	C	C	A	A	C	T	T	G	G	T
3	MuVi/Henan.CHN/48.06	G	A	C	C	A	A	C	T	T	G	G	T
3	MuVi/Jiangsu.CHN/9.12	G	A	C	C	A	A	C	T	T	G	G	T
3	MuVi/Jiangsu.CHN/4.13/3	G	A	C	C	A	A	C	T	T	G	G	T
4	MuVi/Shannxi.CHN/26.09/1	G	A	C	C	A	A	C	T	T	A	G	C
4	MuVi/Jiangsu.CHN/15.12	G	A	C	C	A	A	C	T	T	A	G	C
1	MuVi/Jilin.CHN/15.08/5	G	A	C	C	A	A	T	T	T	A	T	C
1	MuVi/Jilin.CHN/15.08/1	G	A	C	C	A	A	T	T	T	A	T	C
1	MuVi/Jilin.CHN/15.08/3	G	A	C	C	A	A	T	T	T	A	T	C
1	MuVi/Yunnan.CHN/47.10/2	G	A	C	C	A	A	T	T	T	A	T	C
1	MuVi/Neimeng.CHN/18.11	G	A	C	C	A	A	T	T	T	A	T	C
1	MuVi/Sichuan.CHN/23.12/1	G	A	C	C	A	A	C	T	T	A	T	C
1	MuVi/Jiangsu.CHN/3.13/2	G	A	C	C	A	A	C	T	T	A	G	C
1	MuVi/Beijing.CHN/25.06	G	A	C	C	A	A	C	T	T	A	G	C
1	MuVi/Zhejiang.CHN/26.05	G	A	C	C	A	A	C	T	T	A	G	C
1	MuVi/Shanghai.CHN/0.01	G	A	C	C	A	A	C	T	T	A	G	C
1	MuVi/Shandong.CHN/3.05	G	A	C	C	A	A	C	T	T	A	G	C
1	MuVi/Gansu.CHN/0.02	G	A	C	C	A	A	C	T	T	A	G	C
1	MuVi/Beijing.CHN/15.06	G	A	C	A	C	A	C	T	T	A	G	C
1	MuVi/Shandong.CHN/11.07	G	A	C	C	A	A	C	T	T	A	G	C
1	MuVi/Sichuan.CHN/24.15	G	A	C	C	A	A	C	T	T	A	G	C
1	MuVi/Heilongjiang.CHN/24.14	G	A	C	C	A	A	C	T	T	A	G	C
1	MuVi/Shandong.CHN/43.07	G	A	C	C	A	A	C	T	T	A	G	C
1	MuVi/Shandong.CHN/10.08	G	A	C	C	A	A	C	T	T	A	G	C
1	MuVi/Heilongjiang.CHN/26.12/2	G	A	C	C	A	A	C	T	T	A	G	C
1	MuVi/Liaoning.CHN/48.11	G	A	C	C	A	A	C	T	T	A	G	C
1	MuVi/Heilongjiang.CHN/23.13/1	G	A	C	C	A	A	C	T	T	A	G	C
1	MuVi/Liaoning.CHN/50.11	G	A	C	C	A	A	C	T	T	A	G	C
1	MuVs/Kaohsiung.TWN/06.08	G	A	C	C	A	A	C	T	T	A	G	C
	MuVi/Jiangsu.CHN/12.13/2	G	A	C	C	A	A	C	T	T	A	G	C
	MuVi/Shannxi.CHN/20.15/1	G	A	C	C	A	A	C	T	T	A	G	C
	MuVi/Beijing.CHN/10.07	G	A	C	C	A	A	C	T	T	A	G	C
	MuVi/Hunan.CHN/30.11/3	G	A	T	C	A	A	C	T	T	A	G	C
	MuVi/Liaoning.CHN/9.10/1	G	A	T	C	A	A	C	T	T	A	G	C
	MuVi/Jiangsu.CHN/7.12	G	A	T	C	A	A	C	T	T	A	G	C
	MuVi/Heilongjiang.CHN/14.13/1	G	A	T	C	A	G	C	T	T	A	G	C
	MuVi/Hunan.CHN/32.11	G	A	C	C	A	A	C	T	T	A	G	C
	MuVi/Hunan.CHN/30.11/1	G	A	C	C	A	A	C	T	T	A	G	C
	MuVi/Jiangsu.CHN/7.13/1	G	A	C	C	A	A	C	T	T	A	G	C
	MuVi/Hunan.CHN/30.11/2	G	A	C	C	A	A	C	T	T	A	G	C
	MuVi/Liaoning.CHN/5.09/2	G	A	C	C	A	A	C	T	T	A	G	C
	Mui/Zhejiang.CHN/11.06/1	G	A	C	C	A	A	C	T	T	A	G	C
	MuVi/Liaoning.CHN/13.12	G	A	C	C	A	A	C	T	T	A	G	T
	MuVi/Beijing.CHN/17.07/2	G	A	C	C	A	A	C	T	T	A	G	T
	MuVi/Liaoning.CHN/7.12	G	A	C	C	A	A	C	T	T	A	G	C
	MuVi/Beijing.CHN/21.11	G	A	C	C	A	A	C	T	T	A	G	C
	MuVi/Liaoning.CHN/11.12	G	A	C	C	A	A	C	T	T	A	G	C
	MuVi/Heilongjiang.CHN/26.12/1	G	A	C	C	A	A	C	T	T	A	G	C
	MuVi/Liaoning.CHN/1.08/1	G	A	C	C	A	A	C	T	T	A	G	C
	MuVi/Liaoning.CHN/48.10/1	G	A	C	C	A	A	C	T	T	A	G	C
	MuVi/Jiangsu.CHN/4.13/5	G	A	C	C	A	A	C	T	T	A	G	C
	MuVi/Liaoning.CHN/10.09	G	A	C	C	A	A	C	T	T	A	G	C
	MuVi/Liaoning.CHN/1.08/2	G	A	C	C	A	A	C	T	T	A	G	C
	MuVi/Liaoning.CHN/5.09/1	G	A	C	C	A	A	C	T	T	A	G	C
	MuVi/Heilongjiang.CHN/22.12	G	A	C	C	A	A	C	A	T	A	G	C
	MuVi/Heilongjiang.CHN/15.13	G	A	C	C	A	A	C	T	T	A	G	C
	MuVi/Sichuan.CHN/23.12/2	G	A	C	C	A	A	C	T	T	A	G	C
	MuVi/Hubei.CHN/44.12/1	G	G	C	C	A	A	C	T	T	A	G	C
	MuVi/Jiangsu.CHN/2.13/4	G	G	C	C	A	A	C	T	T	A	G	C

1: Lineage based on Figure 1
2: SNPs are noted in green.
3: SNP position corresponding to a lineage-defining node ir

Supplementary Table S2: Listing of SNPs featured in the 7[illegible]

Lineage [1]	Strain name	1350	1354	1359	1365	1371	1386	1390	1395	1398	1404	1410	1416
2	MuVi/Shanxi.CHN/52.10/2	C	A	T	C	C	A	A	C	T	A	G	G
2	MuVi/Shannxi.CHN/26.09/4	C	A	T	C	C	A	A	C	T	A	G	G
2	MuVi/Shanxi.CHN/52.10/1	C	A	T	C	C	A	A	C	T	A	G	G
2	MuVi/Guangdong.CHN/21.09/1	C	A	T	C	C	A	A	C	T	A	G	G
2	MuVi/Liaoning.CHN/48.10/2	C	A	T	C	C	A	A	C	T	A	G	G
2	MuVi/Shandong.CHN/51.15/1	C	A	T	C	C	A	A	C	C	A	G	G
2	MuVi/Heilongjiang.CHN/49.15	C	A	T	C	C	A	A	C	T	A	G	G
2	MuVi/Anhui.CHN/10.11/1	C	A	T	C	C	A	A	C	T	A	G	G
2	MuVi/Shannxi.CHN/20.12	C	A	T	C	C	A	A	C	T	A	G	G
3	MuVi/Shanxi.CHN/52.10/3	C	A	T	C	C	A	A	T	T	A	G	G
3	MuVi/Jiangsu.CHN/3.13/1	C	A	T	C	C	A	A	C	T	A	G	G
3	MuVi/Shandong.CHN/4.05	C	A	T	C	C	A	A	C	T	A	G	G
3	MuVi/Shannxi.CHN/9.09/2	C	A	T	C	C	A	A	C	T	A	G	G
3	MuVi/Incheon.KOR/16.08/22	C	A	T	C	C	A	A	C	T	A	G	G
3	MuVi/Henan.CHN/48.06	C	A	T	C	C	G	A	C	T	A	G	G
3	MuVi/Jiangsu.CHN/9.12	C	A	T	C	C	G	A	C	T	A	G	G
3	MuVi/Jiangsu.CHN/4.13/3	C	A	T	C	C	G	A	C	T	A	G	G
4	MuVi/Shannxi.CHN/26.09/1	C	A	T	C	C	A	A	C	T	A	G	G
4	MuVi/Jiangsu.CHN/15.12	C	A	T	C	C	A	A	C	T	A	G	G
1	MuVi/Jilin.CHN/15.08/5	C	A	T	C	C	A	A	C	T	A	G	G
1	MuVi/Jilin.CHN/15.08/1	C	A	T	C	C	A	A	C	T	A	G	G
1	MuVi/Jilin.CHN/15.08/3	C	A	T	C	C	A	A	C	T	A	G	G
1	MuVi/Yunnan.CHN/47.10/2	C	A	T	C	C	A	A	C	T	A	G	G
1	MuVi/Neimeng.CHN/18.11	C	A	T	C	C	A	A	C	T	A	G	G
1	MuVi/Sichuan.CHN/23.12/1	C	A	T	C	C	A	A	C	T	A	G	G
1	MuVi/Jiangsu.CHN/3.13/2	C	A	T	C	C	A	A	C	T	C	G	G
1	MuVi/Beijing.CHN/25.06	C	A	T	C	C	A	A	C	T	A	G	G
1	MuVi/Zhejiang.CHN/26.05	C	A	T	C	C	A	A	C	T	A	G	G
1	MuVi/Shanghai.CHN/0.01	C	A	T	C	C	A	A	C	T	A	G	G
1	MuVi/Shandong.CHN/3.05	C	A	T	C	C	A	A	C	T	A	G	G
1	MuVi/Gansu.CHN/0.02	C	A	T	C	C	A	C	C	T	A	G	A
1	MuVi/Beijing.CHN/15.06	C	A	T	C	C	A	A	C	T	A	G	A
1	MuVi/Shandong.CHN/11.07	C	A	T	C	C	A	A	C	T	A	G	A
1	MuVi/Sichuan.CHN/24.15	C	A	T	C	C	A	A	C	T	A	G	A
1	MuVi/Heilongjiang.CHN/24.14	C	A	T	C	C	A	A	C	T	A	G	A
1	MuVi/Shandong.CHN/43.07	C	A	T	C	C	A	A	C	T	A	G	A
1	MuVi/Shandong.CHN/10.08	C	A	T	C	C	A	A	C	T	A	G	A
1	MuVi/Heilongjiang.CHN/26.12/2	C	A	T	C	C	A	A	T	T	A	G	A
1	MuVi/Liaoning.CHN/48.11	C	A	T	C	C	A	A	C	T	A	G	A
1	MuVi/Heilongjiang.CHN/23.13/1	C	A	T	C	C	A	A	C	T	A	G	A
1	MuVi/Liaoning.CHN/50.11	C	A	T	C	C	A	A	C	T	A	G	A
1	MuVs/Kaohsiung.TWN/06.08	C	A	T	C	C	A	A	C	T	A	A	A
	MuVi/Jiangsu.CHN/12.13/2	C	A	T	C	C	A	A	C	T	A	G	G
	MuVi/Shannxi.CHN/20.15/1	C	A	T	C	C	A	A	C	T	A	G	G
	MuVi/Beijing.CHN/10.07	C	A	T	T	C	A	A	C	T	A	G	G
	MuVi/Hunan.CHN/30.11/3	C	A	T	C	C	A	A	C	T	A	G	G
	MuVi/Liaoning.CHN/9.10/1	C	A	T	C	C	A	A	C	T	A	G	G
	MuVi/Jiangsu.CHN/7.12	C	A	T	C	C	A	A	C	T	A	G	G
	MuVi/Heilongjiang.CHN/14.13/1	C	A	T	C	C	A	A	C	T	A	G	G
	MuVi/Hunan.CHN/32.11	C	A	T	C	C	A	A	C	T	A	G	G
	MuVi/Hunan.CHN/30.11/1	C	A	T	C	C	A	A	C	T	A	G	G
	MuVi/Jiangsu.CHN/7.13/1	C	A	T	C	T	A	A	C	T	A	G	G
	MuVi/Hunan.CHN/30.11/2	C	A	C	C	C	A	A	C	T	A	G	G
	MuVi/Liaoning.CHN/5.09/2	C	G	T	C	C	A	A	C	T	A	G	G
	Mui/Zhejiang.CHN/11.06/1	C	A	T	C	C	A	A	C	T	A	G	G
	MuVi/Liaoning.CHN/13.12	T	A	T	C	C	A	A	C	T	A	G	G
	MuVi/Beijing.CHN/17.07/2	C	A	T	C	C	A	A	C	T	G	G	G
	MuVi/Liaoning.CHN/7.12	C	A	T	C	C	A	A	C	T	A	G	G
	MuVi/Beijing.CHN/21.11	C	A	T	C	C	A	A	C	T	A	G	G
	MuVi/Liaoning.CHN/11.12	C	A	T	C	C	A	A	C	T	A	G	G
	MuVi/Heilongjiang.CHN/26.12/1	C	A	T	C	C	A	A	C	T	A	G	G
	MuVi/Liaoning.CHN/1.08/1	C	A	T	C	C	A	A	C	T	A	G	G
	MuVi/Liaoning.CHN/48.10/1	C	A	T	C	C	A	A	C	T	A	G	G
	MuVi/Jiangsu.CHN/4.13/5	C	A	T	C	C	A	A	C	T	A	G	G
	MuVi/Liaoning.CHN/10.09	C	A	T	C	C	A	A	C	T	A	G	G
	MuVi/Liaoning.CHN/1.08/2	C	A	T	C	C	A	A	C	T	A	G	G
	MuVi/Liaoning.CHN/5.09/1	C	A	T	C	C	A	A	C	T	A	G	G
	MuVi/Heilongjiang.CHN/22.12	C	A	T	C	C	A	A	C	T	A	G	G
	MuVi/Heilongjiang.CHN/15.13	C	A	T	C	C	A	A	C	T	A	G	G
	MuVi/Sichuan.CHN/23.12/2	C	A	T	C	C	A	A	C	T	A	G	G
	MuVi/Hubei.CHN/44.12/1	C	A	T	C	C	A	A	C	T	A	G	G
	MuVi/Jiangsu.CHN/2.13/4	C	A	T	C	C	A	A	C	T	A	G	G

1: Lineage based on Figure 1
2: SNPs are noted in green.
3: SNP position corresponding to a lineage-defining node ir[illegible]

Supplementary Table S2: Listing of SNPs featured in the 7:

Lineage [1]	Strain name	1418	1420	1421	1422	1425	1428	1449	1456	1458	1461	1467	1469
2	MuVi/Shanxi.CHN/52.10/2	C	G	C	T	T	G	T	T	A	T	T	G
2	MuVi/Shannxi.CHN/26.09/4	C	G	C	T	T	G	T	T	A	T	T	G
2	MuVi/Shanxi.CHN/52.10/1	C	G	C	T	T	G	C	T	A	T	T	G
2	MuVi/Guangdong.CHN/21.09/1	C	G	C	T	T	G	T	T	A	T	T	G
2	MuVi/Liaoning.CHN/48.10/2	C	G	C	T	T	G	T	T	A	T	T	G
2	MuVi/Shandong.CHN/51.15/1	C	G	C	T	T	G	T	T	A	T	T	G
2	MuVi/Heilongjiang.CHN/49.15	C	G	C	T	T	G	T	T	A	T	T	G
2	MuVi/Anhui.CHN/10.11/1	C	G	C	T	T	G	T	T	A	T	T	G
2	MuVi/Shannxi.CHN/20.12	C	G	C	T	T	G	T	T	A	T	T	G
3	MuVi/Shanxi.CHN/52.10/3	C	G	C	T	T	G	T	T	G	T	T	G
3	MuVi/Jiangsu.CHN/3.13/1	C	G	C	T	T	G	T	T	G	T	T	G
3	MuVi/Shandong.CHN/4.05	C	G	C	T	T	G	T	T	A	T	T	G
3	MuVi/Shannxi.CHN/9.09/2	C	G	C	T	T	G	T	T	A	T	T	G
3	MuVi/Incheon.KOR/16.08/22	C	G	C	T	T	G	T	T	A	T	T	G
3	MuVi/Henan.CHN/48.06	C	G	C	T	T	G	T	T	A	T	T	G
3	MuVi/Jiangsu.CHN/9.12	C	G	C	T	T	G	T	T	A	T	T	G
3	MuVi/Jiangsu.CHN/4.13/3	C	G	C	T	T	G	T	T	A	T	T	G
4	MuVi/Shannxi.CHN/26.09/1	C	G	T	T	T	G	T	T	A	T	T	G
4	MuVi/Jiangsu.CHN/15.12	C	G	T	T	T	G	T	T	A	T	T	G
1	MuVi/Jilin.CHN/15.08/5	C	G	C	T	C	G	T	T	A	T	T	G
1	MuVi/Jilin.CHN/15.08/1	C	G	C	T	C	G	T	T	A	T	T	G
1	MuVi/Jilin.CHN/15.08/3	C	G	C	T	C	G	T	T	A	T	T	G
1	MuVi/Yunnan.CHN/47.10/2	C	G	C	C	C	A	T	T	A	T	T	G
1	MuVi/Neimeng.CHN/18.11	C	G	C	T	C	G	T	T	A	T	T	G
1	MuVi/Sichuan.CHN/23.12/1	C	G	C	T	C	G	T	T	A	T	T	G
1	MuVi/Jiangsu.CHN/3.13/2	C	G	C	T	C	G	T	T	A	T	T	A
1	MuVi/Beijing.CHN/25.06	C	G	C	T	C	G	T	T	A	T	T	G
1	MuVi/Zhejiang.CHN/26.05	C	G	C	T	C	G	T	T	A	T	T	G
1	MuVi/Shanghai.CHN/0.01	C	A	C	T	C	G	T	T	A	T	T	G
1	MuVi/Shandong.CHN/3.05	C	A	C	T	C	G	T	T	A	T	T	G
1	MuVi/Gansu.CHN/0.02	C	G	C	T	C	G	T	T	A	T	T	G
1	MuVi/Beijing.CHN/15.06	C	G	C	T	C	G	T	T	A	T	T	A
1	MuVi/Shandong.CHN/11.07	C	G	C	T	C	G	T	T	A	T	T	G
1	MuVi/Sichuan.CHN/24.15	C	A	C	T	C	G	T	T	A	T	T	G
1	MuVi/Heilongjiang.CHN/24.14	C	G	C	T	C	G	T	T	A	T	T	G
1	MuVi/Shandong.CHN/43.07	C	G	C	T	C	G	T	T	A	T	T	G
1	MuVi/Shandong.CHN/10.08	C	G	C	T	C	G	T	T	A	T	T	G
1	MuVi/Heilongjiang.CHN/26.12/2	C	G	C	T	C	G	T	T	A	T	T	G
1	MuVi/Liaoning.CHN/48.11	C	G	C	T	C	G	T	T	A	T	T	G
1	MuVi/Heilongjiang.CHN/23.13/1	C	G	C	T	C	G	T	T	A	T	T	G
1	MuVi/Liaoning.CHN/50.11	C	G	C	T	C	G	T	T	A	T	T	G
1	MuVs/Kaohsiung.TWN/06.08	C	G	C	T	C	G	T	T	A	T	T	G
	MuVi/Jiangsu.CHN/12.13/2	C	G	C	T	T	G	T	T	A	T	T	G
	MuVi/Shannxi.CHN/20.15/1	C	G	C	T	T	G	T	T	A	T	T	G
	MuVi/Beijing.CHN/10.07	C	G	C	T	T	G	T	T	A	T	T	G
	MuVi/Hunan.CHN/30.11/3	C	G	C	T	T	G	T	C	A	T	T	G
	MuVi/Liaoning.CHN/9.10/1	C	G	C	T	T	G	T	C	A	T	T	G
	MuVi/Jiangsu.CHN/7.12	C	G	C	T	T	G	T	C	A	T	T	G
	MuVi/Heilongjiang.CHN/14.13/1	C	G	C	T	T	G	T	C	A	T	T	G
	MuVi/Hunan.CHN/32.11	C	G	C	T	T	G	T	T	A	T	C	G
	MuVi/Hunan.CHN/30.11/1	C	G	C	T	T	G	T	T	A	C	T	G
	MuVi/Jiangsu.CHN/7.13/1	A	G	C	T	T	G	T	T	A	C	T	G
	MuVi/Hunan.CHN/30.11/2	C	G	C	T	T	G	T	T	A	T	T	G
	MuVi/Liaoning.CHN/5.09/2	C	G	C	T	T	G	T	T	A	T	T	G
	Mui/Zhejiang.CHN/11.06/1	C	G	C	T	T	G	T	T	A	T	T	G
	MuVi/Liaoning.CHN/13.12	C	G	C	T	T	G	T	T	A	T	T	G
	MuVi/Beijing.CHN/17.07/2	C	G	C	T	T	G	T	T	A	T	T	G
	MuVi/Liaoning.CHN/7.12	C	G	C	T	T	G	T	T	A	T	T	G
	MuVi/Beijing.CHN/21.11	C	G	C	T	T	G	T	T	A	G	T	G
	MuVi/Liaoning.CHN/11.12	C	G	C	T	T	G	T	T	A	G	T	G
	MuVi/Heilongjiang.CHN/26.12/1	C	G	C	T	T	G	T	T	A	G	T	G
	MuVi/Liaoning.CHN/1.08/1	C	G	C	T	T	G	T	T	A	G	T	G
	MuVi/Liaoning.CHN/48.10/1	C	G	C	T	T	G	T	T	A	G	T	G
	MuVi/Jiangsu.CHN/4.13/5	C	G	C	T	T	G	T	T	A	T	T	G
	MuVi/Liaoning.CHN/10.09	C	G	C	T	T	G	T	T	A	T	T	G
	MuVi/Liaoning.CHN/1.08/2	C	G	C	T	T	G	T	T	A	T	T	G
	MuVi/Liaoning.CHN/5.09/1	C	G	C	T	T	G	T	T	A	T	T	G
	MuVi/Heilongjiang.CHN/22.12	C	G	C	T	T	G	T	T	A	T	T	G
	MuVi/Heilongjiang.CHN/15.13	C	G	C	T	T	G	T	T	A	T	T	G
	MuVi/Sichuan.CHN/23.12/2	C	G	C	T	T	G	T	T	A	T	T	G
	MuVi/Hubei.CHN/44.12/1	C	G	C	T	T	G	T	T	A	T	T	G
	MuVi/Jiangsu.CHN/2.13/4	C	G	C	T	T	G	T	T	A	T	T	G

1: Lineage based on Figure 1
2: SNPs are noted in green.
3: SNP position corresponding to a lineage-defining node ir

Supplementary Table S2: Listing of SNPs featured in the 7

Lineage [1]	Strain name	1476	1485	1488	1491	1500	1503	1509	1515	1516	1521	1526	1529
2	MuVi/Shanxi.CHN/52.10/2	A	T	C	A	T	C	T	A	T	T	G	C
2	MuVi/Shannxi.CHN/26.09/4	A	T	C	A	T	C	T	A	T	T	G	C
2	MuVi/Shanxi.CHN/52.10/1	A	T	C	A	T	C	T	A	T	T	G	C
2	MuVi/Guangdong.CHN/21.09/1	A	T	C	A	T	C	T	A	T	T	G	C
2	MuVi/Liaoning.CHN/48.10/2	A	T	C	A	T	C	T	A	T	T	G	C
2	MuVi/Shandong.CHN/51.15/1	A	T	C	A	T	C	T	A	T	T	A	T
2	MuVi/Heilongjiang.CHN/49.15	A	T	C	A	T	C	T	A	T	T	G	C
2	MuVi/Anhui.CHN/10.11/1	A	T	C	A	T	C	T	A	C	T	G	C
2	MuVi/Shannxi.CHN/20.12	A	T	C	A	T	C	T	A	T	T	G	C
3	MuVi/Shanxi.CHN/52.10/3	A	T	C	A	C	C	T	A	T	T	G	C
3	MuVi/Jiangsu.CHN/3.13/1	A	T	C	A	C	C	T	A	T	T	G	C
3	MuVi/Shandong.CHN/4.05	A	T	C	A	T	C	T	A	T	T	G	C
3	MuVi/Shannxi.CHN/9.09/2	A	T	C	A	T	C	T	A	T	T	G	C
3	MuVi/Incheon.KOR/16.08/22	A	T	C	A	T	C	T	A	T	T	G	C
3	MuVi/Henan.CHN/48.06	G	T	C	A	T	C	T	A	T	T	G	C
3	MuVi/Jiangsu.CHN/9.12	G	T	C	A	T	C	T	A	T	T	G	C
3	MuVi/Jiangsu.CHN/4.13/3	G	T	C	A	T	C	T	A	T	T	G	C
4	MuVi/Shannxi.CHN/26.09/1	A	T	C	A	T	C	T	A	T	T	G	C
4	MuVi/Jiangsu.CHN/15.12	A	T	C	A	T	C	T	A	T	T	G	C
1	MuVi/Jilin.CHN/15.08/5	A	T	C	A	T	C	T	A	T	T	G	C
1	MuVi/Jilin.CHN/15.08/1	A	T	C	A	T	C	T	A	T	T	G	C
1	MuVi/Jilin.CHN/15.08/3	A	T	C	A	T	C	T	A	T	T	G	C
1	MuVi/Yunnan.CHN/47.10/2	A	T	C	A	T	C	T	A	T	T	G	C
1	MuVi/Neimeng.CHN/18.11	A	T	C	A	T	C	T	G	T	T	G	C
1	MuVi/Sichuan.CHN/23.12/1	A	T	C	G	T	C	T	A	T	T	G	C
1	MuVi/Jiangsu.CHN/3.13/2	A	T	C	A	T	C	T	A	T	T	G	C
1	MuVi/Beijing.CHN/25.06	A	T	C	A	T	C	T	A	T	T	G	C
1	MuVi/Zhejiang.CHN/26.05	A	T	C	A	T	C	T	A	T	T	G	C
1	MuVi/Shanghai.CHN/0.01	A	T	C	A	T	C	T	A	T	T	G	C
1	MuVi/Shandong.CHN/3.05	A	T	C	A	T	C	T	A	T	T	G	C
1	MuVi/Gansu.CHN/0.02	A	T	C	A	T	C	T	A	T	T	G	C
1	MuVi/Beijing.CHN/15.06	A	T	C	A	T	C	T	A	T	T	G	C
1	MuVi/Shandong.CHN/11.07	A	T	C	A	T	C	T	A	T	T	G	C
1	MuVi/Sichuan.CHN/24.15	A	T	C	A	T	C	T	A	T	T	G	C
1	MuVi/Heilongjiang.CHN/24.14	A	T	C	A	T	C	T	A	T	T	G	C
1	MuVi/Shandong.CHN/43.07	A	T	C	A	T	C	T	A	T	T	G	C
1	MuVi/Shandong.CHN/10.08	A	T	C	A	T	C	T	A	T	T	G	C
1	MuVi/Heilongjiang.CHN/26.12/2	A	T	C	A	T	C	T	A	T	T	G	C
1	MuVi/Liaoning.CHN/48.11	A	T	C	A	T	C	T	A	T	T	G	C
1	MuVi/Heilongjiang.CHN/23.13/1	A	T	C	A	T	C	T	A	T	T	G	C
1	MuVi/Liaoning.CHN/50.11	A	T	C	A	T	C	T	A	T	T	G	C
1	MuVs/Kaohsiung.TWN/06.08	A	T	C	A	T	C	T	A	T	T	G	C
	MuVi/Jiangsu.CHN/12.13/2	A	T	C	A	T	C	T	A	T	T	G	C
	MuVi/Shannxi.CHN/20.15/1	A	T	C	A	T	T	T	A	T	T	G	C
	MuVi/Beijing.CHN/10.07	A	T	C	A	T	C	T	A	T	C	G	C
	MuVi/Hunan.CHN/30.11/3	A	T	C	A	T	C	T	A	T	T	G	C
	MuVi/Liaoning.CHN/9.10/1	A	T	C	A	T	C	T	A	T	T	G	C
	MuVi/Jiangsu.CHN/7.12	A	T	C	A	T	C	T	A	T	T	G	C
	MuVi/Heilongjiang.CHN/14.13/1	A	T	C	A	T	C	T	A	T	T	G	C
	MuVi/Hunan.CHN/32.11	A	C	C	A	T	C	T	A	T	T	G	C
	MuVi/Hunan.CHN/30.11/1	A	T	C	G	T	C	T	A	C	T	G	C
	MuVi/Jiangsu.CHN/7.13/1	A	T	C	G	T	C	T	A	T	T	G	C
	MuVi/Hunan.CHN/30.11/2	A	T	T	A	T	C	T	A	C	T	G	C
	MuVi/Liaoning.CHN/5.09/2	A	T	C	A	T	C	T	A	T	T	G	C
	Mui/Zhejiang.CHN/11.06/1	A	T	C	A	T	C	T	A	T	T	G	C
	MuVi/Liaoning.CHN/13.12	A	T	C	A	T	C	A	A	T	T	G	C
	MuVi/Beijing.CHN/17.07/2	A	T	C	A	T	C	A	A	T	T	G	C
	MuVi/Liaoning.CHN/7.12	A	T	C	A	T	T	T	A	T	T	G	C
	MuVi/Beijing.CHN/21.11	A	T	C	A	T	C	T	A	T	T	G	C
	MuVi/Liaoning.CHN/11.12	A	T	C	A	T	C	T	A	T	T	G	C
	MuVi/Heilongjiang.CHN/26.12/1	A	T	C	A	T	C	T	A	T	T	G	C
	MuVi/Liaoning.CHN/1.08/1	A	T	C	A	T	C	T	A	T	T	G	C
	MuVi/Liaoning.CHN/48.10/1	A	T	C	A	T	C	T	A	T	T	G	C
	MuVi/Jiangsu.CHN/4.13/5	A	T	C	A	T	C	T	A	T	T	G	C
	MuVi/Liaoning.CHN/10.09	A	T	C	A	T	C	T	A	T	T	G	C
	MuVi/Liaoning.CHN/1.08/2	A	T	C	A	T	C	T	A	T	T	G	C
	MuVi/Liaoning.CHN/5.09/1	A	T	C	A	T	C	T	A	T	T	G	C
	MuVi/Heilongjiang.CHN/22.12	A	T	C	A	T	C	T	A	T	T	G	C
	MuVi/Heilongjiang.CHN/15.13	A	T	C	A	T	C	T	A	T	T	G	C
	MuVi/Sichuan.CHN/23.12/2	A	T	C	A	T	C	T	A	T	T	G	C
	MuVi/Hubei.CHN/44.12/1	A	T	C	A	T	C	T	A	T	T	G	C
	MuVi/Jiangsu.CHN/2.13/4	A	T	C	A	T	C	T	A	T	T	G	C

1: Lineage based on Figure 1
2: SNPs are noted in green.
3: SNP position corresponding to a lineage-defining node ir

Supplementary Table S2: Listing of SNPs featured in the 7

Lineage [1]	Strain name	1533	1537	1545	1548	1549	1554	1557	1562	1566	1569	1572	1573
2	MuVi/Shanxi.CHN/52.10/2	C	G	T	C	C	T	C	G	T	T	T	C
2	MuVi/Shannxi.CHN/26.09/4	C	G	T	C	C	T	C	C	T	T	T	C
2	MuVi/Shanxi.CHN/52.10/1	C	G	T	C	A	T	C	C	T	T	T	C
2	MuVi/Guangdong.CHN/21.09/1	C	G	T	C	C	T	C	C	T	T	T	C
2	MuVi/Liaoning.CHN/48.10/2	C	G	T	C	C	T	C	C	T	T	T	C
2	MuVi/Shandong.CHN/51.15/1	C	G	T	C	C	T	C	C	T	T	T	C
2	MuVi/Heilongjiang.CHN/49.15	C	G	T	C	C	T	C	C	T	T	T	C
2	MuVi/Anhui.CHN/10.11/1	C	G	T	C	C	T	C	C	T	T	T	C
2	MuVi/Shannxi.CHN/20.12	C	G	T	C	C	T	C	C	T	T	T	C
3	MuVi/Shanxi.CHN/52.10/3	C	G	T	C	C	T	C	C	T	T	T	C
3	MuVi/Jiangsu.CHN/3.13/1	C	G	T	C	C	T	C	C	T	T	T	C
3	MuVi/Shandong.CHN/4.05	C	G	T	C	C	T	T	C	T	T	T	C
3	MuVi/Shannxi.CHN/9.09/2	C	G	T	C	C	T	C	C	T	T	T	C
3	MuVi/Incheon.KOR/16.08/22	C	G	T	C	C	T	T	C	T	T	T	C
3	MuVi/Henan.CHN/48.06	C	G	T	C	C	T	C	C	T	T	T	C
3	MuVi/Jiangsu.CHN/9.12	C	G	T	C	C	T	C	C	T	T	T	C
3	MuVi/Jiangsu.CHN/4.13/3	C	G	T	C	C	T	C	C	T	T	T	C
4	MuVi/Shannxi.CHN/26.09/1	C	G	T	C	C	T	C	C	T	T	T	C
4	MuVi/Jiangsu.CHN/15.12	C	G	T	C	C	T	C	C	T	T	T	C
1	MuVi/Jilin.CHN/15.08/5	C	G	T	C	C	T	C	C	T	C	T	C
1	MuVi/Jilin.CHN/15.08/1	C	G	T	C	C	T	C	C	T	C	T	C
1	MuVi/Jilin.CHN/15.08/3	C	G	T	C	C	T	C	C	T	C	T	C
1	MuVi/Yunnan.CHN/47.10/2	C	G	T	C	C	T	C	C	T	C	T	C
1	MuVi/Neimeng.CHN/18.11	C	G	T	C	C	T	C	C	C	C	T	C
1	MuVi/Sichuan.CHN/23.12/1	C	G	T	C	C	T	C	C	T	C	T	C
1	MuVi/Jiangsu.CHN/3.13/2	C	G	T	C	C	T	C	C	T	T	T	C
1	MuVi/Beijing.CHN/25.06	C	G	T	C	C	T	A	C	T	T	T	C
1	MuVi/Zhejiang.CHN/26.05	C	G	T	C	C	T	C	C	T	T	T	C
1	MuVi/Shanghai.CHN/0.01	C	G	T	C	C	C	C	C	T	T	T	C
1	MuVi/Shandong.CHN/3.05	C	G	T	C	C	T	C	C	T	T	T	C
1	MuVi/Gansu.CHN/0.02	C	G	T	C	C	T	C	C	T	T	T	C
1	MuVi/Beijing.CHN/15.06	C	G	T	C	C	T	C	C	T	T	T	C
1	MuVi/Shandong.CHN/11.07	C	G	T	C	C	T	C	C	T	T	T	C
1	MuVi/Sichuan.CHN/24.15	C	G	T	C	C	T	C	C	T	T	T	C
1	MuVi/Heilongjiang.CHN/24.14	C	G	T	C	C	T	C	C	T	T	T	C
1	MuVi/Shandong.CHN/43.07	C	G	T	C	C	T	C	C	T	T	T	C
1	MuVi/Shandong.CHN/10.08	C	G	T	C	C	T	C	C	T	T	T	C
1	MuVi/Heilongjiang.CHN/26.12/2	C	G	T	C	C	T	C	C	T	T	T	C
1	MuVi/Liaoning.CHN/48.11	C	G	T	C	C	T	C	C	T	T	T	C
1	MuVi/Heilongjiang.CHN/23.13/1	C	G	T	C	C	T	C	C	T	T	T	C
1	MuVi/Liaoning.CHN/50.11	C	G	T	C	C	T	C	C	T	T	T	C
1	MuVs/Kaohsiung.TWN/06.08	C	G	T	C	C	T	C	C	T	T	T	C
	MuVi/Jiangsu.CHN/12.13/2	T	G	T	C	C	T	C	C	T	T	T	C
	MuVi/Shannxi.CHN/20.15/1	T	G	T	C	C	T	C	C	T	T	T	C
	MuVi/Beijing.CHN/10.07	C	G	T	C	C	T	C	C	T	T	T	C
	MuVi/Hunan.CHN/30.11/3	T	G	T	C	C	T	C	C	T	T	T	C
	MuVi/Liaoning.CHN/9.10/1	T	G	T	C	C	T	C	C	T	T	T	C
	MuVi/Jiangsu.CHN/7.12	T	G	T	C	C	T	C	C	T	T	C	C
	MuVi/Heilongjiang.CHN/14.13/1	T	G	T	C	C	T	C	C	T	T	C	C
	MuVi/Hunan.CHN/32.11	T	G	T	T	C	T	C	C	T	T	T	C
	MuVi/Hunan.CHN/30.11/1	C	G	T	C	C	T	C	C	T	T	T	C
	MuVi/Jiangsu.CHN/7.13/1	C	G	C	C	C	T	C	C	T	T	T	C
	MuVi/Hunan.CHN/30.11/2	T	G	T	C	C	T	C	C	T	T	T	C
	MuVi/Liaoning.CHN/5.09/2	C	G	T	C	C	T	C	C	T	T	T	C
	Mui/Zhejiang.CHN/11.06/1	C	A	T	C	C	T	C	C	T	T	T	A
	MuVi/Liaoning.CHN/13.12	T	G	T	C	C	T	C	C	T	T	T	C
	MuVi/Beijing.CHN/17.07/2	T	G	T	C	C	T	C	C	T	T	T	C
	MuVi/Liaoning.CHN/7.12	T	G	T	C	C	T	C	C	T	T	T	C
	MuVi/Beijing.CHN/21.11	T	G	T	C	C	T	C	C	T	T	T	C
	MuVi/Liaoning.CHN/11.12	T	G	T	C	C	T	C	C	T	T	T	C
	MuVi/Heilongjiang.CHN/26.12/1	T	G	T	C	C	T	C	C	T	T	T	C
	MuVi/Liaoning.CHN/1.08/1	T	G	T	C	C	T	C	C	T	T	T	C
	MuVi/Liaoning.CHN/48.10/1	T	G	T	C	C	T	C	C	T	T	T	C
	MuVi/Jiangsu.CHN/4.13/5	T	G	T	C	C	T	C	C	T	T	T	C
	MuVi/Liaoning.CHN/10.09	T	G	T	C	C	T	C	C	T	T	T	C
	MuVi/Liaoning.CHN/1.08/2	T	G	T	C	C	T	C	C	T	T	T	C
	MuVi/Liaoning.CHN/5.09/1	T	G	T	C	C	T	C	C	T	T	T	C
	MuVi/Heilongjiang.CHN/22.12	T	G	T	C	C	T	C	C	T	T	T	C
	MuVi/Heilongjiang.CHN/15.13	T	G	T	C	C	T	C	C	T	T	T	C
	MuVi/Sichuan.CHN/23.12/2	T	G	T	C	C	T	C	C	T	T	T	C
	MuVi/Hubei.CHN/44.12/1	T	G	T	C	C	T	C	C	T	T	T	C
	MuVi/Jiangsu.CHN/2.13/4	T	G	T	C	C	T	C	C	T	T	T	C

1: Lineage based on Figure 1
2: SNPs are noted in green.
3: SNP position corresponding to a lineage-defining node ir

Supplementary Table S2: Listing of SNPs featured in the 7

Lineage [1]	Strain name	1575	1578	1581	1584	1586	1587	1588	1590	1593	1594	1608	1610
2	MuVi/Shanxi.CHN/52.10/2	T	A	A	A	C	C	C	A	T	G	G	A
2	MuVi/Shannxi.CHN/26.09/4	T	A	A	A	C	C	C	A	T	G	G	T
2	MuVi/Shanxi.CHN/52.10/1	T	A	A	A	C	C	C	A	T	G	G	T
2	MuVi/Guangdong.CHN/21.09/1	T	A	A	A	C	C	C	A	T	G	G	T
2	MuVi/Liaoning.CHN/48.10/2	T	A	A	A	C	C	C	A	T	G	G	T
2	MuVi/Shandong.CHN/51.15/1	T	A	A	A	C	C	C	A	T	G	G	T
2	MuVi/Heilongjiang.CHN/49.15	T	A	A	A	C	C	C	A	T	G	G	T
2	MuVi/Anhui.CHN/10.11/1	T	A	A	A	C	C	C	A	T	G	G	T
2	MuVi/Shannxi.CHN/20.12	T	A	A	A	C	C	C	A	T	G	G	T
3	MuVi/Shanxi.CHN/52.10/3	T	A	A	A	C	C	C	A	T	G	G	T
3	MuVi/Jiangsu.CHN/3.13/1	T	A	A	A	C	C	C	A	T	G	G	T
3	MuVi/Shandong.CHN/4.05	T	A	A	A	C	C	T	A	T	G	G	T
3	MuVi/Shannxi.CHN/9.09/2	T	A	C	A	C	C	C	A	T	G	G	T
3	MuVi/Incheon.KOR/16.08/22	T	A	A	A	C	C	C	A	T	G	G	T
3	MuVi/Henan.CHN/48.06	C	A	A	A	T	C	C	A	C	G	G	T
3	MuVi/Jiangsu.CHN/9.12	C	A	A	A	T	C	C	A	C	G	G	T
3	MuVi/Jiangsu.CHN/4.13/3	C	A	A	A	T	C	C	A	C	G	G	T
4	MuVi/Shannxi.CHN/26.09/1	T	A	A	A	C	C	C	A	T	G	G	T
4	MuVi/Jiangsu.CHN/15.12	T	A	A	A	C	C	C	A	T	G	G	T
1	MuVi/Jilin.CHN/15.08/5	T	A	A	A	C	C	C	A	T	G	G	T
1	MuVi/Jilin.CHN/15.08/1	T	A	A	A	C	C	C	A	T	G	G	T
1	MuVi/Jilin.CHN/15.08/3	T	A	A	A	C	C	C	A	T	G	G	T
1	MuVi/Yunnan.CHN/47.10/2	T	A	A	A	C	C	C	A	T	G	G	T
1	MuVi/Neimeng.CHN/18.11	T	A	A	A	C	C	C	A	T	G	G	T
1	MuVi/Sichuan.CHN/23.12/1	T	A	A	A	C	C	C	A	T	G	G	T
1	MuVi/Jiangsu.CHN/3.13/2	T	A	A	A	C	C	C	A	T	G	G	T
1	MuVi/Beijing.CHN/25.06	T	A	A	A	C	C	C	A	T	G	G	T
1	MuVi/Zhejiang.CHN/26.05	T	A	A	A	C	C	C	A	T	G	G	T
1	MuVi/Shanghai.CHN/0.01	T	A	A	A	C	C	C	A	T	G	G	T
1	MuVi/Shandong.CHN/3.05	T	A	A	A	C	C	C	A	T	G	G	T
1	MuVi/Gansu.CHN/0.02	T	A	A	A	C	C	C	A	T	G	G	T
1	MuVi/Beijing.CHN/15.06	T	A	A	A	C	T	C	A	T	G	G	T
1	MuVi/Shandong.CHN/11.07	T	A	A	A	C	C	C	A	T	G	G	T
1	MuVi/Sichuan.CHN/24.15	T	G	A	A	C	C	C	A	T	G	G	T
1	MuVi/Heilongjiang.CHN/24.14	T	A	A	A	C	C	C	A	T	G	G	T
1	MuVi/Shandong.CHN/43.07	T	A	A	A	C	C	C	A	T	G	G	T
1	MuVi/Shandong.CHN/10.08	T	A	A	A	C	C	C	A	T	G	G	T
1	MuVi/Heilongjiang.CHN/26.12/2	T	A	A	A	C	C	C	A	T	G	G	T
1	MuVi/Liaoning.CHN/48.11	T	A	A	A	C	C	C	A	T	G	G	T
1	MuVi/Heilongjiang.CHN/23.13/1	T	A	A	A	C	C	C	A	T	G	G	T
1	MuVi/Liaoning.CHN/50.11	T	A	A	A	C	C	C	A	T	G	G	T
1	MuVs/Kaohsiung.TWN/06.08	T	A	A	A	C	C	C	A	T	G	G	T
	MuVi/Jiangsu.CHN/12.13/2	T	A	A	A	C	C	C	T	T	G	G	T
	MuVi/Shannxi.CHN/20.15/1	T	A	A	A	C	C	C	T	T	G	G	T
	MuVi/Beijing.CHN/10.07	T	A	A	A	C	C	C	A	T	G	A	T
	MuVi/Hunan.CHN/30.11/3	T	A	A	A	C	C	C	T	T	G	G	T
	MuVi/Liaoning.CHN/9.10/1	T	A	A	A	C	C	C	T	T	G	G	T
	MuVi/Jiangsu.CHN/7.12	T	A	A	A	C	C	C	T	T	G	G	T
	MuVi/Heilongjiang.CHN/14.13/1	T	A	A	A	C	C	C	T	T	G	G	T
	MuVi/Hunan.CHN/32.11	T	A	A	A	C	C	C	A	T	G	G	T
	MuVi/Hunan.CHN/30.11/1	T	A	A	A	C	C	C	A	T	G	G	T
	MuVi/Jiangsu.CHN/7.13/1	T	A	A	A	C	C	C	A	T	G	G	T
	MuVi/Hunan.CHN/30.11/2	T	A	A	A	C	C	C	T	T	G	G	T
	MuVi/Liaoning.CHN/5.09/2	T	A	A	A	C	C	C	A	T	G	G	T
	Mui/Zhejiang.CHN/11.06/1	T	A	A	A	C	C	C	A	T	G	G	T
	MuVi/Liaoning.CHN/13.12	T	A	A	A	C	C	C	A	T	G	G	T
	MuVi/Beijing.CHN/17.07/2	T	A	A	A	C	C	C	A	T	G	G	T
	MuVi/Liaoning.CHN/7.12	T	A	A	G	C	C	C	A	T	G	G	T
	MuVi/Beijing.CHN/21.11	T	A	A	A	C	C	C	A	T	G	G	T
	MuVi/Liaoning.CHN/11.12	T	A	A	A	C	C	C	A	T	G	G	T
	MuVi/Heilongjiang.CHN/26.12/1	T	A	A	A	C	C	C	A	T	G	G	T
	MuVi/Liaoning.CHN/1.08/1	T	A	A	A	C	C	C	A	T	A	G	T
	MuVi/Liaoning.CHN/48.10/1	T	A	A	A	C	C	C	A	T	G	G	T
	MuVi/Jiangsu.CHN/4.13/5	T	A	A	A	C	C	C	A	T	G	G	T
	MuVi/Liaoning.CHN/10.09	T	A	A	A	C	C	C	A	T	G	G	T
	MuVi/Liaoning.CHN/1.08/2	T	A	A	A	C	C	C	A	T	G	G	T
	MuVi/Liaoning.CHN/5.09/1	T	A	A	A	C	C	C	A	T	G	G	T
	MuVi/Heilongjiang.CHN/22.12	T	A	A	A	C	C	C	A	T	G	G	T
	MuVi/Heilongjiang.CHN/15.13	T	A	A	A	C	C	C	A	T	G	G	T
	MuVi/Sichuan.CHN/23.12/2	T	A	A	A	C	C	C	A	T	G	G	T
	MuVi/Hubei.CHN/44.12/1	T	A	A	A	C	C	C	A	T	G	G	T
	MuVi/Jiangsu.CHN/2.13/4	T	A	A	A	C	C	C	A	T	G	G	T

1: Lineage based on Figure 1
2: SNPs are noted in green.
3: SNP position corresponding to a lineage-defining node ir

Supplementary Table S2: Listing of SNPs featured in the 7:

Lineage [1]	Strain name	1614	1617	1620	1623	1629	1632	1635	1656	1662	1665	1674	1677
2	MuVi/Shanxi.CHN/52.10/2	C	G	C	C	C	C	C	T	G	T	T	T
2	MuVi/Shannxi.CHN/26.09/4	C	G	C	C	C	C	C	T	G	T	T	T
2	MuVi/Shanxi.CHN/52.10/1	C	G	C	C	C	C	C	T	G	T	T	T
2	MuVi/Guangdong.CHN/21.09/1	C	G	C	C	C	C	C	T	G	T	T	T
2	MuVi/Liaoning.CHN/48.10/2	C	G	C	C	C	C	C	T	G	T	T	T
2	MuVi/Shandong.CHN/51.15/1	C	G	C	C	C	C	C	T	G	T	T	T
2	MuVi/Heilongjiang.CHN/49.15	C	G	C	C	C	A	C	T	G	T	T	T
2	MuVi/Anhui.CHN/10.11/1	C	G	C	C	C	C	C	T	G	T	T	T
2	MuVi/Shannxi.CHN/20.12	C	G	C	C	C	C	C	T	G	T	T	T
3	MuVi/Shanxi.CHN/52.10/3	T	G	C	C	C	C	C	T	G	T	T	T
3	MuVi/Jiangsu.CHN/3.13/1	T	G	C	C	C	C	C	T	G	T	T	T
3	MuVi/Shandong.CHN/4.05	C	G	C	C	C	C	C	T	G	T	T	T
3	MuVi/Shannxi.CHN/9.09/2	C	T	C	C	C	C	C	T	G	T	T	T
3	MuVi/Incheon.KOR/16.08/22	C	G	C	C	C	C	C	T	G	T	T	T
3	MuVi/Henan.CHN/48.06	C	T	T	C	C	C	C	T	G	C	T	T
3	MuVi/Jiangsu.CHN/9.12	C	T	T	C	C	C	C	T	G	C	T	T
3	MuVi/Jiangsu.CHN/4.13/3	C	T	T	C	C	C	C	T	G	C	T	T
4	MuVi/Shannxi.CHN/26.09/1	C	G	C	C	C	C	C	T	G	T	T	T
4	MuVi/Jiangsu.CHN/15.12	C	G	C	C	C	C	C	T	G	T	T	T
1	MuVi/Jilin.CHN/15.08/5	C	G	C	C	C	C	C	T	G	T	C	T
1	MuVi/Jilin.CHN/15.08/1	C	G	C	C	C	C	C	T	G	T	C	T
1	MuVi/Jilin.CHN/15.08/3	C	G	C	C	C	C	C	T	G	T	C	T
1	MuVi/Yunnan.CHN/47.10/2	C	G	C	C	C	C	C	T	G	T	C	T
1	MuVi/Neimeng.CHN/18.11	C	G	C	C	C	C	C	T	G	T	C	T
1	MuVi/Sichuan.CHN/23.12/1	C	G	C	C	C	C	C	T	G	T	C	T
1	MuVi/Jiangsu.CHN/3.13/2	C	G	C	C	C	C	C	T	G	T	C	T
1	MuVi/Beijing.CHN/25.06	T	G	C	C	C	C	C	T	G	T	C	T
1	MuVi/Zhejiang.CHN/26.05	C	G	C	C	C	C	C	T	G	T	C	T
1	MuVi/Shanghai.CHN/0.01	C	G	C	C	C	C	C	T	G	T	C	T
1	MuVi/Shandong.CHN/3.05	C	G	C	C	C	C	C	T	G	T	C	T
1	MuVi/Gansu.CHN/0.02	C	G	C	C	C	C	C	T	G	T	T	T
1	MuVi/Beijing.CHN/15.06	C	G	C	C	C	C	C	T	G	T	T	T
1	MuVi/Shandong.CHN/11.07	C	G	C	C	C	C	C	T	G	T	T	T
1	MuVi/Sichuan.CHN/24.15	C	G	C	C	C	C	C	T	G	T	T	T
1	MuVi/Heilongjiang.CHN/24.14	C	G	C	C	C	C	C	T	G	T	T	T
1	MuVi/Shandong.CHN/43.07	C	G	C	C	C	C	C	T	G	T	T	T
1	MuVi/Shandong.CHN/10.08	C	G	C	C	C	C	C	T	G	T	T	T
1	MuVi/Heilongjiang.CHN/26.12/2	C	G	C	C	C	C	C	T	G	T	T	T
1	MuVi/Liaoning.CHN/48.11	C	G	C	C	T	C	C	T	G	T	T	T
1	MuVi/Heilongjiang.CHN/23.13/1	C	G	C	C	T	C	C	T	G	T	T	T
1	MuVi/Liaoning.CHN/50.11	C	G	C	C	T	C	C	T	G	T	T	T
1	MuVs/Kaohsiung.TWN/06.08	C	G	C	C	C	C	C	T	G	T	T	T
	MuVi/Jiangsu.CHN/12.13/2	C	G	T	C	C	C	C	T	G	T	T	T
	MuVi/Shannxi.CHN/20.15/1	C	G	T	C	C	C	C	T	G	T	C	T
	MuVi/Beijing.CHN/10.07	C	G	C	C	C	C	C	T	G	T	T	T
	MuVi/Hunan.CHN/30.11/3	C	T	C	T	C	C	T	T	G	T	T	T
	MuVi/Liaoning.CHN/9.10/1	C	T	C	C	C	C	T	T	G	T	T	T
	MuVi/Jiangsu.CHN/7.12	C	T	C	C	C	C	T	T	G	T	T	T
	MuVi/Heilongjiang.CHN/14.13/1	C	T	C	C	C	C	T	T	G	T	T	T
	MuVi/Hunan.CHN/32.11	C	G	C	C	C	C	C	T	G	T	T	T
	MuVi/Hunan.CHN/30.11/1	C	G	C	C	C	C	C	T	G	T	T	T
	MuVi/Jiangsu.CHN/7.13/1	C	G	C	C	C	C	C	T	G	T	T	T
	MuVi/Hunan.CHN/30.11/2	C	G	C	C	C	C	C	C	G	T	T	T
	MuVi/Liaoning.CHN/5.09/2	C	G	C	C	C	C	C	T	G	T	T	T
	Mui/Zhejiang.CHN/11.06/1	C	T	C	C	C	C	C	T	G	T	T	C
	MuVi/Liaoning.CHN/13.12	C	G	C	C	C	C	C	T	A	T	T	T
	MuVi/Beijing.CHN/17.07/2	C	G	C	C	C	C	C	T	A	T	T	T
	MuVi/Liaoning.CHN/7.12	C	G	C	C	C	C	C	T	G	T	T	T
	MuVi/Beijing.CHN/21.11	C	G	C	C	C	C	C	T	G	T	T	T
	MuVi/Liaoning.CHN/11.12	C	G	C	C	C	C	C	T	G	T	T	T
	MuVi/Heilongjiang.CHN/26.12/1	C	G	C	C	C	C	C	T	G	T	T	T
	MuVi/Liaoning.CHN/1.08/1	C	G	C	C	C	C	C	T	G	T	T	T
	MuVi/Liaoning.CHN/48.10/1	C	G	C	C	C	C	C	T	G	T	T	T
	MuVi/Jiangsu.CHN/4.13/5	C	G	C	C	C	C	C	T	G	T	T	T
	MuVi/Liaoning.CHN/10.09	C	G	C	C	C	C	C	T	G	T	T	T
	MuVi/Liaoning.CHN/1.08/2	C	G	C	C	C	C	C	T	G	T	T	T
	MuVi/Liaoning.CHN/5.09/1	C	G	C	C	C	C	C	T	G	T	T	T
	MuVi/Heilongjiang.CHN/22.12	C	G	C	C	C	C	C	T	G	T	T	T
	MuVi/Heilongjiang.CHN/15.13	C	G	C	C	C	C	C	T	G	T	T	T
	MuVi/Sichuan.CHN/23.12/2	C	G	C	C	C	C	C	T	G	T	T	T
	MuVi/Hubei.CHN/44.12/1	C	G	C	C	C	C	C	T	A	T	T	T
	MuVi/Jiangsu.CHN/2.13/4	C	G	C	C	C	C	C	T	A	T	T	T

1: Lineage based on Figure 1
2: SNPs are noted in green.
3: SNP position corresponding to a lineage-defining node ir

Supplementary Table S2: Listing of SNPs featured in the 7

Lineage [1]	Strain name	1683	1692	1701	1707	1716	1719	1722	1725	1731	1734	1735	1737
2	MuVi/Shanxi.CHN/52.10/2	A	G	T	A	T	A	T	G	C	A	C	G
2	MuVi/Shannxi.CHN/26.09/4	A	G	T	A	T	A	T	G	C	A	C	G
2	MuVi/Shanxi.CHN/52.10/1	G	G	T	A	T	A	T	G	C	A	C	G
2	MuVi/Guangdong.CHN/21.09/1	A	G	T	A	T	A	T	G	C	A	C	G
2	MuVi/Liaoning.CHN/48.10/2	A	G	T	A	T	A	T	G	C	A	C	G
2	MuVi/Shandong.CHN/51.15/1	A	G	T	A	T	A	T	G	C	A	C	G
2	MuVi/Heilongjiang.CHN/49.15	A	G	T	A	T	A	T	G	C	A	C	G
2	MuVi/Anhui.CHN/10.11/1	A	G	T	A	T	A	T	G	C	A	C	G
2	MuVi/Shannxi.CHN/20.12	A	G	T	A	T	A	T	G	C	A	C	G
3	MuVi/Shanxi.CHN/52.10/3	A	T	T	A	T	A	T	G	C	A	C	G
3	MuVi/Jiangsu.CHN/3.13/1	A	G	T	A	T	A	T	G	C	A	C	G
3	MuVi/Shandong.CHN/4.05	A	G	T	A	T	A	T	G	C	A	C	G
3	MuVi/Shannxi.CHN/9.09/2	A	G	T	A	T	A	T	G	C	A	C	G
3	MuVi/Incheon.KOR/16.08/22	A	G	T	A	T	A	T	G	C	A	C	G
3	MuVi/Henan.CHN/48.06	A	G	T	A	T	A	T	G	C	A	C	G
3	MuVi/Jiangsu.CHN/9.12	A	G	T	A	T	A	T	G	T	A	C	G
3	MuVi/Jiangsu.CHN/4.13/3	A	G	T	A	T	A	T	G	T	A	C	G
4	MuVi/Shannxi.CHN/26.09/1	A	G	T	A	T	A	T	G	C	A	C	G
4	MuVi/Jiangsu.CHN/15.12	A	G	T	A	T	A	T	G	C	A	C	G
1	MuVi/Jilin.CHN/15.08/5	A	G	T	A	T	A	T	G	C	A	C	G
1	MuVi/Jilin.CHN/15.08/1	A	G	T	A	T	A	T	G	C	A	C	G
1	MuVi/Jilin.CHN/15.08/3	A	G	T	A	T	A	T	G	C	A	C	G
1	MuVi/Yunnan.CHN/47.10/2	A	G	T	A	T	A	T	G	C	A	C	G
1	MuVi/Neimeng.CHN/18.11	A	G	T	A	T	A	T	G	C	A	C	G
1	MuVi/Sichuan.CHN/23.12/1	A	G	T	A	T	A	T	G	C	A	C	G
1	MuVi/Jiangsu.CHN/3.13/2	A	G	T	A	T	A	T	G	C	A	C	G
1	MuVi/Beijing.CHN/25.06	A	G	T	A	T	A	T	G	C	A	C	G
1	MuVi/Zhejiang.CHN/26.05	A	G	T	A	T	A	T	G	C	A	C	G
1	MuVi/Shanghai.CHN/0.01	A	G	T	A	T	A	T	G	C	A	C	G
1	MuVi/Shandong.CHN/3.05	A	G	T	A	T	A	T	G	C	A	C	G
1	MuVi/Gansu.CHN/0.02	A	G	T	A	T	A	T	G	C	A	C	G
1	MuVi/Beijing.CHN/15.06	A	G	T	A	T	A	T	G	C	A	C	G
1	MuVi/Shandong.CHN/11.07	A	G	T	A	T	A	T	G	C	A	C	G
1	MuVi/Sichuan.CHN/24.15	A	G	T	A	T	A	T	G	C	A	C	G
1	MuVi/Heilongjiang.CHN/24.14	A	G	T	A	T	A	T	G	C	A	C	G
1	MuVi/Shandong.CHN/43.07	A	G	T	A	T	A	T	G	C	A	C	G
1	MuVi/Shandong.CHN/10.08	A	G	T	A	T	A	T	G	C	A	C	G
1	MuVi/Heilongjiang.CHN/26.12/2	A	G	T	A	T	A	T	G	C	A	C	G
1	MuVi/Liaoning.CHN/48.11	A	G	T	A	T	A	T	G	C	A	C	G
1	MuVi/Heilongjiang.CHN/23.13/1	A	G	T	A	T	A	T	G	C	A	C	G
1	MuVi/Liaoning.CHN/50.11	A	G	T	A	T	T	T	G	C	A	C	G
1	MuVs/Kaohsiung.TWN/06.08	A	G	T	A	T	A	T	G	C	A	C	G
	MuVi/Jiangsu.CHN/12.13/2	A	G	G	A	T	A	T	G	C	A	C	G
	MuVi/Shannxi.CHN/20.15/1	A	G	T	A	T	A	C	G	C	A	C	G
	MuVi/Beijing.CHN/10.07	A	G	T	A	T	A	T	G	C	A	C	G
	MuVi/Hunan.CHN/30.11/3	A	G	T	A	T	A	T	G	C	A	C	G
	MuVi/Liaoning.CHN/9.10/1	A	G	T	A	T	A	T	G	C	A	T	G
	MuVi/Jiangsu.CHN/7.12	A	G	T	A	T	A	T	G	C	A	T	G
	MuVi/Heilongjiang.CHN/14.13/1	A	G	T	A	T	A	T	G	C	A	T	G
	MuVi/Hunan.CHN/32.11	A	G	T	A	T	A	T	G	C	A	C	G
	MuVi/Hunan.CHN/30.11/1	A	G	T	G	T	A	T	G	C	A	C	G
	MuVi/Jiangsu.CHN/7.13/1	A	G	T	A	T	A	T	G	C	A	C	G
	MuVi/Hunan.CHN/30.11/2	A	G	T	A	T	A	T	G	C	A	C	G
	MuVi/Liaoning.CHN/5.09/2	A	G	T	A	T	A	T	A	C	A	C	G
	Mui/Zhejiang.CHN/11.06/1	A	G	T	A	T	A	T	G	C	A	C	G
	MuVi/Liaoning.CHN/13.12	A	G	T	A	T	A	T	G	C	A	C	G
	MuVi/Beijing.CHN/17.07/2	A	G	T	A	T	A	T	G	C	A	C	G
	MuVi/Liaoning.CHN/7.12	A	G	T	G	T	A	T	G	C	A	C	G
	MuVi/Beijing.CHN/21.11	G	G	T	A	T	A	T	G	C	A	C	G
	MuVi/Liaoning.CHN/11.12	A	G	T	A	T	A	T	G	C	A	C	T
	MuVi/Heilongjiang.CHN/26.12/1	A	A	T	A	T	A	T	G	C	A	C	G
	MuVi/Liaoning.CHN/1.08/1	A	G	T	A	T	A	T	G	C	A	C	G
	MuVi/Liaoning.CHN/48.10/1	A	G	T	A	T	A	T	G	C	A	C	G
	MuVi/Jiangsu.CHN/4.13/5	A	G	T	A	T	A	T	G	C	A	C	G
	MuVi/Liaoning.CHN/10.09	A	G	T	A	T	A	T	G	C	G	C	G
	MuVi/Liaoning.CHN/1.08/2	A	G	T	A	T	A	T	G	C	G	C	G
	MuVi/Liaoning.CHN/5.09/1	A	G	T	A	T	A	T	G	C	G	C	G
	MuVi/Heilongjiang.CHN/22.12	A	G	T	A	T	A	T	G	C	A	C	G
	MuVi/Heilongjiang.CHN/15.13	A	G	T	A	T	A	T	G	C	A	C	G
	MuVi/Sichuan.CHN/23.12/2	A	G	T	A	T	A	T	G	C	A	C	G
	MuVi/Hubei.CHN/44.12/1	A	G	T	A	C	A	T	G	C	A	C	G
	MuVi/Jiangsu.CHN/2.13/4	A	G	T	A	C	A	T	G	C	A	C	G

1: Lineage based on Figure 1
2: SNPs are noted in green.
3: SNP position corresponding to a lineage-defining node ir

Supplementary Table S2: Listing of SNPs featured in the 7:

Lineage [1]	Strain name	1740	1743	1745	1746
2	MuVi/Shanxi.CHN/52.10/2	C	C	C	T
2	MuVi/Shannxi.CHN/26.09/4	C	C	C	T
2	MuVi/Shanxi.CHN/52.10/1	C	C	C	T
2	MuVi/Guangdong.CHN/21.09/1	C	C	C	T
2	MuVi/Liaoning.CHN/48.10/2	C	C	C	T
2	MuVi/Shandong.CHN/51.15/1	C	C	C	T
2	MuVi/Heilongjiang.CHN/49.15	C	A	C	T
2	MuVi/Anhui.CHN/10.11/1	C	C	C	T
2	MuVi/Shannxi.CHN/20.12	C	C	C	T
3	MuVi/Shanxi.CHN/52.10/3	C	C	C	T
3	MuVi/Jiangsu.CHN/3.13/1	C	C	C	T
3	MuVi/Shandong.CHN/4.05	C	C	C	T
3	MuVi/Shannxi.CHN/9.09/2	C	C	C	T
3	MuVi/Incheon.KOR/16.08/22	T	C	C	T
3	MuVi/Henan.CHN/48.06	C	C	C	T
3	MuVi/Jiangsu.CHN/9.12	C	C	C	T
3	MuVi/Jiangsu.CHN/4.13/3	C	C	C	T
4	MuVi/Shannxi.CHN/26.09/1	C	C	C	T
4	MuVi/Jiangsu.CHN/15.12	C	C	C	T
1	MuVi/Jilin.CHN/15.08/5	C	C	C	T
1	MuVi/Jilin.CHN/15.08/1	C	C	C	T
1	MuVi/Jilin.CHN/15.08/3	C	C	C	T
1	MuVi/Yunnan.CHN/47.10/2	C	C	C	T
1	MuVi/Neimeng.CHN/18.11	C	C	C	T
1	MuVi/Sichuan.CHN/23.12/1	C	C	C	T
1	MuVi/Jiangsu.CHN/3.13/2	C	C	C	T
1	MuVi/Beijing.CHN/25.06	C	C	C	T
1	MuVi/Zhejiang.CHN/26.05	C	C	C	T
1	MuVi/Shanghai.CHN/0.01	C	C	C	T
1	MuVi/Shandong.CHN/3.05	C	C	C	T
1	MuVi/Gansu.CHN/0.02	C	C	T	T
1	MuVi/Beijing.CHN/15.06	C	C	T	T
1	MuVi/Shandong.CHN/11.07	C	C	T	T
1	MuVi/Sichuan.CHN/24.15	C	C	T	T
1	MuVi/Heilongjiang.CHN/24.14	C	C	T	T
1	MuVi/Shandong.CHN/43.07	C	C	T	T
1	MuVi/Shandong.CHN/10.08	C	C	T	T
1	MuVi/Heilongjiang.CHN/26.12/2	C	C	T	T
1	MuVi/Liaoning.CHN/48.11	C	C	T	T
1	MuVi/Heilongjiang.CHN/23.13/1	C	C	T	T
1	MuVi/Liaoning.CHN/50.11	C	C	T	T
1	MuVs/Kaohsiung.TWN/06.08	C	C	T	T
	MuVi/Jiangsu.CHN/12.13/2	C	C	C	T
	MuVi/Shannxi.CHN/20.15/1	C	C	C	T
	MuVi/Beijing.CHN/10.07	C	C	C	T
	MuVi/Hunan.CHN/30.11/3	C	C	C	T
	MuVi/Liaoning.CHN/9.10/1	C	C	C	T
	MuVi/Jiangsu.CHN/7.12	C	C	C	T
	MuVi/Heilongjiang.CHN/14.13/1	C	C	C	T
	MuVi/Hunan.CHN/32.11	C	C	C	T
	MuVi/Hunan.CHN/30.11/1	C	C	C	T
	MuVi/Jiangsu.CHN/7.13/1	C	C	C	T
	MuVi/Hunan.CHN/30.11/2	C	C	C	T
	MuVi/Liaoning.CHN/5.09/2	C	C	C	T
	Mui/Zhejiang.CHN/11.06/1	C	C	C	T
	MuVi/Liaoning.CHN/13.12	C	C	C	T
	MuVi/Beijing.CHN/17.07/2	C	C	C	T
	MuVi/Liaoning.CHN/7.12	C	C	C	T
	MuVi/Beijing.CHN/21.11	C	C	C	T
	MuVi/Liaoning.CHN/11.12	C	C	C	C
	MuVi/Heilongjiang.CHN/26.12/1	C	C	C	T
	MuVi/Liaoning.CHN/1.08/1	C	C	C	T
	MuVi/Liaoning.CHN/48.10/1	C	C	C	T
	MuVi/Jiangsu.CHN/4.13/5	C	C	C	T
	MuVi/Liaoning.CHN/10.09	C	C	C	T
	MuVi/Liaoning.CHN/1.08/2	C	C	C	T
	MuVi/Liaoning.CHN/5.09/1	C	C	C	T
	MuVi/Heilongjiang.CHN/22.12	C	C	C	T
	MuVi/Heilongjiang.CHN/15.13	C	C	C	T
	MuVi/Sichuan.CHN/23.12/2	C	C	C	T
	MuVi/Hubei.CHN/44.12/1	C	C	C	T
	MuVi/Jiangsu.CHN/2.13/4	C	C	C	T

1: Lineage based on Figure 1
2: SNPs are noted in green.
3: SNP position corresponding to a lineage-defining node ir

Supplementary Table S3: Listing of SNPs featured in the 70 F sequences of MuVs of genotype F

Lineage [1]	Strain name	9 [2]	10	11	12	14	15	17	22	25	27	28	32	36	39	43	47
1	MuVi/Liaoning.CHN/50.11	T	T	T	T	T	A	T	T	T	G	A	T	A	C	T	A
1	MuVi/Liaoning.CHN/48.11	T	T	T	T	T	A	T	T	T	G	A	T	A	C	T	A
1	MuVi/Heilongjiang.CHN/23.13/1	T	T	T	T	T	A	T	C	T	G	A	T	A	C	T	A
1	MuVi/Shandong.CHN/43.07	T	T	T	T	T	A	T	T	T	G	A	T	A	C	T	A
1	MuVi/Shandong.CHN/10.08	T	T	T	T	T	A	T	T	T	G	A	T	A	C	T	A
1	MuVi/Heilongjiang.CHN/26.12/2	T	T	T	T	T	A	T	T	T	G	A	T	A	C	T	A
1	MuVi/Heilongjiang.CHN/24.14	T	T	T	T	T	A	T	T	T	G	A	T	A	C	T	A
1	MuVi/Sichuan.CHN/24.15	T	T	T	T	T	A	T	T	T	G	A	T	A	C	T	A
1	MuVi/Shandong.CHN/11.07	T	T	T	T	T	A	T	T	T	T	A	T	A	C	C	A
1	MuVi/Beijing.CHN/15.06	T	T	T	T	T	A	T	T	T	G	G	T	A	C	T	A
1	MuVi/Gansu.CHN/0.02	T	T	T	T	T	A	T	T	T	G	G	T	A	C	T	A
1	MuVi/Jiangsu.CHN/3.13/2	C	T	T	T	T	A	T	T	T	G	G	T	A	C	T	A
1	MuVi/Beijing.CHN/25.06	C	T	T	T	T	A	T	T	T	G	G	T	A	C	T	A
1	MuVi/Shanghai.CHN/0.01	C	T	T	T	T	A	T	T	T	G	G	T	A	C	T	A
1	MuVi/Shandong.CHN/3.05	C	T	T	T	T	A	T	T	T	G	G	T	A	C	T	A
1	MuVi/Zhejiang.CHN/26.05	C	T	T	T	T	A	T	T	T	G	G	T	A	C	T	A
1	MuVi/Sichuan.CHN/23.12/1	C	T	T	T	T	A	T	T	T	G	G	T	A	C	T	A
1	MuVi/Neimeng.CHN/18.11	C	T	T	T	T	A	T	T	T	G	G	T	A	C	T	A
1	MuVi/Yunnan.CHN/47.10/2	C	T	T	T	T	A	T	T	T	G	G	T	A	C	T	A
1	MuVi/Jilin.CHN/15.08/3	C	T	T	T	T	A	T	T	T	G	G	T	A	C	T	A
1	MuVi/Jilin.CHN/15.08/1	C	C	T	T	T	A	T	T	T	G	G	T	A	C	T	A
1	MuVi/Jilin.CHN/15.08/5	C	T	T	T	T	A	T	T	T	G	G	T	A	C	T	A
4	MuVi/Shannxi.CHN/26.09/1	T	T	T	T	T	A	T	T	T	G	A	T	A	C	T	A
4	MuVi/Jiangsu.CHN/15.12	T	T	T	T	T	A	T	T	T	G	G	T	A	C	T	A
3	MuVi/Shandong.CHN/4.05	T	T	T	T	T	G	T	T	T	G	G	T	A	C	T	A
3	MuVi/Shannxi.CHN/9.09/2	T	T	T	T	T	A	T	T	T	G	G	T	A	C	T	A
3	MuVi/Shanxi.CHN/52.10/3	T	T	T	T	T	A	T	T	T	G	G	T	A	C	T	A
3	MuVi/Jiangsu.CHN/3.13/1	T	T	T	T	T	A	T	T	T	G	G	T	A	C	T	A
3	MuVi/Henan.CHN/48.06	T	T	T	T	T	A	T	C	T	G	G	T	A	C	T	A
3	MuVi/Jiangsu.CHN/9.12	T	T	T	T	T	A	T	C	T	G	G	T	A	C	T	A
3	MuVi/Jiangsu.CHN/4.13/3	T	T	T	T	T	A	T	C	T	G	G	T	A	C	T	A
2	MuVi/Shanxi.CHN/52.10/2	T	T	T	T	T	T	T	T	T	G	G	T	A	C	T	A
2	MuVi/Shannxi.CHN/26.09/4	T	T	T	C	T	T	T	T	T	G	G	C	A	C	T	A
2	MuVi/Shanxi.CHN/52.10/1	T	T	T	T	T	T	T	T	T	G	G	T	A	C	T	A
2	MuVi/Guangdong.CHN/21.09/1	T	T	T	T	T	T	T	T	T	G	G	T	A	C	T	A
2	MuVi/Heilongjiang.CHN/49.15	T	T	T	T	C	T	T	T	T	G	G	T	A	C	T	A
2	MuVi/Anhui.CHN/10.11/1	T	T	T	T	T	T	T	T	T	G	G	T	A	C	T	A
2	MuVi/Shannxi.CHN/20.12	T	T	T	T	T	T	T	T	T	G	G	T	A	C	T	A
2	MuVi/Liaoning.CHN/48.10/2	T	T	T	T	T	T	T	T	C	G	G	T	A	C	T	A
2	MuVi/Shandong.CHN/51.15/1	T	T	T	T	T	T	T	T	C	G	G	T	A	C	T	A
	MuVi/Hunan.CHN/32.11	T	T	T	T	T	A	T	T	T	G	G	T	A	C	T	A
	MuVi/Hunan.CHN/30.11/2	T	T	T	T	T	A	T	T	T	G	G	T	A	T	T	A
	MuVi/Heilongjiang.CHN/14.13/1	T	T	T	T	T	A	T	T	T	G	G	T	A	C	T	A
	MuVi/Jiangsu.CHN/7.12	T	T	T	T	T	A	T	T	T	G	G	T	A	C	T	A
	MuVi/Liaoning.CHN/9.10/1	T	T	T	T	T	A	T	T	T	G	G	T	A	C	T	A
	MuVi/Hunan.CHN/30.11/3	T	T	T	T	T	A	T	T	T	G	G	T	A	C	T	A
	MuVi/Jiangsu.CHN/12.13/2	T	T	T	T	T	A	T	T	T	G	G	T	A	C	T	A
	MuVi/Shannxi.CHN/20.15/1	T	T	T	C	T	A	T	T	T	G	G	T	A	C	T	A
	MuVi/Liaoning.CHN/13.12	T	T	C	T	T	A	T	T	T	G	G	T	A	C	T	A
	MuVi/Beijing.CHN/21.11	T	T	T	T	T	A	G	T	T	G	G	T	G	C	T	A
	MuVi/Liaoning.CHN/11.12	T	T	T	T	T	A	G	T	T	G	G	T	G	C	T	A
	MuVi/Jiangsu.CHN/4.13/5	T	T	T	T	T	A	G	T	C	G	A	T	A	C	T	A
	MuVi/Heilongjiang.CHN/26.12/1	T	T	T	T	T	A	G	T	T	G	G	T	A	C	T	A
	MuVi/Beijing.CHN/17.07/2	T	T	T	T	T	A	T	T	T	G	G	T	A	C	T	A
	MuVi/Sichuan.CHN/23.12/2	T	T	T	T	T	A	T	T	T	G	T	T	A	C	T	A
	MuVi/Hubei.CHN/44.12/1	T	T	T	T	T	A	T	T	T	G	G	T	A	C	T	A
	MuVi/Jiangsu.CHN/2.13/4	T	T	T	T	T	A	T	T	T	G	G	T	A	C	T	A
	MuVi/Liaoning.CHN/7.12	T	T	T	T	T	A	T	T	T	G	G	T	A	C	T	A
	MuVi/Heilongjiang.CHN/22.12	T	T	T	T	T	A	T	T	T	G	G	T	A	C	T	A
	MuVi/Heilongjiang.CHN/15.13	T	T	T	T	T	A	T	T	T	G	G	T	A	C	T	A
	MuVi/Liaoning.CHN/5.09/1	T	T	T	T	T	A	G	T	T	G	G	T	A	C	T	A
	MuVi/Liaoning.CHN/1.08/2	T	T	T	T	T	A	G	T	T	G	G	T	A	C	T	A
	MuVi/Liaoning.CHN/10.09	T	T	T	T	T	A	G	T	T	G	G	T	A	C	T	A
	MuVi/Liaoning.CHN/1.08/1	T	T	T	T	T	A	G	T	T	G	G	T	A	C	T	A
	MuVi/Liaoning.CHN/48.10/1	T	T	T	T	T	A	G	T	T	G	G	T	A	C	T	A
	MuVi/Beijing.CHN/10.07	T	T	T	T	T	A	T	T	T	G	G	T	A	C	T	G
	MuVi/Hunan.CHN/30.11/1	T	T	T	T	T	T	T	T	T	G	A	T	A	C	T	A
	MuVi/Jiangsu.CHN/7.13/1	T	T	T	T	T	T	T	T	T	G	A	T	A	C	T	A
	MuVi/Liaoning.CHN/5.09/2	T	T	T	T	T	A	T	T	T	G	G	T	A	C	T	A
	MuVi/Zhejiang.CHN/11.06/1	T	T	T	T	T	A	T	T	T	G	G	T	A	C	T	A

1: Lineage based on Figure 1
2: SNPs are noted in green.
3: SNP position corresponding to a lineage-defining node in Figure 1 are indicated in green, blue, red or orange according to Figure 1.

Supplementary Table S3: Listing of SNPs featured in the 70 F sequences of [illegible]

Lineage [1]	Strain name	48	51	54	55	60	66	69	72	78	87	90	95	96	103	111	112
1	MuVi/Liaoning.CHN/50.11	T	T	C	T	G	C	C	C	G	A	T	A	G	G	A	C
1	MuVi/Liaoning.CHN/48.11	T	T	C	T	G	C	C	C	G	A	T	A	G	G	A	C
1	MuVi/Heilongjiang.CHN/23.13/1	T	T	C	T	G	C	C	C	G	A	T	A	G	G	A	C
1	MuVi/Shandong.CHN/43.07	T	T	C	T	G	C	C	C	G	A	T	A	G	A	A	C
1	MuVi/Shandong.CHN/10.08	T	T	C	T	G	C	C	C	G	A	T	A	G	A	A	C
1	MuVi/Heilongjiang.CHN/26.12/2	T	T	C	T	G	C	C	C	G	A	T	A	G	G	A	C
1	MuVi/Heilongjiang.CHN/24.14	T	T	C	T	G	C	C	C	G	A	T	A	G	G	A	C
1	MuVi/Sichuan.CHN/24.15	T	T	C	T	G	C	C	C	G	A	T	A	G	G	A	C
1	MuVi/Shandong.CHN/11.07	C	C	C	T	G	C	C	C	G	A	T	A	G	G	A	T
1	MuVi/Beijing.CHN/15.06	T	T	C	T	G	C	C	C	G	A	T	A	G	G	A	T
1	MuVi/Gansu.CHN/0.02	T	T	C	T	G	C	C	C	G	A	T	A	G	G	A	C
1	MuVi/Jiangsu.CHN/3.13/2	T	T	C	T	G	C	C	C	G	A	T	A	G	G	A	C
1	MuVi/Beijing.CHN/25.06	T	T	C	T	G	C	C	C	G	A	T	A	G	G	A	C
1	MuVi/Shanghai.CHN/0.01	T	T	C	T	G	C	C	C	G	A	T	A	G	G	A	C
1	MuVi/Shandong.CHN/3.05	T	T	C	T	G	C	C	C	G	A	T	A	G	G	A	C
1	MuVi/Zhejiang.CHN/26.05	T	T	C	T	G	C	C	C	G	A	T	A	G	G	A	C
1	MuVi/Sichuan.CHN/23.12/1	T	T	C	T	G	C	T	C	G	A	T	A	G	G	A	C
1	MuVi/Neimeng.CHN/18.11	T	T	C	T	G	C	C	C	G	G	T	A	G	G	A	C
1	MuVi/Yunnan.CHN/47.10/2	T	T	C	T	G	C	C	C	G	A	T	A	G	G	A	C
1	MuVi/Jilin.CHN/15.08/3	T	T	C	T	G	C	C	C	G	A	T	A	A	G	A	C
1	MuVi/Jilin.CHN/15.08/1	T	T	C	T	G	C	C	C	G	A	T	A	A	G	A	C
1	MuVi/Jilin.CHN/15.08/5	T	T	C	T	G	C	C	C	G	A	T	A	A	G	A	C
4	MuVi/Shannxi.CHN/26.09/1	T	T	C	T	G	C	C	C	G	A	T	A	G	G	A	C
4	MuVi/Jiangsu.CHN/15.12	T	T	C	G	G	C	C	C	G	A	T	A	G	G	A	C
3	MuVi/Shandong.CHN/4.05	T	T	C	T	G	C	C	C	G	A	T	A	G	G	A	C
3	MuVi/Shannxi.CHN/9.09/2	T	T	C	T	G	C	C	C	G	A	T	A	G	G	A	C
3	MuVi/Shanxi.CHN/52.10/3	T	T	C	T	G	C	C	C	A	A	T	A	G	G	A	C
3	MuVi/Jiangsu.CHN/3.13/1	T	T	C	T	G	C	C	C	G	A	T	A	G	G	A	C
3	MuVi/Henan.CHN/48.06	T	T	C	T	G	C	C	C	G	A	T	A	G	G	A	C
3	MuVi/Jiangsu.CHN/9.12	T	T	C	T	G	C	C	C	G	A	T	A	G	G	A	C
3	MuVi/Jiangsu.CHN/4.13/3	T	T	C	T	G	C	C	C	G	A	T	A	G	G	A	C
2	MuVi/Shanxi.CHN/52.10/2	T	T	C	T	G	A	C	C	G	A	T	A	G	G	A	C
2	MuVi/Shannxi.CHN/26.09/4	T	T	C	T	G	C	C	C	G	A	T	A	G	G	A	C
2	MuVi/Shanxi.CHN/52.10/1	T	T	C	T	G	C	C	C	G	A	T	A	G	G	A	C
2	MuVi/Guangdong.CHN/21.09/1	T	T	C	T	A	C	C	C	G	A	T	A	G	G	A	C
2	MuVi/Heilongjiang.CHN/49.15	T	T	C	T	G	C	C	C	A	A	T	A	G	G	A	C
2	MuVi/Anhui.CHN/10.11/1	T	T	C	T	G	C	C	C	G	A	T	A	G	G	A	C
2	MuVi/Shannxi.CHN/20.12	T	T	C	T	G	C	C	C	G	A	T	A	G	G	A	C
2	MuVi/Liaoning.CHN/48.10/2	T	T	C	T	G	C	C	C	G	A	T	A	G	G	A	C
2	MuVi/Shandong.CHN/51.15/1	T	T	C	T	G	C	C	C	G	A	T	A	G	G	A	C
	MuVi/Hunan.CHN/32.11	T	T	C	T	G	T	C	C	G	A	T	A	G	G	A	C
	MuVi/Hunan.CHN/30.11/2	T	T	C	T	G	C	C	C	G	A	C	A	G	G	A	C
	MuVi/Heilongjiang.CHN/14.13/1	T	T	C	T	G	C	T	C	G	A	T	A	G	G	A	T
	MuVi/Jiangsu.CHN/7.12	T	T	C	T	G	C	T	C	G	A	T	A	G	G	A	T
	MuVi/Liaoning.CHN/9.10/1	T	T	C	T	G	C	T	C	G	A	T	A	G	G	A	T
	MuVi/Hunan.CHN/30.11/3	T	T	C	T	G	C	T	C	G	A	T	A	G	G	A	T
	MuVi/Jiangsu.CHN/12.13/2	T	T	C	T	G	C	C	C	G	A	T	A	G	G	A	T
	MuVi/Shannxi.CHN/20.15/1	T	T	C	T	G	C	C	C	G	A	T	A	G	G	A	T
	MuVi/Liaoning.CHN/13.12	T	T	C	T	G	C	C	C	G	A	T	G	G	G	A	C
	MuVi/Beijing.CHN/21.11	T	T	C	T	G	C	C	C	G	A	T	A	G	G	G	C
	MuVi/Liaoning.CHN/11.12	T	T	C	T	G	C	C	C	G	A	T	A	G	G	G	C
	MuVi/Jiangsu.CHN/4.13/5	T	T	C	T	G	C	C	C	G	A	T	A	G	G	A	C
	MuVi/Heilongjiang.CHN/26.12/1	T	T	C	T	G	C	C	C	G	A	T	A	G	G	A	C
	MuVi/Beijing.CHN/17.07/2	T	T	C	T	G	C	C	C	G	A	T	A	G	G	A	C
	MuVi/Sichuan.CHN/23.12/2	T	T	T	T	G	C	T	C	G	A	T	A	G	G	A	C
	MuVi/Hubei.CHN/44.12/1	T	T	C	T	G	C	C	C	G	A	T	A	G	G	A	C
	MuVi/Jiangsu.CHN/2.13/4	T	T	C	T	G	C	C	C	G	A	T	A	G	G	A	C
	MuVi/Liaoning.CHN/7.12	T	T	T	T	G	C	C	C	G	A	T	A	G	G	A	C
	MuVi/Heilongjiang.CHN/22.12	T	T	T	T	G	C	C	C	G	A	T	A	G	G	A	C
	MuVi/Heilongjiang.CHN/15.13	T	T	T	T	G	C	C	C	G	A	T	A	G	G	A	C
	MuVi/Liaoning.CHN/5.09/1	T	T	C	T	G	C	C	C	G	A	T	A	G	G	A	C
	MuVi/Liaoning.CHN/1.08/2	T	T	C	T	G	C	C	C	G	A	T	A	G	G	A	C
	MuVi/Liaoning.CHN/10.09	T	T	C	T	G	C	C	C	G	A	T	A	G	G	A	C
	MuVi/Liaoning.CHN/1.08/1	T	T	C	T	G	C	C	C	G	A	T	A	G	G	A	C
	MuVi/Liaoning.CHN/48.10/1	T	T	C	T	G	C	C	C	G	A	T	A	G	G	A	C
	MuVi/Beijing.CHN/10.07	T	T	C	T	G	C	C	C	G	A	T	A	G	G	A	C
	MuVi/Hunan.CHN/30.11/1	T	T	C	T	G	C	C	C	G	A	T	A	G	G	A	C
	MuVi/Jiangsu.CHN/7.13/1	T	T	C	T	G	C	C	C	G	A	T	A	G	G	A	C
	MuVi/Liaoning.CHN/5.09/2	T	T	C	T	G	C	C	C	G	A	T	A	G	G	A	C
	MuVi/Zhejiang.CHN/11.06/1	T	T	C	T	G	C	C	A	G	A	T	A	G	G	A	C

1: Lineage based on Figure 1
2: SNPs are noted in green.
3: SNP position corresponding to a lineage-defining node in Figure 1 [illegible]

Supplementary Table S3: Listing of SNPs featured in the 70 F sequences of [illegible]

Lineage [1]	Strain name	115	117	121	126	129	138	139	144	150	151	160	165	171
1	MuVi/Liaoning.CHN/50.11	A	C	T	A	A	C	T	C	G	G	T	G	C
1	MuVi/Liaoning.CHN/48.11	A	C	T	A	A	C	T	C	G	G	T	G	C
1	MuVi/Heilongjiang.CHN/23.13/1	A	C	T	A	A	C	T	C	G	G	T	G	C
1	MuVi/Shandong.CHN/43.07	A	C	T	A	A	T	T	C	G	G	T	G	C
1	MuVi/Shandong.CHN/10.08	A	C	T	A	A	T	T	C	G	G	T	G	C
1	MuVi/Heilongjiang.CHN/26.12/2	A	C	T	A	A	C	T	C	G	G	T	A	C
1	MuVi/Heilongjiang.CHN/24.14	A	C	T	A	A	C	T	C	G	G	T	G	C
1	MuVi/Sichuan.CHN/24.15	A	C	T	A	A	C	T	C	G	G	T	G	C
1	MuVi/Shandong.CHN/11.07	A	C	T	A	G	T	T	C	G	G	T	G	C
1	MuVi/Beijing.CHN/15.06	A	C	T	A	G	C	T	C	A	G	T	G	C
1	MuVi/Gansu.CHN/0.02	A	C	T	A	A	C	T	C	G	G	T	G	C
1	MuVi/Jiangsu.CHN/3.13/2	A	C	T	G	A	C	T	C	G	G	T	G	C
1	MuVi/Beijing.CHN/25.06	A	C	T	A	A	C	T	C	G	G	T	G	C
1	MuVi/Shanghai.CHN/0.01	A	C	T	A	A	C	T	C	G	G	T	G	C
1	MuVi/Shandong.CHN/3.05	A	C	T	A	A	C	T	C	G	G	C	G	C
1	MuVi/Zhejiang.CHN/26.05	A	C	T	A	A	C	T	C	G	G	T	G	C
1	MuVi/Sichuan.CHN/23.12/1	A	C	T	A	A	C	T	C	G	G	T	G	C
1	MuVi/Neimeng.CHN/18.11	A	C	T	A	A	C	T	C	G	G	T	G	C
1	MuVi/Yunnan.CHN/47.10/2	A	C	T	A	A	C	T	C	G	G	T	G	C
1	MuVi/Jilin.CHN/15.08/3	A	C	T	A	A	C	T	C	G	G	T	G	C
1	MuVi/Jilin.CHN/15.08/1	A	C	T	A	A	C	T	C	G	G	T	G	C
1	MuVi/Jilin.CHN/15.08/5	A	C	T	A	A	C	T	C	G	G	T	G	C
4	MuVi/Shannxi.CHN/26.09/1	A	C	T	A	A	C	T	C	G	G	T	G	T
4	MuVi/Jiangsu.CHN/15.12	A	C	T	A	A	C	T	C	G	G	T	G	T
3	MuVi/Shandong.CHN/4.05	A	T	T	A	A	C	T	C	G	G	T	G	C
3	MuVi/Shannxi.CHN/9.09/2	A	T	T	A	A	C	T	C	G	G	T	G	C
3	MuVi/Shanxi.CHN/52.10/3	A	C	T	A	A	C	G	C	G	G	T	G	C
3	MuVi/Jiangsu.CHN/3.13/1	A	C	T	A	A	C	T	C	G	G	T	G	C
3	MuVi/Henan.CHN/48.06	A	C	T	A	A	C	T	C	G	G	T	G	C
3	MuVi/Jiangsu.CHN/9.12	A	C	T	A	A	C	T	C	G	G	T	G	C
3	MuVi/Jiangsu.CHN/4.13/3	A	C	T	A	A	C	T	C	G	G	T	G	C
2	MuVi/Shanxi.CHN/52.10/2	A	C	T	C	A	C	T	C	G	G	T	G	C
2	MuVi/Shannxi.CHN/26.09/4	A	C	T	A	A	C	T	C	G	G	T	G	C
2	MuVi/Shanxi.CHN/52.10/1	A	C	C	A	A	C	T	C	G	G	T	G	C
2	MuVi/Guangdong.CHN/21.09/1	A	C	T	A	A	C	T	C	G	G	T	G	C
2	MuVi/Heilongjiang.CHN/49.15	A	C	T	A	A	C	T	C	G	G	T	G	C
2	MuVi/Anhui.CHN/10.11/1	A	C	T	A	A	C	T	C	G	G	T	G	C
2	MuVi/Shannxi.CHN/20.12	A	C	T	A	A	C	T	C	G	G	T	G	C
2	MuVi/Liaoning.CHN/48.10/2	A	C	T	A	A	C	T	C	G	G	T	G	C
2	MuVi/Shandong.CHN/51.15/1	A	C	T	A	A	C	T	C	G	G	T	G	C
	MuVi/Hunan.CHN/32.11	C	C	T	A	A	C	T	T	G	A	T	G	T
	MuVi/Hunan.CHN/30.11/2	A	C	T	A	A	C	T	C	G	G	T	G	C
	MuVi/Heilongjiang.CHN/14.13/1	A	C	T	A	A	C	T	C	G	G	T	G	C
	MuVi/Jiangsu.CHN/7.12	A	C	T	A	A	C	T	C	G	G	T	G	C
	MuVi/Liaoning.CHN/9.10/1	A	C	T	A	A	C	T	C	G	G	T	G	C
	MuVi/Hunan.CHN/30.11/3	A	C	T	A	A	C	T	C	G	G	T	G	C
	MuVi/Jiangsu.CHN/12.13/2	A	T	T	A	A	C	T	C	G	G	T	G	C
	MuVi/Shannxi.CHN/20.15/1	A	T	T	A	A	C	T	C	G	G	T	G	C
	MuVi/Liaoning.CHN/13.12	A	C	T	A	A	C	T	C	G	G	T	G	C
	MuVi/Beijing.CHN/21.11	A	C	T	G	A	C	T	C	G	G	T	G	C
	MuVi/Liaoning.CHN/11.12	A	C	T	G	A	C	T	C	G	G	T	G	C
	MuVi/Jiangsu.CHN/4.13/5	A	C	T	A	A	C	T	C	G	G	T	G	C
	MuVi/Heilongjiang.CHN/26.12/1	A	C	T	A	A	C	T	C	G	G	T	G	C
	MuVi/Beijing.CHN/17.07/2	A	C	T	A	G	C	T	C	G	G	T	G	C
	MuVi/Sichuan.CHN/23.12/2	A	C	T	A	A	C	T	C	G	G	T	G	C
	MuVi/Hubei.CHN/44.12/1	A	C	T	A	A	C	T	C	G	G	T	G	C
	MuVi/Jiangsu.CHN/2.13/4	A	C	T	A	A	C	T	C	G	G	T	G	C
	MuVi/Liaoning.CHN/7.12	A	C	T	A	A	C	T	C	G	G	T	G	C
	MuVi/Heilongjiang.CHN/22.12	A	C	T	A	A	C	T	C	G	G	T	G	C
	MuVi/Heilongjiang.CHN/15.13	A	C	T	A	A	C	T	C	G	G	T	G	C
	MuVi/Liaoning.CHN/5.09/1	A	C	T	A	A	C	T	C	G	G	T	G	C
	MuVi/Liaoning.CHN/1.08/2	A	C	T	A	A	C	T	C	G	G	T	G	C
	MuVi/Liaoning.CHN/10.09	A	C	T	A	A	C	T	C	G	G	T	G	C
	MuVi/Liaoning.CHN/1.08/1	A	C	T	A	A	C	T	C	G	G	T	G	C
	MuVi/Liaoning.CHN/48.10/1	A	C	T	A	A	C	T	C	G	G	T	G	C
	MuVi/Beijing.CHN/10.07	A	C	T	A	A	C	T	C	G	G	T	G	C
	MuVi/Hunan.CHN/30.11/1	A	C	T	A	A	C	T	C	G	G	T	G	C
	MuVi/Jiangsu.CHN/7.13/1	A	C	T	A	A	C	T	C	G	G	T	G	C
	MuVi/Liaoning.CHN/5.09/2	A	C	T	A	A	C	T	C	G	G	T	G	C
	MuVi/Zhejiang.CHN/11.06/1	A	C	T	A	A	C	T	C	G	G	T	G	C

1: Lineage based on Figure 1
2: SNPs are noted in green.
3: SNP position corresponding to a lineage-defining node in Figure 1 [illegible]

Supplementary Table S3: Listing of SNPs featured in the 70 F sequences of I

Lineage [1]	Strain name	178	179	183	184	186	198	201	205	213	219	222 [3]	224
1	MuVi/Liaoning.CHN/50.11	A	C	T	A	C	T	G	G	A	T	A	C
1	MuVi/Liaoning.CHN/48.11	A	C	T	A	C	T	G	G	A	T	A	C
1	MuVi/Heilongjiang.CHN/23.13/1	A	C	T	A	C	T	G	G	A	T	A	C
1	MuVi/Shandong.CHN/43.07	A	C	T	A	C	T	G	G	A	T	A	C
1	MuVi/Shandong.CHN/10.08	A	C	T	A	C	T	G	G	A	T	A	C
1	MuVi/Heilongjiang.CHN/26.12/2	A	C	T	A	C	T	G	G	A	T	A	C
1	MuVi/Heilongjiang.CHN/24.14	A	C	T	A	C	T	G	G	A	T	A	C
1	MuVi/Sichuan.CHN/24.15	A	C	T	A	C	T	G	G	A	T	A	C
1	MuVi/Shandong.CHN/11.07	A	C	T	A	C	T	G	G	A	T	A	C
1	MuVi/Beijing.CHN/15.06	A	C	T	A	C	T	G	G	A	T	A	C
1	MuVi/Gansu.CHN/0.02	A	C	T	A	C	T	G	G	A	T	A	C
1	MuVi/Jiangsu.CHN/3.13/2	A	C	T	A	C	T	G	G	A	T	A	C
1	MuVi/Beijing.CHN/25.06	A	C	T	A	C	T	G	G	A	T	A	C
1	MuVi/Shanghai.CHN/0.01	A	C	T	G	T	T	G	G	A	T	A	C
1	MuVi/Shandong.CHN/3.05	A	C	T	G	T	T	G	G	A	T	A	C
1	MuVi/Zhejiang.CHN/26.05	A	C	T	A	T	T	G	G	A	T	A	C
1	MuVi/Sichuan.CHN/23.12/1	A	C	T	A	C	T	G	A	A	T	A	C
1	MuVi/Neimeng.CHN/18.11	A	C	T	A	C	T	G	G	A	T	A	C
1	MuVi/Yunnan.CHN/47.10/2	A	C	T	A	C	T	G	G	A	T	A	C
1	MuVi/Jilin.CHN/15.08/3	A	C	T	A	C	T	G	G	A	T	A	C
1	MuVi/Jilin.CHN/15.08/1	A	C	T	A	C	T	G	G	A	T	A	T
1	MuVi/Jilin.CHN/15.08/5	A	C	T	A	C	T	G	G	A	T	A	C
4	MuVi/Shannxi.CHN/26.09/1	A	C	T	A	C	T	A	G	A	T	G	C
4	MuVi/Jiangsu.CHN/15.12	A	G	T	A	C	T	A	G	A	T	G	C
3	MuVi/Shandong.CHN/4.05	A	C	C	A	C	T	G	G	A	T	G	C
3	MuVi/Shannxi.CHN/9.09/2	A	C	C	A	C	T	G	G	A	T	G	C
3	MuVi/Shanxi.CHN/52.10/3	A	C	T	A	C	T	G	G	A	T	G	C
3	MuVi/Jiangsu.CHN/3.13/1	A	C	T	A	C	T	A	G	A	T	G	C
3	MuVi/Henan.CHN/48.06	G	C	T	A	C	T	G	G	A	T	G	C
3	MuVi/Jiangsu.CHN/9.12	G	C	T	A	C	T	G	G	A	T	G	C
3	MuVi/Jiangsu.CHN/4.13/3	G	C	T	A	C	T	G	G	A	T	G	C
2	MuVi/Shanxi.CHN/52.10/2	A	C	T	A	C	T	G	G	A	T	G	C
2	MuVi/Shannxi.CHN/26.09/4	A	C	T	A	C	T	G	G	A	T	G	C
2	MuVi/Shanxi.CHN/52.10/1	A	C	T	A	C	T	G	G	A	T	G	C
2	MuVi/Guangdong.CHN/21.09/1	A	C	T	A	C	T	G	G	A	T	G	C
2	MuVi/Heilongjiang.CHN/49.15	A	C	T	A	C	T	G	G	A	T	G	C
2	MuVi/Anhui.CHN/10.11/1	A	C	T	A	C	T	G	G	A	T	G	C
2	MuVi/Shannxi.CHN/20.12	A	C	T	A	C	T	G	G	A	T	G	C
2	MuVi/Liaoning.CHN/48.10/2	A	C	T	A	C	T	G	G	A	T	G	C
2	MuVi/Shandong.CHN/51.15/1	A	C	T	A	C	T	G	G	A	T	G	C
	MuVi/Hunan.CHN/32.11	A	C	T	A	C	T	G	G	A	T	G	C
	MuVi/Hunan.CHN/30.11/2	A	C	T	A	C	C	G	G	A	C	G	C
	MuVi/Heilongjiang.CHN/14.13/1	A	C	T	A	C	T	G	G	G	T	G	C
	MuVi/Jiangsu.CHN/7.12	A	C	T	A	C	T	G	G	G	T	G	C
	MuVi/Liaoning.CHN/9.10/1	A	C	T	A	C	T	G	G	G	T	G	C
	MuVi/Hunan.CHN/30.11/3	A	C	T	A	C	T	G	G	A	T	G	C
	MuVi/Jiangsu.CHN/12.13/2	A	C	T	A	C	T	G	G	A	T	G	C
	MuVi/Shannxi.CHN/20.15/1	A	C	T	A	C	T	G	G	A	T	G	C
	MuVi/Liaoning.CHN/13.12	A	C	T	A	C	T	G	G	A	T	G	C
	MuVi/Beijing.CHN/21.11	A	C	T	A	C	T	G	G	A	T	G	C
	MuVi/Liaoning.CHN/11.12	A	C	T	A	C	T	G	G	A	T	G	C
	MuVi/Jiangsu.CHN/4.13/5	A	C	T	A	C	T	G	G	A	T	G	C
	MuVi/Heilongjiang.CHN/26.12/1	A	C	T	A	C	T	G	G	A	T	G	C
	MuVi/Beijing.CHN/17.07/2	A	C	T	A	C	T	G	G	A	T	G	C
	MuVi/Sichuan.CHN/23.12/2	A	C	T	A	C	T	G	G	A	T	G	C
	MuVi/Hubei.CHN/44.12/1	A	C	T	A	C	T	G	G	A	T	G	C
	MuVi/Jiangsu.CHN/2.13/4	A	C	T	A	C	T	G	G	A	T	G	C
	MuVi/Liaoning.CHN/7.12	A	C	T	A	C	T	G	G	A	T	G	C
	MuVi/Heilongjiang.CHN/22.12	A	C	T	A	C	T	G	G	A	T	G	C
	MuVi/Heilongjiang.CHN/15.13	A	C	T	A	C	T	G	G	A	T	G	C
	MuVi/Liaoning.CHN/5.09/1	A	C	T	A	C	T	G	G	A	T	G	C
	MuVi/Liaoning.CHN/1.08/2	A	C	T	A	C	T	G	G	A	T	G	C
	MuVi/Liaoning.CHN/10.09	A	C	T	A	C	T	G	G	A	T	G	C
	MuVi/Liaoning.CHN/1.08/1	A	C	T	A	C	T	G	G	A	T	G	C
	MuVi/Liaoning.CHN/48.10/1	A	C	T	A	C	T	G	G	A	T	G	C
	MuVi/Beijing.CHN/10.07	A	C	T	A	C	C	G	G	A	T	G	C
	MuVi/Hunan.CHN/30.11/1	A	C	T	A	C	T	G	G	A	T	G	C
	MuVi/Jiangsu.CHN/7.13/1	A	C	T	A	C	T	G	G	A	T	G	C
	MuVi/Liaoning.CHN/5.09/2	A	C	T	A	C	T	G	G	A	T	G	C
	MuVi/Zhejiang.CHN/11.06/1	A	C	T	A	C	T	G	G	A	T	G	C

1: Lineage based on Figure 1
2: SNPs are noted in green.
3: SNP position corresponding to a lineage-defining node in Figure 1 a

Supplementary Table S3: Listing of SNPs featured in the 70 F sequences of l

Lineage [1]	Strain name	225	229	241	246	247	249	264	266	270	272	274	276	278
1	MuVi/Liaoning.CHN/50.11	C	A	A	A	A	T	C	C	T	C	T	G	C
1	MuVi/Liaoning.CHN/48.11	C	A	A	A	A	T	C	C	T	C	T	G	C
1	MuVi/Heilongjiang.CHN/23.13/1	C	A	A	A	A	T	C	C	T	C	T	G	C
1	MuVi/Shandong.CHN/43.07	C	A	A	A	A	T	C	C	T	C	T	G	C
1	MuVi/Shandong.CHN/10.08	C	A	A	A	A	T	C	C	T	C	T	G	C
1	MuVi/Heilongjiang.CHN/26.12/2	C	A	A	A	A	T	C	C	T	C	T	G	C
1	MuVi/Heilongjiang.CHN/24.14	C	A	A	A	A	T	C	C	T	C	T	G	C
1	MuVi/Sichuan.CHN/24.15	C	A	A	A	A	T	C	C	T	C	T	G	C
1	MuVi/Shandong.CHN/11.07	C	A	C	A	A	T	C	A	T	C	T	G	C
1	MuVi/Beijing.CHN/15.06	C	A	C	A	A	T	T	A	T	C	T	G	C
1	MuVi/Gansu.CHN/0.02	C	A	C	A	A	T	C	A	T	C	T	G	C
1	MuVi/Jiangsu.CHN/3.13/2	C	A	C	A	A	T	C	A	T	C	C	G	C
1	MuVi/Beijing.CHN/25.06	C	A	C	A	A	A	C	A	T	C	T	G	C
1	MuVi/Shanghai.CHN/0.01	C	A	C	A	A	T	C	A	T	C	T	G	C
1	MuVi/Shandong.CHN/3.05	C	A	C	A	A	T	C	A	T	C	C	G	C
1	MuVi/Zhejiang.CHN/26.05	C	A	C	A	A	T	C	A	T	T	T	G	C
1	MuVi/Sichuan.CHN/23.12/1	C	A	C	A	A	T	C	A	C	C	T	G	C
1	MuVi/Neimeng.CHN/18.11	C	A	C	A	A	T	C	A	T	C	T	G	C
1	MuVi/Yunnan.CHN/47.10/2	C	A	C	A	A	T	C	A	T	C	T	G	C
1	MuVi/Jilin.CHN/15.08/3	C	A	C	A	A	T	C	A	T	C	T	G	C
1	MuVi/Jilin.CHN/15.08/1	C	A	C	A	A	T	C	A	T	C	T	G	C
1	MuVi/Jilin.CHN/15.08/5	C	A	C	A	A	T	C	A	T	C	T	G	C
4	MuVi/Shannxi.CHN/26.09/1	C	A	C	A	A	T	C	A	T	C	T	G	C
4	MuVi/Jiangsu.CHN/15.12	C	A	C	A	A	T	C	A	T	C	T	G	C
3	MuVi/Shandong.CHN/4.05	C	G	C	A	A	T	C	A	T	C	T	G	C
3	MuVi/Shannxi.CHN/9.09/2	A	G	C	A	A	T	C	A	T	C	T	G	C
3	MuVi/Shanxi.CHN/52.10/3	C	G	C	A	A	T	C	A	T	C	T	G	C
3	MuVi/Jiangsu.CHN/3.13/1	C	G	C	A	A	T	C	A	T	C	T	G	C
3	MuVi/Henan.CHN/48.06	C	G	C	A	A	T	C	A	T	C	T	G	C
3	MuVi/Jiangsu.CHN/9.12	C	G	C	A	A	T	C	A	T	C	T	G	C
3	MuVi/Jiangsu.CHN/4.13/3	C	G	C	A	A	T	C	A	T	C	T	G	C
2	MuVi/Shanxi.CHN/52.10/2	C	A	C	A	A	T	C	A	T	C	T	G	C
2	MuVi/Shannxi.CHN/26.09/4	C	A	C	A	A	T	C	A	T	C	T	G	C
2	MuVi/Shanxi.CHN/52.10/1	C	A	C	A	A	T	C	A	T	C	T	T	C
2	MuVi/Guangdong.CHN/21.09/1	C	A	C	A	G	T	C	A	T	C	T	G	C
2	MuVi/Heilongjiang.CHN/49.15	C	A	C	A	A	T	C	A	T	C	T	G	C
2	MuVi/Anhui.CHN/10.11/1	C	A	C	A	A	T	C	A	T	C	T	G	C
2	MuVi/Shannxi.CHN/20.12	C	A	C	A	A	T	C	A	T	C	T	G	C
2	MuVi/Liaoning.CHN/48.10/2	C	A	C	A	A	T	C	A	T	C	T	G	C
2	MuVi/Shandong.CHN/51.15/1	C	A	C	A	A	T	C	A	T	C	T	G	C
	MuVi/Hunan.CHN/32.11	C	A	C	A	A	T	C	A	T	C	T	G	T
	MuVi/Hunan.CHN/30.11/2	C	A	C	A	A	T	C	A	T	C	T	G	C
	MuVi/Heilongjiang.CHN/14.13/1	C	A	T	A	A	T	C	A	T	C	T	A	C
	MuVi/Jiangsu.CHN/7.12	C	A	C	A	A	T	C	A	T	C	T	A	C
	MuVi/Liaoning.CHN/9.10/1	C	A	C	A	A	T	C	A	T	C	T	A	C
	MuVi/Hunan.CHN/30.11/3	C	A	C	A	A	T	C	A	T	C	T	A	C
	MuVi/Jiangsu.CHN/12.13/2	C	A	C	A	A	T	C	A	T	C	T	G	C
	MuVi/Shannxi.CHN/20.15/1	C	G	C	A	A	T	C	A	T	C	T	G	C
	MuVi/Liaoning.CHN/13.12	C	A	C	A	A	T	C	A	T	C	T	G	C
	MuVi/Beijing.CHN/21.11	C	A	C	A	A	T	C	A	T	C	T	G	C
	MuVi/Liaoning.CHN/11.12	C	A	C	A	A	T	C	A	T	C	T	G	C
	MuVi/Jiangsu.CHN/4.13/5	C	A	C	A	A	T	C	A	T	C	T	G	C
	MuVi/Heilongjiang.CHN/26.12/1	C	A	C	A	A	T	C	A	T	C	T	G	C
	MuVi/Beijing.CHN/17.07/2	C	A	C	A	A	T	C	A	T	C	T	G	C
	MuVi/Sichuan.CHN/23.12/2	C	A	C	A	A	T	C	A	T	C	T	G	C
	MuVi/Hubei.CHN/44.12/1	C	A	C	A	A	T	C	A	T	C	T	G	C
	MuVi/Jiangsu.CHN/2.13/4	C	A	C	A	A	T	C	A	T	C	T	G	C
	MuVi/Liaoning.CHN/7.12	C	A	C	A	A	T	C	A	T	C	T	G	C
	MuVi/Heilongjiang.CHN/22.12	C	A	C	A	A	T	C	A	T	C	T	G	C
	MuVi/Heilongjiang.CHN/15.13	C	A	C	G	A	T	C	A	T	C	T	G	C
	MuVi/Liaoning.CHN/5.09/1	C	A	C	A	A	T	C	A	T	C	T	G	C
	MuVi/Liaoning.CHN/1.08/2	C	A	C	A	A	T	C	A	T	C	T	G	C
	MuVi/Liaoning.CHN/10.09	C	A	C	A	A	T	C	A	T	C	T	G	C
	MuVi/Liaoning.CHN/1.08/1	C	A	C	A	A	T	C	A	T	C	T	G	C
	MuVi/Liaoning.CHN/48.10/1	C	A	C	A	A	T	C	A	T	C	T	G	C
	MuVi/Beijing.CHN/10.07	C	A	C	A	A	T	C	A	T	C	T	G	C
	MuVi/Hunan.CHN/30.11/1	C	A	C	A	A	T	C	A	T	C	T	G	C
	MuVi/Jiangsu.CHN/7.13/1	C	A	C	A	A	T	C	A	T	C	T	G	T
	MuVi/Liaoning.CHN/5.09/2	C	A	C	A	A	T	C	A	T	C	T	G	C
	MuVi/Zhejiang.CHN/11.06/1	C	A	C	A	A	T	C	A	T	C	T	G	C

1: Lineage based on Figure 1
2: SNPs are noted in green.
3: SNP position corresponding to a lineage-defining node in Figure 1

Supplementary Table S3: Listing of SNPs featured in the 70 F sequences of [illegible]

Lineage [1]	Strain name	283	285	288	297	300	303	304	324	339	348	351	354	360
1	MuVi/Liaoning.CHN/50.11	C	T	G	T	T	A	A	C	C	G	C	A	A
1	MuVi/Liaoning.CHN/48.11	C	T	G	T	T	A	A	C	C	G	C	A	A
1	MuVi/Heilongjiang.CHN/23.13/1	C	T	G	T	T	A	A	C	C	G	C	A	A
1	MuVi/Shandong.CHN/43.07	C	T	G	T	T	A	A	C	C	G	C	A	A
1	MuVi/Shandong.CHN/10.08	C	T	G	T	T	A	A	C	C	G	C	A	A
1	MuVi/Heilongjiang.CHN/26.12/2	C	T	G	T	T	A	A	C	C	G	C	A	G
1	MuVi/Heilongjiang.CHN/24.14	C	T	G	T	T	A	A	C	C	G	C	A	A
1	MuVi/Sichuan.CHN/24.15	C	T	G	T	T	A	A	C	C	G	C	A	A
1	MuVi/Shandong.CHN/11.07	C	T	G	T	T	A	A	C	C	G	C	A	A
1	MuVi/Beijing.CHN/15.06	C	T	G	T	T	A	A	C	C	G	C	A	A
1	MuVi/Gansu.CHN/0.02	C	T	G	T	T	A	A	C	C	G	C	A	A
1	MuVi/Jiangsu.CHN/3.13/2	C	T	G	T	T	A	A	C	C	G	C	A	A
1	MuVi/Beijing.CHN/25.06	C	T	G	T	T	A	A	C	C	G	C	A	A
1	MuVi/Shanghai.CHN/0.01	C	T	G	T	T	G	A	C	C	G	C	A	A
1	MuVi/Shandong.CHN/3.05	C	T	G	T	T	A	A	C	C	G	C	A	A
1	MuVi/Zhejiang.CHN/26.05	C	T	G	T	T	A	A	C	C	G	C	A	A
1	MuVi/Sichuan.CHN/23.12/1	C	T	G	T	T	A	A	C	C	G	C	A	A
1	MuVi/Neimeng.CHN/18.11	C	T	G	C	T	A	A	C	C	G	C	A	A
1	MuVi/Yunnan.CHN/47.10/2	C	T	G	T	T	A	A	C	C	G	C	A	A
1	MuVi/Jilin.CHN/15.08/3	C	T	G	T	T	A	A	C	C	G	C	A	A
1	MuVi/Jilin.CHN/15.08/1	C	T	G	T	T	A	A	C	C	G	C	A	A
1	MuVi/Jilin.CHN/15.08/5	C	T	G	T	T	A	A	C	C	G	C	A	A
4	MuVi/Shannxi.CHN/26.09/1	C	T	G	T	T	A	A	C	C	G	C	A	A
4	MuVi/Jiangsu.CHN/15.12	C	T	G	T	T	A	A	C	C	G	C	A	A
3	MuVi/Shandong.CHN/4.05	C	T	G	T	T	A	A	C	C	G	C	A	A
3	MuVi/Shannxi.CHN/9.09/2	C	T	G	T	T	A	A	C	C	G	C	A	A
3	MuVi/Shanxi.CHN/52.10/3	C	T	G	T	T	A	A	C	C	G	C	A	A
3	MuVi/Jiangsu.CHN/3.13/1	C	T	G	T	T	A	A	C	C	G	C	A	A
3	MuVi/Henan.CHN/48.06	C	T	G	T	T	A	A	C	C	G	C	A	A
3	MuVi/Jiangsu.CHN/9.12	C	T	G	T	T	A	A	C	C	G	C	A	A
3	MuVi/Jiangsu.CHN/4.13/3	C	T	G	T	T	A	A	C	C	G	C	A	A
2	MuVi/Shanxi.CHN/52.10/2	C	T	G	T	C	A	A	C	C	G	C	A	A
2	MuVi/Shannxi.CHN/26.09/4	C	T	G	T	C	A	A	C	C	G	C	A	A
2	MuVi/Shanxi.CHN/52.10/1	C	T	G	T	C	A	A	C	C	G	C	A	A
2	MuVi/Guangdong.CHN/21.09/1	C	T	G	T	C	A	C	C	C	G	C	A	A
2	MuVi/Heilongjiang.CHN/49.15	C	A	A	T	C	A	A	C	C	A	T	A	A
2	MuVi/Anhui.CHN/10.11/1	C	A	G	T	C	A	A	C	C	A	C	A	A
2	MuVi/Shannxi.CHN/20.12	C	A	G	T	C	A	A	C	C	A	C	A	A
2	MuVi/Liaoning.CHN/48.10/2	C	A	G	T	C	A	A	C	C	A	C	A	A
2	MuVi/Shandong.CHN/51.15/1	C	A	G	T	C	A	A	C	C	A	C	A	A
	MuVi/Hunan.CHN/32.11	C	T	G	T	T	A	A	C	C	G	C	A	A
	MuVi/Hunan.CHN/30.11/2	C	T	A	T	T	A	A	C	C	G	C	A	A
	MuVi/Heilongjiang.CHN/14.13/1	C	C	G	T	T	A	A	C	C	G	C	A	A
	MuVi/Jiangsu.CHN/7.12	C	T	G	T	T	A	A	C	C	G	C	A	A
	MuVi/Liaoning.CHN/9.10/1	C	T	G	T	T	A	A	C	C	G	C	A	A
	MuVi/Hunan.CHN/30.11/3	C	T	G	T	T	A	A	C	C	G	C	A	A
	MuVi/Jiangsu.CHN/12.13/2	C	T	G	T	T	A	A	C	C	G	C	A	A
	MuVi/Shannxi.CHN/20.15/1	C	T	G	T	T	A	A	C	C	G	C	C	A
	MuVi/Liaoning.CHN/13.12	T	T	G	T	C	A	A	C	C	G	C	A	A
	MuVi/Beijing.CHN/21.11	C	T	G	T	T	A	A	C	C	G	C	A	A
	MuVi/Liaoning.CHN/11.12	C	T	G	T	T	A	A	C	C	G	C	A	A
	MuVi/Jiangsu.CHN/4.13/5	C	T	G	T	T	A	A	C	C	G	C	A	A
	MuVi/Heilongjiang.CHN/26.12/1	C	T	G	T	T	A	A	C	C	G	C	A	A
	MuVi/Beijing.CHN/17.07/2	C	T	G	T	T	A	A	C	C	G	C	A	A
	MuVi/Sichuan.CHN/23.12/2	C	T	G	T	T	A	A	C	C	G	C	A	A
	MuVi/Hubei.CHN/44.12/1	C	T	G	T	T	A	A	C	C	G	C	A	A
	MuVi/Jiangsu.CHN/2.13/4	C	T	G	T	T	A	A	C	C	G	C	A	A
	MuVi/Liaoning.CHN/7.12	C	T	G	T	T	A	A	C	C	G	C	A	A
	MuVi/Heilongjiang.CHN/22.12	C	T	G	T	T	A	A	C	C	G	C	A	A
	MuVi/Heilongjiang.CHN/15.13	C	T	G	T	T	A	A	C	C	G	C	A	A
	MuVi/Liaoning.CHN/5.09/1	C	T	G	T	T	A	A	C	C	G	C	A	A
	MuVi/Liaoning.CHN/1.08/2	C	T	G	T	T	A	A	C	C	G	C	A	A
	MuVi/Liaoning.CHN/10.09	C	T	G	T	T	A	A	C	C	G	C	A	A
	MuVi/Liaoning.CHN/1.08/1	C	T	G	T	T	A	A	C	C	G	C	A	A
	MuVi/Liaoning.CHN/48.10/1	C	T	G	T	T	A	A	C	C	G	C	A	A
	MuVi/Beijing.CHN/10.07	C	T	G	T	T	A	A	T	C	G	C	A	A
	MuVi/Hunan.CHN/30.11/1	C	T	G	T	T	A	A	T	C	G	C	A	A
	MuVi/Jiangsu.CHN/7.13/1	C	T	G	T	T	A	A	C	C	G	C	A	A
	MuVi/Liaoning.CHN/5.09/2	C	T	G	T	T	A	A	C	T	G	C	A	A
	MuVi/Zhejiang.CHN/11.06/1	C	T	G	T	T	A	A	C	C	G	C	A	A

1: Lineage based on Figure 1
2: SNPs are noted in green.
3: SNP position corresponding to a lineage-defining node in Figure 1 [illegible]

Supplementary Table S3: Listing of SNPs featured in the 70 F sequences of [illegible]

Lineage [1]	Strain name	361	363	369	375	384	387	393	414	416	422	423	426	435
1	MuVi/Liaoning.CHN/50.11	G	A	C	C	C	A	G	G	C	A	A	T	G
1	MuVi/Liaoning.CHN/48.11	G	A	C	C	C	A	G	G	C	A	A	T	G
1	MuVi/Heilongjiang.CHN/23.13/1	G	A	C	C	C	A	G	G	C	A	A	T	G
1	MuVi/Shandong.CHN/43.07	G	A	C	C	C	A	G	G	C	A	A	T	G
1	MuVi/Shandong.CHN/10.08	G	A	C	C	C	A	G	G	C	A	A	T	G
1	MuVi/Heilongjiang.CHN/26.12/2	G	A	C	C	C	A	G	G	C	A	A	T	G
1	MuVi/Heilongjiang.CHN/24.14	G	A	C	C	C	A	G	G	C	A	A	T	G
1	MuVi/Sichuan.CHN/24.15	G	A	C	C	C	A	G	G	C	A	A	T	G
1	MuVi/Shandong.CHN/11.07	G	A	C	C	T	A	G	G	C	A	A	T	G
1	MuVi/Beijing.CHN/15.06	G	A	C	C	T	A	G	G	C	A	A	T	G
1	MuVi/Gansu.CHN/0.02	G	A	C	C	T	A	G	G	C	A	A	T	G
1	MuVi/Jiangsu.CHN/3.13/2	G	A	C	C	T	A	G	G	C	A	A	T	G
1	MuVi/Beijing.CHN/25.06	G	A	C	C	T	A	G	G	C	G	A	T	G
1	MuVi/Shanghai.CHN/0.01	G	A	C	C	T	A	G	A	C	A	A	T	G
1	MuVi/Shandong.CHN/3.05	G	A	C	C	T	A	G	A	A	A	A	T	G
1	MuVi/Zhejiang.CHN/26.05	G	A	C	C	T	A	G	A	C	A	G	T	G
1	MuVi/Sichuan.CHN/23.12/1	G	A	C	C	T	A	G	G	C	A	G	T	G
1	MuVi/Neimeng.CHN/18.11	G	A	C	C	T	A	G	G	C	A	G	T	G
1	MuVi/Yunnan.CHN/47.10/2	G	A	C	C	T	A	G	G	C	A	G	T	G
1	MuVi/Jilin.CHN/15.08/3	G	A	C	C	T	A	G	G	C	A	G	T	G
1	MuVi/Jilin.CHN/15.08/1	G	A	C	C	T	A	G	G	C	A	G	T	G
1	MuVi/Jilin.CHN/15.08/5	G	A	C	C	T	A	G	G	C	A	G	T	G
4	MuVi/Shannxi.CHN/26.09/1	G	A	C	C	T	A	G	G	C	A	A	C	G
4	MuVi/Jiangsu.CHN/15.12	G	A	C	C	T	A	G	G	C	A	A	C	G
3	MuVi/Shandong.CHN/4.05	G	A	C	C	T	A	G	G	C	A	A	T	G
3	MuVi/Shannxi.CHN/9.09/2	G	A	C	C	T	A	G	G	C	A	A	T	A
3	MuVi/Shanxi.CHN/52.10/3	G	A	C	C	T	A	G	G	C	A	A	T	G
3	MuVi/Jiangsu.CHN/3.13/1	G	A	C	C	T	A	G	G	C	A	A	T	G
3	MuVi/Henan.CHN/48.06	G	A	C	C	T	A	G	G	C	A	A	T	G
3	MuVi/Jiangsu.CHN/9.12	G	A	C	C	T	A	G	G	C	A	A	T	G
3	MuVi/Jiangsu.CHN/4.13/3	G	A	C	C	T	A	G	G	C	A	A	T	G
2	MuVi/Shanxi.CHN/52.10/2	G	A	C	C	T	A	G	G	C	A	A	T	G
2	MuVi/Shannxi.CHN/26.09/4	G	A	C	C	T	A	G	G	C	A	A	T	G
2	MuVi/Shanxi.CHN/52.10/1	G	A	C	C	T	A	G	G	C	A	A	T	G
2	MuVi/Guangdong.CHN/21.09/1	G	A	C	C	T	A	G	G	C	A	A	T	G
2	MuVi/Heilongjiang.CHN/49.15	G	A	C	C	T	A	G	G	C	A	A	T	G
2	MuVi/Anhui.CHN/10.11/1	G	A	C	C	T	A	G	G	C	A	A	T	G
2	MuVi/Shannxi.CHN/20.12	G	A	C	C	T	A	G	G	C	A	A	T	G
2	MuVi/Liaoning.CHN/48.10/2	G	A	C	C	T	A	G	G	C	A	A	T	G
2	MuVi/Shandong.CHN/51.15/1	G	A	C	C	T	A	G	A	C	A	A	T	G
	MuVi/Hunan.CHN/32.11	G	A	C	C	T	A	G	G	C	A	A	T	G
	MuVi/Hunan.CHN/30.11/2	G	A	C	C	T	A	G	G	C	A	A	T	G
	MuVi/Heilongjiang.CHN/14.13/1	G	A	C	C	T	A	G	G	C	A	A	T	G
	MuVi/Jiangsu.CHN/7.12	G	A	C	C	T	A	G	G	C	A	A	T	G
	MuVi/Liaoning.CHN/9.10/1	G	A	C	C	T	A	G	G	C	A	A	T	G
	MuVi/Hunan.CHN/30.11/3	G	A	C	C	T	A	G	G	C	A	A	T	A
	MuVi/Jiangsu.CHN/12.13/2	G	A	C	C	T	A	G	G	C	A	A	T	G
	MuVi/Shannxi.CHN/20.15/1	G	A	C	C	T	A	G	G	C	A	A	T	G
	MuVi/Liaoning.CHN/13.12	G	A	C	C	T	A	G	G	C	A	A	T	G
	MuVi/Beijing.CHN/21.11	A	A	C	C	T	A	G	G	C	A	A	T	G
	MuVi/Liaoning.CHN/11.12	A	A	C	C	T	A	G	G	C	A	A	T	G
	MuVi/Jiangsu.CHN/4.13/5	G	A	A	C	T	A	A	G	C	A	A	T	G
	MuVi/Heilongjiang.CHN/26.12/1	G	A	C	C	T	A	G	G	C	A	A	T	G
	MuVi/Beijing.CHN/17.07/2	G	A	C	C	T	A	G	G	C	A	A	T	G
	MuVi/Sichuan.CHN/23.12/2	G	A	C	C	T	A	G	G	C	A	A	T	G
	MuVi/Hubei.CHN/44.12/1	G	A	C	C	T	A	G	G	C	A	A	T	G
	MuVi/Jiangsu.CHN/2.13/4	G	A	C	C	T	A	G	G	C	A	A	T	G
	MuVi/Liaoning.CHN/7.12	G	A	C	C	T	A	G	G	C	A	A	T	G
	MuVi/Heilongjiang.CHN/22.12	G	A	C	C	T	A	G	G	C	A	A	T	G
	MuVi/Heilongjiang.CHN/15.13	G	A	C	C	T	G	G	G	C	A	A	T	G
	MuVi/Liaoning.CHN/5.09/1	G	A	C	C	T	A	G	G	C	A	A	T	G
	MuVi/Liaoning.CHN/1.08/2	G	A	C	C	T	A	G	G	C	A	A	T	G
	MuVi/Liaoning.CHN/10.09	G	A	C	C	T	A	G	G	C	A	A	T	G
	MuVi/Liaoning.CHN/1.08/1	G	A	C	C	T	A	G	G	C	A	A	T	G
	MuVi/Liaoning.CHN/48.10/1	G	A	C	C	T	A	G	G	C	A	A	T	G
	MuVi/Beijing.CHN/10.07	G	A	C	A	T	A	G	G	C	A	A	T	G
	MuVi/Hunan.CHN/30.11/1	G	A	C	C	T	A	G	G	C	A	A	T	G
	MuVi/Jiangsu.CHN/7.13/1	G	A	C	C	T	A	G	G	C	A	A	T	G
	MuVi/Liaoning.CHN/5.09/2	G	A	C	C	T	A	G	A	C	A	A	T	G
	MuVi/Zhejiang.CHN/11.06/1	G	G	C	C	T	A	G	G	C	A	A	T	G

1: Lineage based on Figure 1
2: SNPs are noted in green.
3: SNP position corresponding to a lineage-defining node in Figure 1 [illegible]

Supplementary Table S3: Listing of SNPs featured in the 70 F sequences of [illegible]

Lineage [1]	Strain name	441	451	456	459	468	474	489	507	513	519	531	534	546
1	MuVi/Liaoning.CHN/50.11	T	A	C	A	A	T	A	A	C	T	C	T	T
1	MuVi/Liaoning.CHN/48.11	T	A	C	A	A	T	A	A	C	T	C	T	T
1	MuVi/Heilongjiang.CHN/23.13/1	T	A	C	A	A	T	A	A	C	T	C	T	T
1	MuVi/Shandong.CHN/43.07	T	A	C	A	A	T	A	A	C	T	C	T	T
1	MuVi/Shandong.CHN/10.08	T	A	C	A	A	T	A	A	C	T	C	T	T
1	MuVi/Heilongjiang.CHN/26.12/2	T	A	C	A	A	T	A	A	C	T	C	T	T
1	MuVi/Heilongjiang.CHN/24.14	T	A	C	A	A	T	A	A	C	T	C	T	T
1	MuVi/Sichuan.CHN/24.15	T	A	C	A	A	T	A	A	C	T	C	T	T
1	MuVi/Shandong.CHN/11.07	T	A	C	A	A	T	A	A	C	T	C	T	C
1	MuVi/Beijing.CHN/15.06	T	A	C	A	A	T	A	A	C	T	C	T	C
1	MuVi/Gansu.CHN/0.02	T	A	C	A	A	T	A	A	C	T	C	T	T
1	MuVi/Jiangsu.CHN/3.13/2	T	A	C	A	A	T	A	A	C	T	C	T	T
1	MuVi/Beijing.CHN/25.06	T	A	C	A	A	T	A	A	C	T	C	T	T
1	MuVi/Shanghai.CHN/0.01	T	A	C	A	A	T	A	A	C	T	C	T	T
1	MuVi/Shandong.CHN/3.05	T	A	C	A	A	T	A	A	C	T	C	T	T
1	MuVi/Zhejiang.CHN/26.05	T	A	C	A	A	T	A	A	C	T	C	T	T
1	MuVi/Sichuan.CHN/23.12/1	T	A	C	A	A	T	A	A	C	T	C	T	T
1	MuVi/Neimeng.CHN/18.11	T	A	C	A	A	T	A	A	C	T	C	T	T
1	MuVi/Yunnan.CHN/47.10/2	T	A	C	A	A	T	A	A	C	T	C	T	T
1	MuVi/Jilin.CHN/15.08/3	T	A	C	A	A	T	A	A	C	T	C	T	T
1	MuVi/Jilin.CHN/15.08/1	T	A	C	A	A	T	A	A	C	T	C	T	T
1	MuVi/Jilin.CHN/15.08/5	T	A	C	A	A	T	A	A	C	T	C	T	T
4	MuVi/Shannxi.CHN/26.09/1	T	G	C	A	A	T	A	A	C	T	C	T	T
4	MuVi/Jiangsu.CHN/15.12	T	G	C	A	A	T	A	A	C	T	C	T	T
3	MuVi/Shandong.CHN/4.05	T	G	C	A	G	T	A	A	C	T	C	T	T
3	MuVi/Shannxi.CHN/9.09/2	T	G	C	A	A	T	A	A	T	T	C	T	T
3	MuVi/Shanxi.CHN/52.10/3	T	G	C	A	A	T	A	A	C	T	C	T	T
3	MuVi/Jiangsu.CHN/3.13/1	T	G	C	A	A	T	A	A	C	T	C	T	T
3	MuVi/Henan.CHN/48.06	T	G	C	A	A	T	A	A	C	T	C	T	T
3	MuVi/Jiangsu.CHN/9.12	T	G	C	A	A	T	A	A	C	T	C	T	T
3	MuVi/Jiangsu.CHN/4.13/3	T	G	C	A	A	T	A	A	C	T	C	T	T
2	MuVi/Shanxi.CHN/52.10/2	T	G	C	A	A	T	A	A	C	C	C	T	T
2	MuVi/Shannxi.CHN/26.09/4	T	G	C	A	A	T	A	A	C	T	C	T	T
2	MuVi/Shanxi.CHN/52.10/1	T	G	C	A	A	T	A	A	C	T	C	T	T
2	MuVi/Guangdong.CHN/21.09/1	T	G	C	A	A	T	A	A	C	T	C	T	T
2	MuVi/Heilongjiang.CHN/49.15	C	G	C	A	A	T	A	A	C	T	C	T	T
2	MuVi/Anhui.CHN/10.11/1	T	G	C	A	A	T	A	A	C	T	C	T	T
2	MuVi/Shannxi.CHN/20.12	T	G	C	A	A	T	A	A	C	T	C	T	T
2	MuVi/Liaoning.CHN/48.10/2	T	G	C	A	A	T	A	A	C	T	C	T	T
2	MuVi/Shandong.CHN/51.15/1	T	G	C	A	A	T	A	A	C	T	C	T	T
	MuVi/Hunan.CHN/32.11	T	G	C	A	A	C	A	A	C	T	C	T	T
	MuVi/Hunan.CHN/30.11/2	T	G	C	A	A	T	A	A	C	T	C	T	T
	MuVi/Heilongjiang.CHN/14.13/1	T	G	C	A	A	T	A	A	C	T	C	T	T
	MuVi/Jiangsu.CHN/7.12	T	G	C	G	A	T	A	A	C	T	C	T	T
	MuVi/Liaoning.CHN/9.10/1	T	G	C	A	A	T	A	A	C	T	C	T	T
	MuVi/Hunan.CHN/30.11/3	T	G	C	A	A	T	A	A	C	T	C	T	T
	MuVi/Jiangsu.CHN/12.13/2	T	G	T	A	A	T	C	A	C	T	C	T	T
	MuVi/Shannxi.CHN/20.15/1	T	G	C	A	A	T	A	A	C	T	C	T	T
	MuVi/Liaoning.CHN/13.12	T	G	C	A	A	T	A	A	C	T	C	T	T
	MuVi/Beijing.CHN/21.11	T	G	C	A	A	T	A	A	C	T	C	T	T
	MuVi/Liaoning.CHN/11.12	T	G	C	A	A	T	A	A	C	T	C	T	T
	MuVi/Jiangsu.CHN/4.13/5	T	G	C	A	A	T	A	A	C	T	C	T	T
	MuVi/Heilongjiang.CHN/26.12/1	T	G	C	A	A	T	A	A	C	T	T	T	T
	MuVi/Beijing.CHN/17.07/2	T	G	C	A	A	T	A	A	C	T	C	G	T
	MuVi/Sichuan.CHN/23.12/2	C	G	C	A	A	T	A	A	C	T	C	T	T
	MuVi/Hubei.CHN/44.12/1	T	G	C	A	A	T	A	A	C	T	C	T	T
	MuVi/Jiangsu.CHN/2.13/4	T	G	T	A	A	T	A	A	C	T	C	T	T
	MuVi/Liaoning.CHN/7.12	T	G	C	A	A	T	A	A	C	T	C	T	T
	MuVi/Heilongjiang.CHN/22.12	T	G	C	A	A	T	A	A	C	T	C	T	T
	MuVi/Heilongjiang.CHN/15.13	T	G	C	A	A	T	A	A	C	T	C	T	T
	MuVi/Liaoning.CHN/5.09/1	T	G	C	A	A	T	A	A	C	T	C	T	T
	MuVi/Liaoning.CHN/1.08/2	T	G	C	A	A	T	A	A	C	T	C	T	T
	MuVi/Liaoning.CHN/10.09	T	G	C	A	A	T	A	A	C	T	C	T	T
	MuVi/Liaoning.CHN/1.08/1	T	G	C	A	A	T	A	A	C	T	C	T	T
	MuVi/Liaoning.CHN/48.10/1	T	G	C	A	A	T	A	A	C	T	C	T	T
	MuVi/Beijing.CHN/10.07	T	G	C	A	A	T	A	A	C	T	C	T	T
	MuVi/Hunan.CHN/30.11/1	T	G	C	A	A	T	A	G	C	T	C	T	T
	MuVi/Jiangsu.CHN/7.13/1	T	G	C	A	A	T	A	G	C	T	C	T	T
	MuVi/Liaoning.CHN/5.09/2	T	G	C	A	A	T	A	A	C	T	C	T	T
	MuVi/Zhejiang.CHN/11.06/1	T	G	C	A	A	T	A	A	C	T	C	T	T

1: Lineage based on Figure 1
2: SNPs are noted in green.
3: SNP position corresponding to a lineage-defining node in Figure 1 [illegible]

Supplementary Table S3: Listing of SNPs featured in the 70 F sequences of [illegible]

Lineage [1]	Strain name	561	564	570	572	573	579	584	585	597	600	603	606	621
1	MuVi/Liaoning.CHN/50.11	C	T	C	A	G	G	C	C	C	A	A	A	T
1	MuVi/Liaoning.CHN/48.11	C	T	C	A	G	G	C	C	C	A	A	A	T
1	MuVi/Heilongjiang.CHN/23.13/1	C	T	C	A	G	G	C	C	C	A	A	A	T
1	MuVi/Shandong.CHN/43.07	C	T	C	A	G	G	C	C	C	A	A	A	T
1	MuVi/Shandong.CHN/10.08	C	T	C	A	G	G	C	C	C	A	A	A	T
1	MuVi/Heilongjiang.CHN/26.12/2	C	T	C	A	G	G	C	C	C	A	A	A	T
1	MuVi/Heilongjiang.CHN/24.14	C	T	C	A	G	G	C	C	C	A	A	A	T
1	MuVi/Sichuan.CHN/24.15	C	T	C	A	G	G	C	C	C	A	A	A	T
1	MuVi/Shandong.CHN/11.07	C	T	C	A	G	G	C	C	C	A	A	A	T
1	MuVi/Beijing.CHN/15.06	C	T	C	A	G	G	C	C	C	A	A	A	T
1	MuVi/Gansu.CHN/0.02	C	T	C	A	G	G	C	C	C	A	A	A	T
1	MuVi/Jiangsu.CHN/3.13/2	C	T	C	A	G	G	C	C	C	A	A	A	T
1	MuVi/Beijing.CHN/25.06	C	T	C	A	G	G	C	C	C	A	A	A	T
1	MuVi/Shanghai.CHN/0.01	C	T	C	A	A	G	C	C	C	A	A	G	T
1	MuVi/Shandong.CHN/3.05	C	T	C	A	G	A	C	C	C	A	A	A	T
1	MuVi/Zhejiang.CHN/26.05	C	T	C	A	G	G	C	C	C	A	A	A	T
1	MuVi/Sichuan.CHN/23.12/1	C	T	C	A	G	G	C	C	C	A	A	G	T
1	MuVi/Neimeng.CHN/18.11	C	T	C	A	G	G	C	C	C	A	A	G	T
1	MuVi/Yunnan.CHN/47.10/2	C	T	C	G	G	G	C	C	C	A	A	G	T
1	MuVi/Jilin.CHN/15.08/3	C	T	C	A	G	G	T	C	C	A	A	G	T
1	MuVi/Jilin.CHN/15.08/1	C	T	C	A	G	G	C	C	C	A	A	G	T
1	MuVi/Jilin.CHN/15.08/5	C	T	C	A	G	G	C	C	C	A	A	G	T
4	MuVi/Shannxi.CHN/26.09/1	C	T	C	A	G	G	C	C	C	A	A	A	T
4	MuVi/Jiangsu.CHN/15.12	C	T	C	A	G	G	C	C	C	A	A	A	T
3	MuVi/Shandong.CHN/4.05	C	T	C	A	G	G	C	C	C	A	A	A	T
3	MuVi/Shannxi.CHN/9.09/2	C	T	C	A	G	G	C	C	C	A	A	A	T
3	MuVi/Shanxi.CHN/52.10/3	C	T	C	A	G	G	C	C	C	A	A	A	T
3	MuVi/Jiangsu.CHN/3.13/1	C	T	C	A	G	G	C	C	C	A	A	A	T
3	MuVi/Henan.CHN/48.06	C	T	C	A	G	G	C	C	C	A	A	A	T
3	MuVi/Jiangsu.CHN/9.12	C	T	C	A	G	G	C	C	C	A	A	A	T
3	MuVi/Jiangsu.CHN/4.13/3	C	T	C	A	G	G	C	C	C	A	A	A	T
2	MuVi/Shanxi.CHN/52.10/2	C	C	C	A	G	G	C	C	C	A	A	A	T
2	MuVi/Shannxi.CHN/26.09/4	C	T	C	A	G	G	C	C	C	A	A	A	T
2	MuVi/Shanxi.CHN/52.10/1	C	T	C	A	G	G	C	C	C	A	A	A	T
2	MuVi/Guangdong.CHN/21.09/1	C	T	C	A	G	G	C	C	C	A	A	A	T
2	MuVi/Heilongjiang.CHN/49.15	C	T	C	A	G	G	C	C	C	A	A	A	T
2	MuVi/Anhui.CHN/10.11/1	C	T	T	A	G	G	C	C	C	A	A	A	T
2	MuVi/Shannxi.CHN/20.12	C	T	T	A	G	G	C	C	C	A	A	A	T
2	MuVi/Liaoning.CHN/48.10/2	C	T	C	A	G	G	C	C	C	T	A	A	T
2	MuVi/Shandong.CHN/51.15/1	C	T	C	A	G	G	C	C	C	T	A	A	T
	MuVi/Hunan.CHN/32.11	C	T	C	A	G	G	C	C	C	A	A	A	T
	MuVi/Hunan.CHN/30.11/2	C	T	C	A	G	G	C	C	C	A	A	A	C
	MuVi/Heilongjiang.CHN/14.13/1	C	T	C	A	G	G	C	C	C	A	A	A	T
	MuVi/Jiangsu.CHN/7.12	C	T	C	A	G	G	C	C	C	A	A	A	T
	MuVi/Liaoning.CHN/9.10/1	C	T	C	A	G	G	C	C	C	A	A	A	T
	MuVi/Hunan.CHN/30.11/3	C	T	C	A	G	G	C	C	C	A	A	A	T
	MuVi/Jiangsu.CHN/12.13/2	C	T	C	A	G	G	C	C	T	A	A	A	T
	MuVi/Shannxi.CHN/20.15/1	C	T	C	A	G	G	C	C	C	A	A	A	T
	MuVi/Liaoning.CHN/13.12	C	T	C	A	G	G	C	C	C	A	A	A	T
	MuVi/Beijing.CHN/21.11	C	T	C	A	G	G	C	C	C	A	C	A	T
	MuVi/Liaoning.CHN/11.12	C	T	C	A	G	G	C	C	C	A	C	A	T
	MuVi/Jiangsu.CHN/4.13/5	C	T	C	A	G	G	C	C	C	A	A	A	T
	MuVi/Heilongjiang.CHN/26.12/1	C	T	C	A	G	G	C	C	C	A	A	A	T
	MuVi/Beijing.CHN/17.07/2	C	T	C	A	G	G	C	C	C	A	A	A	T
	MuVi/Sichuan.CHN/23.12/2	C	T	C	A	G	G	C	C	C	A	A	A	T
	MuVi/Hubei.CHN/44.12/1	C	T	C	A	G	G	C	C	C	A	A	A	T
	MuVi/Jiangsu.CHN/2.13/4	C	T	C	A	G	G	C	C	C	A	A	A	T
	MuVi/Liaoning.CHN/7.12	C	T	C	A	G	G	C	C	C	A	A	A	T
	MuVi/Heilongjiang.CHN/22.12	C	T	C	A	G	G	C	C	C	A	A	A	T
	MuVi/Heilongjiang.CHN/15.13	C	T	C	A	G	G	C	C	C	A	A	A	T
	MuVi/Liaoning.CHN/5.09/1	C	T	C	A	G	G	C	C	C	A	A	A	T
	MuVi/Liaoning.CHN/1.08/2	C	T	C	A	G	G	C	C	C	A	A	A	T
	MuVi/Liaoning.CHN/10.09	C	T	C	A	G	G	C	C	C	A	A	A	T
	MuVi/Liaoning.CHN/1.08/1	C	T	C	A	G	G	T	C	C	A	A	A	T
	MuVi/Liaoning.CHN/48.10/1	C	T	C	A	G	G	T	C	C	A	A	A	T
	MuVi/Beijing.CHN/10.07	C	T	C	A	G	G	C	T	C	A	A	A	T
	MuVi/Hunan.CHN/30.11/1	C	T	C	A	G	G	C	C	C	A	A	A	T
	MuVi/Jiangsu.CHN/7.13/1	C	T	C	A	G	G	C	C	C	A	A	A	T
	MuVi/Liaoning.CHN/5.09/2	A	T	C	A	G	G	C	C	C	A	A	A	T
	MuVi/Zhejiang.CHN/11.06/1	C	T	C	A	G	G	C	C	C	A	A	A	T

1: Lineage based on Figure 1
2: SNPs are noted in green.
3: SNP position corresponding to a lineage-defining node in Figure 1 [illegible]

Supplementary Table S3: Listing of SNPs featured in the 70 F sequences of [illegible]

Lineage [1]	Strain name	624	626	630	642	646	648	654	660	662	666	669	675	678
1	MuVi/Liaoning.CHN/50.11	G	C	A	G	C	G	T	T	T	A	C	G	T
1	MuVi/Liaoning.CHN/48.11	G	C	A	G	C	G	T	T	T	A	C	G	T
1	MuVi/Heilongjiang.CHN/23.13/1	G	C	A	G	C	G	T	T	T	A	C	G	T
1	MuVi/Shandong.CHN/43.07	G	C	A	G	C	G	T	T	T	A	C	G	T
1	MuVi/Shandong.CHN/10.08	G	C	A	G	C	G	T	T	T	A	C	G	T
1	MuVi/Heilongjiang.CHN/26.12/2	G	C	A	G	C	G	T	T	T	A	C	G	T
1	MuVi/Heilongjiang.CHN/24.14	G	C	A	G	C	G	T	T	T	A	C	G	T
1	MuVi/Sichuan.CHN/24.15	G	C	A	G	C	G	T	T	T	A	C	G	T
1	MuVi/Shandong.CHN/11.07	G	C	A	G	C	G	G	T	C	A	C	G	T
1	MuVi/Beijing.CHN/15.06	G	C	A	G	C	G	G	T	T	A	C	G	T
1	MuVi/Gansu.CHN/0.02	G	C	A	G	C	G	G	T	T	A	C	G	T
1	MuVi/Jiangsu.CHN/3.13/2	G	C	A	G	C	G	G	T	T	A	C	A	T
1	MuVi/Beijing.CHN/25.06	G	C	A	G	C	G	G	T	T	A	C	G	T
1	MuVi/Shanghai.CHN/0.01	G	C	A	G	C	G	G	T	T	A	C	G	T
1	MuVi/Shandong.CHN/3.05	G	C	A	G	C	G	G	T	T	A	C	G	T
1	MuVi/Zhejiang.CHN/26.05	G	C	A	G	C	G	G	T	T	A	C	G	T
1	MuVi/Sichuan.CHN/23.12/1	G	C	A	G	C	G	G	T	T	A	C	G	T
1	MuVi/Neimeng.CHN/18.11	G	C	A	G	C	G	G	T	T	A	C	G	T
1	MuVi/Yunnan.CHN/47.10/2	G	C	A	G	C	G	G	T	T	A	C	G	T
1	MuVi/Jilin.CHN/15.08/3	G	C	A	G	C	G	G	T	T	A	C	G	T
1	MuVi/Jilin.CHN/15.08/1	G	C	A	G	C	G	G	T	T	A	C	G	T
1	MuVi/Jilin.CHN/15.08/5	G	C	A	G	C	G	G	T	T	A	C	G	T
4	MuVi/Shannxi.CHN/26.09/1	G	C	A	G	C	G	G	T	T	A	C	G	T
4	MuVi/Jiangsu.CHN/15.12	G	C	A	G	C	G	G	T	T	A	C	G	T
3	MuVi/Shandong.CHN/4.05	G	T	A	G	C	G	G	T	T	A	C	G	T
3	MuVi/Shannxi.CHN/9.09/2	G	C	A	G	C	G	G	T	T	A	C	G	T
3	MuVi/Shanxi.CHN/52.10/3	G	C	A	G	C	G	G	T	T	A	C	G	T
3	MuVi/Jiangsu.CHN/3.13/1	G	C	A	G	C	G	G	T	T	A	C	G	T
3	MuVi/Henan.CHN/48.06	G	C	A	G	C	G	G	C	T	A	C	G	T
3	MuVi/Jiangsu.CHN/9.12	G	C	A	G	C	G	G	C	T	A	C	G	T
3	MuVi/Jiangsu.CHN/4.13/3	G	C	A	G	C	G	G	C	T	A	C	G	T
2	MuVi/Shanxi.CHN/52.10/2	G	C	A	G	C	G	G	T	T	A	C	G	T
2	MuVi/Shannxi.CHN/26.09/4	G	C	A	G	C	G	G	T	T	A	C	G	T
2	MuVi/Shanxi.CHN/52.10/1	A	C	A	A	C	G	G	T	T	A	C	G	T
2	MuVi/Guangdong.CHN/21.09/1	G	C	A	G	C	G	G	T	T	A	C	G	T
2	MuVi/Heilongjiang.CHN/49.15	G	C	A	G	C	G	G	T	T	A	C	G	T
2	MuVi/Anhui.CHN/10.11/1	G	C	A	G	C	G	T	T	T	A	C	G	T
2	MuVi/Shannxi.CHN/20.12	G	C	A	G	C	G	G	T	T	A	C	G	T
2	MuVi/Liaoning.CHN/48.10/2	G	C	A	G	C	G	G	T	T	A	C	G	T
2	MuVi/Shandong.CHN/51.15/1	G	C	A	G	C	G	G	T	T	A	C	G	T
	MuVi/Hunan.CHN/32.11	G	C	A	G	C	G	G	T	T	A	C	G	T
	MuVi/Hunan.CHN/30.11/2	G	C	A	G	C	G	G	T	T	A	C	G	T
	MuVi/Heilongjiang.CHN/14.13/1	G	C	A	G	C	G	G	T	T	A	C	G	T
	MuVi/Jiangsu.CHN/7.12	G	C	A	G	C	G	G	T	T	A	C	G	T
	MuVi/Liaoning.CHN/9.10/1	G	C	A	G	C	G	G	T	T	A	C	G	T
	MuVi/Hunan.CHN/30.11/3	G	C	G	G	C	G	G	T	T	A	C	G	T
	MuVi/Jiangsu.CHN/12.13/2	G	C	A	G	C	G	G	T	T	A	C	G	T
	MuVi/Shannxi.CHN/20.15/1	G	C	A	G	C	G	G	T	T	G	C	G	T
	MuVi/Liaoning.CHN/13.12	G	C	A	G	C	G	G	T	T	A	C	G	T
	MuVi/Beijing.CHN/21.11	G	C	A	G	C	G	G	T	T	A	C	G	C
	MuVi/Liaoning.CHN/11.12	G	C	A	G	C	G	G	T	T	A	C	G	C
	MuVi/Jiangsu.CHN/4.13/5	G	C	A	G	C	G	G	T	T	A	C	G	T
	MuVi/Heilongjiang.CHN/26.12/1	G	C	A	G	C	G	G	T	T	A	C	G	C
	MuVi/Beijing.CHN/17.07/2	G	C	A	G	C	G	G	T	T	A	C	A	T
	MuVi/Sichuan.CHN/23.12/2	G	C	A	G	C	G	G	T	T	A	C	G	T
	MuVi/Hubei.CHN/44.12/1	G	C	A	G	C	G	G	T	T	A	C	G	T
	MuVi/Jiangsu.CHN/2.13/4	G	C	A	G	C	G	G	T	T	A	C	G	T
	MuVi/Liaoning.CHN/7.12	G	C	A	G	C	G	G	T	T	A	C	G	T
	MuVi/Heilongjiang.CHN/22.12	G	C	A	G	T	G	G	T	T	A	C	G	T
	MuVi/Heilongjiang.CHN/15.13	G	C	A	G	T	G	G	T	T	A	C	G	T
	MuVi/Liaoning.CHN/5.09/1	G	C	C	G	C	G	G	T	T	A	C	G	T
	MuVi/Liaoning.CHN/1.08/2	G	C	A	G	C	G	G	T	T	A	C	G	T
	MuVi/Liaoning.CHN/10.09	G	C	A	G	C	G	G	T	T	A	C	G	T
	MuVi/Liaoning.CHN/1.08/1	G	C	A	G	C	G	G	T	T	A	C	G	C
	MuVi/Liaoning.CHN/48.10/1	G	C	A	G	C	G	G	T	T	A	C	G	C
	MuVi/Beijing.CHN/10.07	G	C	A	G	C	G	G	T	T	A	C	G	T
	MuVi/Hunan.CHN/30.11/1	G	C	A	G	C	A	G	T	T	A	G	G	T
	MuVi/Jiangsu.CHN/7.13/1	G	C	A	G	C	A	G	T	T	A	G	G	T
	MuVi/Liaoning.CHN/5.09/2	G	C	A	G	C	G	G	T	T	A	C	G	T
	MuVi/Zhejiang.CHN/11.06/1	G	C	A	G	C	G	G	T	T	A	C	G	T

1: Lineage based on Figure 1
2: SNPs are noted in green.
3: SNP position corresponding to a lineage-defining node in Figure 1 [illegible]

Supplementary Table S3: Listing of SNPs featured in the 70 F sequences of l

Lineage [1]	Strain name	679	684	688	705	711	720	723	729	732	735	738	744	748
1	MuVi/Liaoning.CHN/50.11	**C**	T	A	G	A	A	T	A	T	T	T	A	C
1	MuVi/Liaoning.CHN/48.11	**C**	T	A	G	A	A	T	A	T	T	T	A	C
1	MuVi/Heilongjiang.CHN/23.13/1	**C**	T	A	G	A	A	T	A	T	T	T	A	C
1	MuVi/Shandong.CHN/43.07	T	T	A	G	A	A	T	A	T	T	T	A	C
1	MuVi/Shandong.CHN/10.08	T	T	A	G	A	A	T	A	T	T	T	A	C
1	MuVi/Heilongjiang.CHN/26.12/2	T	T	A	G	A	A	T	A	T	T	T	A	C
1	MuVi/Heilongjiang.CHN/24.14	T	T	A	G	A	A	T	A	T	T	T	A	C
1	MuVi/Sichuan.CHN/24.15	T	T	A	G	A	A	T	A	T	T	T	A	C
1	MuVi/Shandong.CHN/11.07	T	T	A	G	A	A	T	A	T	**C**	T	A	C
1	MuVi/Beijing.CHN/15.06	T	T	A	G	A	A	T	A	T	T	T	A	C
1	MuVi/Gansu.CHN/0.02	T	T	A	G	A	A	T	A	T	T	T	A	C
1	MuVi/Jiangsu.CHN/3.13/2	T	T	A	G	A	A	T	A	T	T	T	A	C
1	MuVi/Beijing.CHN/25.06	T	T	A	G	A	A	T	A	**C**	T	T	A	**T**
1	MuVi/Shanghai.CHN/0.01	T	T	A	G	A	A	T	A	T	T	T	A	C
1	MuVi/Shandong.CHN/3.05	T	T	A	G	A	A	T	**G**	T	T	T	A	C
1	MuVi/Zhejiang.CHN/26.05	T	T	A	**A**	A	A	T	A	T	T	T	**G**	C
1	MuVi/Sichuan.CHN/23.12/1	T	T	A	G	**G**	A	T	A	T	T	T	A	C
1	MuVi/Neimeng.CHN/18.11	T	T	A	G	**G**	A	T	A	T	T	T	A	C
1	MuVi/Yunnan.CHN/47.10/2	T	T	A	G	**G**	A	T	A	T	T	T	A	C
1	MuVi/Jilin.CHN/15.08/3	T	T	A	G	**G**	A	T	A	T	T	T	A	C
1	MuVi/Jilin.CHN/15.08/1	T	T	A	G	**G**	A	T	A	T	T	T	A	C
1	MuVi/Jilin.CHN/15.08/5	T	T	A	G	**G**	A	T	A	T	T	T	A	C
4	MuVi/Shannxi.CHN/26.09/1	T	T	A	G	A	A	T	A	T	T	T	A	C
4	MuVi/Jiangsu.CHN/15.12	T	T	A	G	A	A	T	A	T	T	T	A	C
3	MuVi/Shandong.CHN/4.05	T	T	A	G	A	A	T	A	T	T	T	A	C
3	MuVi/Shannxi.CHN/9.09/2	T	T	A	G	A	A	T	A	T	T	T	A	C
3	MuVi/Shanxi.CHN/52.10/3	T	T	A	G	A	A	T	A	T	T	**C**	A	C
3	MuVi/Jiangsu.CHN/3.13/1	T	T	A	G	A	A	T	A	T	T	T	A	C
3	MuVi/Henan.CHN/48.06	T	T	A	G	A	A	T	A	T	T	T	A	C
3	MuVi/Jiangsu.CHN/9.12	T	T	A	G	A	A	T	A	T	T	T	A	C
3	MuVi/Jiangsu.CHN/4.13/3	T	T	A	G	A	A	T	A	T	T	T	A	C
2	MuVi/Shanxi.CHN/52.10/2	T	T	A	G	A	A	T	A	T	T	T	A	C
2	MuVi/Shannxi.CHN/26.09/4	T	T	A	G	A	A	T	A	T	T	T	A	C
2	MuVi/Shanxi.CHN/52.10/1	T	T	A	G	A	A	T	A	T	T	T	A	C
2	MuVi/Guangdong.CHN/21.09/1	T	T	A	G	A	A	T	A	T	T	T	A	C
2	MuVi/Heilongjiang.CHN/49.15	T	T	A	G	A	A	T	A	T	T	T	A	C
2	MuVi/Anhui.CHN/10.11/1	T	T	A	G	A	A	T	A	T	T	T	A	C
2	MuVi/Shannxi.CHN/20.12	T	T	A	G	A	A	T	A	T	T	T	A	C
2	MuVi/Liaoning.CHN/48.10/2	T	T	**G**	G	A	A	T	A	T	T	T	A	C
2	MuVi/Shandong.CHN/51.15/1	T	T	A	G	A	A	T	A	T	T	T	A	C
	MuVi/Hunan.CHN/32.11	T	T	A	G	A	A	T	A	T	T	T	A	C
	MuVi/Hunan.CHN/30.11/2	T	T	A	G	A	A	T	A	T	T	T	A	C
	MuVi/Heilongjiang.CHN/14.13/1	T	T	A	G	A	A	T	A	T	T	T	A	C
	MuVi/Jiangsu.CHN/7.12	T	T	A	G	A	**G**	T	A	**A**	T	T	A	C
	MuVi/Liaoning.CHN/9.10/1	T	T	A	G	A	A	T	A	T	T	T	A	C
	MuVi/Hunan.CHN/30.11/3	T	T	A	G	A	A	T	A	T	T	T	A	C
	MuVi/Jiangsu.CHN/12.13/2	T	T	**G**	G	A	A	T	A	T	T	T	A	C
	MuVi/Shannxi.CHN/20.15/1	T	T	A	G	A	A	T	A	T	T	T	A	C
	MuVi/Liaoning.CHN/13.12	T	T	A	G	A	A	T	A	T	T	T	A	C
	MuVi/Beijing.CHN/21.11	T	T	A	G	A	A	T	A	T	T	T	A	C
	MuVi/Liaoning.CHN/11.12	T	T	A	G	A	A	T	A	T	T	T	A	C
	MuVi/Jiangsu.CHN/4.13/5	T	T	A	G	A	A	T	A	T	T	T	A	C
	MuVi/Heilongjiang.CHN/26.12/1	T	T	A	G	A	A	T	A	T	T	T	A	C
	MuVi/Beijing.CHN/17.07/2	T	T	A	G	A	A	T	A	T	T	T	A	C
	MuVi/Sichuan.CHN/23.12/2	T	T	A	G	A	A	T	A	T	T	T	A	C
	MuVi/Hubei.CHN/44.12/1	T	T	A	G	A	A	T	A	T	T	T	A	C
	MuVi/Jiangsu.CHN/2.13/4	T	T	A	G	A	A	T	A	T	T	T	A	C
	MuVi/Liaoning.CHN/7.12	T	T	A	G	A	A	T	A	T	T	T	A	C
	MuVi/Heilongjiang.CHN/22.12	T	T	A	G	A	A	T	A	T	T	T	A	C
	MuVi/Heilongjiang.CHN/15.13	T	T	A	G	A	A	T	A	T	T	T	A	C
	MuVi/Liaoning.CHN/5.09/1	T	**A**	A	G	A	A	T	A	T	T	T	A	C
	MuVi/Liaoning.CHN/1.08/2	T	**A**	A	G	A	A	T	A	T	T	T	A	C
	MuVi/Liaoning.CHN/10.09	T	**A**	A	G	A	A	T	A	T	T	T	A	C
	MuVi/Liaoning.CHN/1.08/1	T	T	A	G	A	A	T	A	T	T	T	A	C
	MuVi/Liaoning.CHN/48.10/1	T	T	A	G	A	A	T	A	T	T	T	A	**T**
	MuVi/Beijing.CHN/10.07	T	T	A	G	A	A	T	A	T	T	T	A	C
	MuVi/Hunan.CHN/30.11/1	T	T	A	G	A	A	T	A	T	T	T	A	C
	MuVi/Jiangsu.CHN/7.13/1	T	T	A	G	A	A	T	A	T	T	T	A	C
	MuVi/Liaoning.CHN/5.09/2	T	T	A	G	A	A	**C**	A	T	T	T	A	C
	MuVi/Zhejiang.CHN/11.06/1	T	T	A	G	A	A	T	A	T	T	T	A	C

1: Lineage based on Figure 1
2: SNPs are noted in green.
3: SNP position corresponding to a lineage-defining node in Figure 1

Supplementary Table S3: Listing of SNPs featured in the 70 F sequences of I

Lineage [1]	Strain name	756	759	773	789	791	793	795	796	806	807	811	813	816
1	MuVi/Liaoning.CHN/50.11	C	T	A	G	T	G	**C**	G	T	G	G	T	G
1	MuVi/Liaoning.CHN/48.11	C	T	A	G	T	G	T	G	T	G	G	T	G
1	MuVi/Heilongjiang.CHN/23.13/1	C	T	A	G	T	G	T	G	T	G	G	T	G
1	MuVi/Shandong.CHN/43.07	C	T	A	G	T	G	T	G	T	G	G	T	G
1	MuVi/Shandong.CHN/10.08	C	T	A	G	T	G	T	G	T	G	G	T	G
1	MuVi/Heilongjiang.CHN/26.12/2	C	T	A	G	T	G	T	G	T	G	G	T	G
1	MuVi/Heilongjiang.CHN/24.14	C	T	A	G	T	G	T	G	T	G	G	T	G
1	MuVi/Sichuan.CHN/24.15	C	T	A	G	T	G	T	G	T	G	G	T	G
1	MuVi/Shandong.CHN/11.07	C	T	A	G	T	G	T	G	T	G	G	T	G
1	MuVi/Beijing.CHN/15.06	C	T	A	G	**A**	G	T	G	T	G	**A**	T	G
1	MuVi/Gansu.CHN/0.02	C	T	A	G	T	G	T	G	T	G	G	T	G
1	MuVi/Jiangsu.CHN/3.13/2	C	T	A	**T**	T	G	T	G	T	G	G	T	G
1	MuVi/Beijing.CHN/25.06	C	T	A	G	T	G	T	G	T	G	G	T	G
1	MuVi/Shanghai.CHN/0.01	**A**	T	A	G	T	G	T	G	T	G	G	T	G
1	MuVi/Shandong.CHN/3.05	**A**	T	A	G	T	G	T	G	T	G	G	T	G
1	MuVi/Zhejiang.CHN/26.05	C	T	A	G	T	G	T	G	T	G	G	T	G
1	MuVi/Sichuan.CHN/23.12/1	C	T	A	G	T	G	T	G	T	G	G	T	G
1	MuVi/Neimeng.CHN/18.11	C	T	A	**T**	T	G	T	G	T	G	G	T	G
1	MuVi/Yunnan.CHN/47.10/2	C	T	A	**T**	T	G	T	G	T	G	G	T	G
1	MuVi/Jilin.CHN/15.08/3	C	T	A	G	T	G	T	G	T	G	G	T	G
1	MuVi/Jilin.CHN/15.08/1	C	T	A	G	T	G	T	G	T	G	G	T	G
1	MuVi/Jilin.CHN/15.08/5	C	T	A	G	T	G	T	G	T	G	G	T	G
4	MuVi/Shannxi.CHN/26.09/1	**T**	T	A	G	T	G	T	G	T	G	G	T	G
4	MuVi/Jiangsu.CHN/15.12	**T**	T	A	G	T	G	T	G	T	G	G	T	G
3	MuVi/Shandong.CHN/4.05	C	T	A	G	T	G	T	G	T	G	G	T	G
3	MuVi/Shannxi.CHN/9.09/2	C	T	A	G	T	G	T	G	T	G	G	T	G
3	MuVi/Shanxi.CHN/52.10/3	C	T	A	G	T	G	T	G	T	G	G	T	G
3	MuVi/Jiangsu.CHN/3.13/1	C	T	A	G	T	G	T	G	T	G	G	T	G
3	MuVi/Henan.CHN/48.06	C	T	A	G	T	G	T	G	**C**	G	G	T	G
3	MuVi/Jiangsu.CHN/9.12	C	T	A	G	T	G	T	G	T	G	G	T	G
3	MuVi/Jiangsu.CHN/4.13/3	C	T	A	G	T	G	T	G	T	G	G	T	G
2	MuVi/Shanxi.CHN/52.10/2	C	T	A	G	T	G	T	G	T	G	G	T	**A**
2	MuVi/Shannxi.CHN/26.09/4	C	T	A	G	T	G	T	G	T	G	G	T	G
2	MuVi/Shanxi.CHN/52.10/1	C	T	A	G	T	G	T	G	T	G	G	T	G
2	MuVi/Guangdong.CHN/21.09/1	C	T	A	G	T	G	T	G	T	G	G	T	G
2	MuVi/Heilongjiang.CHN/49.15	C	T	A	**A**	T	G	T	G	T	G	G	T	G
2	MuVi/Anhui.CHN/10.11/1	C	T	A	**A**	T	G	T	G	T	G	G	T	G
2	MuVi/Shannxi.CHN/20.12	C	T	A	**A**	T	G	T	G	T	G	G	T	G
2	MuVi/Liaoning.CHN/48.10/2	C	T	A	**A**	T	G	T	G	T	G	G	T	G
2	MuVi/Shandong.CHN/51.15/1	C	T	A	**A**	T	G	T	G	T	G	G	T	G
	MuVi/Hunan.CHN/32.11	C	T	A	G	T	G	T	G	T	G	G	T	G
	MuVi/Hunan.CHN/30.11/2	C	T	A	G	T	G	T	G	T	G	G	T	G
	MuVi/Heilongjiang.CHN/14.13/1	C	T	A	G	T	G	T	G	T	G	G	T	G
	MuVi/Jiangsu.CHN/7.12	C	T	A	G	T	G	T	G	T	G	G	T	G
	MuVi/Liaoning.CHN/9.10/1	C	T	A	G	T	G	T	G	T	G	G	T	G
	MuVi/Hunan.CHN/30.11/3	C	T	A	G	T	G	T	G	T	G	G	T	G
	MuVi/Jiangsu.CHN/12.13/2	C	T	A	G	T	G	T	G	T	G	G	T	G
	MuVi/Shannxi.CHN/20.15/1	C	T	A	G	T	G	T	G	T	G	G	T	G
	MuVi/Liaoning.CHN/13.12	C	T	A	**A**	T	G	T	**A**	T	G	G	T	G
	MuVi/Beijing.CHN/21.11	**T**	T	A	G	T	G	T	G	T	G	G	T	G
	MuVi/Liaoning.CHN/11.12	**T**	T	A	G	T	G	T	G	T	G	G	T	G
	MuVi/Jiangsu.CHN/4.13/5	C	T	A	G	T	G	T	G	T	G	G	T	G
	MuVi/Heilongjiang.CHN/26.12/1	C	T	A	G	T	G	T	G	T	G	G	**C**	G
	MuVi/Beijing.CHN/17.07/2	C	T	A	G	T	G	T	G	T	G	G	T	G
	MuVi/Sichuan.CHN/23.12/2	C	T	A	G	T	G	T	G	T	G	G	T	G
	MuVi/Hubei.CHN/44.12/1	C	T	A	G	T	G	T	G	T	G	G	T	G
	MuVi/Jiangsu.CHN/2.13/4	C	T	A	G	T	G	T	G	T	G	G	T	G
	MuVi/Liaoning.CHN/7.12	C	T	A	G	T	G	T	G	T	G	G	T	G
	MuVi/Heilongjiang.CHN/22.12	C	T	A	G	T	G	T	G	T	G	G	T	G
	MuVi/Heilongjiang.CHN/15.13	C	T	A	G	T	G	T	G	T	G	G	T	G
	MuVi/Liaoning.CHN/5.09/1	C	T	A	G	T	G	T	G	T	G	G	T	G
	MuVi/Liaoning.CHN/1.08/2	C	T	A	G	T	G	T	G	T	G	G	T	G
	MuVi/Liaoning.CHN/10.09	C	T	A	G	T	G	T	G	T	G	G	T	G
	MuVi/Liaoning.CHN/1.08/1	C	T	A	G	T	G	T	G	T	G	G	T	G
	MuVi/Liaoning.CHN/48.10/1	C	T	A	G	T	G	T	G	T	G	G	T	G
	MuVi/Beijing.CHN/10.07	C	T	A	G	T	G	T	G	T	G	G	T	G
	MuVi/Hunan.CHN/30.11/1	C	T	A	G	T	G	T	G	T	G	G	**C**	G
	MuVi/Jiangsu.CHN/7.13/1	C	**C**	**C**	G	T	**T**	T	G	T	G	G	**C**	G
	MuVi/Liaoning.CHN/5.09/2	C	T	A	G	T	G	T	G	T	G	G	T	G
	MuVi/Zhejiang.CHN/11.06/1	C	T	A	G	T	G	T	G	T	**A**	G	T	G

1: Lineage based on Figure 1
2: SNPs are noted in green.
3: SNP position corresponding to a lineage-defining node in Figure 1 [illegible]

Supplementary Table S3: Listing of SNPs featured in the 70 F sequences of l

Lineage [1]	Strain name	817	821	828	831	832	838	846	849	852	855	868	870	879
1	MuVi/Liaoning.CHN/50.11	A	A	A	C	A	A	A	T	A	G	C	C	T
1	MuVi/Liaoning.CHN/48.11	A	A	A	C	A	A	A	T	A	G	T	C	T
1	MuVi/Heilongjiang.CHN/23.13/1	A	A	A	C	A	A	A	T	A	G	T	C	T
1	MuVi/Shandong.CHN/43.07	A	A	A	C	A	A	A	T	A	G	T	C	T
1	MuVi/Shandong.CHN/10.08	A	A	A	C	A	A	A	T	A	G	T	C	T
1	MuVi/Heilongjiang.CHN/26.12/2	A	A	A	C	A	A	A	T	A	G	T	C	T
1	MuVi/Heilongjiang.CHN/24.14	A	A	A	C	A	A	A	T	A	A	T	C	T
1	MuVi/Sichuan.CHN/24.15	A	A	A	C	A	A	A	T	A	G	T	C	T
1	MuVi/Shandong.CHN/11.07	A	A	A	C	A	A	A	T	A	G	T	C	T
1	MuVi/Beijing.CHN/15.06	G	A	A	C	A	A	A	T	A	G	T	C	T
1	MuVi/Gansu.CHN/0.02	A	A	A	C	A	A	A	T	A	G	C	C	T
1	MuVi/Jiangsu.CHN/3.13/2	A	A	A	C	A	A	A	T	A	G	T	C	T
1	MuVi/Beijing.CHN/25.06	A	A	A	C	A	A	A	T	A	G	T	C	T
1	MuVi/Shanghai.CHN/0.01	A	A	A	C	A	A	A	T	A	G	T	C	T
1	MuVi/Shandong.CHN/3.05	A	A	A	C	A	A	A	T	A	G	T	C	T
1	MuVi/Zhejiang.CHN/26.05	A	A	A	C	A	A	A	T	A	G	T	C	T
1	MuVi/Sichuan.CHN/23.12/1	A	A	A	C	A	A	A	T	A	G	T	C	A
1	MuVi/Neimeng.CHN/18.11	A	A	A	C	A	A	A	T	A	G	T	C	A
1	MuVi/Yunnan.CHN/47.10/2	A	A	A	C	A	A	A	T	A	G	T	C	A
1	MuVi/Jilin.CHN/15.08/3	A	A	A	C	A	A	A	T	A	G	T	C	A
1	MuVi/Jilin.CHN/15.08/1	A	A	A	C	A	A	A	T	A	G	T	C	A
1	MuVi/Jilin.CHN/15.08/5	A	A	A	C	A	A	A	T	A	G	T	C	A
4	MuVi/Shannxi.CHN/26.09/1	A	A	A	C	A	A	A	T	A	A	T	C	G
4	MuVi/Jiangsu.CHN/15.12	A	A	A	C	A	A	A	T	A	A	T	C	G
3	MuVi/Shandong.CHN/4.05	A	A	A	T	A	A	A	T	A	G	T	C	G
3	MuVi/Shannxi.CHN/9.09/2	A	A	A	C	A	A	A	T	A	G	T	C	G
3	MuVi/Shanxi.CHN/52.10/3	A	A	A	C	A	G	A	T	A	G	T	C	G
3	MuVi/Jiangsu.CHN/3.13/1	A	A	A	C	A	A	A	T	A	G	T	C	G
3	MuVi/Henan.CHN/48.06	A	A	A	C	A	A	A	T	A	G	T	C	G
3	MuVi/Jiangsu.CHN/9.12	A	A	A	C	A	A	A	T	A	G	T	C	G
3	MuVi/Jiangsu.CHN/4.13/3	A	A	A	C	A	A	A	T	A	G	T	C	G
2	MuVi/Shanxi.CHN/52.10/2	A	A	G	C	A	A	A	T	G	G	T	C	G
2	MuVi/Shannxi.CHN/26.09/4	A	A	G	C	A	A	A	T	G	G	T	C	G
2	MuVi/Shanxi.CHN/52.10/1	A	A	G	C	A	A	A	T	G	G	T	C	G
2	MuVi/Guangdong.CHN/21.09/1	A	A	G	C	A	A	A	T	G	G	T	C	G
2	MuVi/Heilongjiang.CHN/49.15	A	A	G	C	A	A	A	T	G	G	T	C	G
2	MuVi/Anhui.CHN/10.11/1	A	A	G	C	A	A	A	T	G	G	T	C	G
2	MuVi/Shannxi.CHN/20.12	A	A	G	C	A	A	A	T	G	G	T	C	G
2	MuVi/Liaoning.CHN/48.10/2	A	A	G	C	A	A	A	T	G	G	T	C	G
2	MuVi/Shandong.CHN/51.15/1	A	G	G	C	A	A	A	T	G	G	T	C	G
	MuVi/Hunan.CHN/32.11	A	A	A	C	A	A	A	T	A	G	T	C	G
	MuVi/Hunan.CHN/30.11/2	A	A	A	C	A	A	A	T	A	G	T	C	G
	MuVi/Heilongjiang.CHN/14.13/1	A	A	A	C	A	A	A	T	A	G	T	C	G
	MuVi/Jiangsu.CHN/7.12	A	A	A	C	A	A	A	T	A	G	T	C	G
	MuVi/Liaoning.CHN/9.10/1	A	A	A	C	A	A	A	T	A	G	T	C	G
	MuVi/Hunan.CHN/30.11/3	A	A	A	C	A	A	A	T	A	G	T	C	G
	MuVi/Jiangsu.CHN/12.13/2	A	A	A	C	A	A	A	T	A	G	T	C	G
	MuVi/Shannxi.CHN/20.15/1	A	A	A	C	A	A	A	T	A	G	T	C	G
	MuVi/Liaoning.CHN/13.12	A	A	A	C	A	A	A	T	A	G	T	C	G
	MuVi/Beijing.CHN/21.11	A	A	A	C	A	A	A	T	A	G	T	C	G
	MuVi/Liaoning.CHN/11.12	A	A	A	C	A	A	A	T	A	G	T	C	G
	MuVi/Jiangsu.CHN/4.13/5	A	A	A	C	A	A	A	T	A	G	T	C	G
	MuVi/Heilongjiang.CHN/26.12/1	A	A	A	C	A	A	A	T	A	G	T	C	G
	MuVi/Beijing.CHN/17.07/2	A	A	A	C	A	A	A	T	A	G	T	C	G
	MuVi/Sichuan.CHN/23.12/2	A	A	A	C	A	A	A	T	A	G	T	C	G
	MuVi/Hubei.CHN/44.12/1	A	A	A	C	A	A	A	T	A	G	T	T	G
	MuVi/Jiangsu.CHN/2.13/4	A	A	A	C	A	A	A	T	A	G	T	C	G
	MuVi/Liaoning.CHN/7.12	A	A	A	C	A	A	A	T	A	G	T	C	G
	MuVi/Heilongjiang.CHN/22.12	A	A	A	C	A	A	A	T	A	G	T	C	G
	MuVi/Heilongjiang.CHN/15.13	A	A	A	C	A	A	A	T	A	G	T	C	G
	MuVi/Liaoning.CHN/5.09/1	A	A	A	C	A	A	A	T	A	G	T	C	G
	MuVi/Liaoning.CHN/1.08/2	A	A	A	C	A	A	A	T	A	G	T	C	G
	MuVi/Liaoning.CHN/10.09	A	A	A	C	A	A	A	T	A	G	T	C	G
	MuVi/Liaoning.CHN/1.08/1	A	A	A	C	A	A	A	T	A	G	T	C	G
	MuVi/Liaoning.CHN/48.10/1	A	A	A	C	A	A	A	C	A	G	T	C	G
	MuVi/Beijing.CHN/10.07	A	A	A	C	A	A	A	T	A	G	T	C	G
	MuVi/Hunan.CHN/30.11/1	A	A	A	C	A	A	A	T	A	G	T	C	A
	MuVi/Jiangsu.CHN/7.13/1	A	A	A	C	T	A	T	T	A	G	T	C	G
	MuVi/Liaoning.CHN/5.09/2	A	A	A	C	A	A	A	T	A	G	T	C	G
	MuVi/Zhejiang.CHN/11.06/1	A	A	A	C	A	A	A	T	A	G	T	C	G

1: Lineage based on Figure 1
2: SNPs are noted in green.
3: SNP position corresponding to a lineage-defining node in Figure 1 a

Supplementary Table S3: Listing of SNPs featured in the 70 F sequences of

Lineage [1]	Strain name	891	897	903	906	915	918	919	925	928	936	939	941	947
1	MuVi/Liaoning.CHN/50.11	T	G	C	A	A	A	G	A	T	C	G	A	A
1	MuVi/Liaoning.CHN/48.11	T	G	C	A	A	A	G	A	T	C	G	A	A
1	MuVi/Heilongjiang.CHN/23.13/1	T	G	C	A	A	A	G	A	T	C	G	A	A
1	MuVi/Shandong.CHN/43.07	T	G	C	A	A	A	G	A	T	C	G	A	A
1	MuVi/Shandong.CHN/10.08	T	G	C	A	A	A	G	A	T	C	G	A	A
1	MuVi/Heilongjiang.CHN/26.12/2	T	G	C	A	A	A	G	A	T	C	A	A	A
1	MuVi/Heilongjiang.CHN/24.14	T	G	C	A	A	A	G	A	T	C	G	A	A
1	MuVi/Sichuan.CHN/24.15	T	G	C	A	A	A	G	A	T	C	G	A	A
1	MuVi/Shandong.CHN/11.07	T	G	C	A	A	A	G	A	T	C	G	A	A
1	MuVi/Beijing.CHN/15.06	T	G	C	A	A	A	G	A	T	C	G	A	A
1	MuVi/Gansu.CHN/0.02	T	G	C	A	A	A	G	A	T	C	G	A	A
1	MuVi/Jiangsu.CHN/3.13/2	T	G	C	A	A	A	G	A	T	C	G	A	A
1	MuVi/Beijing.CHN/25.06	T	G	C	G	A	A	G	A	T	C	G	A	A
1	MuVi/Shanghai.CHN/0.01	T	G	C	A	A	A	G	A	T	C	G	A	T
1	MuVi/Shandong.CHN/3.05	T	G	C	A	A	A	G	A	T	C	G	A	A
1	MuVi/Zhejiang.CHN/26.05	T	G	C	A	A	A	G	A	T	C	G	A	A
1	MuVi/Sichuan.CHN/23.12/1	T	G	C	A	A	A	G	A	T	C	G	A	A
1	MuVi/Neimeng.CHN/18.11	C	G	C	A	A	A	G	A	T	C	G	A	A
1	MuVi/Yunnan.CHN/47.10/2	T	G	C	A	A	A	G	A	T	C	G	A	A
1	MuVi/Jilin.CHN/15.08/3	T	G	C	A	A	A	G	A	T	C	G	A	A
1	MuVi/Jilin.CHN/15.08/1	T	G	C	A	A	A	G	A	T	C	G	A	A
1	MuVi/Jilin.CHN/15.08/5	T	G	C	A	A	A	G	A	T	C	G	A	A
4	MuVi/Shannxi.CHN/26.09/1	T	G	C	A	A	G	G	A	T	C	G	A	A
4	MuVi/Jiangsu.CHN/15.12	T	G	C	A	A	G	G	A	T	C	G	G	A
3	MuVi/Shandong.CHN/4.05	T	G	C	A	A	A	G	A	T	C	G	A	A
3	MuVi/Shannxi.CHN/9.09/2	T	G	C	A	A	A	G	A	T	C	G	A	A
3	MuVi/Shanxi.CHN/52.10/3	T	G	C	A	A	A	G	A	T	A	G	A	A
3	MuVi/Jiangsu.CHN/3.13/1	T	G	C	A	A	A	G	A	T	C	G	A	A
3	MuVi/Henan.CHN/48.06	C	G	C	A	A	A	G	A	T	C	G	A	A
3	MuVi/Jiangsu.CHN/9.12	C	G	C	A	A	A	G	A	T	C	G	A	A
3	MuVi/Jiangsu.CHN/4.13/3	C	G	C	A	A	A	G	A	T	C	G	A	A
2	MuVi/Shanxi.CHN/52.10/2	T	G	C	A	A	A	G	A	T	C	G	A	A
2	MuVi/Shannxi.CHN/26.09/4	T	G	C	A	A	A	G	A	T	C	G	A	A
2	MuVi/Shanxi.CHN/52.10/1	T	G	C	A	A	A	G	A	T	C	G	A	A
2	MuVi/Guangdong.CHN/21.09/1	T	G	C	A	A	A	G	A	T	C	G	A	A
2	MuVi/Heilongjiang.CHN/49.15	T	G	C	A	A	A	G	A	T	C	G	A	A
2	MuVi/Anhui.CHN/10.11/1	T	G	C	A	A	A	G	A	T	C	G	A	A
2	MuVi/Shannxi.CHN/20.12	T	G	C	A	A	A	G	A	T	C	G	A	A
2	MuVi/Liaoning.CHN/48.10/2	T	G	C	A	A	A	G	A	T	C	G	A	A
2	MuVi/Shandong.CHN/51.15/1	T	G	C	A	A	A	G	A	T	C	G	A	A
	MuVi/Hunan.CHN/32.11	T	G	C	A	A	A	G	G	T	C	A	A	A
	MuVi/Hunan.CHN/30.11/2	T	G	C	A	A	A	G	G	T	C	G	A	A
	MuVi/Heilongjiang.CHN/14.13/1	T	G	C	A	A	A	G	G	T	C	G	A	A
	MuVi/Jiangsu.CHN/7.12	T	G	C	A	A	A	G	G	T	C	G	A	A
	MuVi/Liaoning.CHN/9.10/1	T	G	C	A	A	A	G	G	T	C	G	A	A
	MuVi/Hunan.CHN/30.11/3	T	G	C	A	A	A	G	G	T	C	G	A	A
	MuVi/Jiangsu.CHN/12.13/2	T	G	C	A	A	A	G	G	T	C	G	A	A
	MuVi/Shannxi.CHN/20.15/1	T	G	C	A	A	A	G	G	T	C	G	A	A
	MuVi/Liaoning.CHN/13.12	T	G	C	A	A	A	G	G	T	C	G	A	A
	MuVi/Beijing.CHN/21.11	T	G	C	A	A	A	G	G	T	C	G	A	A
	MuVi/Liaoning.CHN/11.12	T	G	C	A	A	A	G	G	T	C	G	A	A
	MuVi/Jiangsu.CHN/4.13/5	T	G	C	A	A	A	G	G	T	C	G	A	A
	MuVi/Heilongjiang.CHN/26.12/1	T	G	C	A	A	A	G	G	T	C	G	A	A
	MuVi/Beijing.CHN/17.07/2	T	G	C	A	A	A	G	G	C	C	G	A	A
	MuVi/Sichuan.CHN/23.12/2	T	G	C	A	A	A	G	G	T	C	G	A	A
	MuVi/Hubei.CHN/44.12/1	T	G	C	A	G	A	G	G	T	C	G	A	A
	MuVi/Jiangsu.CHN/2.13/4	T	G	C	A	A	A	G	G	T	C	G	A	A
	MuVi/Liaoning.CHN/7.12	T	G	C	A	A	A	G	G	T	C	G	A	A
	MuVi/Heilongjiang.CHN/22.12	T	G	C	A	A	A	G	G	T	C	G	A	A
	MuVi/Heilongjiang.CHN/15.13	T	G	C	A	A	A	G	G	T	C	G	A	A
	MuVi/Liaoning.CHN/5.09/1	T	G	C	A	A	A	G	G	T	C	G	A	A
	MuVi/Liaoning.CHN/1.08/2	T	G	C	A	A	A	G	G	T	C	A	A	A
	MuVi/Liaoning.CHN/10.09	T	G	C	A	A	A	G	G	T	C	A	A	A
	MuVi/Liaoning.CHN/1.08/1	T	G	C	A	A	A	G	G	T	C	G	A	A
	MuVi/Liaoning.CHN/48.10/1	T	A	C	A	A	A	G	G	T	C	G	A	A
	MuVi/Beijing.CHN/10.07	T	G	C	A	A	A	T	A	T	C	G	A	A
	MuVi/Hunan.CHN/30.11/1	T	G	T	A	A	A	G	A	T	C	G	A	A
	MuVi/Jiangsu.CHN/7.13/1	T	G	T	A	A	A	G	A	T	C	G	A	A
	MuVi/Liaoning.CHN/5.09/2	T	G	C	A	A	A	G	A	T	C	G	A	A
	MuVi/Zhejiang.CHN/11.06/1	T	G	C	A	A	A	G	A	T	C	G	A	A

1: Lineage based on Figure 1
2: SNPs are noted in green.
3: SNP position corresponding to a lineage-defining node in Figure 1

Supplementary Table S3: Listing of SNPs featured in the 70 F sequences of I

Lineage [1]	Strain name	948	954	960	963	969	974	975	976	978	985	986	987	988
						SNP position in F								
1	MuVi/Liaoning.CHN/50.11	G	C	A	T	T	A	G	T	G	C	A	C	A
1	MuVi/Liaoning.CHN/48.11	G	C	A	T	T	A	G	T	G	C	A	C	A
1	MuVi/Heilongjiang.CHN/23.13/1	G	C	A	T	T	A	G	T	G	C	A	C	A
1	MuVi/Shandong.CHN/43.07	G	C	A	T	T	A	G	T	G	C	A	C	A
1	MuVi/Shandong.CHN/10.08	G	C	A	T	T	A	G	T	G	C	A	C	A
1	MuVi/Heilongjiang.CHN/26.12/2	G	C	A	T	T	A	G	T	G	C	A	C	A
1	MuVi/Heilongjiang.CHN/24.14	G	C	A	T	T	A	A	T	G	C	A	C	A
1	MuVi/Sichuan.CHN/24.15	G	C	A	T	T	A	A	T	G	C	A	C	A
1	MuVi/Shandong.CHN/11.07	G	T	T	T	T	A	G	T	G	C	A	C	A
1	MuVi/Beijing.CHN/15.06	G	C	A	T	T	A	G	T	G	C	A	C	A
1	MuVi/Gansu.CHN/0.02	G	C	A	T	T	A	G	T	G	C	A	C	A
1	MuVi/Jiangsu.CHN/3.13/2	G	C	A	T	T	A	G	T	G	C	A	A	A
1	MuVi/Beijing.CHN/25.06	G	C	A	T	T	A	G	T	G	C	A	A	A
1	MuVi/Shanghai.CHN/0.01	G	C	A	T	T	A	G	T	G	C	A	A	A
1	MuVi/Shandong.CHN/3.05	G	C	A	T	T	A	G	T	G	C	A	A	A
1	MuVi/Zhejiang.CHN/26.05	G	C	A	T	T	A	G	T	G	C	A	A	A
1	MuVi/Sichuan.CHN/23.12/1	G	C	A	T	T	A	G	T	G	C	A	A	A
1	MuVi/Neimeng.CHN/18.11	G	C	A	T	T	A	G	T	G	C	A	A	A
1	MuVi/Yunnan.CHN/47.10/2	G	C	A	T	T	A	G	T	G	C	A	A	A
1	MuVi/Jilin.CHN/15.08/3	G	C	A	T	T	A	G	T	G	C	A	A	A
1	MuVi/Jilin.CHN/15.08/1	G	C	A	T	T	A	G	T	G	C	A	A	A
1	MuVi/Jilin.CHN/15.08/5	G	C	A	T	T	A	G	T	G	C	A	A	A
4	MuVi/Shannxi.CHN/26.09/1	G	C	A	T	T	A	G	T	G	C	A	C	A
4	MuVi/Jiangsu.CHN/15.12	G	C	A	T	T	A	G	T	G	C	A	C	A
3	MuVi/Shandong.CHN/4.05	G	C	A	T	T	A	G	T	G	C	A	C	A
3	MuVi/Shannxi.CHN/9.09/2	G	C	G	A	T	A	G	T	G	C	A	C	A
3	MuVi/Shanxi.CHN/52.10/3	G	C	A	C	T	A	G	T	G	C	A	C	A
3	MuVi/Jiangsu.CHN/3.13/1	G	C	A	C	T	A	G	T	G	C	A	C	A
3	MuVi/Henan.CHN/48.06	G	C	A	T	T	A	G	C	A	C	A	C	A
3	MuVi/Jiangsu.CHN/9.12	G	C	A	T	T	A	G	C	G	C	A	C	A
3	MuVi/Jiangsu.CHN/4.13/3	G	C	A	T	T	A	G	C	G	C	A	C	A
2	MuVi/Shanxi.CHN/52.10/2	G	C	A	T	T	A	G	T	G	T	A	C	A
2	MuVi/Shannxi.CHN/26.09/4	G	C	A	T	T	A	G	T	G	T	A	C	A
2	MuVi/Shanxi.CHN/52.10/1	G	C	A	T	T	A	G	T	G	T	A	C	A
2	MuVi/Guangdong.CHN/21.09/1	G	C	A	T	T	A	G	T	G	T	A	C	A
2	MuVi/Heilongjiang.CHN/49.15	G	C	A	T	T	A	G	T	G	T	A	C	A
2	MuVi/Anhui.CHN/10.11/1	G	C	A	T	T	A	G	T	G	T	A	C	A
2	MuVi/Shannxi.CHN/20.12	G	C	A	T	T	A	G	T	G	T	A	C	A
2	MuVi/Liaoning.CHN/48.10/2	G	C	A	T	T	A	G	T	G	T	A	C	A
2	MuVi/Shandong.CHN/51.15/1	G	C	A	T	T	A	G	T	G	T	C	C	A
	MuVi/Hunan.CHN/32.11	G	C	A	T	T	A	G	T	G	C	A	C	A
	MuVi/Hunan.CHN/30.11/2	A	C	A	T	T	A	G	T	G	C	A	C	A
	MuVi/Heilongjiang.CHN/14.13/1	G	C	T	T	T	A	G	T	G	C	A	C	A
	MuVi/Jiangsu.CHN/7.12	G	C	T	T	T	A	G	T	G	C	A	C	A
	MuVi/Liaoning.CHN/9.10/1	G	C	T	T	T	A	G	T	G	C	A	C	A
	MuVi/Hunan.CHN/30.11/3	G	C	T	T	T	A	G	T	G	C	A	C	A
	MuVi/Jiangsu.CHN/12.13/2	G	C	A	T	T	A	G	T	G	C	A	C	A
	MuVi/Shannxi.CHN/20.15/1	G	C	A	T	T	A	G	T	G	C	A	C	A
	MuVi/Liaoning.CHN/13.12	G	C	A	T	T	A	G	T	G	C	A	C	A
	MuVi/Beijing.CHN/21.11	G	C	A	T	T	A	G	T	G	C	A	C	A
	MuVi/Liaoning.CHN/11.12	G	C	A	T	T	A	G	T	G	C	A	C	A
	MuVi/Jiangsu.CHN/4.13/5	G	C	A	T	T	A	G	T	G	C	A	C	A
	MuVi/Heilongjiang.CHN/26.12/1	G	C	A	C	C	A	G	C	G	C	A	C	A
	MuVi/Beijing.CHN/17.07/2	G	C	A	T	T	A	G	T	G	C	A	C	A
	MuVi/Sichuan.CHN/23.12/2	G	C	A	T	T	A	G	T	G	C	A	C	A
	MuVi/Hubei.CHN/44.12/1	G	C	A	T	T	A	G	T	G	C	A	C	A
	MuVi/Jiangsu.CHN/2.13/4	G	C	A	T	T	A	G	T	G	C	A	C	A
	MuVi/Liaoning.CHN/7.12	G	C	A	T	T	G	G	T	G	C	A	C	A
	MuVi/Heilongjiang.CHN/22.12	G	C	A	T	T	A	G	T	G	C	A	C	A
	MuVi/Heilongjiang.CHN/15.13	G	C	A	T	T	A	G	T	G	C	A	C	A
	MuVi/Liaoning.CHN/5.09/1	G	C	A	T	T	A	G	T	G	C	A	C	A
	MuVi/Liaoning.CHN/1.08/2	G	C	A	T	T	A	G	T	G	C	A	C	A
	MuVi/Liaoning.CHN/10.09	G	C	A	T	T	A	G	T	G	C	A	C	A
	MuVi/Liaoning.CHN/1.08/1	G	C	A	T	T	A	G	T	G	C	A	C	A
	MuVi/Liaoning.CHN/48.10/1	G	C	A	T	T	A	G	T	G	C	A	C	A
	MuVi/Beijing.CHN/10.07	G	C	A	T	T	A	G	T	G	C	A	C	A
	MuVi/Hunan.CHN/30.11/1	G	C	A	T	T	A	G	T	G	C	A	C	A
	MuVi/Jiangsu.CHN/7.13/1	G	C	A	T	T	A	G	T	G	C	A	C	C
	MuVi/Liaoning.CHN/5.09/2	G	C	A	T	T	A	G	T	G	C	A	C	A
	MuVi/Zhejiang.CHN/11.06/1	G	C	A	T	T	A	G	T	G	C	A	C	A

1: Lineage based on Figure 1
2: SNPs are noted in green.
3: SNP position corresponding to a lineage-defining node in Figure 1 a

Supplementary Table S3: Listing of SNPs featured in the 70 F sequences of [illegible]

Lineage [1]	Strain name	990	996	999	1002	1011	1014	1023	1027	1032	1033	1034
1	MuVi/Liaoning.CHN/50.11	C	C	C	A	G	G	G	C	A	T	C
1	MuVi/Liaoning.CHN/48.11	C	C	C	A	G	G	G	C	A	T	C
1	MuVi/Heilongjiang.CHN/23.13/1	C	C	C	A	G	G	G	C	A	T	C
1	MuVi/Shandong.CHN/43.07	C	C	C	A	G	G	G	C	A	T	C
1	MuVi/Shandong.CHN/10.08	C	C	C	A	G	G	G	C	A	T	C
1	MuVi/Heilongjiang.CHN/26.12/2	C	C	C	A	G	G	G	C	A	T	C
1	MuVi/Heilongjiang.CHN/24.14	C	C	C	A	G	G	G	C	A	T	C
1	MuVi/Sichuan.CHN/24.15	C	C	C	A	G	A	G	C	A	T	C
1	MuVi/Shandong.CHN/11.07	C	C	C	A	G	A	G	C	A	T	C
1	MuVi/Beijing.CHN/15.06	C	C	C	A	G	A	G	C	A	T	C
1	MuVi/Gansu.CHN/0.02	C	C	C	A	G	A	G	C	A	T	C
1	MuVi/Jiangsu.CHN/3.13/2	C	C	C	A	G	A	G	C	A	T	C
1	MuVi/Beijing.CHN/25.06	T	C	C	A	G	A	G	C	A	T	C
1	MuVi/Shanghai.CHN/0.01	C	C	C	A	G	A	G	C	A	T	C
1	MuVi/Shandong.CHN/3.05	C	C	C	A	G	A	G	C	A	T	C
1	MuVi/Zhejiang.CHN/26.05	C	C	C	A	G	A	G	C	A	T	C
1	MuVi/Sichuan.CHN/23.12/1	C	C	C	A	G	A	G	C	A	T	C
1	MuVi/Neimeng.CHN/18.11	C	C	C	A	G	A	G	C	A	T	C
1	MuVi/Yunnan.CHN/47.10/2	C	C	C	A	G	A	G	C	A	T	C
1	MuVi/Jilin.CHN/15.08/3	C	C	C	A	G	A	G	C	A	T	C
1	MuVi/Jilin.CHN/15.08/1	C	C	C	A	G	A	G	C	A	T	C
1	MuVi/Jilin.CHN/15.08/5	C	C	C	A	G	A	G	C	A	T	C
4	MuVi/Shannxi.CHN/26.09/1	C	C	C	A	G	A	G	C	A	T	C
4	MuVi/Jiangsu.CHN/15.12	C	C	C	A	G	A	G	C	A	T	C
3	MuVi/Shandong.CHN/4.05	C	T	C	A	G	A	G	C	A	T	C
3	MuVi/Shannxi.CHN/9.09/2	C	T	C	A	G	A	G	C	A	T	C
3	MuVi/Shanxi.CHN/52.10/3	C	T	C	A	G	A	G	C	A	A	C
3	MuVi/Jiangsu.CHN/3.13/1	C	T	C	A	G	A	G	C	A	T	C
3	MuVi/Henan.CHN/48.06	C	T	C	A	G	A	G	C	A	T	C
3	MuVi/Jiangsu.CHN/9.12	C	T	C	A	G	A	G	C	A	T	C
3	MuVi/Jiangsu.CHN/4.13/3	C	T	C	A	G	A	G	C	A	T	C
2	MuVi/Shanxi.CHN/52.10/2	C	C	C	A	G	A	G	T	A	T	C
2	MuVi/Shannxi.CHN/26.09/4	C	C	C	A	G	A	G	T	A	T	C
2	MuVi/Shanxi.CHN/52.10/1	C	C	T	A	G	A	G	T	A	T	C
2	MuVi/Guangdong.CHN/21.09/1	C	C	C	A	G	A	G	T	A	T	C
2	MuVi/Heilongjiang.CHN/49.15	C	C	C	A	G	A	G	T	A	T	C
2	MuVi/Anhui.CHN/10.11/1	C	C	C	A	G	A	G	T	A	T	C
2	MuVi/Shannxi.CHN/20.12	C	C	C	A	G	A	G	T	A	T	C
2	MuVi/Liaoning.CHN/48.10/2	C	C	C	A	G	A	G	T	A	T	C
2	MuVi/Shandong.CHN/51.15/1	C	C	C	A	G	A	G	T	A	T	C
	MuVi/Hunan.CHN/32.11	C	C	C	A	G	A	G	C	G	T	C
	MuVi/Hunan.CHN/30.11/2	C	C	C	A	G	A	G	C	G	T	C
	MuVi/Heilongjiang.CHN/14.13/1	C	C	C	A	G	A	G	C	G	T	C
	MuVi/Jiangsu.CHN/7.12	C	C	C	A	G	A	G	C	G	T	C
	MuVi/Liaoning.CHN/9.10/1	C	C	C	A	G	A	G	C	G	T	C
	MuVi/Hunan.CHN/30.11/3	C	C	C	A	G	A	G	C	G	T	C
	MuVi/Jiangsu.CHN/12.13/2	C	C	C	A	G	A	G	C	G	T	C
	MuVi/Shannxi.CHN/20.15/1	C	C	C	A	G	A	G	C	G	T	C
	MuVi/Liaoning.CHN/13.12	C	C	C	G	G	A	G	C	G	T	C
	MuVi/Beijing.CHN/21.11	T	C	C	A	G	A	G	C	G	T	C
	MuVi/Liaoning.CHN/11.12	T	C	C	A	G	A	G	C	G	T	C
	MuVi/Jiangsu.CHN/4.13/5	C	C	C	A	G	A	G	C	G	T	C
	MuVi/Heilongjiang.CHN/26.12/1	C	C	C	A	G	A	G	C	G	T	C
	MuVi/Beijing.CHN/17.07/2	C	C	C	A	G	A	G	C	G	T	C
	MuVi/Sichuan.CHN/23.12/2	C	C	C	A	G	A	G	C	G	T	C
	MuVi/Hubei.CHN/44.12/1	C	C	C	A	G	A	G	C	G	T	C
	MuVi/Jiangsu.CHN/2.13/4	C	C	C	A	G	A	G	C	G	T	C
	MuVi/Liaoning.CHN/7.12	C	C	C	A	G	A	G	C	G	T	C
	MuVi/Heilongjiang.CHN/22.12	C	C	C	A	G	A	G	C	G	T	C
	MuVi/Heilongjiang.CHN/15.13	C	C	C	A	G	A	G	C	G	T	C
	MuVi/Liaoning.CHN/5.09/1	C	C	C	A	G	A	G	C	G	T	C
	MuVi/Liaoning.CHN/1.08/2	C	C	C	A	G	A	G	C	G	T	C
	MuVi/Liaoning.CHN/10.09	C	C	C	A	G	A	G	C	G	T	C
	MuVi/Liaoning.CHN/1.08/1	C	C	C	A	G	A	G	C	G	T	C
	MuVi/Liaoning.CHN/48.10/1	C	C	C	A	G	A	G	C	G	T	C
	MuVi/Beijing.CHN/10.07	C	C	C	A	A	A	G	C	A	T	C
	MuVi/Hunan.CHN/30.11/1	C	C	C	A	A	A	G	C	A	T	C
	MuVi/Jiangsu.CHN/7.13/1	C	C	C	A	A	A	G	C	A	T	C
	MuVi/Liaoning.CHN/5.09/2	C	C	C	A	G	A	A	C	A	T	C
	MuVi/Zhejiang.CHN/11.06/1	C	C	C	A	A	A	G	C	A	T	T

1: Lineage based on Figure 1
2: SNPs are noted in green.
3: SNP position corresponding to a lineage-defining node in Figure 1 [illegible]

Supplementary Table S3: Listing of SNPs featured in the 70 F sequences of [illegible]

Lineage [1]	Strain name	1035	1038	1039	1044	1047	1048	1050	1053	1056	1057
1	MuVi/Liaoning.CHN/50.11	A	A	C	C	C	G	A	C	T	A
1	MuVi/Liaoning.CHN/48.11	A	A	C	C	C	G	A	C	T	A
1	MuVi/Heilongjiang.CHN/23.13/1	A	A	C	C	C	G	A	C	T	A
1	MuVi/Shandong.CHN/43.07	A	A	C	C	C	G	A	C	T	A
1	MuVi/Shandong.CHN/10.08	A	A	C	C	C	G	A	C	T	A
1	MuVi/Heilongjiang.CHN/26.12/2	A	A	C	C	C	G	A	C	T	A
1	MuVi/Heilongjiang.CHN/24.14	A	A	C	C	C	G	A	C	T	A
1	MuVi/Sichuan.CHN/24.15	A	A	C	C	C	G	A	C	T	A
1	MuVi/Shandong.CHN/11.07	A	A	C	C	T	G	A	C	T	A
1	MuVi/Beijing.CHN/15.06	A	A	C	C	C	G	A	C	T	A
1	MuVi/Gansu.CHN/0.02	A	A	C	C	T	G	A	C	T	A
1	MuVi/Jiangsu.CHN/3.13/2	A	A	T	C	T	G	A	C	T	A
1	MuVi/Beijing.CHN/25.06	A	A	C	C	T	G	A	T	T	A
1	MuVi/Shanghai.CHN/0.01	A	A	C	T	T	G	A	C	T	A
1	MuVi/Shandong.CHN/3.05	A	A	C	T	T	G	A	C	T	A
1	MuVi/Zhejiang.CHN/26.05	A	A	C	C	T	G	A	C	T	A
1	MuVi/Sichuan.CHN/23.12/1	A	G	C	C	T	G	A	C	T	A
1	MuVi/Neimeng.CHN/18.11	A	G	C	C	T	G	C	C	T	A
1	MuVi/Yunnan.CHN/47.10/2	A	G	C	C	T	G	A	C	T	A
1	MuVi/Jilin.CHN/15.08/3	A	G	C	C	T	G	A	C	T	A
1	MuVi/Jilin.CHN/15.08/1	A	G	C	C	T	G	A	C	T	A
1	MuVi/Jilin.CHN/15.08/5	A	G	C	C	T	G	A	C	T	A
4	MuVi/Shannxi.CHN/26.09/1	A	A	C	C	T	G	A	C	C	A
4	MuVi/Jiangsu.CHN/15.12	A	A	C	C	T	G	A	C	C	A
3	MuVi/Shandong.CHN/4.05	A	A	C	C	T	G	A	C	T	A
3	MuVi/Shannxi.CHN/9.09/2	A	A	C	C	T	G	A	C	T	A
3	MuVi/Shanxi.CHN/52.10/3	A	A	C	C	T	G	A	C	T	G
3	MuVi/Jiangsu.CHN/3.13/1	A	A	C	C	T	G	A	C	T	G
3	MuVi/Henan.CHN/48.06	A	A	C	C	T	G	A	C	T	A
3	MuVi/Jiangsu.CHN/9.12	A	A	C	C	T	G	A	C	T	A
3	MuVi/Jiangsu.CHN/4.13/3	A	A	C	C	T	G	A	C	T	A
2	MuVi/Shanxi.CHN/52.10/2	A	A	C	C	C	G	A	C	T	A
2	MuVi/Shannxi.CHN/26.09/4	A	A	C	C	T	G	A	C	T	A
2	MuVi/Shanxi.CHN/52.10/1	A	A	C	C	T	G	A	C	T	A
2	MuVi/Guangdong.CHN/21.09/1	A	A	C	C	T	G	A	C	T	A
2	MuVi/Heilongjiang.CHN/49.15	A	A	C	C	T	G	A	C	T	A
2	MuVi/Anhui.CHN/10.11/1	A	A	C	C	T	G	A	C	T	A
2	MuVi/Shannxi.CHN/20.12	A	A	C	C	T	G	A	C	T	A
2	MuVi/Liaoning.CHN/48.10/2	A	A	C	C	T	G	A	C	T	A
2	MuVi/Shandong.CHN/51.15/1	A	A	C	C	T	G	A	C	T	A
	MuVi/Hunan.CHN/32.11	A	A	C	C	T	G	A	C	T	A
	MuVi/Hunan.CHN/30.11/2	A	A	C	C	T	G	A	C	T	A
	MuVi/Heilongjiang.CHN/14.13/1	A	A	C	C	T	G	A	C	T	A
	MuVi/Jiangsu.CHN/7.12	A	A	C	C	T	G	A	C	T	A
	MuVi/Liaoning.CHN/9.10/1	A	A	C	C	T	G	A	C	T	A
	MuVi/Hunan.CHN/30.11/3	A	A	C	C	T	G	A	C	T	A
	MuVi/Jiangsu.CHN/12.13/2	A	A	C	C	T	G	A	C	T	A
	MuVi/Shannxi.CHN/20.15/1	A	A	C	C	T	G	A	C	T	A
	MuVi/Liaoning.CHN/13.12	T	A	C	C	T	G	A	A	T	A
	MuVi/Beijing.CHN/21.11	A	A	C	C	T	G	A	A	T	A
	MuVi/Liaoning.CHN/11.12	A	A	C	C	T	G	A	A	T	A
	MuVi/Jiangsu.CHN/4.13/5	A	A	C	C	T	G	A	A	T	A
	MuVi/Heilongjiang.CHN/26.12/1	A	A	C	C	T	G	A	A	T	A
	MuVi/Beijing.CHN/17.07/2	T	A	C	C	T	G	A	A	T	A
	MuVi/Sichuan.CHN/23.12/2	A	A	C	C	T	G	A	A	T	A
	MuVi/Hubei.CHN/44.12/1	A	A	C	C	T	G	A	A	T	A
	MuVi/Jiangsu.CHN/2.13/4	A	A	C	C	T	G	A	A	T	A
	MuVi/Liaoning.CHN/7.12	A	A	C	C	T	G	A	A	T	A
	MuVi/Heilongjiang.CHN/22.12	A	A	C	C	T	G	A	A	T	A
	MuVi/Heilongjiang.CHN/15.13	A	A	C	C	T	G	A	A	T	A
	MuVi/Liaoning.CHN/5.09/1	A	A	C	C	T	G	A	A	T	A
	MuVi/Liaoning.CHN/1.08/2	A	A	C	C	T	G	A	A	T	A
	MuVi/Liaoning.CHN/10.09	A	A	C	C	T	G	A	A	T	A
	MuVi/Liaoning.CHN/1.08/1	A	A	C	C	T	G	A	A	T	A
	MuVi/Liaoning.CHN/48.10/1	A	A	C	C	T	G	A	A	T	A
	MuVi/Beijing.CHN/10.07	A	A	C	C	T	G	A	C	T	A
	MuVi/Hunan.CHN/30.11/1	A	A	C	C	T	G	A	C	T	A
	MuVi/Jiangsu.CHN/7.13/1	A	A	C	C	T	A	A	C	T	A
	MuVi/Liaoning.CHN/5.09/2	A	A	C	T	T	G	A	C	T	A
	MuVi/Zhejiang.CHN/11.06/1	A	A	C	C	T	G	A	C	T	A

1: Lineage based on Figure 1
2: SNPs are noted in green.
3: SNP position corresponding to a lineage-defining node in Figure 1 [illegible]

Supplementary Table S3: Listing of SNPs featured in the 70 F sequences of [illegible]

Lineage [1]	Strain name	1059	1065	1069	1077	1080	1089	1095	1104	1107	1108
1	MuVi/Liaoning.CHN/50.11	A	C	G	A	C	G	T	A	**C**	**A**
1	MuVi/Liaoning.CHN/48.11	A	C	G	A	C	G	T	A	T	G
1	MuVi/Heilongjiang.CHN/23.13/1	A	C	G	A	C	**T**	T	A	T	G
1	MuVi/Shandong.CHN/43.07	A	C	G	A	C	G	T	A	T	G
1	MuVi/Shandong.CHN/10.08	A	C	G	A	C	G	T	A	T	G
1	MuVi/Heilongjiang.CHN/26.12/2	A	C	G	A	C	G	T	A	T	G
1	MuVi/Heilongjiang.CHN/24.14	A	C	G	A	C	G	T	A	T	G
1	MuVi/Sichuan.CHN/24.15	A	C	G	A	C	G	T	A	T	G
1	MuVi/Shandong.CHN/11.07	A	C	G	A	C	G	T	A	T	G
1	MuVi/Beijing.CHN/15.06	A	C	G	A	C	G	T	A	T	G
1	MuVi/Gansu.CHN/0.02	A	C	G	A	C	G	T	A	T	G
1	MuVi/Jiangsu.CHN/3.13/2	A	**T**	G	A	C	G	T	A	T	G
1	MuVi/Beijing.CHN/25.06	A	C	G	A	C	G	T	A	T	G
1	MuVi/Shanghai.CHN/0.01	A	C	G	A	C	G	T	A	T	G
1	MuVi/Shandong.CHN/3.05	A	C	G	A	C	G	T	A	T	G
1	MuVi/Zhejiang.CHN/26.05	A	C	G	A	C	G	T	A	T	G
1	MuVi/Sichuan.CHN/23.12/1	A	C	G	A	C	G	T	A	T	G
1	MuVi/Neimeng.CHN/18.11	A	C	G	A	C	G	T	A	T	G
1	MuVi/Yunnan.CHN/47.10/2	A	C	G	A	C	G	T	A	T	G
1	MuVi/Jilin.CHN/15.08/3	A	C	G	A	C	G	T	**G**	T	G
1	MuVi/Jilin.CHN/15.08/1	A	C	G	A	C	G	T	**G**	T	G
1	MuVi/Jilin.CHN/15.08/5	A	C	G	A	C	G	T	**G**	T	G
4	MuVi/Shannxi.CHN/26.09/1	A	C	G	A	C	G	T	A	T	G
4	MuVi/Jiangsu.CHN/15.12	A	C	G	A	C	G	T	A	T	G
3	MuVi/Shandong.CHN/4.05	A	C	G	A	C	G	T	A	T	G
3	MuVi/Shannxi.CHN/9.09/2	A	C	G	A	C	G	T	A	T	G
3	MuVi/Shanxi.CHN/52.10/3	A	C	G	A	C	G	T	A	T	G
3	MuVi/Jiangsu.CHN/3.13/1	A	C	G	A	C	G	T	A	T	G
3	MuVi/Henan.CHN/48.06	**C**	C	G	A	C	G	T	A	T	G
3	MuVi/Jiangsu.CHN/9.12	**C**	C	G	A	C	G	T	A	T	G
3	MuVi/Jiangsu.CHN/4.13/3	**C**	C	G	A	C	G	T	A	T	G
2	MuVi/Shanxi.CHN/52.10/2	A	C	G	A	**T**	G	T	A	T	G
2	MuVi/Shannxi.CHN/26.09/4	A	C	G	A	C	G	T	A	T	G
2	MuVi/Shanxi.CHN/52.10/1	A	C	G	A	C	G	T	A	T	G
2	MuVi/Guangdong.CHN/21.09/1	A	C	G	**T**	C	G	T	A	T	G
2	MuVi/Heilongjiang.CHN/49.15	A	C	G	A	C	G	T	A	T	G
2	MuVi/Anhui.CHN/10.11/1	A	C	G	A	C	G	T	A	T	G
2	MuVi/Shannxi.CHN/20.12	A	C	G	A	C	G	T	A	T	G
2	MuVi/Liaoning.CHN/48.10/2	A	C	G	A	C	G	T	A	T	G
2	MuVi/Shandong.CHN/51.15/1	A	C	G	A	C	G	T	A	T	G
	MuVi/Hunan.CHN/32.11	A	C	G	A	C	G	T	A	T	G
	MuVi/Hunan.CHN/30.11/2	A	C	G	A	C	G	T	A	T	G
	MuVi/Heilongjiang.CHN/14.13/1	A	C	G	A	C	**A**	T	A	T	G
	MuVi/Jiangsu.CHN/7.12	A	C	G	A	C	**A**	T	A	T	G
	MuVi/Liaoning.CHN/9.10/1	A	C	G	A	C	G	T	A	T	G
	MuVi/Hunan.CHN/30.11/3	A	C	G	A	C	G	T	A	T	G
	MuVi/Jiangsu.CHN/12.13/2	A	C	G	A	C	G	T	A	T	G
	MuVi/Shannxi.CHN/20.15/1	A	C	G	A	C	G	T	A	T	G
	MuVi/Liaoning.CHN/13.12	A	C	G	A	C	G	T	A	T	G
	MuVi/Beijing.CHN/21.11	A	C	G	A	C	G	T	A	T	G
	MuVi/Liaoning.CHN/11.12	A	C	G	A	C	G	T	A	T	G
	MuVi/Jiangsu.CHN/4.13/5	A	C	G	A	C	G	T	A	T	G
	MuVi/Heilongjiang.CHN/26.12/1	A	C	G	A	C	G	T	A	T	G
	MuVi/Beijing.CHN/17.07/2	A	C	G	A	C	G	T	A	T	G
	MuVi/Sichuan.CHN/23.12/2	A	C	**A**	A	C	G	T	A	T	G
	MuVi/Hubei.CHN/44.12/1	A	C	G	A	C	**A**	T	A	T	G
	MuVi/Jiangsu.CHN/2.13/4	A	C	G	A	C	**A**	T	A	T	G
	MuVi/Liaoning.CHN/7.12	A	C	G	A	C	G	T	A	T	G
	MuVi/Heilongjiang.CHN/22.12	A	C	G	A	C	G	T	A	T	G
	MuVi/Heilongjiang.CHN/15.13	A	C	G	A	C	G	T	A	T	G
	MuVi/Liaoning.CHN/5.09/1	A	C	G	A	C	G	T	A	T	G
	MuVi/Liaoning.CHN/1.08/2	A	C	G	A	C	G	T	A	T	G
	MuVi/Liaoning.CHN/10.09	A	C	G	A	C	G	T	A	T	G
	MuVi/Liaoning.CHN/1.08/1	A	C	G	A	C	G	T	A	T	G
	MuVi/Liaoning.CHN/48.10/1	A	C	G	A	C	G	T	A	T	G
	MuVi/Beijing.CHN/10.07	A	C	G	A	C	G	T	A	T	G
	MuVi/Hunan.CHN/30.11/1	A	C	G	A	C	G	**C**	A	T	G
	MuVi/Jiangsu.CHN/7.13/1	A	C	G	A	C	G	**C**	A	T	G
	MuVi/Liaoning.CHN/5.09/2	A	C	G	A	C	G	T	A	T	G
	MuVi/Zhejiang.CHN/11.06/1	A	C	G	A	C	G	T	A	T	G

1: Lineage based on Figure 1
2: SNPs are noted in green.
3: SNP position corresponding to a lineage-defining node in Figure 1 [illegible]

Supplementary Table S3: Listing of SNPs featured in the 70 F sequences of I

Lineage [1]	Strain name	1110	1114	1116	1147	1152	1155	1173	1178	1185	1194
1	MuVi/Liaoning.CHN/50.11	A	C	G	C	G	T	T	C	C	C
1	MuVi/Liaoning.CHN/48.11	A	C	G	C	G	T	T	C	C	C
1	MuVi/Heilongjiang.CHN/23.13/1	A	C	G	C	G	T	T	C	C	C
1	MuVi/Shandong.CHN/43.07	A	C	G	C	G	T	T	C	C	C
1	MuVi/Shandong.CHN/10.08	A	C	G	C	G	T	T	C	C	C
1	MuVi/Heilongjiang.CHN/26.12/2	A	C	G	C	G	T	T	C	C	C
1	MuVi/Heilongjiang.CHN/24.14	A	C	G	C	G	T	T	C	C	C
1	MuVi/Sichuan.CHN/24.15	A	C	G	C	G	T	T	C	C	C
1	MuVi/Shandong.CHN/11.07	A	C	G	C	G	T	T	C	C	C
1	MuVi/Beijing.CHN/15.06	A	C	G	C	G	T	T	C	C	C
1	MuVi/Gansu.CHN/0.02	A	C	G	C	G	T	T	C	C	C
1	MuVi/Jiangsu.CHN/3.13/2	A	C	G	C	G	T	T	C	C	C
1	MuVi/Beijing.CHN/25.06	A	C	G	C	G	T	T	C	C	C
1	MuVi/Shanghai.CHN/0.01	A	C	G	C	G	T	T	C	C	C
1	MuVi/Shandong.CHN/3.05	A	C	G	C	G	T	T	C	C	C
1	MuVi/Zhejiang.CHN/26.05	A	C	G	C	G	T	T	C	C	C
1	MuVi/Sichuan.CHN/23.12/1	A	C	G	C	G	C	T	C	C	C
1	MuVi/Neimeng.CHN/18.11	A	C	G	C	G	T	T	C	C	C
1	MuVi/Yunnan.CHN/47.10/2	A	C	G	C	A	T	T	C	C	C
1	MuVi/Jilin.CHN/15.08/3	A	C	G	C	G	T	T	C	C	C
1	MuVi/Jilin.CHN/15.08/1	A	C	G	C	G	T	T	C	C	C
1	MuVi/Jilin.CHN/15.08/5	A	C	G	C	G	T	T	C	C	C
4	MuVi/Shannxi.CHN/26.09/1	A	C	G	C	G	T	T	C	T	C
4	MuVi/Jiangsu.CHN/15.12	A	C	G	C	G	T	T	C	T	C
3	MuVi/Shandong.CHN/4.05	A	C	G	C	G	T	T	C	C	C
3	MuVi/Shannxi.CHN/9.09/2	A	C	G	C	G	T	T	C	C	C
3	MuVi/Shanxi.CHN/52.10/3	A	C	G	C	G	T	T	C	C	C
3	MuVi/Jiangsu.CHN/3.13/1	A	C	G	C	G	T	T	C	C	C
3	MuVi/Henan.CHN/48.06	A	C	G	C	G	T	C	C	C	C
3	MuVi/Jiangsu.CHN/9.12	A	C	G	C	G	T	C	C	C	C
3	MuVi/Jiangsu.CHN/4.13/3	A	C	G	C	G	T	C	C	C	C
2	MuVi/Shanxi.CHN/52.10/2	A	C	G	C	G	T	T	C	C	T
2	MuVi/Shannxi.CHN/26.09/4	A	C	G	C	G	T	T	C	C	T
2	MuVi/Shanxi.CHN/52.10/1	A	C	G	C	G	T	T	C	C	T
2	MuVi/Guangdong.CHN/21.09/1	A	C	G	C	A	T	T	A	C	T
2	MuVi/Heilongjiang.CHN/49.15	A	C	G	C	G	T	T	C	C	T
2	MuVi/Anhui.CHN/10.11/1	A	C	G	C	G	T	T	C	C	T
2	MuVi/Shannxi.CHN/20.12	A	C	G	C	G	T	T	C	C	T
2	MuVi/Liaoning.CHN/48.10/2	A	C	G	C	G	T	T	C	C	T
2	MuVi/Shandong.CHN/51.15/1	A	C	G	C	G	T	T	C	C	T
	MuVi/Hunan.CHN/32.11	A	C	A	C	G	T	T	C	C	C
	MuVi/Hunan.CHN/30.11/2	G	T	G	C	G	T	T	C	C	C
	MuVi/Heilongjiang.CHN/14.13/1	A	C	G	C	G	T	T	C	C	C
	MuVi/Jiangsu.CHN/7.12	A	C	G	C	G	T	T	C	C	C
	MuVi/Liaoning.CHN/9.10/1	A	C	G	C	G	T	T	C	C	C
	MuVi/Hunan.CHN/30.11/3	A	C	G	C	G	T	T	C	C	C
	MuVi/Jiangsu.CHN/12.13/2	A	C	G	T	G	T	T	C	C	C
	MuVi/Shannxi.CHN/20.15/1	A	C	G	T	G	T	T	C	C	C
	MuVi/Liaoning.CHN/13.12	A	C	G	C	G	T	T	C	C	C
	MuVi/Beijing.CHN/21.11	A	C	G	C	G	T	T	C	C	C
	MuVi/Liaoning.CHN/11.12	A	C	G	C	G	T	T	C	C	C
	MuVi/Jiangsu.CHN/4.13/5	A	C	G	C	A	T	T	C	C	C
	MuVi/Heilongjiang.CHN/26.12/1	A	C	G	C	G	T	T	C	C	C
	MuVi/Beijing.CHN/17.07/2	A	C	G	C	G	T	T	C	C	C
	MuVi/Sichuan.CHN/23.12/2	A	C	G	C	G	T	T	C	C	C
	MuVi/Hubei.CHN/44.12/1	A	C	G	C	G	T	T	C	C	C
	MuVi/Jiangsu.CHN/2.13/4	A	C	G	C	G	T	T	C	C	C
	MuVi/Liaoning.CHN/7.12	A	C	G	C	G	T	T	C	C	C
	MuVi/Heilongjiang.CHN/22.12	A	C	G	C	G	T	T	C	C	C
	MuVi/Heilongjiang.CHN/15.13	A	C	G	C	G	T	T	C	C	C
	MuVi/Liaoning.CHN/5.09/1	A	C	G	C	G	T	T	C	C	C
	MuVi/Liaoning.CHN/1.08/2	A	C	G	C	G	T	T	C	C	C
	MuVi/Liaoning.CHN/10.09	A	C	G	C	G	T	T	C	C	C
	MuVi/Liaoning.CHN/1.08/1	A	C	G	C	G	T	T	C	C	C
	MuVi/Liaoning.CHN/48.10/1	A	C	G	C	G	T	T	C	C	C
	MuVi/Beijing.CHN/10.07	A	C	G	C	G	T	T	C	C	T
	MuVi/Hunan.CHN/30.11/1	A	C	G	C	G	T	T	C	C	C
	MuVi/Jiangsu.CHN/7.13/1	A	C	G	C	G	T	T	C	C	C
	MuVi/Liaoning.CHN/5.09/2	A	C	G	C	G	T	T	C	C	C
	MuVi/Zhejiang.CHN/11.06/1	A	C	G	C	G	T	T	C	C	C

1: Lineage based on Figure 1
2: SNPs are noted in green.
3: SNP position corresponding to a lineage-defining node in Figure 1

Supplementary Table S3: Listing of SNPs featured in the 70 F sequences of I

Lineage [1]	Strain name	1197	1200	1203	1206	1211	1212	1213	1215	1221	1224
1	MuVi/Liaoning.CHN/50.11	T	T	A	C	C	C	A	T	A	C
1	MuVi/Liaoning.CHN/48.11	T	T	A	C	C	C	A	T	A	C
1	MuVi/Heilongjiang.CHN/23.13/1	T	T	A	C	C	C	A	T	A	C
1	MuVi/Shandong.CHN/43.07	T	T	A	C	C	C	A	T	A	C
1	MuVi/Shandong.CHN/10.08	T	T	A	C	C	C	A	T	A	C
1	MuVi/Heilongjiang.CHN/26.12/2	T	T	A	C	C	C	A	T	A	C
1	MuVi/Heilongjiang.CHN/24.14	T	T	A	C	C	C	A	T	A	C
1	MuVi/Sichuan.CHN/24.15	T	T	A	C	C	C	A	T	A	C
1	MuVi/Shandong.CHN/11.07	T	T	A	A	C	C	A	T	A	C
1	MuVi/Beijing.CHN/15.06	T	T	A	A	C	C	A	T	A	C
1	MuVi/Gansu.CHN/0.02	T	T	A	C	C	C	A	T	A	C
1	MuVi/Jiangsu.CHN/3.13/2	T	T	A	C	C	C	A	C	G	C
1	MuVi/Beijing.CHN/25.06	T	T	A	C	C	C	A	C	A	C
1	MuVi/Shanghai.CHN/0.01	T	T	A	C	C	C	A	T	A	C
1	MuVi/Shandong.CHN/3.05	T	T	A	C	C	C	A	T	A	C
1	MuVi/Zhejiang.CHN/26.05	T	T	A	C	C	C	A	T	A	C
1	MuVi/Sichuan.CHN/23.12/1	T	T	A	C	C	C	A	T	A	C
1	MuVi/Neimeng.CHN/18.11	T	T	A	C	C	C	A	T	A	C
1	MuVi/Yunnan.CHN/47.10/2	T	T	A	C	C	C	A	T	A	C
1	MuVi/Jilin.CHN/15.08/3	T	T	A	C	C	C	A	T	A	C
1	MuVi/Jilin.CHN/15.08/1	T	T	A	C	C	C	A	T	A	C
1	MuVi/Jilin.CHN/15.08/5	T	T	A	C	C	C	A	T	A	C
4	MuVi/Shannxi.CHN/26.09/1	T	T	A	C	C	C	A	T	A	C
4	MuVi/Jiangsu.CHN/15.12	T	T	A	C	C	C	A	T	A	C
3	MuVi/Shandong.CHN/4.05	T	T	A	C	C	C	A	T	A	C
3	MuVi/Shannxi.CHN/9.09/2	T	T	A	C	C	C	A	T	A	C
3	MuVi/Shanxi.CHN/52.10/3	T	T	A	T	C	C	A	T	A	C
3	MuVi/Jiangsu.CHN/3.13/1	T	T	A	C	C	C	A	T	A	C
3	MuVi/Henan.CHN/48.06	T	T	A	C	C	C	A	T	A	C
3	MuVi/Jiangsu.CHN/9.12	T	T	A	C	C	C	A	T	A	C
3	MuVi/Jiangsu.CHN/4.13/3	T	T	A	C	C	C	A	T	A	C
2	MuVi/Shanxi.CHN/52.10/2	T	T	A	C	C	C	A	T	A	T
2	MuVi/Shannxi.CHN/26.09/4	T	T	A	C	C	T	A	T	A	T
2	MuVi/Shanxi.CHN/52.10/1	T	T	A	C	C	C	A	T	A	T
2	MuVi/Guangdong.CHN/21.09/1	T	T	A	C	C	C	A	T	A	T
2	MuVi/Heilongjiang.CHN/49.15	T	T	G	C	C	C	A	T	A	T
2	MuVi/Anhui.CHN/10.11/1	T	T	A	C	C	C	A	T	A	T
2	MuVi/Shannxi.CHN/20.12	T	T	A	C	C	C	A	T	A	T
2	MuVi/Liaoning.CHN/48.10/2	T	C	A	C	C	C	A	T	A	T
2	MuVi/Shandong.CHN/51.15/1	T	C	A	C	C	C	A	T	A	T
	MuVi/Hunan.CHN/32.11	T	T	A	C	C	C	A	T	A	C
	MuVi/Hunan.CHN/30.11/2	T	T	A	C	C	C	A	T	A	C
	MuVi/Heilongjiang.CHN/14.13/1	T	T	A	C	C	C	A	T	A	C
	MuVi/Jiangsu.CHN/7.12	T	T	A	C	C	C	A	T	A	C
	MuVi/Liaoning.CHN/9.10/1	T	T	A	C	C	C	A	T	A	C
	MuVi/Hunan.CHN/30.11/3	T	T	A	C	C	C	A	T	A	C
	MuVi/Jiangsu.CHN/12.13/2	T	T	A	C	C	C	A	T	A	C
	MuVi/Shannxi.CHN/20.15/1	T	T	A	C	C	C	A	T	A	C
	MuVi/Liaoning.CHN/13.12	T	T	A	C	T	C	G	T	A	C
	MuVi/Beijing.CHN/21.11	T	T	A	C	C	C	G	T	A	C
	MuVi/Liaoning.CHN/11.12	T	T	A	C	C	C	G	T	A	C
	MuVi/Jiangsu.CHN/4.13/5	T	T	A	C	C	C	G	T	A	C
	MuVi/Heilongjiang.CHN/26.12/1	T	T	A	C	C	C	G	T	A	C
	MuVi/Beijing.CHN/17.07/2	T	T	A	C	C	C	G	T	A	C
	MuVi/Sichuan.CHN/23.12/2	T	T	A	C	C	C	G	T	A	C
	MuVi/Hubei.CHN/44.12/1	T	T	A	C	C	C	G	T	A	C
	MuVi/Jiangsu.CHN/2.13/4	T	T	A	C	C	C	G	T	A	C
	MuVi/Liaoning.CHN/7.12	T	T	A	C	C	C	G	T	A	C
	MuVi/Heilongjiang.CHN/22.12	T	T	A	C	C	C	G	T	A	C
	MuVi/Heilongjiang.CHN/15.13	T	T	A	C	C	C	G	T	A	C
	MuVi/Liaoning.CHN/5.09/1	T	T	A	C	C	C	G	T	A	C
	MuVi/Liaoning.CHN/1.08/2	T	T	A	C	C	C	G	T	A	C
	MuVi/Liaoning.CHN/10.09	T	T	A	C	C	C	G	T	A	C
	MuVi/Liaoning.CHN/1.08/1	T	T	A	C	C	C	G	T	A	C
	MuVi/Liaoning.CHN/48.10/1	T	T	A	C	C	C	G	T	A	C
	MuVi/Beijing.CHN/10.07	T	T	A	C	C	C	A	T	A	C
	MuVi/Hunan.CHN/30.11/1	C	T	A	C	C	C	A	C	A	C
	MuVi/Jiangsu.CHN/7.13/1	C	T	A	C	C	C	A	T	A	C
	MuVi/Liaoning.CHN/5.09/2	T	T	A	C	C	C	A	T	A	C
	MuVi/Zhejiang.CHN/11.06/1	T	T	A	C	C	C	A	T	A	C

1: Lineage based on Figure 1
2: SNPs are noted in green.
3: SNP position corresponding to a lineage-defining node in Figure 1 a

Supplementary Table S3: Listing of SNPs featured in the 70 F sequences of [illegible]

Lineage [1]	Strain name	1225	1226	1227	1233	1242	1243	1247	1251	1260	1263
1	MuVi/Liaoning.CHN/50.11	G	C	A	A	C	C	A	A	C	C
1	MuVi/Liaoning.CHN/48.11	G	C	A	A	C	C	A	A	C	C
1	MuVi/Heilongjiang.CHN/23.13/1	G	C	A	A	C	C	A	A	C	C
1	MuVi/Shandong.CHN/43.07	G	C	A	A	C	C	G	A	C	C
1	MuVi/Shandong.CHN/10.08	G	C	A	A	C	C	A	A	C	C
1	MuVi/Heilongjiang.CHN/26.12/2	G	C	A	A	C	C	A	A	C	C
1	MuVi/Heilongjiang.CHN/24.14	G	C	A	A	C	C	A	A	C	C
1	MuVi/Sichuan.CHN/24.15	G	C	A	A	C	C	A	A	C	C
1	MuVi/Shandong.CHN/11.07	G	C	A	A	C	C	A	A	C	C
1	MuVi/Beijing.CHN/15.06	G	C	A	A	C	C	A	A	C	C
1	MuVi/Gansu.CHN/0.02	G	C	A	A	C	C	A	A	C	C
1	MuVi/Jiangsu.CHN/3.13/2	T	C	A	A	C	C	A	A	C	C
1	MuVi/Beijing.CHN/25.06	G	C	A	A	C	C	A	A	C	C
1	MuVi/Shanghai.CHN/0.01	G	C	A	A	C	C	A	A	C	C
1	MuVi/Shandong.CHN/3.05	G	T	A	A	C	C	A	A	C	C
1	MuVi/Zhejiang.CHN/26.05	G	C	A	A	C	C	A	A	C	C
1	MuVi/Sichuan.CHN/23.12/1	G	C	A	A	C	C	A	A	C	C
1	MuVi/Neimeng.CHN/18.11	G	C	A	A	C	C	A	A	C	C
1	MuVi/Yunnan.CHN/47.10/2	G	C	A	A	C	C	A	A	C	C
1	MuVi/Jilin.CHN/15.08/3	G	C	A	A	C	C	A	A	C	C
1	MuVi/Jilin.CHN/15.08/1	G	C	A	A	C	C	A	A	C	C
1	MuVi/Jilin.CHN/15.08/5	G	C	A	A	C	C	A	A	C	C
4	MuVi/Shannxi.CHN/26.09/1	G	C	A	A	C	C	A	A	C	C
4	MuVi/Jiangsu.CHN/15.12	G	C	A	A	C	C	A	A	C	C
3	MuVi/Shandong.CHN/4.05	G	C	A	A	C	C	A	A	C	C
3	MuVi/Shannxi.CHN/9.09/2	G	C	A	A	C	C	A	T	C	C
3	MuVi/Shanxi.CHN/52.10/3	G	C	A	A	T	C	A	A	C	C
3	MuVi/Jiangsu.CHN/3.13/1	G	C	A	A	T	C	A	A	C	C
3	MuVi/Henan.CHN/48.06	G	C	A	A	C	C	A	A	C	C
3	MuVi/Jiangsu.CHN/9.12	G	C	A	A	C	C	A	A	C	C
3	MuVi/Jiangsu.CHN/4.13/3	G	C	A	A	C	C	A	A	C	C
2	MuVi/Shanxi.CHN/52.10/2	G	C	A	A	C	C	A	A	C	C
2	MuVi/Shannxi.CHN/26.09/4	G	C	A	A	C	C	A	A	C	C
2	MuVi/Shanxi.CHN/52.10/1	G	C	A	A	C	C	A	A	C	C
2	MuVi/Guangdong.CHN/21.09/1	G	C	A	A	C	C	A	A	C	C
2	MuVi/Heilongjiang.CHN/49.15	G	C	A	A	C	C	A	A	C	C
2	MuVi/Anhui.CHN/10.11/1	G	C	A	A	C	C	A	A	C	C
2	MuVi/Shannxi.CHN/20.12	G	C	A	A	C	C	A	A	C	C
2	MuVi/Liaoning.CHN/48.10/2	G	C	A	A	C	C	A	A	C	C
2	MuVi/Shandong.CHN/51.15/1	G	C	A	A	C	C	A	A	C	C
	MuVi/Hunan.CHN/32.11	G	C	A	A	C	C	A	A	C	C
	MuVi/Hunan.CHN/30.11/2	G	C	A	A	C	C	A	A	C	C
	MuVi/Heilongjiang.CHN/14.13/1	G	C	A	A	C	C	A	A	C	C
	MuVi/Jiangsu.CHN/7.12	G	C	A	A	C	C	A	A	C	C
	MuVi/Liaoning.CHN/9.10/1	G	C	A	A	C	C	A	A	C	C
	MuVi/Hunan.CHN/30.11/3	G	C	A	A	C	C	A	A	C	C
	MuVi/Jiangsu.CHN/12.13/2	G	C	A	A	C	C	A	A	C	T
	MuVi/Shannxi.CHN/20.15/1	G	C	A	A	C	C	A	A	C	C
	MuVi/Liaoning.CHN/13.12	G	C	A	A	C	C	A	A	C	C
	MuVi/Beijing.CHN/21.11	G	C	A	G	C	C	A	A	C	C
	MuVi/Liaoning.CHN/11.12	G	C	A	G	C	C	A	A	C	C
	MuVi/Jiangsu.CHN/4.13/5	G	C	A	A	C	C	A	A	C	C
	MuVi/Heilongjiang.CHN/26.12/1	G	C	A	A	C	C	A	A	C	C
	MuVi/Beijing.CHN/17.07/2	G	C	A	A	C	C	A	A	C	C
	MuVi/Sichuan.CHN/23.12/2	G	C	A	A	C	C	A	A	C	C
	MuVi/Hubei.CHN/44.12/1	G	C	A	A	C	C	A	A	C	C
	MuVi/Jiangsu.CHN/2.13/4	G	C	A	A	C	C	A	A	C	C
	MuVi/Liaoning.CHN/7.12	G	C	A	A	C	C	A	A	C	C
	MuVi/Heilongjiang.CHN/22.12	G	C	A	A	C	C	A	A	C	C
	MuVi/Heilongjiang.CHN/15.13	G	C	A	A	C	C	A	A	C	C
	MuVi/Liaoning.CHN/5.09/1	G	C	A	A	C	C	A	A	C	C
	MuVi/Liaoning.CHN/1.08/2	G	C	A	A	C	C	A	A	C	C
	MuVi/Liaoning.CHN/10.09	G	C	A	A	C	C	A	A	C	C
	MuVi/Liaoning.CHN/1.08/1	G	C	A	A	C	C	A	A	C	C
	MuVi/Liaoning.CHN/48.10/1	G	C	A	A	C	C	A	A	C	T
	MuVi/Beijing.CHN/10.07	G	C	A	A	C	T	A	A	C	C
	MuVi/Hunan.CHN/30.11/1	G	C	G	A	C	C	A	A	C	C
	MuVi/Jiangsu.CHN/7.13/1	G	C	G	A	C	C	A	A	C	C
	MuVi/Liaoning.CHN/5.09/2	G	C	A	A	C	C	A	A	T	C
	MuVi/Zhejiang.CHN/11.06/1	G	C	A	A	C	C	A	A	C	C

1: Lineage based on Figure 1
2: SNPs are noted in green.
3: SNP position corresponding to a lineage-defining node in Figure 1 [illegible]

Supplementary Table S3: Listing of SNPs featured in the 70 F sequences of I

Lineage [1]	Strain name	1269	1272	1275	1278	1281	1287	1290	1296	1299	1302
1	MuVi/Liaoning.CHN/50.11	C	T	A	C	C	T	C	G	C	T
1	MuVi/Liaoning.CHN/48.11	C	T	A	C	C	T	C	G	C	T
1	MuVi/Heilongjiang.CHN/23.13/1	C	T	A	C	C	T	C	G	C	T
1	MuVi/Shandong.CHN/43.07	C	T	A	C	C	T	C	G	C	T
1	MuVi/Shandong.CHN/10.08	C	T	A	C	C	T	C	G	C	T
1	MuVi/Heilongjiang.CHN/26.12/2	C	T	A	C	C	T	C	G	C	T
1	MuVi/Heilongjiang.CHN/24.14	C	T	A	C	C	T	C	G	C	T
1	MuVi/Sichuan.CHN/24.15	C	T	A	C	T	T	C	G	C	T
1	MuVi/Shandong.CHN/11.07	C	T	A	C	C	T	C	G	C	T
1	MuVi/Beijing.CHN/15.06	C	T	A	C	C	T	C	G	C	T
1	MuVi/Gansu.CHN/0.02	C	T	A	C	C	T	C	G	C	T
1	MuVi/Jiangsu.CHN/3.13/2	C	T	A	C	C	T	C	G	C	T
1	MuVi/Beijing.CHN/25.06	C	T	A	C	C	T	C	G	C	T
1	MuVi/Shanghai.CHN/0.01	C	T	A	C	C	T	C	G	C	G
1	MuVi/Shandong.CHN/3.05	C	T	A	C	C	T	C	G	C	T
1	MuVi/Zhejiang.CHN/26.05	C	T	A	C	C	T	C	G	C	T
1	MuVi/Sichuan.CHN/23.12/1	C	C	A	C	C	T	C	G	C	T
1	MuVi/Neimeng.CHN/18.11	C	C	A	C	C	T	C	G	C	T
1	MuVi/Yunnan.CHN/47.10/2	C	C	A	C	C	T	C	G	C	T
1	MuVi/Jilin.CHN/15.08/3	C	C	A	C	C	T	C	G	C	T
1	MuVi/Jilin.CHN/15.08/1	C	C	A	C	C	T	C	G	C	T
1	MuVi/Jilin.CHN/15.08/5	C	C	A	C	C	T	C	G	C	T
4	MuVi/Shannxi.CHN/26.09/1	C	T	A	C	C	T	C	G	C	T
4	MuVi/Jiangsu.CHN/15.12	C	T	A	C	C	T	C	G	C	T
3	MuVi/Shandong.CHN/4.05	C	T	A	C	C	T	C	G	C	T
3	MuVi/Shannxi.CHN/9.09/2	C	T	A	C	C	T	C	G	C	T
3	MuVi/Shanxi.CHN/52.10/3	C	T	A	C	C	T	C	G	C	T
3	MuVi/Jiangsu.CHN/3.13/1	C	T	A	C	C	T	C	G	C	T
3	MuVi/Henan.CHN/48.06	C	T	A	C	C	T	C	G	C	T
3	MuVi/Jiangsu.CHN/9.12	C	T	A	C	C	T	T	G	C	T
3	MuVi/Jiangsu.CHN/4.13/3	C	T	A	C	C	T	T	G	C	T
2	MuVi/Shanxi.CHN/52.10/2	C	T	A	C	C	T	C	G	C	T
2	MuVi/Shannxi.CHN/26.09/4	C	T	A	C	C	T	C	G	C	T
2	MuVi/Shanxi.CHN/52.10/1	C	T	A	C	C	T	C	G	C	T
2	MuVi/Guangdong.CHN/21.09/1	C	T	A	C	C	T	C	G	C	T
2	MuVi/Heilongjiang.CHN/49.15	C	T	A	C	C	T	C	G	C	T
2	MuVi/Anhui.CHN/10.11/1	C	T	A	C	C	T	C	G	C	T
2	MuVi/Shannxi.CHN/20.12	C	T	A	C	C	T	C	G	C	T
2	MuVi/Liaoning.CHN/48.10/2	C	T	A	C	C	T	C	G	C	T
2	MuVi/Shandong.CHN/51.15/1	C	T	A	C	C	T	C	G	C	T
	MuVi/Hunan.CHN/32.11	C	T	A	C	C	T	C	G	C	T
	MuVi/Hunan.CHN/30.11/2	C	T	A	C	C	T	C	G	C	T
	MuVi/Heilongjiang.CHN/14.13/1	C	T	A	C	C	C	C	A	C	T
	MuVi/Jiangsu.CHN/7.12	C	T	A	C	C	C	C	A	C	T
	MuVi/Liaoning.CHN/9.10/1	C	T	A	C	C	C	C	A	C	T
	MuVi/Hunan.CHN/30.11/3	C	T	A	C	C	C	C	A	C	T
	MuVi/Jiangsu.CHN/12.13/2	C	T	A	C	C	T	C	G	C	T
	MuVi/Shannxi.CHN/20.15/1	C	T	A	C	C	T	C	G	C	T
	MuVi/Liaoning.CHN/13.12	T	T	A	C	C	T	C	G	C	T
	MuVi/Beijing.CHN/21.11	C	T	G	C	C	T	C	G	C	T
	MuVi/Liaoning.CHN/11.12	C	T	G	C	C	T	C	G	C	T
	MuVi/Jiangsu.CHN/4.13/5	C	T	A	T	C	T	C	G	C	T
	MuVi/Heilongjiang.CHN/26.12/1	C	T	G	C	C	T	C	G	C	T
	MuVi/Beijing.CHN/17.07/2	C	T	A	C	C	T	C	G	C	T
	MuVi/Sichuan.CHN/23.12/2	C	T	A	C	C	T	C	G	C	T
	MuVi/Hubei.CHN/44.12/1	C	T	A	C	C	T	C	G	T	T
	MuVi/Jiangsu.CHN/2.13/4	C	T	A	C	C	T	C	G	T	T
	MuVi/Liaoning.CHN/7.12	C	T	A	C	C	T	T	G	C	T
	MuVi/Heilongjiang.CHN/22.12	C	T	A	C	C	T	C	G	C	T
	MuVi/Heilongjiang.CHN/15.13	C	T	A	C	C	T	C	G	C	T
	MuVi/Liaoning.CHN/5.09/1	C	T	A	C	C	T	C	G	C	T
	MuVi/Liaoning.CHN/1.08/2	C	T	A	C	C	T	C	G	C	T
	MuVi/Liaoning.CHN/10.09	C	T	A	C	C	T	C	G	C	T
	MuVi/Liaoning.CHN/1.08/1	C	T	A	C	C	T	C	G	C	T
	MuVi/Liaoning.CHN/48.10/1	C	T	A	C	C	T	C	G	C	T
	MuVi/Beijing.CHN/10.07	C	T	A	C	C	T	C	G	C	T
	MuVi/Hunan.CHN/30.11/1	C	T	A	C	C	T	C	G	C	T
	MuVi/Jiangsu.CHN/7.13/1	C	T	A	C	C	T	C	G	C	T
	MuVi/Liaoning.CHN/5.09/2	C	T	A	C	C	T	C	G	C	T
	MuVi/Zhejiang.CHN/11.06/1	C	T	A	C	C	T	C	G	C	T

1: Lineage based on Figure 1
2: SNPs are noted in green.
3: SNP position corresponding to a lineage-defining node in Figure 1 a

Supplementary Table S3: Listing of SNPs featured in the 70 F sequences of [illegible]

Lineage [1]	Strain name	1305	1309	1311	1314	1323	1326	1329	1330	1331	1336
1	MuVi/Liaoning.CHN/50.11	C	T	A	G	G	C	C	A	C	C
1	MuVi/Liaoning.CHN/48.11	C	T	A	G	G	C	C	A	C	C
1	MuVi/Heilongjiang.CHN/23.13/1	C	T	A	G	G	C	C	A	T	C
1	MuVi/Shandong.CHN/43.07	C	T	A	G	A	C	C	A	C	C
1	MuVi/Shandong.CHN/10.08	C	T	A	G	A	C	C	A	C	C
1	MuVi/Heilongjiang.CHN/26.12/2	C	T	A	G	A	C	C	A	C	C
1	MuVi/Heilongjiang.CHN/24.14	C	T	A	G	A	C	C	A	C	C
1	MuVi/Sichuan.CHN/24.15	C	T	A	G	A	C	C	A	C	C
1	MuVi/Shandong.CHN/11.07	C	T	G	A	A	C	C	A	C	C
1	MuVi/Beijing.CHN/15.06	C	T	A	G	A	C	C	A	C	C
1	MuVi/Gansu.CHN/0.02	C	T	A	G	A	C	T	A	C	C
1	MuVi/Jiangsu.CHN/3.13/2	C	G	A	G	A	C	T	G	C	C
1	MuVi/Beijing.CHN/25.06	C	T	A	G	A	C	T	G	C	C
1	MuVi/Shanghai.CHN/0.01	C	T	A	G	A	C	T	A	C	C
1	MuVi/Shandong.CHN/3.05	C	T	A	G	A	C	T	A	C	C
1	MuVi/Zhejiang.CHN/26.05	C	T	A	G	A	C	T	A	C	C
1	MuVi/Sichuan.CHN/23.12/1	C	T	A	G	A	C	T	A	C	C
1	MuVi/Neimeng.CHN/18.11	C	T	A	G	A	C	T	A	C	C
1	MuVi/Yunnan.CHN/47.10/2	C	T	A	G	A	C	T	A	C	C
1	MuVi/Jilin.CHN/15.08/3	C	T	A	G	A	C	T	A	C	C
1	MuVi/Jilin.CHN/15.08/1	C	T	A	G	A	C	T	A	C	C
1	MuVi/Jilin.CHN/15.08/5	C	T	A	G	A	C	T	A	C	C
4	MuVi/Shannxi.CHN/26.09/1	C	T	A	G	A	C	T	A	C	C
4	MuVi/Jiangsu.CHN/15.12	C	T	A	G	A	C	T	A	C	C
3	MuVi/Shandong.CHN/4.05	C	T	A	G	A	C	T	A	C	C
3	MuVi/Shannxi.CHN/9.09/2	C	T	A	G	A	C	T	A	C	C
3	MuVi/Shanxi.CHN/52.10/3	C	T	A	A	A	C	T	A	C	C
3	MuVi/Jiangsu.CHN/3.13/1	C	T	A	A	A	C	T	A	C	C
3	MuVi/Henan.CHN/48.06	C	T	A	G	A	C	T	A	C	C
3	MuVi/Jiangsu.CHN/9.12	C	T	A	G	A	C	T	A	C	C
3	MuVi/Jiangsu.CHN/4.13/3	C	T	A	G	A	C	T	A	C	C
2	MuVi/Shanxi.CHN/52.10/2	C	T	A	G	A	C	T	A	C	C
2	MuVi/Shannxi.CHN/26.09/4	C	T	A	G	A	C	T	A	C	C
2	MuVi/Shanxi.CHN/52.10/1	C	T	A	G	A	C	T	A	C	C
2	MuVi/Guangdong.CHN/21.09/1	C	T	A	G	A	C	T	A	C	C
2	MuVi/Heilongjiang.CHN/49.15	C	T	A	G	A	C	C	A	C	C
2	MuVi/Anhui.CHN/10.11/1	C	T	A	G	A	C	T	A	C	C
2	MuVi/Shannxi.CHN/20.12	C	T	A	G	A	C	T	A	C	C
2	MuVi/Liaoning.CHN/48.10/2	C	T	A	G	A	C	T	A	C	C
2	MuVi/Shandong.CHN/51.15/1	C	T	A	G	A	C	T	A	C	C
	MuVi/Hunan.CHN/32.11	C	T	A	G	A	T	T	A	C	C
	MuVi/Hunan.CHN/30.11/2	T	T	A	G	A	C	T	A	C	C
	MuVi/Heilongjiang.CHN/14.13/1	C	T	A	G	A	C	T	A	C	C
	MuVi/Jiangsu.CHN/7.12	C	T	A	G	A	C	T	A	C	C
	MuVi/Liaoning.CHN/9.10/1	C	T	A	G	A	C	T	A	C	C
	MuVi/Hunan.CHN/30.11/3	C	T	A	G	A	C	T	A	C	C
	MuVi/Jiangsu.CHN/12.13/2	C	T	A	G	A	C	T	A	C	C
	MuVi/Shannxi.CHN/20.15/1	C	T	A	G	A	C	T	A	C	C
	MuVi/Liaoning.CHN/13.12	C	T	A	G	A	C	T	A	C	C
	MuVi/Beijing.CHN/21.11	C	T	A	G	A	C	T	A	C	C
	MuVi/Liaoning.CHN/11.12	C	T	A	G	A	C	T	A	C	C
	MuVi/Jiangsu.CHN/4.13/5	C	T	A	G	A	C	T	A	C	C
	MuVi/Heilongjiang.CHN/26.12/1	C	T	A	G	A	C	T	A	C	C
	MuVi/Beijing.CHN/17.07/2	C	T	A	G	A	C	T	A	C	C
	MuVi/Sichuan.CHN/23.12/2	C	T	A	G	A	C	T	A	C	C
	MuVi/Hubei.CHN/44.12/1	C	T	A	G	A	C	T	A	C	C
	MuVi/Jiangsu.CHN/2.13/4	C	T	A	G	A	C	T	A	C	C
	MuVi/Liaoning.CHN/7.12	C	T	A	G	A	C	T	A	C	C
	MuVi/Heilongjiang.CHN/22.12	C	T	A	G	A	C	T	A	C	C
	MuVi/Heilongjiang.CHN/15.13	C	T	A	G	A	C	T	A	C	C
	MuVi/Liaoning.CHN/5.09/1	C	T	A	G	A	C	T	A	C	C
	MuVi/Liaoning.CHN/1.08/2	C	T	A	G	A	C	T	A	C	C
	MuVi/Liaoning.CHN/10.09	C	T	A	G	A	C	T	A	C	C
	MuVi/Liaoning.CHN/1.08/1	C	T	A	G	A	C	T	A	C	T
	MuVi/Liaoning.CHN/48.10/1	C	T	A	G	A	C	T	A	C	C
	MuVi/Beijing.CHN/10.07	C	T	A	A	A	C	T	A	C	C
	MuVi/Hunan.CHN/30.11/1	C	T	A	G	A	C	T	A	C	C
	MuVi/Jiangsu.CHN/7.13/1	C	T	A	G	A	C	T	A	C	C
	MuVi/Liaoning.CHN/5.09/2	C	T	A	G	A	C	T	A	C	C
	MuVi/Zhejiang.CHN/11.06/1	C	T	A	G	A	C	T	A	C	C

1: Lineage based on Figure 1
2: SNPs are noted in green.
3: SNP position corresponding to a lineage-defining node in Figure 1 [illegible]

Supplementary Table S3: Listing of SNPs featured in the 70 F sequences of [illegible]

Lineage [1]	Strain name	1338	1341	1350	1353	1356	1357	1359	1365	1368	1377
1	MuVi/Liaoning.CHN/50.11	C	T	A	T	A	C	A	A	T	C
1	MuVi/Liaoning.CHN/48.11	C	T	A	T	A	C	A	A	T	C
1	MuVi/Heilongjiang.CHN/23.13/1	C	T	A	T	A	C	A	A	T	C
1	MuVi/Shandong.CHN/43.07	C	T	A	T	A	C	A	A	T	C
1	MuVi/Shandong.CHN/10.08	C	T	A	T	A	C	A	A	T	C
1	MuVi/Heilongjiang.CHN/26.12/2	C	T	A	T	A	C	A	A	T	C
1	MuVi/Heilongjiang.CHN/24.14	C	T	A	T	A	C	A	A	T	C
1	MuVi/Sichuan.CHN/24.15	C	T	A	T	A	C	A	A	T	C
1	MuVi/Shandong.CHN/11.07	C	T	A	T	A	C	A	A	T	C
1	MuVi/Beijing.CHN/15.06	C	T	A	T	A	T	A	A	T	C
1	MuVi/Gansu.CHN/0.02	C	T	A	T	A	C	A	A	T	C
1	MuVi/Jiangsu.CHN/3.13/2	A	T	A	T	A	C	A	A	T	C
1	MuVi/Beijing.CHN/25.06	C	T	A	T	A	C	A	A	T	C
1	MuVi/Shanghai.CHN/0.01	C	T	A	T	A	C	A	A	T	C
1	MuVi/Shandong.CHN/3.05	C	T	A	T	A	C	A	A	T	C
1	MuVi/Zhejiang.CHN/26.05	C	C	A	T	A	C	A	A	T	C
1	MuVi/Sichuan.CHN/23.12/1	C	T	A	T	A	C	A	A	T	C
1	MuVi/Neimeng.CHN/18.11	C	T	A	T	A	C	A	A	T	C
1	MuVi/Yunnan.CHN/47.10/2	C	T	A	T	A	C	A	A	T	C
1	MuVi/Jilin.CHN/15.08/3	C	T	A	T	A	C	A	A	T	C
1	MuVi/Jilin.CHN/15.08/1	C	T	A	T	A	C	A	A	T	C
1	MuVi/Jilin.CHN/15.08/5	C	T	A	T	A	C	A	A	T	C
4	MuVi/Shannxi.CHN/26.09/1	C	T	A	T	A	C	A	A	T	C
4	MuVi/Jiangsu.CHN/15.12	C	T	A	T	A	C	A	A	T	C
3	MuVi/Shandong.CHN/4.05	C	T	A	T	A	C	A	A	T	C
3	MuVi/Shannxi.CHN/9.09/2	C	T	A	T	A	C	A	A	T	C
3	MuVi/Shanxi.CHN/52.10/3	C	T	A	T	A	C	A	G	T	C
3	MuVi/Jiangsu.CHN/3.13/1	C	T	A	T	A	C	A	A	T	C
3	MuVi/Henan.CHN/48.06	C	T	A	T	A	C	A	A	T	C
3	MuVi/Jiangsu.CHN/9.12	C	T	A	T	A	C	A	A	T	C
3	MuVi/Jiangsu.CHN/4.13/3	C	T	A	T	A	C	A	A	T	C
2	MuVi/Shanxi.CHN/52.10/2	C	T	A	T	A	C	A	A	T	C
2	MuVi/Shannxi.CHN/26.09/4	C	T	A	T	A	C	A	A	T	C
2	MuVi/Shanxi.CHN/52.10/1	C	T	A	A	A	C	A	A	T	C
2	MuVi/Guangdong.CHN/21.09/1	C	T	A	A	A	C	A	A	T	C
2	MuVi/Heilongjiang.CHN/49.15	C	T	A	A	A	C	A	A	T	C
2	MuVi/Anhui.CHN/10.11/1	C	T	A	A	A	C	A	A	T	C
2	MuVi/Shannxi.CHN/20.12	C	T	A	A	A	C	A	A	T	C
2	MuVi/Liaoning.CHN/48.10/2	C	T	A	A	A	C	A	A	T	C
2	MuVi/Shandong.CHN/51.15/1	C	T	A	A	A	C	A	A	T	C
	MuVi/Hunan.CHN/32.11	C	T	A	T	A	C	A	A	T	C
	MuVi/Hunan.CHN/30.11/2	C	T	A	T	A	C	A	A	T	C
	MuVi/Heilongjiang.CHN/14.13/1	C	T	G	T	A	C	A	A	T	C
	MuVi/Jiangsu.CHN/7.12	C	T	G	T	A	C	A	A	T	C
	MuVi/Liaoning.CHN/9.10/1	C	T	G	T	A	C	A	A	T	C
	MuVi/Hunan.CHN/30.11/3	C	T	G	T	A	C	A	A	T	C
	MuVi/Jiangsu.CHN/12.13/2	C	T	A	T	A	C	A	A	T	C
	MuVi/Shannxi.CHN/20.15/1	C	T	A	T	A	C	A	A	T	C
	MuVi/Liaoning.CHN/13.12	C	T	A	T	A	C	A	A	T	C
	MuVi/Beijing.CHN/21.11	C	T	A	T	A	C	A	A	T	C
	MuVi/Liaoning.CHN/11.12	C	T	A	T	C	C	A	A	T	C
	MuVi/Jiangsu.CHN/4.13/5	C	T	A	T	A	C	A	A	G	T
	MuVi/Heilongjiang.CHN/26.12/1	C	T	A	T	A	C	A	A	T	C
	MuVi/Beijing.CHN/17.07/2	C	T	A	T	A	C	A	A	T	C
	MuVi/Sichuan.CHN/23.12/2	C	T	A	T	A	C	A	A	T	C
	MuVi/Hubei.CHN/44.12/1	C	T	A	T	A	C	A	A	T	C
	MuVi/Jiangsu.CHN/2.13/4	C	T	A	T	A	C	A	A	T	C
	MuVi/Liaoning.CHN/7.12	C	T	A	T	A	C	A	A	T	C
	MuVi/Heilongjiang.CHN/22.12	C	T	A	T	A	C	A	A	T	C
	MuVi/Heilongjiang.CHN/15.13	C	T	A	T	A	C	A	A	T	C
	MuVi/Liaoning.CHN/5.09/1	C	T	A	T	A	C	A	A	T	C
	MuVi/Liaoning.CHN/1.08/2	C	T	A	T	A	C	A	A	T	C
	MuVi/Liaoning.CHN/10.09	C	T	A	T	A	C	A	A	T	C
	MuVi/Liaoning.CHN/1.08/1	C	T	A	T	A	C	A	A	T	C
	MuVi/Liaoning.CHN/48.10/1	C	T	A	T	A	C	A	A	T	C
	MuVi/Beijing.CHN/10.07	C	T	A	T	A	C	A	A	T	C
	MuVi/Hunan.CHN/30.11/1	C	T	A	T	A	C	G	A	T	C
	MuVi/Jiangsu.CHN/7.13/1	C	T	A	T	A	C	G	A	T	C
	MuVi/Liaoning.CHN/5.09/2	C	T	A	T	A	C	A	A	T	C
	MuVi/Zhejiang.CHN/11.06/1	C	T	A	T	A	C	A	A	T	C

1: Lineage based on Figure 1
2: SNPs are noted in green.
3: SNP position corresponding to a lineage-defining node in Figure 1 [illegible]

Supplementary Table S3: Listing of SNPs featured in the 70 F sequences of I

Lineage [1]	Strain name	1380	1384	1389	1392	1395	1398	1403	1404	1410	1413
1	MuVi/Liaoning.CHN/50.11	C	A	T	T	G	C	A	A	C	C
1	MuVi/Liaoning.CHN/48.11	C	A	T	T	G	C	A	A	C	C
1	MuVi/Heilongjiang.CHN/23.13/1	C	A	T	T	G	C	A	A	C	C
1	MuVi/Shandong.CHN/43.07	C	A	T	T	G	C	A	A	C	C
1	MuVi/Shandong.CHN/10.08	C	A	T	T	G	C	A	A	C	C
1	MuVi/Heilongjiang.CHN/26.12/2	C	A	T	T	G	C	A	A	C	C
1	MuVi/Heilongjiang.CHN/24.14	C	A	T	G	A	C	A	A	C	C
1	MuVi/Sichuan.CHN/24.15	C	A	T	G	A	C	A	A	C	C
1	MuVi/Shandong.CHN/11.07	C	A	T	T	G	C	A	A	C	C
1	MuVi/Beijing.CHN/15.06	C	A	T	T	G	C	A	A	C	C
1	MuVi/Gansu.CHN/0.02	C	A	T	T	G	C	A	A	C	C
1	MuVi/Jiangsu.CHN/3.13/2	C	A	T	T	G	C	A	A	C	C
1	MuVi/Beijing.CHN/25.06	C	A	T	T	G	C	A	A	C	C
1	MuVi/Shanghai.CHN/0.01	C	A	T	C	G	C	A	A	C	C
1	MuVi/Shandong.CHN/3.05	C	A	T	T	G	C	A	A	C	C
1	MuVi/Zhejiang.CHN/26.05	C	A	T	T	G	C	A	A	C	C
1	MuVi/Sichuan.CHN/23.12/1	C	A	T	T	G	C	A	A	C	C
1	MuVi/Neimeng.CHN/18.11	C	A	T	T	G	C	A	A	C	C
1	MuVi/Yunnan.CHN/47.10/2	C	A	T	T	G	C	A	A	C	C
1	MuVi/Jilin.CHN/15.08/3	C	A	T	T	G	C	A	A	C	C
1	MuVi/Jilin.CHN/15.08/1	C	A	T	T	G	C	A	A	C	C
1	MuVi/Jilin.CHN/15.08/5	C	A	T	T	G	C	A	A	C	C
4	MuVi/Shannxi.CHN/26.09/1	C	A	C	T	G	C	A	A	C	C
4	MuVi/Jiangsu.CHN/15.12	C	A	C	T	G	C	A	A	C	C
3	MuVi/Shandong.CHN/4.05	C	A	C	T	G	C	A	A	C	C
3	MuVi/Shannxi.CHN/9.09/2	C	A	C	T	G	C	A	A	C	C
3	MuVi/Shanxi.CHN/52.10/3	C	A	C	T	G	C	A	A	C	C
3	MuVi/Jiangsu.CHN/3.13/1	C	A	C	T	G	C	A	A	C	C
3	MuVi/Henan.CHN/48.06	C	A	C	T	G	C	A	A	C	C
3	MuVi/Jiangsu.CHN/9.12	C	A	C	T	G	C	A	A	C	C
3	MuVi/Jiangsu.CHN/4.13/3	C	A	C	C	G	C	A	A	C	C
2	MuVi/Shanxi.CHN/52.10/2	C	A	C	T	G	C	A	A	C	T
2	MuVi/Shannxi.CHN/26.09/4	C	A	C	T	G	T	A	A	C	C
2	MuVi/Shanxi.CHN/52.10/1	C	A	C	T	G	C	A	A	C	C
2	MuVi/Guangdong.CHN/21.09/1	C	A	C	T	G	C	A	A	C	C
2	MuVi/Heilongjiang.CHN/49.15	G	A	C	T	G	C	A	A	C	C
2	MuVi/Anhui.CHN/10.11/1	C	A	C	T	G	C	A	A	C	C
2	MuVi/Shannxi.CHN/20.12	C	A	C	T	G	C	A	A	C	C
2	MuVi/Liaoning.CHN/48.10/2	C	A	C	T	G	C	A	A	C	C
2	MuVi/Shandong.CHN/51.15/1	C	A	C	T	G	C	A	A	C	C
	MuVi/Hunan.CHN/32.11	C	A	C	T	G	C	A	A	C	C
	MuVi/Hunan.CHN/30.11/2	C	A	C	T	G	C	A	A	C	C
	MuVi/Heilongjiang.CHN/14.13/1	C	A	T	T	G	C	A	A	C	C
	MuVi/Jiangsu.CHN/7.12	C	A	C	T	G	C	A	A	C	C
	MuVi/Liaoning.CHN/9.10/1	C	A	C	T	G	C	A	A	C	C
	MuVi/Hunan.CHN/30.11/3	C	A	C	T	G	C	A	A	C	C
	MuVi/Jiangsu.CHN/12.13/2	C	A	C	T	G	C	A	A	C	C
	MuVi/Shannxi.CHN/20.15/1	C	A	C	T	G	C	A	A	C	C
	MuVi/Liaoning.CHN/13.12	C	A	C	T	G	C	A	A	C	C
	MuVi/Beijing.CHN/21.11	C	A	C	T	G	C	A	A	C	C
	MuVi/Liaoning.CHN/11.12	C	A	C	T	G	C	A	A	C	C
	MuVi/Jiangsu.CHN/4.13/5	C	A	T	T	G	C	A	A	C	C
	MuVi/Heilongjiang.CHN/26.12/1	C	A	C	T	G	C	A	A	C	C
	MuVi/Beijing.CHN/17.07/2	C	A	C	T	G	C	A	A	C	C
	MuVi/Sichuan.CHN/23.12/2	C	A	C	T	G	C	A	A	C	C
	MuVi/Hubei.CHN/44.12/1	C	A	C	T	G	C	A	A	C	C
	MuVi/Jiangsu.CHN/2.13/4	C	A	C	T	G	C	A	A	C	C
	MuVi/Liaoning.CHN/7.12	C	A	C	T	G	C	A	A	C	C
	MuVi/Heilongjiang.CHN/22.12	C	A	C	T	G	C	A	A	C	C
	MuVi/Heilongjiang.CHN/15.13	C	A	C	T	G	C	A	A	C	C
	MuVi/Liaoning.CHN/5.09/1	C	A	C	T	G	C	A	A	C	C
	MuVi/Liaoning.CHN/1.08/2	C	A	C	T	G	C	A	A	C	C
	MuVi/Liaoning.CHN/10.09	C	A	C	T	G	C	A	A	T	C
	MuVi/Liaoning.CHN/1.08/1	C	A	C	T	G	C	A	A	C	C
	MuVi/Liaoning.CHN/48.10/1	C	A	C	T	G	C	A	A	C	C
	MuVi/Beijing.CHN/10.07	C	A	C	T	G	C	A	A	C	C
	MuVi/Hunan.CHN/30.11/1	C	A	C	T	G	C	A	G	C	C
	MuVi/Jiangsu.CHN/7.13/1	C	A	C	T	G	C	G	G	C	C
	MuVi/Liaoning.CHN/5.09/2	C	G	C	T	G	C	A	A	C	C
	MuVi/Zhejiang.CHN/11.06/1	C	A	C	T	G	C	A	A	C	C

1: Lineage based on Figure 1
2: SNPs are noted in green.
3: SNP position corresponding to a lineage-defining node in Figure 1 a

Supplementary Table S3: Listing of SNPs featured in the 70 F sequences of [illegible]

Lineage [1]	Strain name	1418	1419	1422	1428	1431	1437	1443	1449	1450	1458
1	MuVi/Liaoning.CHN/50.11	A	A	C	T	G	A	C	C	G	A
1	MuVi/Liaoning.CHN/48.11	A	A	C	T	G	A	C	C	G	A
1	MuVi/Heilongjiang.CHN/23.13/1	A	A	C	T	G	A	C	C	G	A
1	MuVi/Shandong.CHN/43.07	A	A	C	T	G	A	C	C	G	A
1	MuVi/Shandong.CHN/10.08	A	A	C	T	G	A	C	C	G	A
1	MuVi/Heilongjiang.CHN/26.12/2	A	A	C	T	G	A	C	C	G	A
1	MuVi/Heilongjiang.CHN/24.14	A	A	C	T	G	A	C	C	G	A
1	MuVi/Sichuan.CHN/24.15	A	A	C	T	G	A	C	C	G	A
1	MuVi/Shandong.CHN/11.07	A	A	C	T	G	A	C	C	G	A
1	MuVi/Beijing.CHN/15.06	A	A	C	T	G	A	C	C	G	A
1	MuVi/Gansu.CHN/0.02	A	A	C	T	G	A	C	C	G	A
1	MuVi/Jiangsu.CHN/3.13/2	A	A	C	G	G	A	C	C	G	A
1	MuVi/Beijing.CHN/25.06	A	A	C	G	G	A	C	C	G	A
1	MuVi/Shanghai.CHN/0.01	A	A	C	G	G	A	C	C	G	A
1	MuVi/Shandong.CHN/3.05	A	A	C	G	G	A	C	C	G	A
1	MuVi/Zhejiang.CHN/26.05	A	A	C	G	G	A	C	C	G	A
1	MuVi/Sichuan.CHN/23.12/1	A	A	C	G	G	A	C	C	G	A
1	MuVi/Neimeng.CHN/18.11	A	A	C	G	G	A	C	C	G	A
1	MuVi/Yunnan.CHN/47.10/2	A	A	C	G	A	A	C	C	G	A
1	MuVi/Jilin.CHN/15.08/3	A	A	C	G	G	A	C	C	G	A
1	MuVi/Jilin.CHN/15.08/1	A	A	C	G	G	A	C	C	G	A
1	MuVi/Jilin.CHN/15.08/5	A	A	C	G	G	A	C	C	G	A
4	MuVi/Shannxi.CHN/26.09/1	A	A	C	T	G	A	C	C	G	A
4	MuVi/Jiangsu.CHN/15.12	A	A	C	T	G	A	C	C	G	A
3	MuVi/Shandong.CHN/4.05	A	A	C	T	G	A	T	C	G	A
3	MuVi/Shannxi.CHN/9.09/2	A	A	C	T	G	A	C	C	G	A
3	MuVi/Shanxi.CHN/52.10/3	A	A	C	T	G	A	C	C	G	A
3	MuVi/Jiangsu.CHN/3.13/1	A	A	C	T	G	A	C	C	G	A
3	MuVi/Henan.CHN/48.06	A	A	C	T	G	A	C	C	G	A
3	MuVi/Jiangsu.CHN/9.12	A	A	C	T	G	A	C	C	G	A
3	MuVi/Jiangsu.CHN/4.13/3	A	A	C	T	G	A	C	C	G	A
2	MuVi/Shanxi.CHN/52.10/2	A	A	C	T	G	A	C	C	G	A
2	MuVi/Shannxi.CHN/26.09/4	A	A	C	T	G	A	C	C	G	A
2	MuVi/Shanxi.CHN/52.10/1	A	G	C	T	G	A	C	A	G	A
2	MuVi/Guangdong.CHN/21.09/1	A	A	C	T	G	A	C	C	G	A
2	MuVi/Heilongjiang.CHN/49.15	A	A	C	T	G	A	C	C	G	A
2	MuVi/Anhui.CHN/10.11/1	A	A	C	T	G	A	C	C	G	A
2	MuVi/Shannxi.CHN/20.12	A	A	C	T	G	A	C	C	G	A
2	MuVi/Liaoning.CHN/48.10/2	A	A	C	T	G	A	C	C	G	A
2	MuVi/Shandong.CHN/51.15/1	A	A	C	T	G	A	C	C	G	A
	MuVi/Hunan.CHN/32.11	A	A	C	T	G	A	C	C	G	G
	MuVi/Hunan.CHN/30.11/2	A	A	C	T	G	A	C	C	G	A
	MuVi/Heilongjiang.CHN/14.13/1	A	A	C	T	G	A	C	C	G	A
	MuVi/Jiangsu.CHN/7.12	A	A	C	T	G	A	C	C	G	A
	MuVi/Liaoning.CHN/9.10/1	A	A	C	T	G	A	C	C	G	A
	MuVi/Hunan.CHN/30.11/3	A	A	C	T	G	A	C	C	G	A
	MuVi/Jiangsu.CHN/12.13/2	A	A	C	T	G	A	C	C	G	A
	MuVi/Shannxi.CHN/20.15/1	A	A	C	T	G	A	C	C	G	A
	MuVi/Liaoning.CHN/13.12	A	A	C	T	G	A	C	C	G	A
	MuVi/Beijing.CHN/21.11	A	A	C	T	G	A	C	C	A	A
	MuVi/Liaoning.CHN/11.12	A	A	C	T	G	A	C	C	G	A
	MuVi/Jiangsu.CHN/4.13/5	A	A	C	T	G	A	C	C	G	A
	MuVi/Heilongjiang.CHN/26.12/1	A	A	C	T	G	A	C	C	G	A
	MuVi/Beijing.CHN/17.07/2	A	A	C	T	G	A	C	C	G	A
	MuVi/Sichuan.CHN/23.12/2	A	A	C	T	G	A	C	C	G	A
	MuVi/Hubei.CHN/44.12/1	A	A	C	T	G	A	C	C	G	A
	MuVi/Jiangsu.CHN/2.13/4	A	A	C	T	G	A	C	C	G	A
	MuVi/Liaoning.CHN/7.12	A	A	C	T	G	A	C	C	G	A
	MuVi/Heilongjiang.CHN/22.12	A	A	C	T	G	A	C	C	G	A
	MuVi/Heilongjiang.CHN/15.13	A	A	C	T	G	A	C	C	G	A
	MuVi/Liaoning.CHN/5.09/1	A	A	C	T	G	A	C	C	G	A
	MuVi/Liaoning.CHN/1.08/2	A	A	C	T	G	A	C	C	G	A
	MuVi/Liaoning.CHN/10.09	A	A	C	T	G	A	C	C	G	A
	MuVi/Liaoning.CHN/1.08/1	A	A	C	T	A	A	C	C	G	A
	MuVi/Liaoning.CHN/48.10/1	A	A	C	T	G	A	C	C	G	A
	MuVi/Beijing.CHN/10.07	G	A	A	T	G	A	C	C	G	A
	MuVi/Hunan.CHN/30.11/1	A	A	C	T	G	A	C	C	G	A
	MuVi/Jiangsu.CHN/7.13/1	A	A	C	T	G	A	C	C	G	A
	MuVi/Liaoning.CHN/5.09/2	A	A	C	T	G	A	C	C	G	A
	MuVi/Zhejiang.CHN/11.06/1	A	A	C	T	G	G	C	C	G	A

1: Lineage based on Figure 1
2: SNPs are noted in green.
3: SNP position corresponding to a lineage-defining node in Figure 1 [illegible]

Supplementary Table S3: Listing of SNPs featured in the 70 F sequences of I

Lineage [1]	Strain name	1461	1462	1470	1476	1479	1482	1483	1485	1492	1494
1	MuVi/Liaoning.CHN/50.11	T	A	C	T	G	C	A	T	A	T
1	MuVi/Liaoning.CHN/48.11	T	A	C	T	G	C	A	T	A	T
1	MuVi/Heilongjiang.CHN/23.13/1	T	A	C	T	G	C	A	T	A	T
1	MuVi/Shandong.CHN/43.07	T	A	C	T	G	C	A	T	A	T
1	MuVi/Shandong.CHN/10.08	T	A	C	T	G	C	A	T	A	T
1	MuVi/Heilongjiang.CHN/26.12/2	T	A	C	T	G	C	A	T	A	T
1	MuVi/Heilongjiang.CHN/24.14	T	A	C	T	G	C	A	T	A	T
1	MuVi/Sichuan.CHN/24.15	T	A	C	T	G	C	A	T	A	T
1	MuVi/Shandong.CHN/11.07	T	A	C	T	G	C	A	C	A	T
1	MuVi/Beijing.CHN/15.06	T	A	C	T	G	C	A	C	A	T
1	MuVi/Gansu.CHN/0.02	T	A	C	T	G	C	A	C	A	T
1	MuVi/Jiangsu.CHN/3.13/2	T	A	C	T	G	T	A	C	A	T
1	MuVi/Beijing.CHN/25.06	T	A	C	T	A	C	A	C	A	T
1	MuVi/Shanghai.CHN/0.01	T	A	C	T	G	C	A	C	A	T
1	MuVi/Shandong.CHN/3.05	T	A	C	T	G	C	A	C	A	T
1	MuVi/Zhejiang.CHN/26.05	T	A	C	T	G	C	A	C	A	T
1	MuVi/Sichuan.CHN/23.12/1	T	A	C	T	G	C	A	C	A	T
1	MuVi/Neimeng.CHN/18.11	T	A	C	T	G	C	A	C	A	T
1	MuVi/Yunnan.CHN/47.10/2	C	A	C	T	G	C	A	C	A	T
1	MuVi/Jilin.CHN/15.08/3	T	A	C	T	G	C	A	C	A	T
1	MuVi/Jilin.CHN/15.08/1	T	A	C	T	G	C	A	C	A	T
1	MuVi/Jilin.CHN/15.08/5	T	A	C	T	G	C	A	C	A	T
4	MuVi/Shannxi.CHN/26.09/1	T	A	C	T	G	C	A	C	A	T
4	MuVi/Jiangsu.CHN/15.12	T	A	C	T	G	C	A	C	G	T
3	MuVi/Shandong.CHN/4.05	T	A	C	T	G	C	A	C	A	T
3	MuVi/Shannxi.CHN/9.09/2	T	A	C	T	G	C	A	C	A	T
3	MuVi/Shanxi.CHN/52.10/3	T	A	T	T	G	C	A	C	A	T
3	MuVi/Jiangsu.CHN/3.13/1	T	A	G	T	G	C	A	C	A	T
3	MuVi/Henan.CHN/48.06	T	A	C	T	G	C	A	C	A	T
3	MuVi/Jiangsu.CHN/9.12	T	A	C	T	G	C	A	C	A	T
3	MuVi/Jiangsu.CHN/4.13/3	T	A	C	C	G	C	A	C	A	T
2	MuVi/Shanxi.CHN/52.10/2	T	A	C	T	G	C	A	C	A	T
2	MuVi/Shannxi.CHN/26.09/4	T	A	C	T	G	C	A	C	A	T
2	MuVi/Shanxi.CHN/52.10/1	C	A	C	T	G	C	A	C	A	T
2	MuVi/Guangdong.CHN/21.09/1	T	A	C	T	A	C	A	C	C	T
2	MuVi/Heilongjiang.CHN/49.15	T	A	C	T	G	C	A	C	A	T
2	MuVi/Anhui.CHN/10.11/1	T	A	C	T	G	C	A	C	A	T
2	MuVi/Shannxi.CHN/20.12	T	A	C	T	G	C	A	C	A	T
2	MuVi/Liaoning.CHN/48.10/2	T	A	C	T	G	C	A	C	C	T
2	MuVi/Shandong.CHN/51.15/1	T	A	C	T	G	C	A	C	A	T
	MuVi/Hunan.CHN/32.11	T	A	C	T	G	C	A	C	A	C
	MuVi/Hunan.CHN/30.11/2	T	A	C	T	G	C	A	C	A	C
	MuVi/Heilongjiang.CHN/14.13/1	T	A	C	T	G	C	A	C	A	C
	MuVi/Jiangsu.CHN/7.12	T	A	C	T	G	C	A	C	A	C
	MuVi/Liaoning.CHN/9.10/1	T	A	C	T	G	C	A	C	A	C
	MuVi/Hunan.CHN/30.11/3	T	A	C	T	G	C	A	C	A	C
	MuVi/Jiangsu.CHN/12.13/2	T	A	C	T	G	C	A	C	A	C
	MuVi/Shannxi.CHN/20.15/1	T	A	C	T	G	C	G	C	A	C
	MuVi/Liaoning.CHN/13.12	T	A	C	T	G	C	A	C	A	C
	MuVi/Beijing.CHN/21.11	T	A	C	T	G	C	A	C	A	C
	MuVi/Liaoning.CHN/11.12	T	A	C	T	G	C	A	C	A	C
	MuVi/Jiangsu.CHN/4.13/5	T	A	C	T	G	C	A	C	A	C
	MuVi/Heilongjiang.CHN/26.12/1	T	A	C	T	G	C	A	C	A	C
	MuVi/Beijing.CHN/17.07/2	T	A	C	T	G	C	A	C	A	C
	MuVi/Sichuan.CHN/23.12/2	T	C	C	T	G	C	A	C	A	C
	MuVi/Hubei.CHN/44.12/1	T	C	C	T	G	C	A	C	A	C
	MuVi/Jiangsu.CHN/2.13/4	T	C	C	T	G	C	A	C	A	C
	MuVi/Liaoning.CHN/7.12	T	C	C	T	G	C	A	C	A	C
	MuVi/Heilongjiang.CHN/22.12	T	C	C	T	G	C	A	C	A	C
	MuVi/Heilongjiang.CHN/15.13	T	C	C	T	G	C	A	C	A	C
	MuVi/Liaoning.CHN/5.09/1	T	A	C	T	G	C	A	A	A	C
	MuVi/Liaoning.CHN/1.08/2	T	A	C	T	G	C	A	A	A	C
	MuVi/Liaoning.CHN/10.09	T	A	C	T	G	C	A	A	A	C
	MuVi/Liaoning.CHN/1.08/1	T	A	C	T	G	C	A	C	A	C
	MuVi/Liaoning.CHN/48.10/1	T	A	C	T	G	C	A	C	A	C
	MuVi/Beijing.CHN/10.07	T	A	C	T	G	C	A	C	A	T
	MuVi/Hunan.CHN/30.11/1	T	A	C	T	G	C	A	C	A	T
	MuVi/Jiangsu.CHN/7.13/1	T	A	C	T	G	C	A	C	A	T
	MuVi/Liaoning.CHN/5.09/2	T	A	C	T	G	C	A	C	A	T
	MuVi/Zhejiang.CHN/11.06/1	T	A	C	T	G	C	A	C	A	T

1: Lineage based on Figure 1
2: SNPs are noted in green.
3: SNP position corresponding to a lineage-defining node in Figure 1

Supplementary Table S3: Listing of SNPs featured in the 70 F sequences of [illegible]

Lineage [1]	Strain name	1497	1500	1503	1515	1524	1527	1536	1549	1551	1557
1	MuVi/Liaoning.CHN/50.11	C	T	G	C	T	C	T	A	A	C
1	MuVi/Liaoning.CHN/48.11	C	T	G	C	T	C	T	A	A	C
1	MuVi/Heilongjiang.CHN/23.13/1	C	T	G	C	T	C	T	A	A	C
1	MuVi/Shandong.CHN/43.07	C	A	G	C	T	C	T	A	A	C
1	MuVi/Shandong.CHN/10.08	C	A	G	C	T	C	T	A	A	C
1	MuVi/Heilongjiang.CHN/26.12/2	C	T	G	C	T	C	T	A	A	C
1	MuVi/Heilongjiang.CHN/24.14	C	T	G	C	T	C	T	A	A	C
1	MuVi/Sichuan.CHN/24.15	C	T	G	C	T	C	T	A	A	C
1	MuVi/Shandong.CHN/11.07	C	T	G	C	T	C	T	A	A	C
1	MuVi/Beijing.CHN/15.06	C	T	G	C	T	C	T	A	A	C
1	MuVi/Gansu.CHN/0.02	C	T	G	C	T	C	T	A	A	C
1	MuVi/Jiangsu.CHN/3.13/2	C	T	G	C	T	C	T	A	A	C
1	MuVi/Beijing.CHN/25.06	C	T	G	C	T	C	G	A	A	C
1	MuVi/Shanghai.CHN/0.01	C	T	G	C	T	C	T	A	A	C
1	MuVi/Shandong.CHN/3.05	C	T	G	C	T	C	T	A	A	C
1	MuVi/Zhejiang.CHN/26.05	C	T	G	C	T	C	T	A	A	C
1	MuVi/Sichuan.CHN/23.12/1	C	T	G	C	T	C	T	A	A	C
1	MuVi/Neimeng.CHN/18.11	C	T	G	C	T	C	T	A	A	C
1	MuVi/Yunnan.CHN/47.10/2	C	T	G	C	T	C	T	A	A	C
1	MuVi/Jilin.CHN/15.08/3	C	T	G	C	T	C	T	A	A	C
1	MuVi/Jilin.CHN/15.08/1	C	T	G	C	T	C	T	A	A	C
1	MuVi/Jilin.CHN/15.08/5	C	T	G	C	T	C	T	A	A	C
4	MuVi/Shannxi.CHN/26.09/1	C	T	T	C	T	C	T	A	A	C
4	MuVi/Jiangsu.CHN/15.12	C	T	T	C	T	C	T	A	A	C
3	MuVi/Shandong.CHN/4.05	C	T	G	C	C	C	T	A	A	C
3	MuVi/Shannxi.CHN/9.09/2	C	T	G	C	T	C	T	A	A	C
3	MuVi/Shanxi.CHN/52.10/3	C	T	T	C	T	C	T	A	A	C
3	MuVi/Jiangsu.CHN/3.13/1	C	T	G	C	T	C	T	A	A	C
3	MuVi/Henan.CHN/48.06	C	T	G	C	T	C	T	A	A	C
3	MuVi/Jiangsu.CHN/9.12	C	T	G	C	T	C	T	A	A	C
3	MuVi/Jiangsu.CHN/4.13/3	C	T	G	C	T	C	T	A	A	C
2	MuVi/Shanxi.CHN/52.10/2	C	T	G	C	T	C	T	A	A	C
2	MuVi/Shannxi.CHN/26.09/4	C	T	G	T	T	C	T	A	A	C
2	MuVi/Shanxi.CHN/52.10/1	C	T	G	C	T	C	T	A	A	C
2	MuVi/Guangdong.CHN/21.09/1	C	T	G	C	T	C	T	A	A	C
2	MuVi/Heilongjiang.CHN/49.15	T	T	G	C	T	T	T	A	A	C
2	MuVi/Anhui.CHN/10.11/1	C	T	G	C	T	C	T	A	A	C
2	MuVi/Shannxi.CHN/20.12	C	T	G	C	T	C	T	A	A	C
2	MuVi/Liaoning.CHN/48.10/2	C	T	G	C	T	C	T	A	A	C
2	MuVi/Shandong.CHN/51.15/1	C	T	G	C	T	C	T	A	A	C
	MuVi/Hunan.CHN/32.11	C	T	G	C	T	C	T	A	A	C
	MuVi/Hunan.CHN/30.11/2	C	T	G	C	T	C	T	A	A	C
	MuVi/Heilongjiang.CHN/14.13/1	C	T	G	C	T	C	T	A	A	C
	MuVi/Jiangsu.CHN/7.12	C	T	G	C	T	C	T	A	A	C
	MuVi/Liaoning.CHN/9.10/1	C	T	G	C	T	C	T	A	A	C
	MuVi/Hunan.CHN/30.11/3	C	T	G	C	T	C	T	A	A	C
	MuVi/Jiangsu.CHN/12.13/2	C	T	G	C	T	T	T	A	A	C
	MuVi/Shannxi.CHN/20.15/1	C	T	G	C	T	T	T	A	A	C
	MuVi/Liaoning.CHN/13.12	C	T	G	C	T	C	T	A	A	C
	MuVi/Beijing.CHN/21.11	C	T	G	C	T	C	T	A	A	C
	MuVi/Liaoning.CHN/11.12	C	T	G	C	T	C	T	A	A	C
	MuVi/Jiangsu.CHN/4.13/5	C	T	G	C	T	C	T	A	A	C
	MuVi/Heilongjiang.CHN/26.12/1	C	T	G	C	T	C	C	A	A	C
	MuVi/Beijing.CHN/17.07/2	C	T	G	C	T	C	T	A	A	C
	MuVi/Sichuan.CHN/23.12/2	C	T	G	C	T	C	T	A	A	C
	MuVi/Hubei.CHN/44.12/1	C	T	G	C	T	C	T	A	A	C
	MuVi/Jiangsu.CHN/2.13/4	C	T	G	C	T	C	T	A	A	C
	MuVi/Liaoning.CHN/7.12	C	T	G	C	T	C	T	A	A	C
	MuVi/Heilongjiang.CHN/22.12	C	T	G	C	T	C	T	A	A	C
	MuVi/Heilongjiang.CHN/15.13	C	T	G	C	T	C	T	A	A	C
	MuVi/Liaoning.CHN/5.09/1	C	T	G	C	T	C	T	A	A	C
	MuVi/Liaoning.CHN/1.08/2	C	T	G	C	T	C	T	A	A	C
	MuVi/Liaoning.CHN/10.09	C	T	G	C	T	C	T	A	A	C
	MuVi/Liaoning.CHN/1.08/1	C	T	G	C	T	C	T	A	A	C
	MuVi/Liaoning.CHN/48.10/1	C	T	G	C	T	C	T	A	A	C
	MuVi/Beijing.CHN/10.07	C	T	G	C	T	C	T	A	A	C
	MuVi/Hunan.CHN/30.11/1	C	T	G	C	T	C	T	A	G	C
	MuVi/Jiangsu.CHN/7.13/1	C	T	G	C	T	C	T	A	G	C
	MuVi/Liaoning.CHN/5.09/2	C	T	G	C	T	C	T	C	A	T
	MuVi/Zhejiang.CHN/11.06/1	C	T	G	C	T	C	T	A	A	C

1: Lineage based on Figure 1
2: SNPs are noted in green.
3: SNP position corresponding to a lineage-defining node in Figure 1 [illegible]

Supplementary Table S3: Listing of SNPs featured in the 70 F sequences of [illegible]

Lineage [1]	Strain name	1562	1566	1572	1578	1581	1582	1584	1590	1594	1603
1	MuVi/Liaoning.CHN/50.11	A	A	T	C	A	A	A	T	G	C
1	MuVi/Liaoning.CHN/48.11	A	A	T	C	A	A	A	T	G	C
1	MuVi/Heilongjiang.CHN/23.13/1	A	A	T	C	A	A	A	T	G	C
1	MuVi/Shandong.CHN/43.07	A	A	T	C	A	A	A	T	G	C
1	MuVi/Shandong.CHN/10.08	A	A	T	C	A	A	A	T	G	C
1	MuVi/Heilongjiang.CHN/26.12/2	A	A	T	C	A	**C**	A	T	G	C
1	MuVi/Heilongjiang.CHN/24.14	A	A	T	C	A	A	A	T	G	C
1	MuVi/Sichuan.CHN/24.15	A	A	T	C	A	A	A	T	G	C
1	MuVi/Shandong.CHN/11.07	**G**	A	T	C	A	A	A	**C**	G	C
1	MuVi/Beijing.CHN/15.06	**G**	A	T	C	A	A	A	T	G	C
1	MuVi/Gansu.CHN/0.02	A	A	T	C	A	A	A	T	G	C
1	MuVi/Jiangsu.CHN/3.13/2	A	A	T	C	A	A	A	T	G	C
1	MuVi/Beijing.CHN/25.06	A	A	T	C	A	A	A	T	G	C
1	MuVi/Shanghai.CHN/0.01	A	A	T	C	A	A	A	T	G	C
1	MuVi/Shandong.CHN/3.05	A	A	T	C	A	A	A	T	G	C
1	MuVi/Zhejiang.CHN/26.05	A	A	T	C	A	A	A	T	G	C
1	MuVi/Sichuan.CHN/23.12/1	A	A	T	C	A	A	A	T	G	C
1	MuVi/Neimeng.CHN/18.11	A	**G**	T	C	A	A	A	T	G	C
1	MuVi/Yunnan.CHN/47.10/2	A	A	T	C	A	A	A	T	G	C
1	MuVi/Jilin.CHN/15.08/3	A	A	T	C	A	A	A	T	G	C
1	MuVi/Jilin.CHN/15.08/1	A	A	T	C	A	A	A	T	G	C
1	MuVi/Jilin.CHN/15.08/5	A	A	T	C	A	A	A	T	G	C
4	MuVi/Shannxi.CHN/26.09/1	A	A	T	C	A	A	A	T	G	C
4	MuVi/Jiangsu.CHN/15.12	A	A	T	C	A	A	A	T	G	C
3	MuVi/Shandong.CHN/4.05	A	A	T	C	A	A	A	T	G	C
3	MuVi/Shannxi.CHN/9.09/2	A	A	T	C	A	A	A	T	G	C
3	MuVi/Shanxi.CHN/52.10/3	A	A	**C**	C	A	A	A	T	G	**T**
3	MuVi/Jiangsu.CHN/3.13/1	A	A	**C**	C	A	A	A	T	G	C
3	MuVi/Henan.CHN/48.06	A	A	T	C	A	A	A	T	G	C
3	MuVi/Jiangsu.CHN/9.12	A	A	T	C	A	A	A	T	G	C
3	MuVi/Jiangsu.CHN/4.13/3	A	A	T	C	A	A	A	T	G	C
2	MuVi/Shanxi.CHN/52.10/2	A	A	**C**	C	A	A	**T**	T	G	C
2	MuVi/Shannxi.CHN/26.09/4	A	A	**C**	C	A	A	**T**	T	G	C
2	MuVi/Shanxi.CHN/52.10/1	A	A	**C**	C	A	A	**T**	T	G	C
2	MuVi/Guangdong.CHN/21.09/1	A	A	**C**	C	A	A	**T**	T	G	C
2	MuVi/Heilongjiang.CHN/49.15	A	A	**C**	C	A	A	**T**	T	G	C
2	MuVi/Anhui.CHN/10.11/1	A	A	**C**	C	A	A	**T**	T	G	C
2	MuVi/Shannxi.CHN/20.12	A	A	**C**	C	A	A	**T**	T	G	C
2	MuVi/Liaoning.CHN/48.10/2	A	A	**C**	C	A	A	**T**	T	G	C
2	MuVi/Shandong.CHN/51.15/1	A	A	**C**	C	A	A	**T**	T	G	C
	MuVi/Hunan.CHN/32.11	A	A	T	C	A	A	A	T	G	C
	MuVi/Hunan.CHN/30.11/2	A	A	T	C	A	A	A	T	G	C
	MuVi/Heilongjiang.CHN/14.13/1	A	A	T	C	A	A	A	T	G	C
	MuVi/Jiangsu.CHN/7.12	A	A	T	C	A	A	A	T	G	C
	MuVi/Liaoning.CHN/9.10/1	A	A	T	C	A	A	A	T	G	C
	MuVi/Hunan.CHN/30.11/3	A	A	T	C	A	A	A	T	G	C
	MuVi/Jiangsu.CHN/12.13/2	A	A	T	C	A	A	A	T	G	C
	MuVi/Shannxi.CHN/20.15/1	A	A	T	C	A	A	A	T	G	C
	MuVi/Liaoning.CHN/13.12	A	A	T	C	A	A	A	T	G	C
	MuVi/Beijing.CHN/21.11	A	A	T	C	A	A	A	T	G	C
	MuVi/Liaoning.CHN/11.12	A	A	T	C	A	A	A	T	G	C
	MuVi/Jiangsu.CHN/4.13/5	A	A	T	C	A	A	A	T	G	C
	MuVi/Heilongjiang.CHN/26.12/1	A	A	T	C	A	A	A	T	G	C
	MuVi/Beijing.CHN/17.07/2	A	A	T	C	A	A	A	T	G	C
	MuVi/Sichuan.CHN/23.12/2	A	A	T	C	A	A	A	T	G	C
	MuVi/Hubei.CHN/44.12/1	A	A	T	C	A	A	A	T	G	C
	MuVi/Jiangsu.CHN/2.13/4	A	A	T	C	A	A	A	T	G	C
	MuVi/Liaoning.CHN/7.12	A	A	T	C	A	A	A	T	G	C
	MuVi/Heilongjiang.CHN/22.12	A	A	T	C	**G**	A	A	T	G	C
	MuVi/Heilongjiang.CHN/15.13	A	A	T	C	**G**	A	A	T	G	C
	MuVi/Liaoning.CHN/5.09/1	A	A	T	C	A	A	A	T	G	C
	MuVi/Liaoning.CHN/1.08/2	A	A	T	C	A	A	A	T	G	C
	MuVi/Liaoning.CHN/10.09	A	A	T	C	A	A	A	T	G	C
	MuVi/Liaoning.CHN/1.08/1	A	A	T	C	A	A	A	T	G	C
	MuVi/Liaoning.CHN/48.10/1	A	A	T	C	A	A	A	T	G	C
	MuVi/Beijing.CHN/10.07	A	A	T	**T**	A	A	A	T	G	C
	MuVi/Hunan.CHN/30.11/1	A	A	T	C	A	A	A	T	**A**	C
	MuVi/Jiangsu.CHN/7.13/1	A	A	T	C	A	A	A	T	G	C
	MuVi/Liaoning.CHN/5.09/2	A	A	T	C	A	A	A	T	G	C
	MuVi/Zhejiang.CHN/11.06/1	**G**	A	T	C	A	A	A	T	G	C

1: Lineage based on Figure 1
2: SNPs are noted in green.
3: SNP position corresponding to a lineage-defining node in Figure 1 [illegible]

Supplementary Table S3: Listing of SNPs featured in the 70 F sequences of I

Lineage [1]	Strain name	1605	1608	1614
1	MuVi/Liaoning.CHN/50.11	C	C	C
1	MuVi/Liaoning.CHN/48.11	C	C	C
1	MuVi/Heilongjiang.CHN/23.13/1	C	C	C
1	MuVi/Shandong.CHN/43.07	C	C	C
1	MuVi/Shandong.CHN/10.08	C	C	C
1	MuVi/Heilongjiang.CHN/26.12/2	C	C	C
1	MuVi/Heilongjiang.CHN/24.14	C	C	C
1	MuVi/Sichuan.CHN/24.15	C	C	C
1	MuVi/Shandong.CHN/11.07	C	C	C
1	MuVi/Beijing.CHN/15.06	C	C	C
1	MuVi/Gansu.CHN/0.02	C	C	C
1	MuVi/Jiangsu.CHN/3.13/2	C	C	C
1	MuVi/Beijing.CHN/25.06	C	C	C
1	MuVi/Shanghai.CHN/0.01	C	C	C
1	MuVi/Shandong.CHN/3.05	C	C	C
1	MuVi/Zhejiang.CHN/26.05	C	C	C
1	MuVi/Sichuan.CHN/23.12/1	C	C	C
1	MuVi/Neimeng.CHN/18.11	C	C	C
1	MuVi/Yunnan.CHN/47.10/2	C	C	C
1	MuVi/Jilin.CHN/15.08/3	C	C	C
1	MuVi/Jilin.CHN/15.08/1	C	C	C
1	MuVi/Jilin.CHN/15.08/5	C	C	C
4	MuVi/Shannxi.CHN/26.09/1	C	C	C
4	MuVi/Jiangsu.CHN/15.12	C	C	C
3	MuVi/Shandong.CHN/4.05	C	C	C
3	MuVi/Shannxi.CHN/9.09/2	C	C	C
3	MuVi/Shanxi.CHN/52.10/3	A	C	C
3	MuVi/Jiangsu.CHN/3.13/1	A	C	C
3	MuVi/Henan.CHN/48.06	T	C	C
3	MuVi/Jiangsu.CHN/9.12	T	C	C
3	MuVi/Jiangsu.CHN/4.13/3	T	C	C
2	MuVi/Shanxi.CHN/52.10/2	C	C	C
2	MuVi/Shannxi.CHN/26.09/4	C	C	C
2	MuVi/Shanxi.CHN/52.10/1	C	C	C
2	MuVi/Guangdong.CHN/21.09/1	C	C	C
2	MuVi/Heilongjiang.CHN/49.15	C	C	C
2	MuVi/Anhui.CHN/10.11/1	C	C	C
2	MuVi/Shannxi.CHN/20.12	C	C	C
2	MuVi/Liaoning.CHN/48.10/2	C	C	C
2	MuVi/Shandong.CHN/51.15/1	C	C	C
	MuVi/Hunan.CHN/32.11	T	C	C
	MuVi/Hunan.CHN/30.11/2	C	C	C
	MuVi/Heilongjiang.CHN/14.13/1	C	C	C
	MuVi/Jiangsu.CHN/7.12	C	C	C
	MuVi/Liaoning.CHN/9.10/1	C	C	C
	MuVi/Hunan.CHN/30.11/3	C	C	C
	MuVi/Jiangsu.CHN/12.13/2	C	C	C
	MuVi/Shannxi.CHN/20.15/1	C	C	C
	MuVi/Liaoning.CHN/13.12	C	C	C
	MuVi/Beijing.CHN/21.11	C	C	C
	MuVi/Liaoning.CHN/11.12	C	C	C
	MuVi/Jiangsu.CHN/4.13/5	C	C	C
	MuVi/Heilongjiang.CHN/26.12/1	C	C	C
	MuVi/Beijing.CHN/17.07/2	C	C	C
	MuVi/Sichuan.CHN/23.12/2	A	C	C
	MuVi/Hubei.CHN/44.12/1	C	C	C
	MuVi/Jiangsu.CHN/2.13/4	C	C	T
	MuVi/Liaoning.CHN/7.12	C	C	C
	MuVi/Heilongjiang.CHN/22.12	C	C	C
	MuVi/Heilongjiang.CHN/15.13	C	C	C
	MuVi/Liaoning.CHN/5.09/1	C	C	C
	MuVi/Liaoning.CHN/1.08/2	C	C	C
	MuVi/Liaoning.CHN/10.09	C	C	C
	MuVi/Liaoning.CHN/1.08/1	C	C	C
	MuVi/Liaoning.CHN/48.10/1	C	C	C
	MuVi/Beijing.CHN/10.07	C	C	C
	MuVi/Hunan.CHN/30.11/1	C	C	C
	MuVi/Jiangsu.CHN/7.13/1	C	C	C
	MuVi/Liaoning.CHN/5.09/2	C	A	C
	MuVi/Zhejiang.CHN/11.06/1	C	C	C

1: Lineage based on Figure 1
2: SNPs are noted in green.
3: SNP position corresponding to a lineage-defining node in Figure 1

Supplementary table S4: List of sequences of MuVs of non-F genotype analyzed in this study

Gene	Reference stains	Year	Genotype	Genbank ID
SH	MuVi/Boston.USA/0.45[A]	1945	A	GU980052
	MuVi/Vector.RUS/0.53[N](VAC)	1953	N	AY508995
	MuVi/Pennsylvannia.USA/13.63[A](VAC)	1963	A	AF338106
	MuVi/Urabe.JPN/0.67[B]	1967	B	AB000388
	MuVi/SBL-1.SWE/0.69[A]	1969	A	D00663
	MuVi/BELFAST.GBR/0.75[C]	1975	C	X63709
	MuVi/Ge9.DEU/0.77[D]	1977	D	JQ945275
	MuVi/DK81-01.DNK/0.81[K]	1981	K	AF365891
	MuVi/Stockholm.SWE/26.83[K]	1983	K	JQ945270
	MuVi/Matsuyama.JPN/0.84[B]	1984	B	D90233
	MuVi/Stockholm.SWE/46.84[C]	1984	C	JQ945268
	MuVi/Ed2.GBR/0.88[C]	1988	C	X63711
	MuVi/Bedford.GBR/0.89[H]	1989	H	JQ945273
	MuVi/Tokyo.JPN/0.93	1993	outlier	AB003415
	MuVi/Akita.JPN/42.93[I]	1993	I	JQ945274
	MuVi/MP94H.JPN/0.94[J]	1994	J	AB003417
HN	MuVi/Boston.USA/0.45[A]	1945	A	GU980052
	MuVi/Kilham.USA/0.50[A]	1950	A	EF493026
	MuVi/L3_vector.RUS/0.53[N]	1953	N	AY508995
	MuVi/Pennsylvannia.USA/13.63[A](VAC)	1963	A	FN431985
	MuVi/Urabe.JPN/0.67[B]	1967	B	AB000388
	MuVi/SBL-1.SWE/0.69[A]	1969	A	M55065
	MuVi/V6.SWE/0.71[K]	1971	K	KF864468
	MuVi/Ge9.DEU/0.77[D]	1977	D	JQ946039
	MuVi/V27.SWE/0.83[D]	1983	D	KF864462
	MuVi/Stockholm.SWE/26.83[K]	1983	K	JQ946045
	MuVi/Stockholm.SWE/46.84[C]	1984	C	JQ999999
	MuVi/1961.USA/0.88[H]	1988	H	AF467767
	MuVi/Bedford.GBR/0.89[H]	1989	H	JQ946035
	MuVi/Akita.JPN/42.93[I]	1993	I	JQ946037
	MuVi/Manchester.GBR/31.95[H]	1995	H	KF864469
	MuVi/Gloucester.GBR/32.96[G]	1996	G	AF280799
	MuVi/Zagreb.HRV/39.98[C]	1998	C	EU370206
	MuVi/Stamford_Hill.GBR/51.98[C]	1998	C	JQ034466
	MuVi/Dg1062.KOR/46.98[I]	1998	I	AY309060
	MuVi/Lit976.LTU/0.99[C]	1999	C	AY502059
	MuVi/Himeji.JPN/24.00[B]	2000	B	JQ946041
	MuVi/Sapporo.JPN/12.00[J]	2000	J	JQ946044
	MuVi/Fukuoka.JPN/41.00[L]	2000	L	JQ946036
F	MuVi/Boston.USA/0.45[A]	1945	A	GU980052
	MuVi/L3_vector.RUS/0.53[N]	1953	N	AY508995
	MuVi/Pennsylvannia.USA/13.63[A](VAC)	1963	A	AF201473
	MuVi/L-Zagreb.HRV/0.71[N](VAC)	1971	N	AY685920
	MuVi/1961.USA/0.88[H]	1988	H	AF467767
	MuVi/ODATE-3.JPN/00.93[I]	1993	I	AB600942
	MuVi/Gw7.CAN/00.96[B]	1996	B	FJ375178
	MuVi/Gloucester.GBR/32.96[G]	1996	G	AF280799
	MuVi/Zagreb.HRV/39.98[C]	1998	C	EU370206
	MuVi/Dg1062.KOR/46.98[I]	1998	I	AY309060

Supplementary Figure S1: Geographic distribution of the sequences analyzed in this study. The number of sequences for each gene is represented for each province and each administrative region as a bar graph, SH in blue, HN in red and F in green.

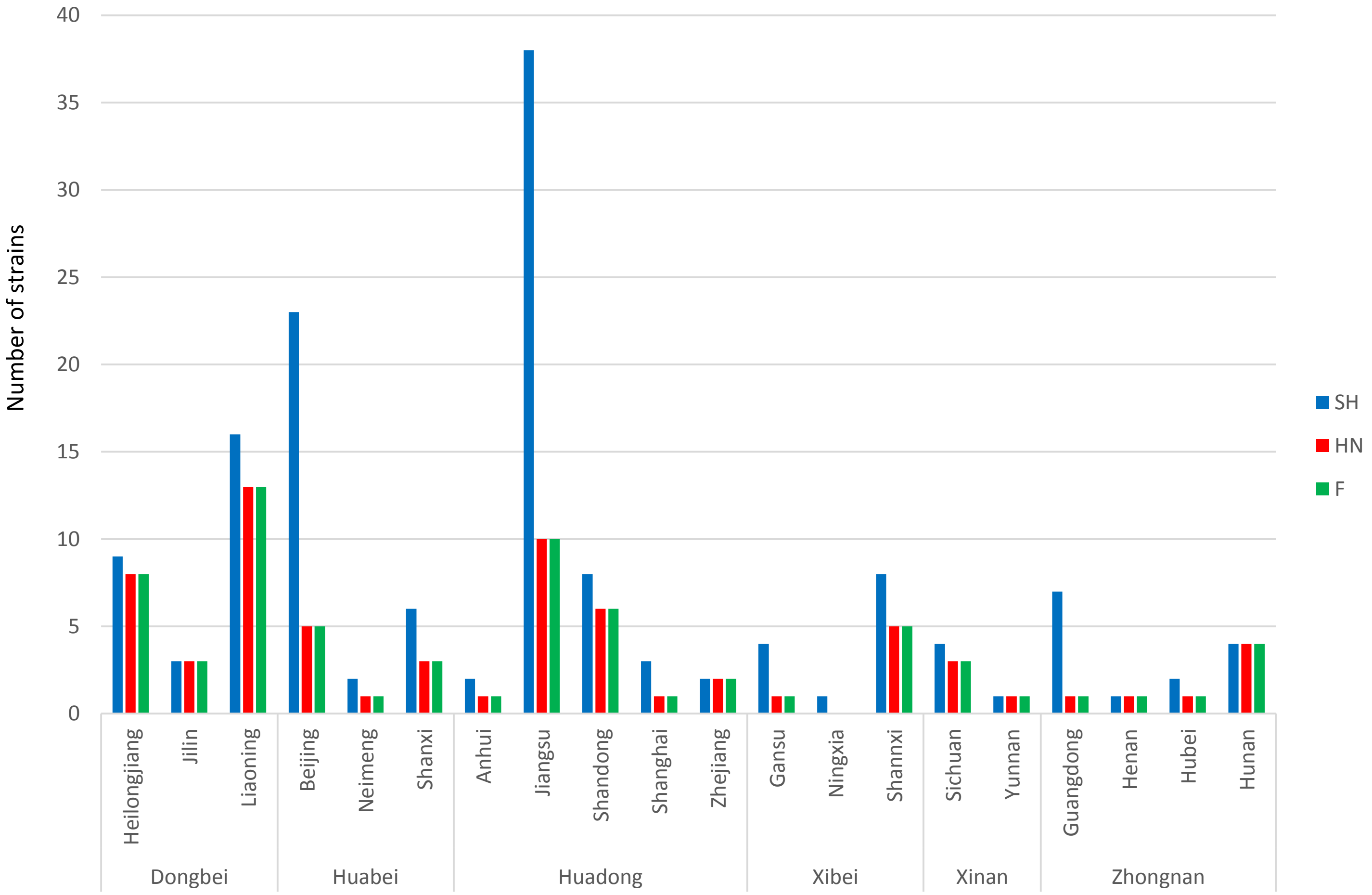

Supplementary Figure S1

Supplementary Figure S2: Maximum likelihood tree on SH, HN and F dataset. Lineages identified in Figure 1 are shown. Bootstrap values greater than 80% are indicated on the trees.

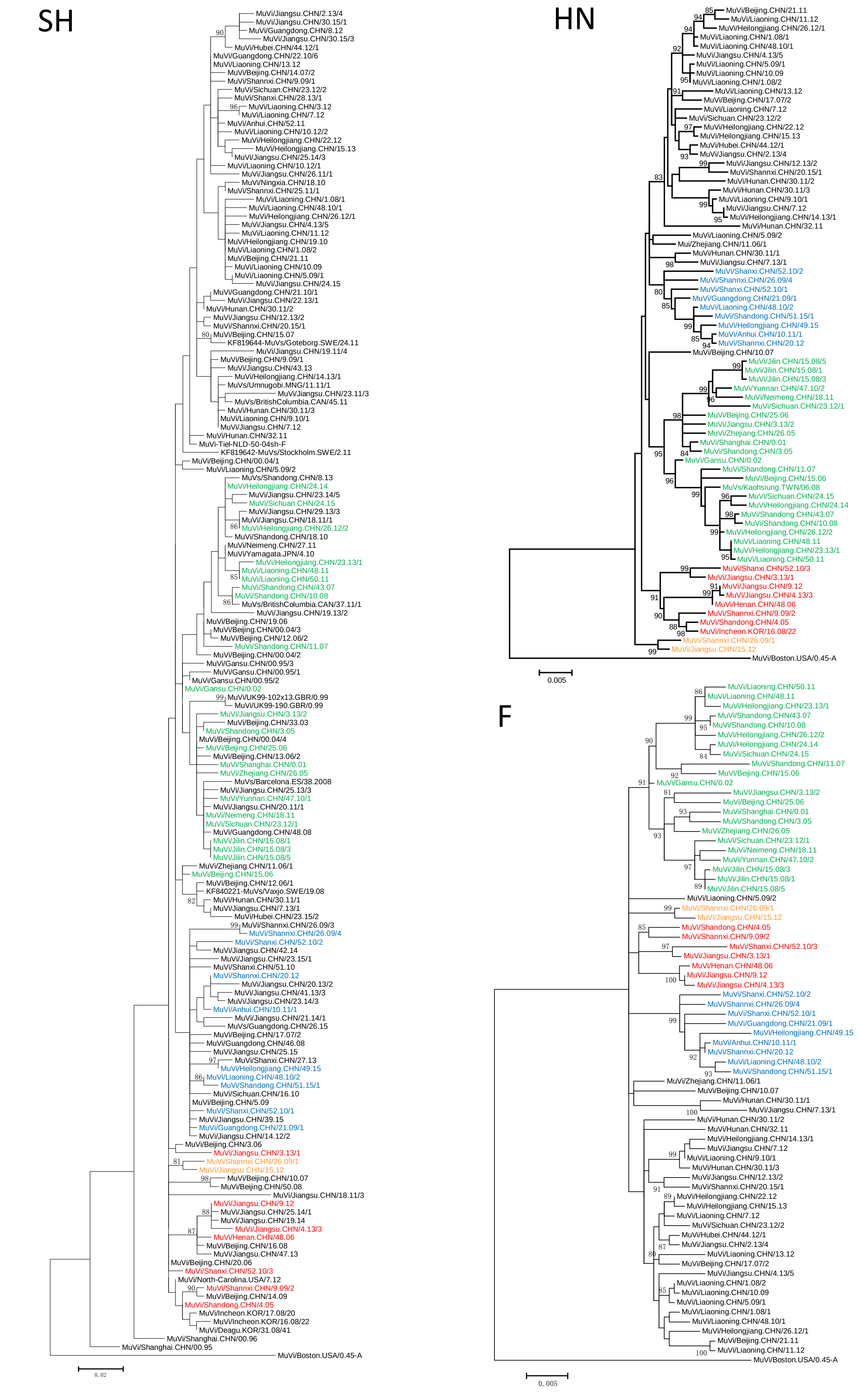

Supplementary Figure S2

Supplementary Figure S3: Maximum Clade Credibility (MCC) trees for SH, HN and F genes. 95% HPD intervals are shown with horizontal blue bars. Sequences belonging to the lineages 1 to 4 are colored according to Figure 1. TMRCA at relevant nodes are indicated.

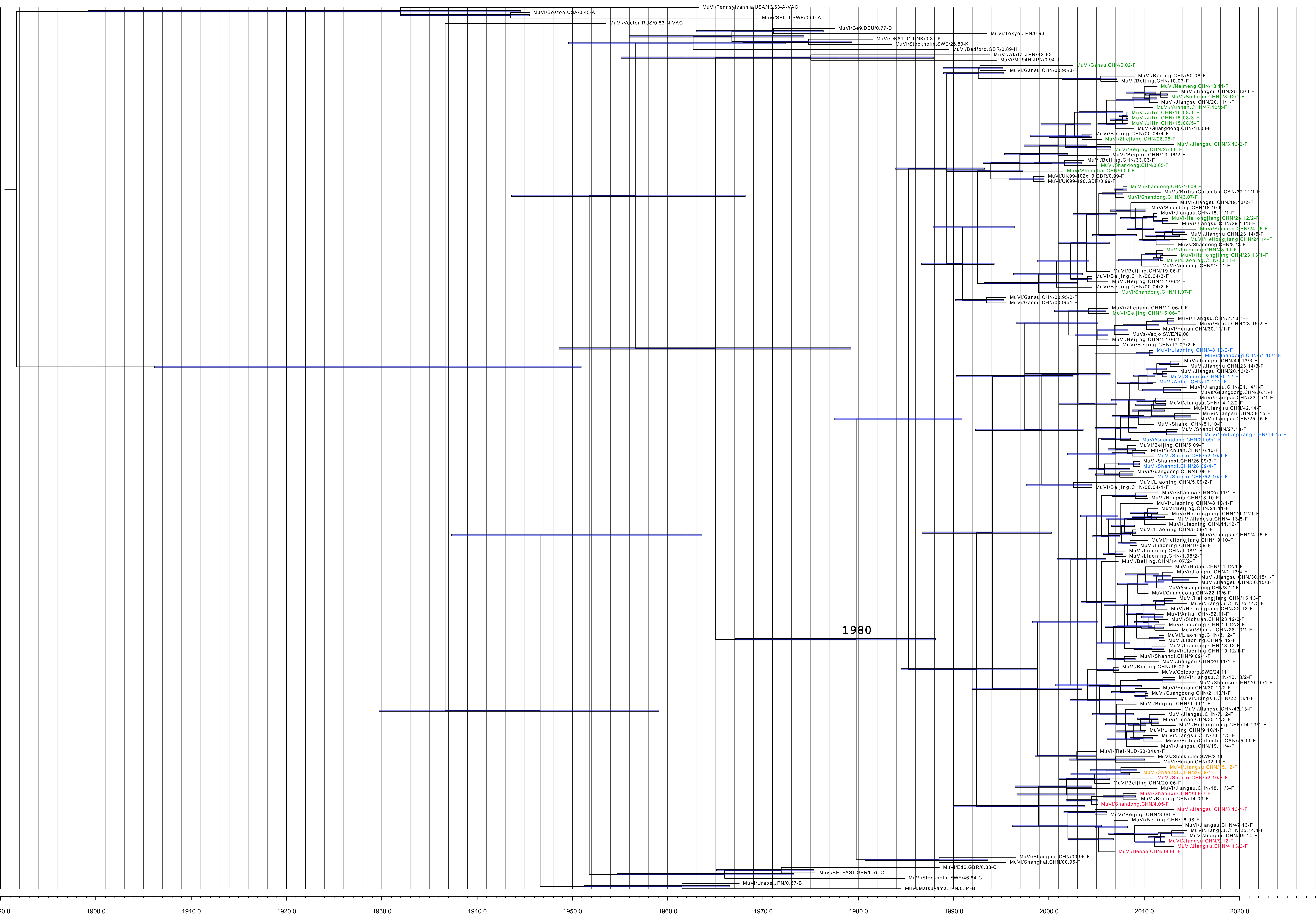

MuVi/Pennsylvannia.USA/13.63-A-VAC
MuVi/Boston.USA/0.45-A
MuVi/SBL-1.SWE/0.69-A
MuVi/Vector.RUS/0.53-N-VAC
MuVi/Ge9.DEU/0.77-D
MuVi/Tokyo.JPN/0.93
MuVi/DK81-01.DNK/0.81-K
MuVi/Stockholm.SWE/25.83-K
MuVi/Bedford.GBR/0.89-H
MuVi/Akita.JPN/42.93-I
MuVi/MP94H.JPN/0.94-J
MuVi/Shanghai.CHN/00.96-F
MuVi/Shanghai.CHN/00.95-F
MuVi/Ed2.GBR/0.88-C
MuVi/BELFAST.GBR/0.75-C
MuVi/Stockholm.SWE/46.84-C
MuVi/Urabe.JPN/0.67-B
MuVi/Matsuyama.JPN/0.84-B
1980
1900.0
1910.0
1920.0
1930.0
1940.0
1950.0
1960.0
1970.0
1980.0
1990.0
2000.0
2010.0
2020.0

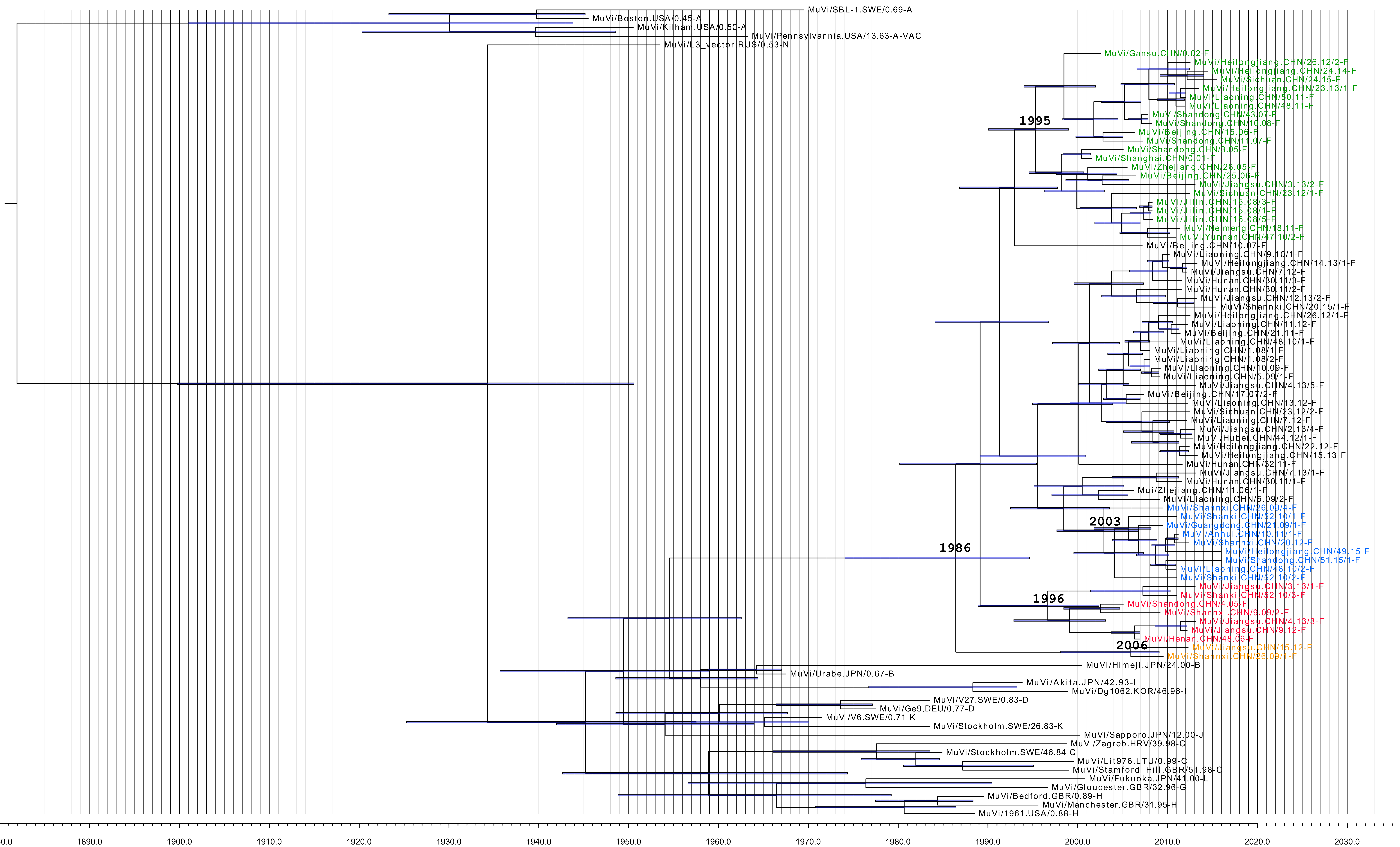

MuVi/SBL-1.SWE/0.69-A
MuVi/Boston.USA/0.45-A
MuVi/Kilham.USA/0.50-A
MuVi/Pennsylvannia.USA/13.63-A-VAC
MuVi/L3_vector.RUS/0.53-N
MuVi/Gansu.CHN/0.02-F
MuVi/Heilongjiang.CHN/26.12/2-F
MuVi/Heilongjiang.CHN/24.14-F
MuVi/Sichuan.CHN/24.15-F
MuVi/Heilongjiang.CHN/23.13/1-F
MuVi/Liaoning.CHN/50.11-F
MuVi/Liaoning.CHN/48.11-F
MuVi/Shandong.CHN/43.07-F
MuVi/Shandong.CHN/10.08-F
1995
MuVi/Beijing.CHN/15.06-F
MuVi/Shandong.CHN/11.07-F
MuVi/Shandong.CHN/3.05-F
MuVi/Shanghai.CHN/0.01-F
MuVi/Zhejiang.CHN/26.05-F
MuVi/Beijing.CHN/25.06-F
MuVi/Jiangsu.CHN/3.13/2-F
MuVi/Sichuan.CHN/23.12/1-F
MuVi/Jilin.CHN/15.08/3-F
MuVi/Jilin.CHN/15.08/1-F
MuVi/Jilin.CHN/15.08/5-F
MuVi/Neimeng.CHN/18.11-F
MuVi/Yunnan.CHN/47.10/2-F
MuVi/Beijing.CHN/10.07-F
MuVi/Liaoning.CHN/9.10/1-F
MuVi/Heilongjiang.CHN/14.13/1-F
MuVi/Jiangsu.CHN/7.12-F
MuVi/Hunan.CHN/30.11/3-F
MuVi/Hunan.CHN/30.11/2-F
MuVi/Jiangsu.CHN/12.13/2-F
MuVi/Shannxi.CHN/20.15/1-F
MuVi/Heilongjiang.CHN/26.12/1-F
MuVi/Liaoning.CHN/11.12-F
MuVi/Beijing.CHN/21.11-F
MuVi/Liaoning.CHN/48.10/1-F
MuVi/Liaoning.CHN/1.08/1-F
MuVi/Liaoning.CHN/1.08/2-F
MuVi/Liaoning.CHN/10.09-F
MuVi/Liaoning.CHN/5.09/1-F
MuVi/Jiangsu.CHN/4.13/5-F
MuVi/Beijing.CHN/17.07/2-F
MuVi/Liaoning.CHN/13.12-F
MuVi/Sichuan.CHN/23.12/2-F
MuVi/Liaoning.CHN/7.12-F
MuVi/Jiangsu.CHN/2.13/4-F
MuVi/Hubei.CHN/44.12/1-F
MuVi/Heilongjiang.CHN/22.12-F
MuVi/Heilongjiang.CHN/15.13-F
MuVi/Hunan.CHN/32.11-F
MuVi/Jiangsu.CHN/7.13/1-F
MuVi/Hunan.CHN/30.11/1-F
Mui/Zhejiang.CHN/11.06/1-F
MuVi/Liaoning.CHN/5.09/2-F
MuVi/Shannxi.CHN/26.09/4-F
MuVi/Shanxi.CHN/52.10/1-F
2003
MuVi/Guangdong.CHN/21.09/1-F
MuVi/Anhui.CHN/10.11/1-F
MuVi/Shannxi.CHN/20.12-F
MuVi/Heilongjiang.CHN/49.15-F
MuVi/Shandong.CHN/51.15/1-F
1986
MuVi/Liaoning.CHN/48.10/2-F
MuVi/Shanxi.CHN/52.10/2-F
MuVi/Jiangsu.CHN/3.13/1-F
MuVi/Shanxi.CHN/52.10/3-F
1996
MuVi/Shandong.CHN/4.05-F
MuVi/Shannxi.CHN/9.09/2-F
MuVi/Jiangsu.CHN/4.13/3-F
MuVi/Jiangsu.CHN/9.12-F
MuVi/Henan.CHN/48.06-F
2006
MuVi/Jiangsu.CHN/15.12-F
MuVi/Shannxi.CHN/26.09/1-F
MuVi/Himeji.JPN/24.00-B
MuVi/Urabe.JPN/0.67-B
MuVi/Akita.JPN/42.93-I
MuVi/Dg1062.KOR/46.98-I
MuVi/V27.SWE/0.83-D
MuVi/Ge9.DEU/0.77-D
MuVi/V6.SWE/0.71-K
MuVi/Stockholm.SWE/26.83-K
MuVi/Sapporo.JPN/12.00-J
MuVi/Zagreb.HRV/39.98-C
MuVi/Stockholm.SWE/46.84-C
MuVi/Lit976.LTU/0.99-C
MuVi/Stamford_Hill.GBR/51.98-C
MuVi/Fukuoka.JPN/41.00-L
MuVi/Gloucester.GBR/32.96-G
MuVi/Bedford.GBR/0.89-H
MuVi/Manchester.GBR/31.95-H
MuVi/1961.USA/0.88-H
1890.0
1900.0
1910.0
1920.0
1930.0
1940.0
1950.0
1960.0
1970.0
1980.0
1990.0
2000.0
2010.0
2020.0
2030.0

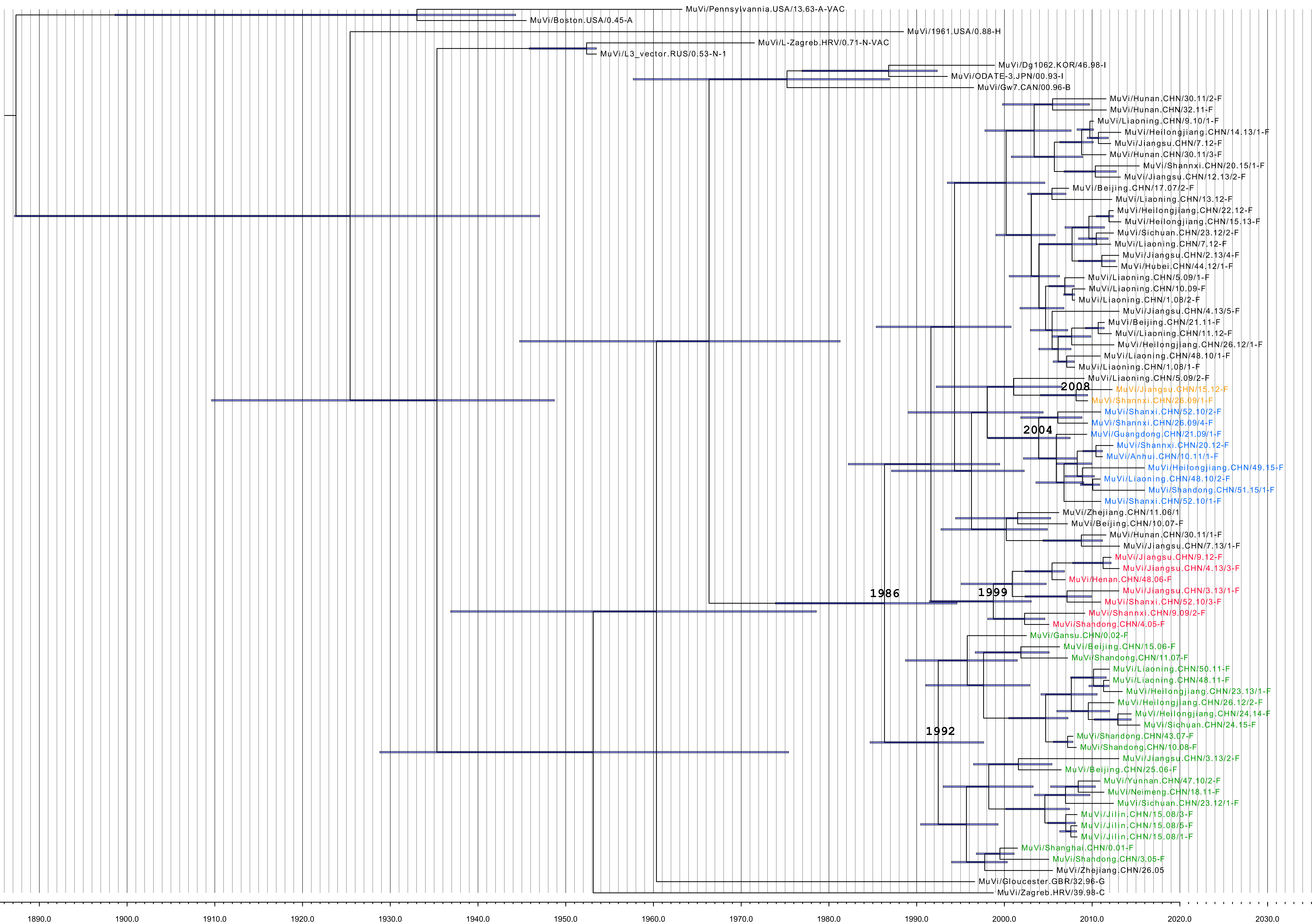
MuVi/Pennsylvannia.USA/13.63-A-VAC
MuVi/Boston.USA/0.45-A
MuVi/1961.USA/0.88-H
MuVi/L-Zagreb.HRV/0.71-N-VAC
MuVi/L3_vector.RUS/0.53-N-1
MuVi/Dg1062.KOR/46.98-I
MuVi/ODATE-3.JPN/00.93-I
MuVi/Gw7.CAN/00.96-B
MuVi/Hunan.CHN/30.11/2-F
MuVi/Hunan.CHN/32.11-F
MuVi/Liaoning.CHN/9.10/1-F
MuVi/Heilongjiang.CHN/14.13/1-F
MuVi/Jiangsu.CHN/7.12-F
MuVi/Hunan.CHN/30.11/3-F
MuVi/Shannxi.CHN/20.15/1-F
MuVi/Jiangsu.CHN/12.13/2-F
MuVi/Beijing.CHN/17.07/2-F
MuVi/Liaoning.CHN/13.12-F
MuVi/Heilongjiang.CHN/22.12-F
MuVi/Heilongjiang.CHN/15.13-F
MuVi/Sichuan.CHN/23.12/2-F
MuVi/Liaoning.CHN/7.12-F
MuVi/Jiangsu.CHN/2.13/4-F
MuVi/Hubei.CHN/44.12/1-F
MuVi/Liaoning.CHN/5.09/1-F
MuVi/Liaoning.CHN/10.09-F
MuVi/Liaoning.CHN/1.08/2-F
MuVi/Jiangsu.CHN/4.13/5-F
MuVi/Beijing.CHN/21.11-F
MuVi/Liaoning.CHN/11.12-F
MuVi/Heilongjiang.CHN/26.12/1-F
MuVi/Liaoning.CHN/48.10/1-F
MuVi/Liaoning.CHN/1.08/1-F
MuVi/Liaoning.CHN/5.09/2-F
2008
MuVi/Jiangsu.CHN/15.12-F
MuVi/Shannxi.CHN/26.09/1-F
MuVi/Shanxi.CHN/52.10/2-F
MuVi/Shannxi.CHN/26.09/4-F
2004
MuVi/Guangdong.CHN/21.09/1-F
MuVi/Shannxi.CHN/20.12-F
MuVi/Anhui.CHN/10.11/1-F
MuVi/Heilongjiang.CHN/49.15-F
MuVi/Liaoning.CHN/48.10/2-F
MuVi/Shandong.CHN/51.15/1-F
MuVi/Shanxi.CHN/52.10/1-F
MuVi/Zhejiang.CHN/11.06/1
MuVi/Beijing.CHN/10.07-F
MuVi/Hunan.CHN/30.11/1-F
MuVi/Jiangsu.CHN/7.13/1-F
MuVi/Jiangsu.CHN/9.12-F
MuVi/Jiangsu.CHN/4.13/3-F
MuVi/Henan.CHN/48.06-F
1986
1999
MuVi/Jiangsu.CHN/3.13/1-F
MuVi/Shanxi.CHN/52.10/3-F
MuVi/Shannxi.CHN/9.09/2-F
MuVi/Shandong.CHN/4.05-F
MuVi/Gansu.CHN/0.02-F
MuVi/Beijing.CHN/15.06-F
MuVi/Shandong.CHN/11.07-F
MuVi/Liaoning.CHN/50.11-F
MuVi/Liaoning.CHN/48.11-F
MuVi/Heilongjiang.CHN/23.13/1-F
MuVi/Heilongjiang.CHN/26.12/2-F
MuVi/Heilongjiang.CHN/24.14-F
MuVi/Sichuan.CHN/24.15-F
1992
MuVi/Shandong.CHN/43.07-F
MuVi/Shandong.CHN/10.08-F
MuVi/Jiangsu.CHN/3.13/2-F
MuVi/Beijing.CHN/25.06-F
MuVi/Yunnan.CHN/47.10/2-F
MuVi/Neimeng.CHN/18.11-F
MuVi/Sichuan.CHN/23.12/1-F
MuVi/Jilin.CHN/15.08/3-F
MuVi/Jilin.CHN/15.08/5-F
MuVi/Jilin.CHN/15.08/1-F
MuVi/Shanghai.CHN/0.01-F
MuVi/Shandong.CHN/3.05-F
MuVi/Zhejiang.CHN/26.05
MuVi/Gloucester.GBR/32.96-G
MuVi/Zagreb.HRV/39.98-C
1890.0
1900.0
1910.0
1920.0
1930.0
1940.0
1950.0
1960.0
1970.0
1980.0
1990.0
2000.0
2010.0
2020.0
2030.0

Supplementary Figure S4: Time distribution of sequences analyzed in this study. The number of sequences for each gene is represented as a bar graph, SH in blue, HN in red and F in green.

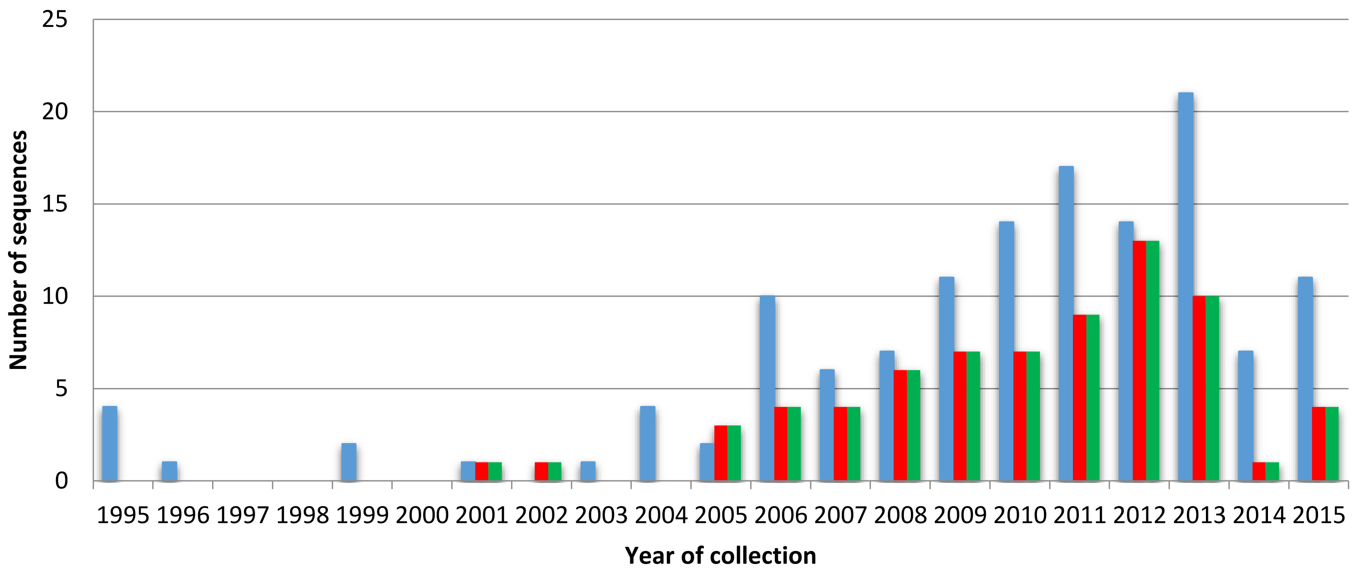

Supplementary Figure S4

Supplementary Figure S5: MCC tree for HN dataset with locations. Posterior probabilities are shown at each node, low values in green and high values in red. Average value is 0.78. Forty nodes have a value > 0.9.

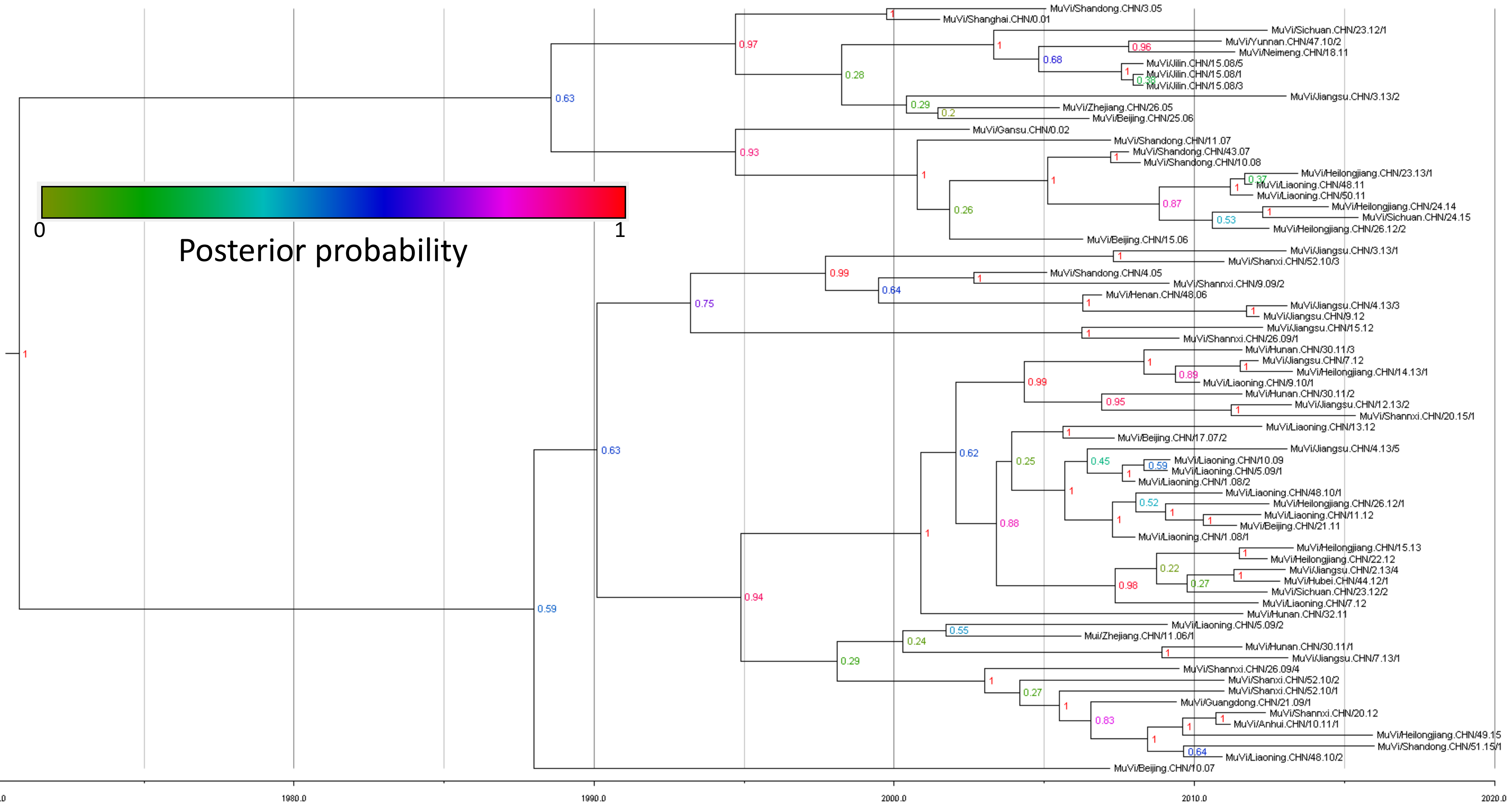

Supplementary Figure S5

Supplementary figure S6: Bayes Factor (BF) for each geographic links. All BF values are greater than 3 and show a well-supported diffusion route in the migration graph. The higher the BF value is, the more likely that a migration may exist between two locations

BF cutoff = 3.0			
mean Poisson Prior = 0.6931471805599453			
Poisson Prior offset = 18.0			
0	pk=0.99906785068794	BF=8732.65935192681	between Henan (long: 113.7536; lat: 34.765514) and Heilongjiang (long: 126.66167; lat: 45.742367)
1	pk=0.998173047928391	BF=4451.595592410126	between Liaoning (long: 123.43138; lat: 41.836174) and Sichuan (long: 104.07588; lat: 30.651226)
2	pk=0.9981151420831522	BF=4314.585155311667	between Shannxi (long: 108.954346; lat: 34.265503) and Heilongjiang (long: 126.66167; lat: 45.742367)
3	pk=0.9980686131571169	BF=4210.446453232117	between Shannxi (long: 108.954346; lat: 34.265503) and Anhui (long: 117.284904; lat: 31.86112)
4	pk=0.9978089716413354	BF=3710.534078401733	between Yunnan (long: 102.71; lat: 25.045807) and Heilongjiang (long: 126.66167; lat: 45.742367)
5	pk=0.9973927005129827	BF=3116.8240412076148	between Liaoning (long: 123.43138; lat: 41.836174) and Neimeng (long: 111.76629; lat: 40.81739)
6	pk=0.9973417792954992	BF=3056.9616801573966	between Sichuan (long: 104.07588; lat: 30.651226) and Hubei (long: 114.34174; lat: 30.546558)
7	pk=0.996417205242179	BF=2265.9812222910837	between Liaoning (long: 123.43138; lat: 41.836174) and Jiangsu (long: 118.76323; lat: 32.061707)
8	pk=0.9962913809323537	BF=2188.82562836867	between Shanghai (long: 121.4737; lat: 31.230415) and Henan (long: 113.7536; lat: 34.765514)
9	pk=0.9961988574464443	BF=2135.349171914024	between Shanxi (long: 112.562675; lat: 37.8735) and Anhui (long: 117.284904; lat: 31.86112)
10	pk=0.9950236818946145	BF=1629.1546100304797	between Shanghai (long: 121.4737; lat: 31.230415) and Jiangsu (long: 118.76323; lat: 32.061707)
11	pk=0.9948675397142481	BF=1579.3438089331396	between Henan (long: 113.7536; lat: 34.765514) and Sichuan (long: 104.07588; lat: 30.651226)
12	pk=0.9946035639694498	BF=1501.6889899174926	between Henan (long: 113.7536; lat: 34.765514) and Shanxi (long: 112.562675; lat: 37.8735)
13	pk=0.9942974137219975	BF=1420.6315775223718	between Gansu (long: 103.82645; lat: 36.059563) and Anhui (long: 117.284904; lat: 31.86112)
14	pk=0.9940724791888731	BF=1366.412989623441	between Shannxi (long: 108.954346; lat: 34.265503) and Hubei (long: 114.34174; lat: 30.546558)
15	pk=0.9928858081170284	BF=1137.1316491726682	between Yunnan (long: 102.71; lat: 25.045807) and Shanxi (long: 112.562675; lat: 37.8735)
16	pk=0.9927373881756337	BF=1113.726529425972	between Neimeng (long: 111.76629; lat: 40.81739) and Heilongjiang (long: 126.66167; lat: 45.742367)
17	pk=0.9925161582438159	BF=1080.5627913853837	between Liaoning (long: 123.43138; lat: 41.836174) and Guangdong (long: 113.26653; lat: 23.13219)
18	pk=0.9920055927447441	BF=1011.0319285355791	between Anhui (long: 117.284904; lat: 31.86112) and Hunan (long: 112.98381; lat: 28.11245)
19	pk=0.9911136934858492	BF=908.7390819850561	between Gansu (long: 103.82645; lat: 36.059563) and Liaoning (long: 123.43138; lat: 41.836174)
20	pk=0.9907285924498782	BF=870.6548901218688	between Neimeng (long: 111.76629; lat: 40.81739) and Hubei (long: 114.34174; lat: 30.546558)
21	pk=0.9906947544883581	BF=867.4591776117808	between Beijing (long: 116.407394; lat: 39.90421) and Sichuan (long: 104.07588; lat: 30.651226)
22	pk=0.9884581105016245	BF=697.7797727544398	between Henan (long: 113.7536; lat: 34.765514) and Jilin (long: 125.32586; lat: 43.89654)
23	pk=0.9880185875333269	BF=671.883714548308	between Beijing (long: 116.407394; lat: 39.90421) and Shannxi (long: 108.954346; lat: 34.265503)
24	pk=0.9867920069201047	BF=608.7315467517744	between Zhejiang (long: 120.152794; lat: 30.267443) and Hubei (long: 114.34174; lat: 30.546558)
25	pk=0.9862784933870207	BF=585.6454622872676	between Gansu (long: 103.82645; lat: 36.059563) and Neimeng (long: 111.76629; lat: 40.81739)
26	pk=0.9853081203045794	BF=546.4264549596745	between Liaoning (long: 123.43138; lat: 41.836174) and Heilongjiang (long: 126.66167; lat: 45.742367)
27	pk=0.9844360934040792	BF=515.3543323530354	between Shanghai (long: 121.4737; lat: 31.230415) and Heilongjiang (long: 126.66167; lat: 45.742367)
28	pk=0.9835921119614411	BF=488.42667225782844	between Shandong (long: 117.020355; lat: 36.66853) and Shanxi (long: 112.562675; lat: 37.8735)
29	pk=0.9827989365823279	BF=465.52863473496865	between Guangdong (long: 113.26653; lat: 23.13219) and Yunnan (long: 102.71; lat: 25.045807)
30	pk=0.9825427108520873	BF=458.5763483119232	between Jiangsu (long: 118.76323; lat: 32.061707) and Sichuan (long: 104.07588; lat: 30.651226)
31	pk=0.9823678839002742	BF=453.94865933991053	between Liaoning (long: 123.43138; lat: 41.836174) and Shannxi (long: 108.954346; lat: 34.265503)
32	pk=0.9818151963548848	BF=439.9042446635311	between Shanghai (long: 121.4737; lat: 31.230415) and Zhejiang (long: 120.152794; lat: 30.267443)
33	pk=0.9808267729395405	BF=416.8061470463381	between Jilin (long: 125.32586; lat: 43.89654) and Sichuan (long: 104.07588; lat: 30.651226)
34	pk=0.9807319260031114	BF=414.71431503127496	between Jilin (long: 125.32586; lat: 43.89654) and Anhui (long: 117.284904; lat: 31.86112)
35	pk=0.9805926613321272	BF=411.6799090470468	between Shanghai (long: 121.4737; lat: 31.230415) and Shanxi (long: 112.562675; lat: 37.8735)
36	pk=0.9795853443513606	BF=390.9644228564167	between Shandong (long: 117.020355; lat: 36.66853) and Anhui (long: 117.284904; lat: 31.86112)
37	pk=0.9789771496898925	BF=379.4180396798878	between Zhejiang (long: 120.152794; lat: 30.267443) and Anhui (long: 117.284904; lat: 31.86112)
38	pk=0.9785280580040944	BF=371.31199281270125	between Anhui (long: 117.284904; lat: 31.86112) and Jiangsu (long: 118.76323; lat: 32.061707)
39	pk=0.9782267328550265	BF=366.06056412335033	between Beijing (long: 116.407394; lat: 39.90421) and Shanxi (long: 112.562675; lat: 37.8735)
40	pk=0.9776837053119677	BF=356.9548672609006	between Yunnan (long: 102.71; lat: 25.045807) and Sichuan (long: 104.07588; lat: 30.651226)
41	pk=0.9764200485989842	BF=337.38890847863127	between Gansu (long: 103.82645; lat: 36.059563) and Henan (long: 113.7536; lat: 34.765514)
42	pk=0.9725066660220024	BF=288.2054558482582	between Beijing (long: 116.407394; lat: 39.90421) and Heilongjiang (long: 126.66167; lat: 45.742367)
43	pk=0.9708160520835665	BF=271.0378388933285	between Hunan (long: 112.98381; lat: 28.11245) and Sichuan (long: 104.07588; lat: 30.651226)
44	pk=0.9701447947489562	BF=264.76069679460727	between Liaoning (long: 123.43138; lat: 41.836174) and Yunnan (long: 102.71; lat: 25.045807)
45	pk=0.9701110447684193	BF=264.4525343222756	between Beijing (long: 116.407394; lat: 39.90421) and Anhui (long: 117.284904; lat: 31.86112)
46	pk=0.9697986824355563	BF=261.6331190233895	between Heilongjiang (long: 126.66167; lat: 45.742367) and Sichuan (long: 104.07588; lat: 30.651226)
47	pk=0.9688809230010244	BF=253.67678001729033	between Gansu (long: 103.82645; lat: 36.059563) and Sichuan (long: 104.07588; lat: 30.651226)
48	pk=0.9687790417689739	BF=252.8223858565009	between Zhejiang (long: 120.152794; lat: 30.267443) and Beijing (long: 116.407394; lat: 39.90421)
49	pk=0.9657437783936759	BF=229.69919772405672	between Shandong (long: 117.020355; lat: 36.66853) and Guangdong (long: 113.26653; lat: 23.13219)
50	pk=0.9643483876692515	BF=220.38995796492952	between Shanghai (long: 121.4737; lat: 31.230415) and Anhui (long: 117.284904; lat: 31.86112)
51	pk=0.9623599414245754	BF=208.31678541563977	between Jilin (long: 125.32586; lat: 43.89654) and Shanxi (long: 112.562675; lat: 37.8735)
52	pk=0.9618151313111298	BF=205.22833532440677	between Shandong (long: 117.020355; lat: 36.66853) and Yunnan (long: 102.71; lat: 25.045807)
53	pk=0.9611414665275299	BF=201.52917498676987	between Shanghai (long: 121.4737; lat: 31.230415) and Neimeng (long: 111.76629; lat: 40.81739)
54	pk=0.9602142466022581	BF=196.6425863375078	between Shandong (long: 117.020355; lat: 36.66853) and Zhejiang (long: 120.152794; lat: 30.267443)
55	pk=0.9601580741938285	BF=196.35385617525097	between Shanghai (long: 121.4737; lat: 31.230415) and Jilin (long: 125.32586; lat: 43.89654)
56	pk=0.9590017845769032	BF=190.5862131996415	between Jilin (long: 125.32586; lat: 43.89654) and Jiangsu (long: 118.76323; lat: 32.061707)
57	pk=0.9575023342918303	BF=183.57425915964137	between Yunnan (long: 102.71; lat: 25.045807) and Jiangsu (long: 118.76323; lat: 32.061707)
58	pk=0.9574874882917904	BF=183.50730699302173	between Jilin (long: 125.32586; lat: 43.89654) and Guangdong (long: 113.26653; lat: 23.13219)
59	pk=0.9562077924243838	BF=177.90676338614313	between Beijing (long: 116.407394; lat: 39.90421) and Hubei (long: 114.34174; lat: 30.546558)
60	pk=0.9539503016774217	BF=168.78582819952217	between Shannxi (long: 108.954346; lat: 34.265503) and Neimeng (long: 111.76629; lat: 40.81739)
61	pk=0.9522789396247612	BF=162.5889825108946	between Liaoning (long: 123.43138; lat: 41.836174) and Hunan (long: 112.98381; lat: 28.11245)
62	pk=0.9520733625705996	BF=161.85662251961963	between Shanxi (long: 112.562675; lat: 37.8735) and Jiangsu (long: 118.76323; lat: 32.061707)
63	pk=0.9499212807803716	BF=154.55085885612655	between Shanghai (long: 121.4737; lat: 31.230415) and Sichuan (long: 104.07588; lat: 30.651226)
64	pk=0.9491459898055814	BF=152.0704496860268	between Shannxi (long: 108.954346; lat: 34.265503) and Yunnan (long: 102.71; lat: 25.045807)
65	pk=0.9478875997232378	BF=148.2015628862917	between Anhui (long: 117.284904; lat: 31.86112) and Hubei (long: 114.34174; lat: 30.546558)
66	pk=0.947784003092662	BF=147.8913655970876	between Gansu (long: 103.82645; lat: 36.059563) and Shanxi (long: 112.562675; lat: 37.8735)
67	pk=0.9477433352855802	BF=147.76993097876422	between Zhejiang (long: 120.152794; lat: 30.267443) and Guangdong (long: 113.26653; lat: 23.13219)
68	pk=0.9469716703173872	BF=145.50102672956712	between Shandong (long: 117.020355; lat: 36.66853) and Sichuan (long: 104.07588; lat: 30.651226)
69	pk=0.9464108381196349	BF=143.8930304296165	between Beijing (long: 116.407394; lat: 39.90421) and Liaoning (long: 123.43138; lat: 41.836174)
70	pk=0.9435613441001436	BF=136.21674453123993	between Shanghai (long: 121.4737; lat: 31.230415) and Beijing (long: 116.407394; lat: 39.90421)
71	pk=0.9428595548950456	BF=134.44368457741513	between Guangdong (long: 113.26653; lat: 23.13219) and Sichuan (long: 104.07588; lat: 30.651226)
72	pk=0.9415769822870486	BF=131.31334556012527	between Guangdong (long: 113.26653; lat: 23.13219) and Hunan (long: 112.98381; lat: 28.11245)
73	pk=0.9401459614033406	BF=127.97903885771855	between Yunnan (long: 102.71; lat: 25.045807) and Hunan (long: 112.98381; lat: 28.11245)
74	pk=0.934745849939969	BF=116.71385857713378	between Shannxi (long: 108.954346; lat: 34.265503) and Guangdong (long: 113.26653; lat: 23.13219)
75	pk=0.9319045791774451	BF=111.50402805570852	between Shanghai (long: 121.4737; lat: 31.230415) and Liaoning (long: 123.43138; lat: 41.836174)
76	pk=0.9285771001111562	BF=105.92964301764022	between Guangdong (long: 113.26653; lat: 23.13219) and Anhui (long: 117.284904; lat: 31.86112)
77	pk=0.9273302682893029	BF=103.97235895171357	between Guangdong (long: 113.26653; lat: 23.13219) and Shanxi (long: 112.562675; lat: 37.8735)
78	pk=0.9209335549408194	BF=94.90150574860992	between Shanghai (long: 121.4737; lat: 31.230415) and Guangdong (long: 113.26653; lat: 23.13219)
79	pk=0.9189413789332983	BF=92.36886669155301	between Guangdong (long: 113.26653; lat: 23.13219) and Neimeng (long: 111.76629; lat: 40.81739)
80	pk=0.9050642804094908	BF=77.67599004727973	between Beijing (long: 116.407394; lat: 39.90421) and Yunnan (long: 102.71; lat: 25.045807)
81	pk=0.9048320197034959	BF=77.46653430968614	between Shandong (long: 117.020355; lat: 36.66853) and Beijing (long: 116.407394; lat: 39.90421)
82	pk=0.8861048409480482	BF=63.38943233203992	between Henan (long: 113.7536; lat: 34.765514) and Anhui (long: 117.284904; lat: 31.86112)
83	pk=0.8631782276292245	BF=51.402268315642004	between Jilin (long: 125.32586; lat: 43.89654) and Yunnan (long: 102.71; lat: 25.045807)
84	pk=0.8317261331714694	BF=40.27176755900319	between Gansu (long: 103.82645; lat: 36.059563) and Yunnan (long: 102.71; lat: 25.045807)

Supplementary figure S6
